# Iron-Catalyzed Transfer Hydrogenation of Allylic Alcohols with Isopropanol

**DOI:** 10.1021/acs.joc.4c01701

**Published:** 2024-09-25

**Authors:** Md Abdul Bari, Salma A. Elsherbeni, Tahir Maqbool, Daniel E. Latham, Edward B. Gushlow, Emily J. Harper, Louis C. Morrill

**Affiliations:** † Cardiff Catalysis Institute, School of Chemistry, Cardiff University, Main Building, Park Place, Cardiff CF10 3AT, United Kingdom; ‡ Department of Pharmaceutical Chemistry, Faculty of Pharmacy, Tanta University, Tanta 31527, Egypt; § Department of Chemistry, Government College University Faisalabad, Faisalabad 38000, Pakistan; ∥ Department of Chemistry, University of Bath, Claverton Down, Bath BA2 7AY, United Kingdom

## Abstract

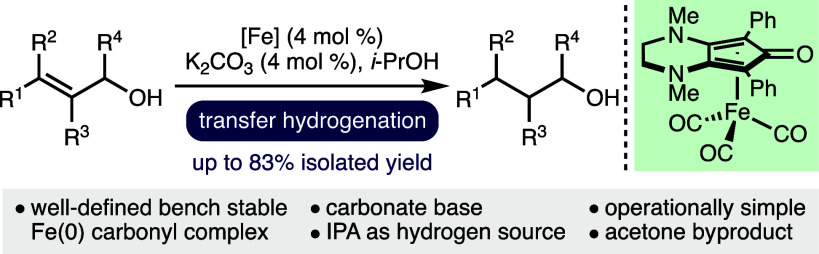

Herein, we report an iron-catalyzed transfer hydrogenation of allylic alcohols. The operationally simple protocol employs a well-defined bench stable (cyclopentadienone)iron(0) carbonyl complex as a precatalyst in combination with K_2_CO_3_ (4 mol %) and isopropanol as the hydrogen donor. A diverse range of allylic alcohols undergo transfer hydrogenation to form the corresponding alcohols in good yields (33 examples, ≤83% isolated yield). The scope and limitations of the method have been investigated, and experiments that shed light on the reaction mechanism have been conducted.

The borrowing hydrogen approach, which combines transfer hydrogenation with a reaction on the *in situ*-generated reactive intermediate, has undergone a renaissance over the past decade.^1^ Most commonly, this strategy has been utilized to diversify the synthetic utility of commodity alcohols, particularly benzylic alcohols and aliphatic alcohols, which can be employed as alkylating agents whereby the sole byproduct of this one-pot reaction is water.^2^ Allylic alcohols, which are privileged motifs in synthetic chemistry due to their widespread availability and diverse reactivity profile,^3^ have received comparatively less attention in this domain. Dehydrogenation of allylic alcohols generates synthetically versatile α,β-unsaturated carbonyl compounds, which can undergo either 1,2- or 1,4-addition by nucleophiles (Scheme 1A). The 1,2-addition pathway has been utilized for the N-allylation of amines^4^ and sulfinamides^5^ and the C-allylation of ketones^6^ and oxindoles.^7^ Conversely, the 1,4-addition pathway has enabled various regioselective alkene hydrofunctionalization methods.^8^

**Scheme 1:**
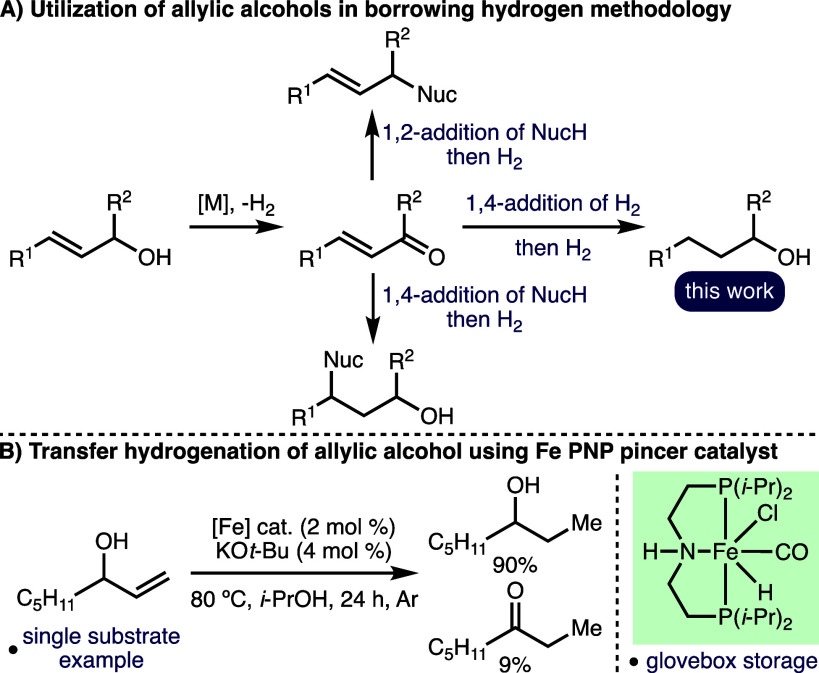
Background and Context

The 1,4-addition of hydrogen results in the redox isomerization of allylic alcohols to form carbonyl compounds,^9,10^ which can undergo further hydrogenation in the presence of a hydrogen donor to complete the formal transfer hydrogenation of allylic alcohols.^11^ This powerful one-pot transformation has been predominantly explored using a variety of heterogeneous and homogeneous catalyst systems based on precious metals (e.g., Rh, Ru, Pd, and Ir),^12^ alongside a few examples that employ catalysts based on more earth-abundant transition metals (e.g., Fe, Ni, and Mo).^13^ Most pertinent to this study, in 2018, de Vries and co-workers reported the isomerization of secondary allylic alcohols to ketones catalyzed by a well-defined iron PNP pincer catalyst, employing KO*t*-Bu as the base and toluene as the solvent.^14^ During optimization of the reaction with a model substrate, the authors observed competitive transfer hydrogenation when 2-propanol was employed as the reaction solvent (Scheme 1B). Building upon our interest in the development of borrowing hydrogen transformations that employ catalysts based on earth-abundant 3d transition metals,^15^ herein, we report the use of an air stable phosphine-free (cyclopentadienone)iron(0) carbonyl complex^16,17^ as a precatalyst for the transfer hydrogenation of allylic alcohols, with isopropanol as the hydrogen donor.^18^

The transfer hydrogenation of (*E*)-2-methyl-3-phenylprop-2-en-1-ol (**1**) to form 2-methyl-3-phenylpropan-1-ol (**2**) was selected as the model system for reaction optimization due to facile determination of conversion data via ^1^H nuclear magnetic resonance (NMR) analysis of the crude reaction mixtures (Table 1).^19^ It was found that Renaud’s (cyclopentadienone)iron(0) carbonyl complex **3** (4 mol %),^20^ Me_3_NO (8 mol %), and K_2_CO_3_ (4 mol %) in *i*-PrOH ([**1**] = 0.5 M) at 130 °C for 18 h under N_2_ in a sealed tube enabled the transfer hydrogenation of **1**, which formed product **2** in 93% NMR yield (entry 1). Control experiments confirmed that no product was formed in the absence of iron precatalyst **3** or K_2_CO_3_ (entry 2 or 3, respectively). It was found that precatalyst **4**, which contains a less electron-rich cyclopentadienone framework, gave product **2** in a reduced NMR yield of 72% (entry 4). A selection of other structurally related (cyclopentadienone)iron carbonyl precatalysts **5**–**7** did not enable product formation in significant quantities (entry 5). Substituting K_2_CO_3_ for KOH or Na_2_CO_3_ as the base decreased the observed NMR yield of **2** (entry 6 or 7, respectively). Altering the concentration (entries 8 and 9), decreasing the reaction temperature (entry 10), reducing the reaction time (entry 11), and decreasing the catalyst loading (entry 12) all reduced the efficiency of the transfer hydrogenation of **1** to form **2**. The quantity of K_2_CO_3_ could be decreased to 2 mol % without a significant reduction in conversion (entry 13); however, a small amount of unreacted **1** (7%) was observed, which is challenging to separate from **2** via silica gel flash chromatography. Furthermore, it was found that Me_3_NO was not required (entry 14) and that the NMR yield of **2** could be increased to >98% by extending the reaction time to 24 h (entry 15).

**Table 1:**
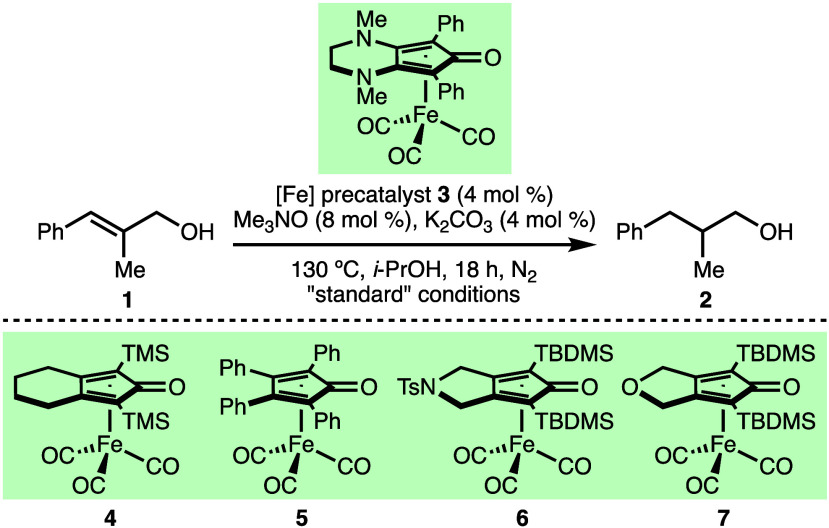
Reaction Optimization^a^

entry	variation from the standard conditions	yield^b^ (%)
1	none	93
2	no [Fe] precatalyst	<2
3	no K_2_CO_3_	<2
4	[Fe] precatalyst **4** (4 mol %) instead of **3**	72
5	[Fe] precatalysts **5**–**7** (4 mol %) instead of **3**	<7
6	KOH (4 mol %) instead of K_2_CO_3_	44
7	Na_2_CO_3_ (4 mol %) instead of K_2_CO_3_	86
8	[**1**] = 0.25 M	80
9	[**1**] = 1 M	79
10	110 °C	36
11	8 h	86
12^c^	[Fe] precatalyst **3** (2 mol %)	78
13	K_2_CO_3_ (2 mol %)	92
14	no Me_3_NO	94
15	no Me_3_NO, 24 h	>98 (80)

a Performed using 1 mmol of **1** and reagent grade *i*-PrOH. [**1**] = 0.5 M.

b Determined by ^1^H NMR analysis of the crude reaction mixture with 1,3,5-trimethylbenzene as the internal standard. Isolated yield in parentheses.

c With 4 mol % Me_3_NO.

With the optimized reaction conditions in hand (Table 1, entry 15), we attempted to establish the scope and limitations of this synthetic method (Scheme 2). Initially, it was found that the reaction could be performed successfully on an increased scale (Scheme 2A), using 5.5 mmol of allylic alcohol **1**, which provided access to 0.66 g of product **2** (80% isolated yield). Next, the impact of various substituents on the aromatic ring within the allylic alcohol scaffold upon conversion was investigated. It was found that methyl substituents could be incorporated at positions 4, 3, and 2 of the aromatic ring, providing access to hydrogenated products **8**–**10**, respectively, in high isolated yields (71–83%). At position 4, fluorine and chlorine substituents were tolerated, which gave **11** and **12** in 61% and 76% isolated yields, respectively. However, the incorporation of an aryl bromide motif resulted in reduced conversion (33%) to the corresponding product **13**. A selection of electron-releasing (4-OMe and 4-NMe_2_) and electron-withdrawing (4-CF_3_) aromatic substituents could be present within the allylic alcohol substrates to afford products **14**–**16**, respectively, in high yields. In some cases, the reaction time was extended to 48 h to ensure full consumption of allylic alcohol starting materials and to facilitate product purification via silica gel flash chromatography. Benzylic alcohol and styrene functionalities were preserved during the transfer hydrogenation process, as demonstrated by the formation of hydrogenated products **17** and **18**. Extended aromatic systems (1-naphthyl and 2-naphthyl) and various heteroaromatics (indole, furans, thiophenes, and pyridines) were well tolerated, which provided access to **19**–**27** in good yields. It was found that the presence of an aromatic ring at position 3 within the allylic alcohol scaffold was not essential for reactivity. Allylic alcohols bearing benzyl, homobenzyl, and cyclohexyl groups at position 3 all successfully underwent transfer hydrogenation to give reduced products **28**–**30**, respectively, in 58–72% isolated yields. Furthermore, cinnamyl alcohol and a selection of derivatives containing 4-F, 4-OMe, and 4-CF_3_ aromatic substituents could be converted into hydrogenated products **31**–**34**, respectively, with high conversion (66–88%). These results demonstrated that the 2-methyl substituent within the allylic alcohols was also not required for successful transfer hydrogenation. 2-Methylprop-2-en-1-ol was converted into aliphatic alcohol **35** in 62% NMR yield using the optimized reaction conditions. A 3,3-disubstituted cinnamyl alcohol derivative gave only 24% conversion to product **36** after 48 h, whereas the naturally occurring monoterpenoid geraniol gave **37** in 61% NMR yield using the same reaction conditions. Within geraniol, the allylic alcohol functionality underwent selective hydrogenation, with the other alkene left untouched. Gratifyingly, a secondary allylic alcohol gave 76% conversion to hydrogenated product **38**. It was found that an allylic alcohol, which contained a 4-CO_2_Me aromatic substituent, underwent both transfer hydrogenation and transesterification, giving 88% conversion to product **39** (Scheme 2B). Allylic alcohols that contained a 2-phenyl substitution or those that contained two methyl substituents at position 1, 2, or 3 were found to be incompatible with the transfer hydrogenation protocol (Scheme 2C), with significant quantities of recovered starting materials and/or complex reaction mixtures observed in each case. This indicated that the transfer hydrogenation protocol is somewhat sensitive to the degree and type of substitution on the allylic alcohol scaffold.

**Scheme 2:**
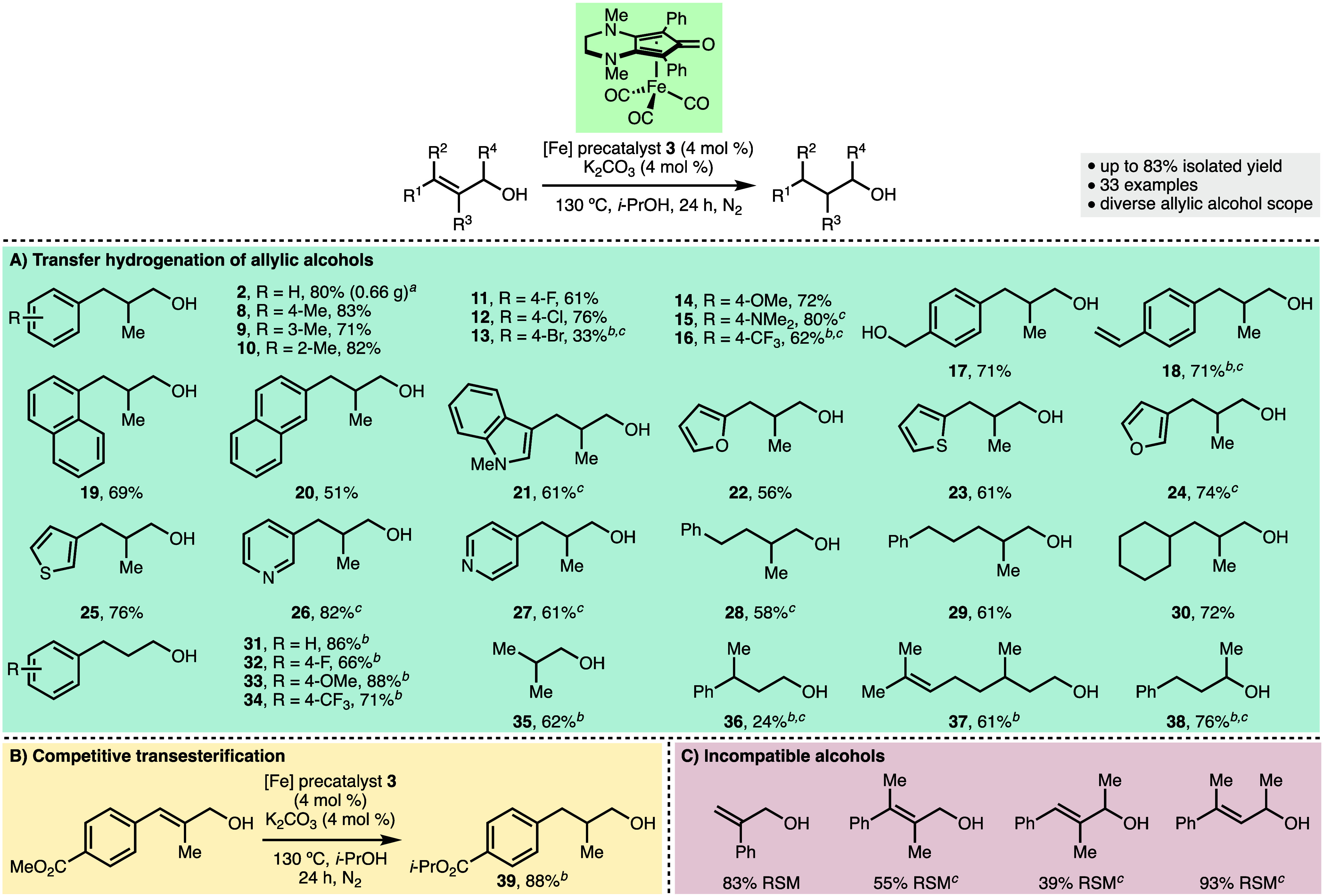
Scope of Iron-Catalyzed Transfer Hydrogenation of Allylic Alcohols With 5.5 mmol of allylic alcohol as the starting material. As determined by ^1^H NMR analysis of the crude reaction mixture with 1,3,5-trimethylbenzene as the internal standard. For 48 h. Reactions performed using 1 mmol of allylic alcohol starting material and reagent grade *i*-PrOH. Isolated yields after chromatographic purification unless stated otherwise. RSM = recovered starting material.

A range of experiments were performed to gain insight into the reaction mechanism (Scheme 3). First, it was found that alkene **40** and tertiary allylic alcohol **41** were both unreactive when subjected to the optimized reaction conditions, with no observable formation of reduced products **42** and **43** (Scheme 3A). In combination with the previous observation of preserved alkenes within products **18** and **37** (cf., Scheme 2), this confirmed that primary or secondary allylic alcohol functionalities are required for successful transfer hydrogenation to occur. Next, employing the optimized reaction conditions, enal **44** and aldehyde **45** were each converted into hydrogenated alcohol **2** in 90% and 43% NMR yields, respectively, which validated both **44** and **45** as plausible reaction intermediates (Scheme 3B). The progress of the reaction with time was monitored for the transfer hydrogenation of allylic alcohol **1**.^19^ Product **2** was initially formed slowly, with an only 13% conversion to **2** observed after 2 h. Beyond 2 h, the rate of formation of **2** increased, with 46% conversion observed after 4 h and 86% conversion after 8 h. Trace quantities of enal **44** (<2%) were observed throughout the reaction monitoring, until 18 h. Conversion to **2** reached >98% at 24 h. The initial slow formation of product **2** during the reaction over the first 2 h may be attributed to activation of precatalyst **3**.^18^ When toluene was employed as the reaction solvent (no *i*-PrOH hydrogen donor), a complex mixture of products that consisted of allylic alcohol **1** (35%), enal **44** (33%), aldehyde **45** (2%), and alcohol **2** (20%) was observed. Further mechanistic information was provided by deuterium labeling studies (Scheme 3C). Subjecting allylic alcohol **1** to the standard reaction conditions, except for isopropanol-*d*_8_ as the solvent, resulted in the formation of product **46** with significant deuterium incorporation at positions 1–3. The reaction of allylic alcohol **47**, which was deuterated at position 1 (75% D), also formed product **46** with deuterium incorporation at positions 1 and 3. Altogether, the incorporation of deuterium at positions 1 and 3 indicated the involvement of an iron hydride species in the reaction mechanism, which would be formed upon dehydrogenation of allylic alcohol **1** or isopropanol. The incorporation of deuterium at position 2 can be explained by the protonation of enolate intermediates. In line with these observations and related previous works,^18^ the proposed mechanism proceeds via initial conversion of precatalyst **3** to **48** in the presence of K_2_CO_3_ and *i*-PrOH (Scheme 4). Complex **48** promotes dehydrogenation of allylic alcohol **1** and isopropanol in the presence of K_2_CO_3_ to form enal **44** and acetone, respectively. Hydrogenation of **44** by iron–hydrogen complex **49** gives aldehyde **45**, which can undergo further hydrogenation to form alcohol **2** with the regeneration of **48**.

**Scheme 3:**
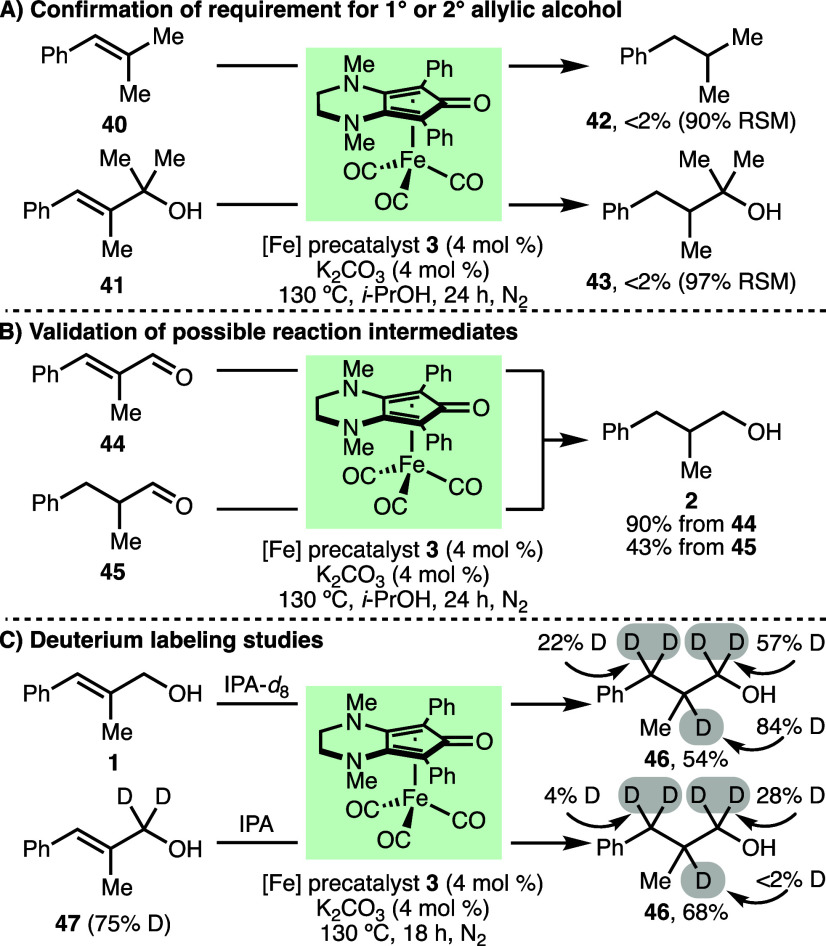
Mechanistic Experiments Yields and deuterium incorporation determined by ^1^H NMR analysis of the crude reaction mixture with 1,3,5-trimethylbenzene as the internal standard.

**Scheme 4:**
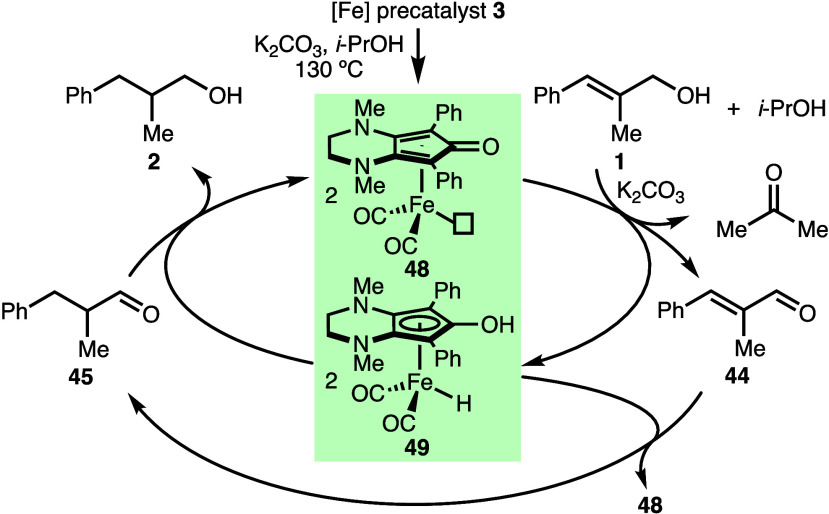
Plausible Mechanism

In summary, an operationally simple and efficient iron-catalyzed transfer hydrogenation of allylic alcohols has been developed (33 examples, ≤83% isolated yield). The protocol employs a bench stable precatalyst based on an earth-abundant transition metal, a carbonate base, and isopropyl alcohol as the hydrogen donor.

## Data Availability

The data underlying this study are openly available in the Cardiff University data catalogue at 10.17035/d.2024.0324584208.

## Supporting Information Available

Optimization data, experimental procedures, characterization of new compounds, and spectral data (PDF)
