# Supplementary material for: Iron-Catalyzed Transfer Hydrogenation of Allylic Alcohols with Isopropanol
Source: J Org Chem. 2024 Sep 25;89(19):14571–6. doi: 10.1021/acs.joc.4c01701 (PMC11459429; doi:10.1021/acs.joc.4c01701)
Supplement: Supplementary file 1 — jo4c01701_si_001.pdf [file jo4c01701_si_001.pdf]

SUPPORTING INFORMATION

Iron-Catalyzed Transfer Hydrogenation of Allylic Alcohols with Isopropanol

Md Abdul Bari,[1] Salma A. Elsherbeni,[1,2] Tahir Maqbool,[1,3] Daniel E. Latham,[1] Edward B. Gushlow,[1] Emily J. Harper,[1] and Louis C. Morrill*,[1,4]

[1] Cardiff Catalysis Institute, School of Chemistry, Cardiff University, Main Building, Park Place, Cardiff, CF10 3AT, United Kingdom.

[2] Department of Pharmaceutical Chemistry, Faculty of Pharmacy, Tanta University, Tanta, Egypt.

[3] Department of Chemistry, Government College University Faisalabad, Faisalabad 38000, Pakistan.

[4] Department of Chemistry, University of Bath, Claverton Down, Bath, BA2 7AY, United Kingdom.

Table of Contents

1. General information........ S2
2. Experimental and characterization data........ S3
 2.1. Synthesis of substrates........ S3
  2.1.1. General procedure 1........ S3
  2.1.2. General procedure 2........ S7
  2.1.3. General procedure 3........ S9
  2.1.4. General procedure 4........ S12
  2.1.5. General procedure 5........ S51
  2.1.7. General procedure 6........ S59
 2.2. Optimization of reaction conditions........ S71
 2.3. Scope of the iron-catalyzed TH protocol........ S73
  2.3.1. General procedure 7........ S73
 2.4. Validation of possible reaction intermediates........ S122
  2.4.1. Synthesis of aldehyde 45........ S122
  2.4.2. Mechanistic experiments........ S124
 2.5. Kinetic time course experiments........ S127
 2.6. Deuterium labelling experiments........ S128
 2.7. References........ S133

1. General information

Unless stated otherwise, all reactions were performed using oven-dried 20 mL microwave vials sealed with an aluminium crimp caps, and were stirred with Teflon-coated magnetic stirrer bars. Dry tetrahydrofuran (THF), toluene, hexanes and diethyl ether were obtained after passing these previously degassed solvents through activated alumina columns (Mbraun, SPS-800). All other solvents and commercial reagents were used as supplied without further purification unless stated otherwise. Isopropyl alcohol was supplied as reagent grade from ThermoScientific (99.5%) and was not degassed before use.

Room temperature (rt) refers to 20–25 °C. Ice/water and CO_2(s)/acetone baths were used to obtain temperatures of 0 °C and –78 °C respectively. All reactions involving heating were carried out using DrySyn blocks and a contact thermometer. In vacuo refers to reduced pressure through the use of a rotary evaporator. [Fe] precatalysts **3**,[1] **4**,[2] **5**,[3] **6**,[4] and **7**[4] were prepared according to the corresponding literature procedures.

Analytical thin layer chromatography was carried out using aluminium plates coated with silica (Kieselgel 60 F254 silica) and visualization was achieved using ultraviolet light (254 nm), followed by staining with a 1% aqueous KMnO4 solution. Flash chromatography used Kieselgel 60 silica in the solvent system stated. Melting points were recorded on a Gallenkamp melting point apparatus, and corrected by linear interpolation of melting point standards benzophenone (47–49 °C), and benzoic acid (121–123 ˚C). Infrared spectra were recorded on a Shimadzu IRAffinity-1 Fourier Transform ATIR spectrometer as thin films using a Pike MIRacle ATR accessory. Characteristic peaks are quoted (νmax / cm^{-1}).

^{1}H, $^{13}C\{^{1}H\}$, $^{19}F\{^{1}H\}$ NMR spectra were obtained on either a Bruker Avance 300 (300 MHz ^{1}H, 75 MHz $^{13}C\{^{1}H\}$) or a Bruker Avance 400 (400 MHz ^{1}H, 101 MHz $^{13}C\{^{1}H\}$, 376 MHz $^{19}F\{^{1}H\}$) or a Bruker Avance 500 (500 MHz ^{1}H, 126 MHz $^{13}C\{^{1}H\}$, 471 MHz $^{19}F\{^{1}H\}$) spectrometer at rt in the solvent stated. Chemical shifts are reported in parts per million (ppm) relative to the residual solvent signal. All coupling constants, J, are quoted in Hz. Multiplicities are reported with the following symbols: s = singlet, d = doublet, t = triplet, q = quartet, hept = heptet, m = multiplet and multiples thereof. The abbreviation br to denote broad.

High resolution mass spectrometry (HRMS, m/z) data was acquired at Cardiff University on a Micromass LCT spectrometer.

2. Experimental and characterization data

2.1. Synthesis of substrates

2.1.1. General procedure 1

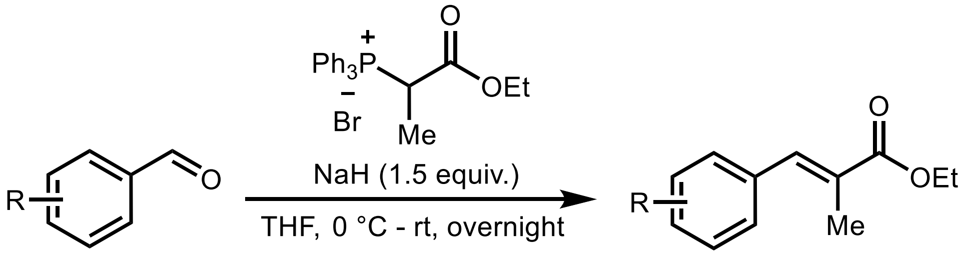

To a stirred solution of NaH (1.5 equiv., 60% in paraffin oil) in anhydrous THF (0.2 M solution) at 0 °C was added triphenylphosphonium bromide (1.5 equiv.). The reaction mixture was allowed to warm to rt and stirred for 1 h. Then the corresponding aldehyde was added to the reaction mixture and the mixture was stirred at rt overnight. The reaction was then quenched by the addition of H_2O and extracted with EtOAc (3×). The combined organics were washed with brine, dried over anhydrous $MgSO_4$, filtered and concentrated in vacuo.

Ethyl (*E*)-3-(4-(dimethylamino)phenyl)-2-methylacrylate

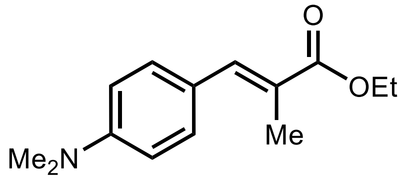

The title compound was prepared according to general procedure 1 using 4-(dimethylamino)benzaldehyde (2 g, 13.4 mmol). The crude ester was directly used in the next step.

Methyl (*E*)-4-(3-ethoxy-2-methyl-3-oxoprop-1-en-1-yl)benzoate

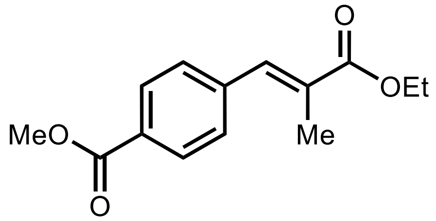

The title compound was prepared according to general procedure 1 using methyl 4-formylbenzoate (2 g, 12.1 mmol). The crude ester was directly used in the next step.

Ethyl (*E*)-3-(4-fluorophenyl)-2-methylacrylate

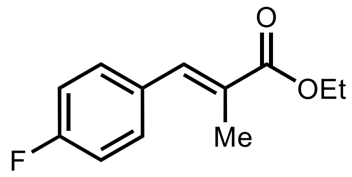

The title compound was prepared according to general procedure 1 using 4-fluorobenzaldehyde (2.5 g, 20 mmol). The crude ester was directly used in the next step.

Ethyl (*E*)-3-(4-chlorophenyl)-2-methylacrylate

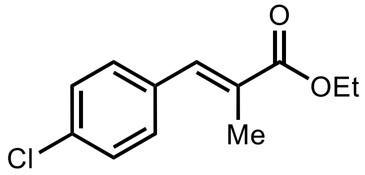

The title compound was prepared according to general procedure 1 using 4-chlorobenzaldehyde (2 g, 14.2 mmol). The crude ester was directly used in the next step.

Ethyl (*E*)-2-methyl-3-(naphthalen-2-yl)acrylate

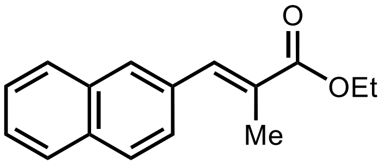

The title compound was prepared according to general procedure 1 using 2-naphthaldehyde (2 g, 12.8 mmol). The crude ester was directly used in the next step.

Ethyl (*E*)-2-methyl-3-(naphthalen-1-yl)acrylate

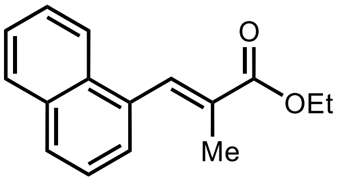

The title compound was prepared according to general procedure 1 using 1-naphthaldehyde (2 g, 12.8 mmol). The crude ester was directly used in the next step.

Ethyl (*E*)-2-methyl-3-(furan-2-yl)acrylate

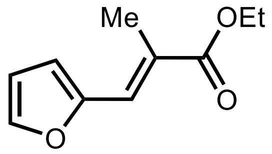

The title compound was prepared according to general procedure 1 using 2-furfural (2 g, 20.8 mmol). The crude ester was directly used in the next step.

Ethyl (*E*)-3-(furan-3-yl)-2-methylacrylate

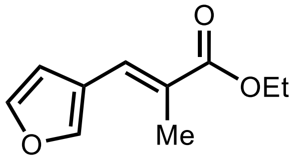

The title compound was prepared according to general procedure 1 using 4-nitrobenzaldehyde (2 g, 20.8 mmol). The crude ester was directly used in the next step.

Ethyl (*E*)-2-methyl-3-(thiophen-2-yl)acrylate

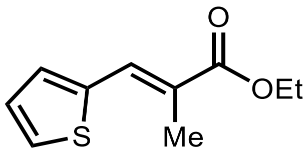

The title compound was prepared according to general procedure 1 using thiophene-2-carbaldehyde (1.2 g, 10.7 mmol). The crude ester was directly used in the next step.

Ethyl (*E*)-2-methyl-3-(thiophen-3-yl)acrylate

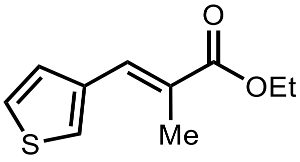

The title compound was prepared according to general procedure 1 using thiophene-3-carbaldehyde (1.2 g, 10.7 mmol). The crude ester was directly used in the next step.

Ethyl (*E*)-2-methyl-3-(pyridin-3-yl)acrylate

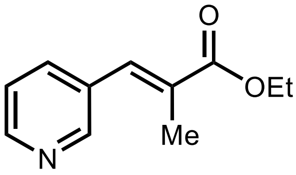

The title compound was prepared according to general procedure 1 using nicotinaldehyde (2 g, 18.7 mmol). The crude ester was directly used in the next step.

Ethyl (*E*)-2-methyl-3-(pyridine-4-yl)acrylate

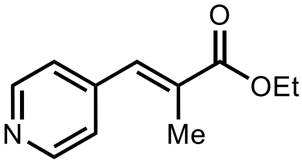

The title compound was prepared according to general procedure 1 using 4-pyridine carbaldehyde (2 g, 18.7 mmol). The crude ester was directly used in the next step.

Ethyl (E)-3-cyclohexyl-2-methylacrylate

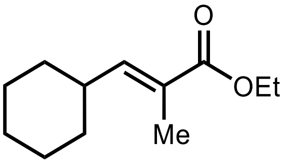

The title compound was prepared according to general procedure 1 using cyclohexanecarbaldehyde (3.7 g, 18.8 mmol). The reaction mixture was allowed to warm to rt and stirred for 1 h. Then the corresponding aldehyde was added to the reaction mixture and the mixture was reflux for 60 h. The crude ester was directly used in the next step.

Ethyl (*E*)-2-methyl-5-phenylpent-2-enoate

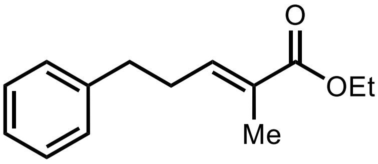

The title compound was prepared according to general procedure 1 using 3-phenylpropanal (2.47 mL, 18.8 mmol). After the addition of 3-phenylpropanal, the reaction mixture was reflux for 4 h. The crude ester was directly used in the next step.

2.1.2. General procedure 2

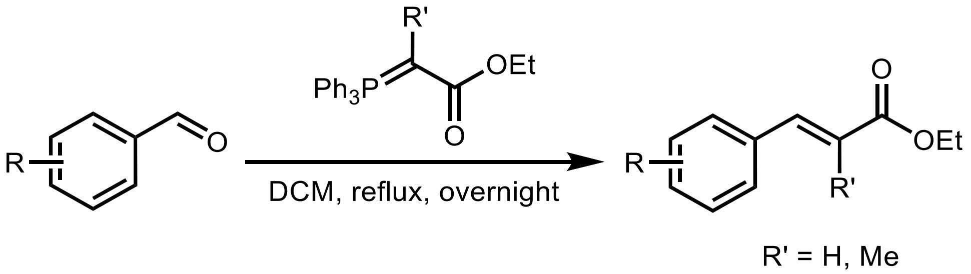

To a solution of the aldehyde (1 equiv.) in DCM (2 mL/mmol) was added the corresponding (carbethoxyethylidene)triphenylphosphorane (1.3 equiv.). The mixture was refluxed overnight and then allowed to cool and the solvent was removed under vacuum. The residue was washed with petroleum ether (5×) and filtered. The combined washings was concentrated in vacuo.

Ethyl (E)-2-methyl-3-phenylacrylate

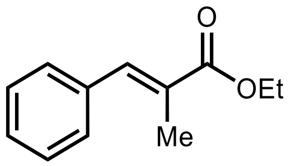

The title compound was prepared according to general procedure 2 using benzaldehyde (2 g, 18.8 mmol). The crude ester was directly used in the next step.

Ethyl (*E*)-2-methyl-3-(*o*-tolyl)acrylate

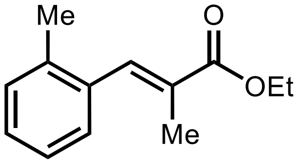

The title compound was prepared according to general procedure 2 using *o*-tolualdehyde (1.03 g, 8.6 mmol). The crude ester was directly used in the next step.

Ethyl (*E*)-2-methyl-3-(*p*-tolyl)acrylate

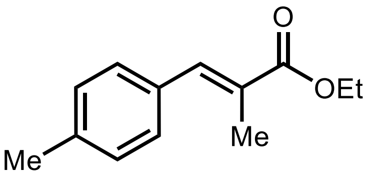

The title compound was prepared according to general procedure 2 using *p*-tolualdehyde (1.01 g, 8.4 mmol). The crude ester was directly used in the next step.

Ethyl(*E*)-3-(4-methoxyphenyl)acrylate

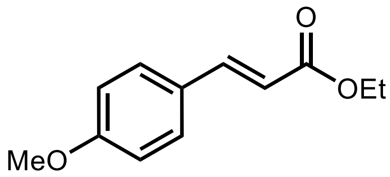

The title compound was prepared according to general procedure 2 using 4-methoxybenzaldehyde (1.01 g, 7.4 mmol). The crude ester was directly used in the next step.

Ethyl (E)-3-(4-methoxyphenyl)-2-methylacrylate

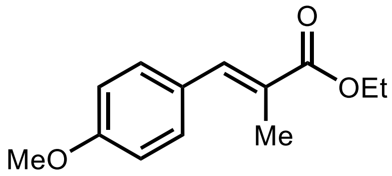

The title compound was prepared according to general procedure 2 using 4-methoxybenzaldehyde (2 g, 14.7 mmol). The crude ester was directly used in the next step.

Ethyl (*E*)-2-methyl-3-(4-vinylphenyl)acrylate

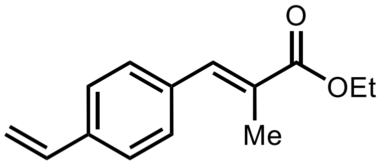

The title compound was prepared according to general procedure 2 using 4-vinylbenzaldehyde (1 g, 7.7 mmol). The crude ester was directly used in the next step.

Ethyl (*E*)-2-methyl-3-(4-(trifluoromethyl)phenyl)acrylate

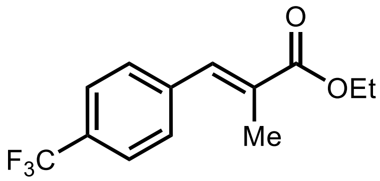

The title compound was prepared according to general procedure 2 using 4-(trifluoromethyl)benzaldehyde (2 g, 11.5 mmol). The crude ester was directly used in the next step.

Ethyl (*E*)-2-methyl-4-phenylbut-2-enoate

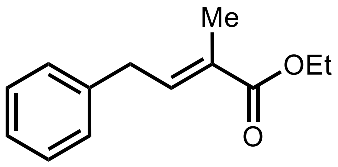

The title compound was prepared according to general procedure 2 using 2-phenylacetaldehyde (1.28 g, 10.6 mmol). The crude ester was directly used in the next step.

2.1.3. General procedure 3

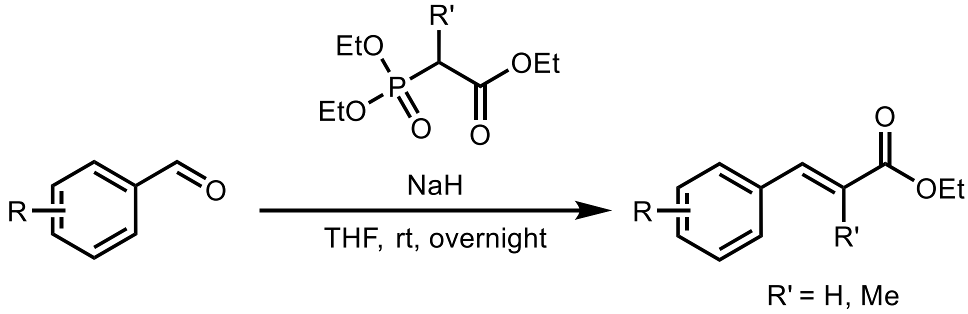

To a stirred solution of NaH (2.5 equiv.) in anhydrous THF (0.2 M solution) at 0 °C was added triethyl phosphonoacetate or triethyl 2-phosphonopropionate (1.5 equiv.). The reaction mixture was allowed to warm to rt and stirred for 1 h. Then the corresponding aldehyde was added to the reaction mixture and the mixture was stirred at rt overnight. The reaction was then quenched by the addition of water and extracted with EtOAc (3×). The combined organics were washed with brine, dried over anhydrous $MgSO_4$, filtered and concentrated in vacuo.

Ethyl cinnamate

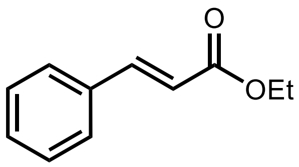

The title compound was prepared according to general procedure 3 using benzaldehyde (5 g, 47 mmol). The crude ester was directly used in the next step.

Ethyl (*E*)-3-(4-fluorophenyl)acrylate

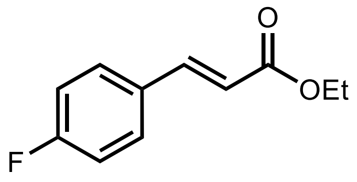

The title compound was prepared according to general procedure 3 using 4-fluorobenzaldehyde (1.5 g, 12.1 mmol). The crude ester was directly used in the next step.

Ethyl (*E*)-3-(4-bromophenyl)-2-methylacrylate

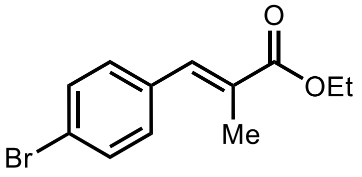

The title compound was prepared according to general procedure 3 using 4-bromobenzaldehyde (4 g, 21.6 mmol). The crude ester was directly used in the next step.

Ethyl (*E*)-2-methyl-3-(*m*-tolyl)acrylate

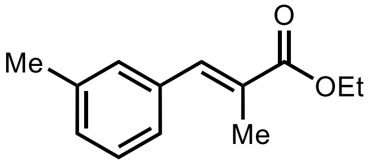

The title compound was prepared according to general procedure 3 using *m*-tolualdehyde (3.4 g, 28 mmol). The crude ester was directly used in the next step.

Ethyl (*E*)-2-methyl-3-(1-methyl-1H-indol-3-yl)acrylate

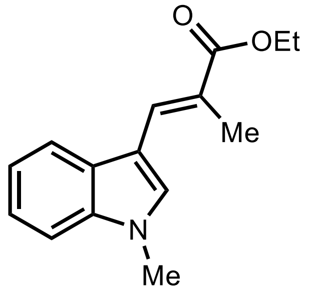

The title compound was prepared according to general procedure 3 using 1-methyl-1H-indole-3-carbaldehyde (2.5 g, 15.7 mmol). The crude ester was directly used in the next step.

Ethyl (*E*)-3-phenylbut-2-enoate

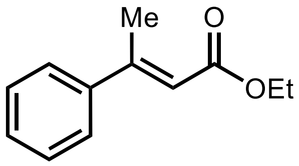

The title compound was prepared according to general procedure 3 using acetophenone (5 g, 41.6 mmol 1 equiv.). The crude ester was directly used in the next step.

Ethyl (*E*)-3-(4-(trifluoromethyl)phenyl)acrylate

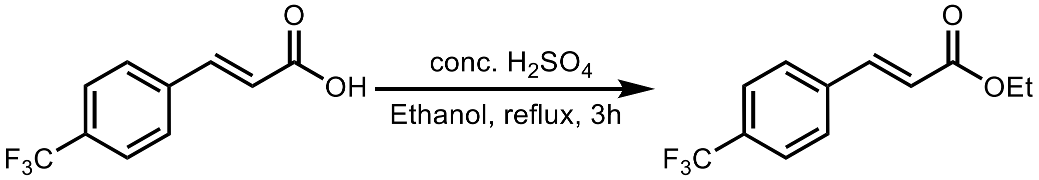

Conc. H_2SO_4 (0.5 mL) was added in one portion to a solution of 4-(trifluoromethyl)cinnamic acid (5 g, 23 mmol) in ethanol (50 mL). The reaction mixture was refluxed for 3 h. After completion, the reaction mixture was allowed to cool, neutralized by slow addition of sat. aq. $NaHCO_3$ to form a white suspension and then extracted with EtOAc (2×). The combined organics was washed with brine (1×), dried over anhydrous $MgSO_4$, filtered and concentrated in vacuo. The crude ester was used in the next step without further purification.

Methyl (*E*)-4-(2-methyl-3-oxoprop-1-en-1-yl)benzoate

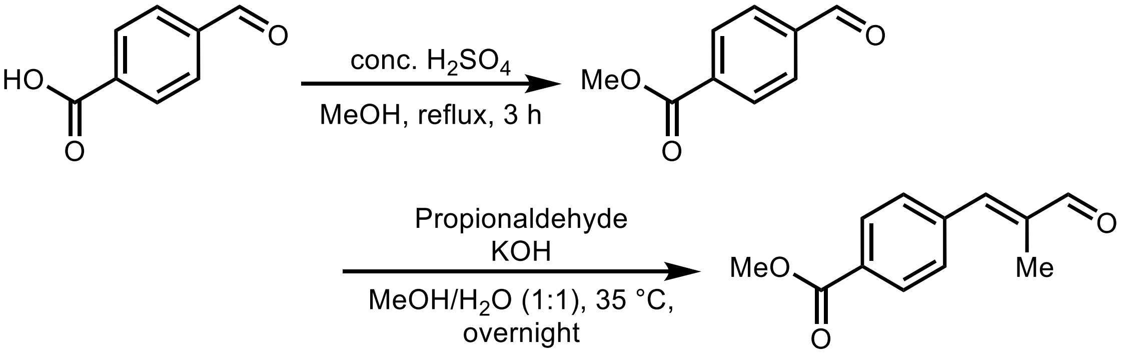

Conc. H_2SO_4 (0.2 mL) was added in one portion to a solution of 4-formylbenzoic acid (2 g, 13.3 mmol) in methanol (20 mL). The reaction mixture was refluxed for 3 h. After completion, the reaction mixture was allowed to cool, neutralized by slow addition of sat. aq. $NaHCO_3$ to form a white suspension and then extracted with EtOAc (2×). The combined organics was washed with brine (1×), dried over anhydrous $MgSO_4$, filtered and concentrated in vacuo. The crude aldehyde was used in the next step without further purification.

To a solution of KOH (256 mg, 4.57 mmol, 0.5 equiv.) in MeOH/H_2O (1:1) (10 mL) at 0 °C, propionaldehyde (1.31 mL, 18.3 mmol, 2 equiv.) was added and the mixture was stirred for 15 min at 0 °C. Methyl 4-formylbenzoate (1.5 g, 9.1 mmol, 1 equiv.) was then added slowly at rt. After stirring at 35 °C overnight, the mixture was acidified with 1 M HCl, extracted with Et_2O (2×). The combined organic layers were washed with brine, dried over anhydrous $MgSO_4$, filtered and concentrated in vacuo to yield the corresponding unsaturated aldehyde that was used without further purification.

2.1.4. General procedure 4

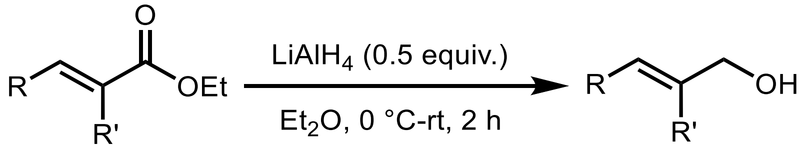

To a stirred solution of the acrylic ester (1 equiv.) in dry Et_2O (2 mL/mmol) at 0 °C under N_2 was added $LiAlH_4$ (0.5 equiv.) at once. The reaction mixture was stirred for 2 h at rt and then quenched with H_2O. The reaction mixture was then acidified with 1 M HCl, extracted with EtOAc (3×) and the combined organic layers were washed with H_2O (2×), dried over anhydrous $MgSO_4$, filtered and concentrated in vacuo.

(*E*)-2-methyl-3-(p-tolyl)prop-2-en-1-ol

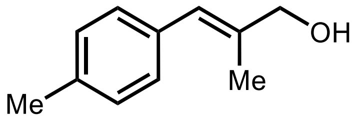

The title compound was prepared according to general procedure 4 using ethyl (*E*)-3-(4-methoxyphenyl)-2-methylacrylate (1.6 g, 7.9 mmol). Purification by flash silica chromatography (eluent = 10-30% EtOAc in petroleum ether, 35 × 160 mm silica) gave the title compound as a clear oil (730 mg, 56%); R_f = 0.15 (eluent = 10% EtOAc in petroleum ether); ^{1}H NMR (500 MHz, Chloroform-*d*) δ 7.17 (dd, *J* = 19.7, 8.1 Hz, 4H), 6.49 (s, 1H), 4.19 (s, 2H), 2.35 (s, 3H), 1.91 (s, 3H), 1.53 (s, 1H); ^{13}C{^{1}H} NMR (126 MHz, Chloroform-*d*) δ 137.0, 136.3, 134.7, 129.0, 128.9, 125.2, 69.4, 21.3, 15.5. Spectroscopic data in accordance with that stated in the literature.[5]

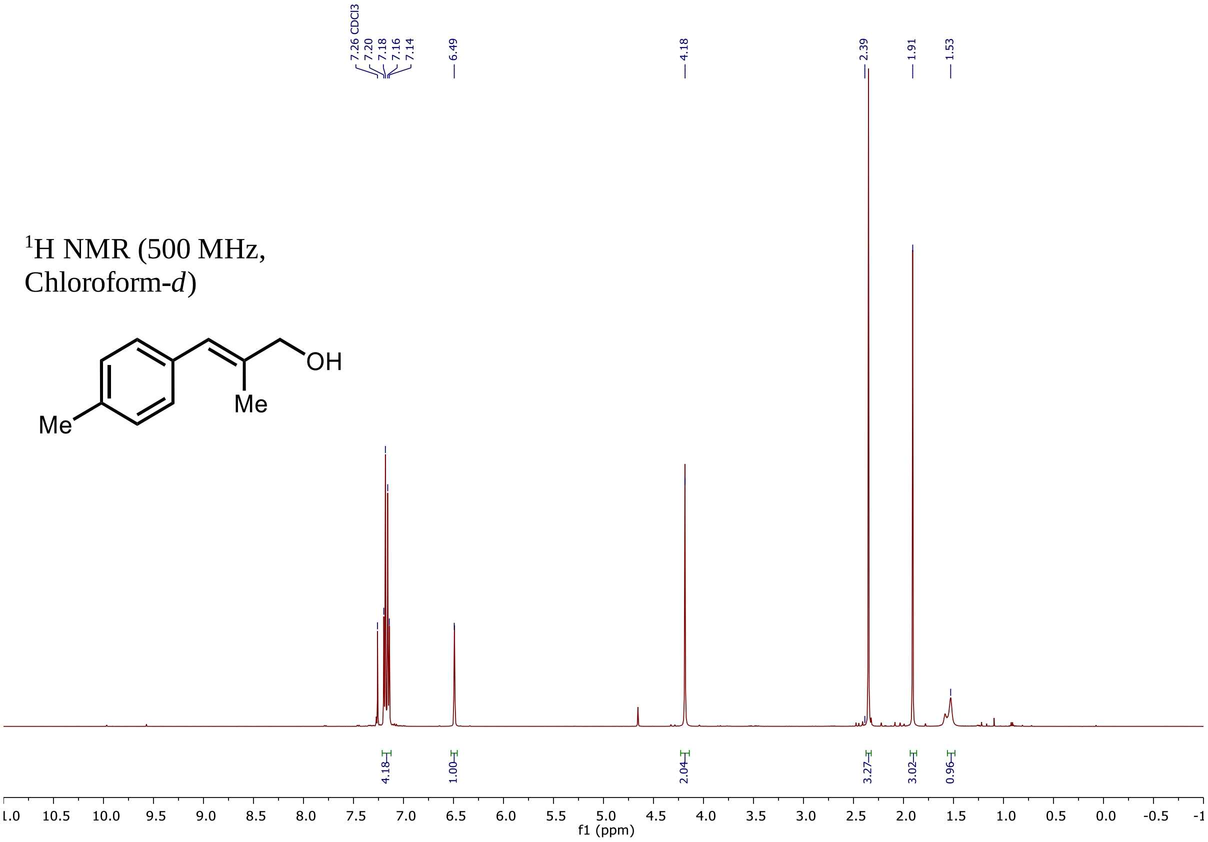
1H NMR (500 MHz, Chloroform-d)
OH
Me
Me
7.26 CDCl3
7.20
7.18
7.16
7.14
6.49
4.18
2.39
1.91
1.53
4.18
1.00
2.04
3.27
3.02
0.96
f1 (ppm)

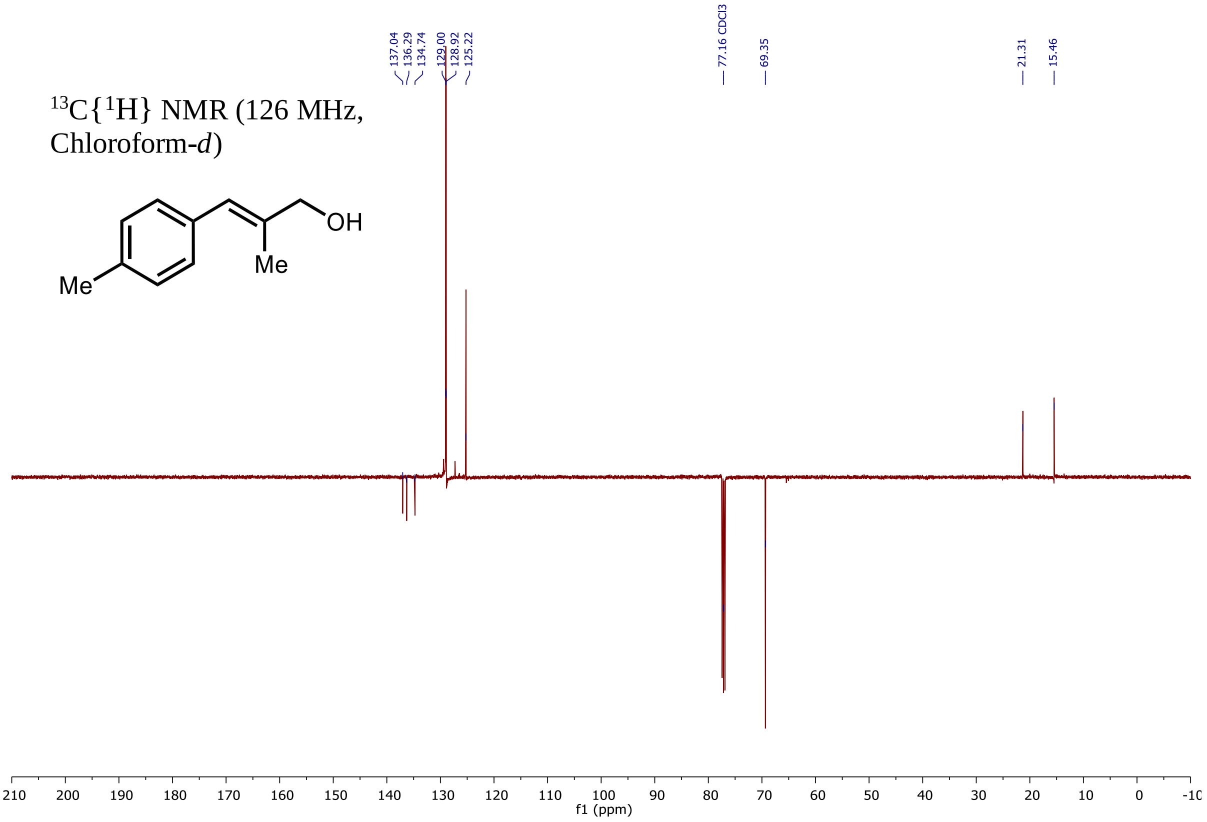
13C{1H} NMR (126 MHz, Chloroform-d)
OH
Me
Me
137.04
136.29
134.74
129.00
128.92
125.22
77.16 CDCl3
69.35
21.31
15.46
f1 (ppm)

(*E*)-2-methyl-3-(m-tolyl)prop-2-en-1-ol

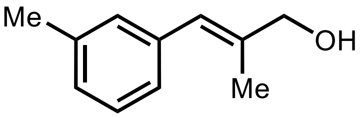

The title compound was prepared according to general procedure 4 using ethyl (*E*)-2-methyl-3-(m-tolyl)acrylate (1.5 g, 7.3 mmol). Purification by flash silica chromatography (eluent = 20% EtOAc in petroleum ether, 35 × 160 mm silica) gave the title compound as a colourless oil (412 mg, 35%); R_f = 0.31 (eluent = 20% EtOAc in petroleum ether); ν_{max} / cm^{-1} (film) 3323, 2916, 2858, 1603, 1485, 1445, 1377, 1067, 1007, 783, 698; ^{1}H NMR (500 MHz, Chloroform-*d*) δ 7.40 (t, *J* = 7.8 Hz, 1H), 7.29 – 7.24 (m, 2H), 7.21 (d, *J* = 7.6 Hz, 1H), 6.66 (s, 1H), 4.35 (d, *J* = 1.7 Hz, 2H), 2.52 (s, 3H), 2.07 (d, *J* = 1.3 Hz, 4H); ^{13}C{^{1}H} NMR (126 MHz, Chloroform-*d*) δ 137.8, 137.6, 137.6, 129.8, 128.2, 127.4, 126.1, 125.3, 69.2 (d, *J* = 1.4 Hz), 21.6, 15.5; HRMS (EI-quadrupole) m/z: $(M)^+$ Calcd for $C_{11}H_{14}O$ 162.1039; Found 162.1037.

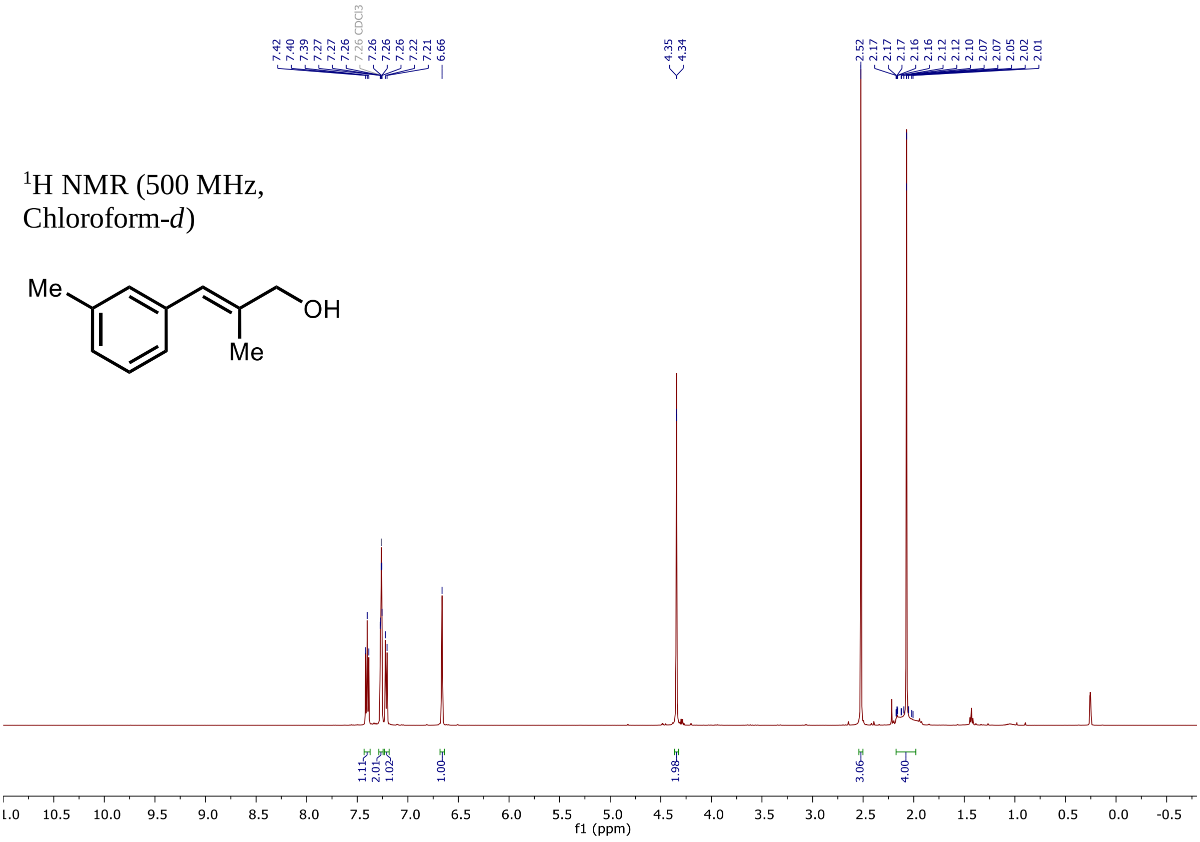

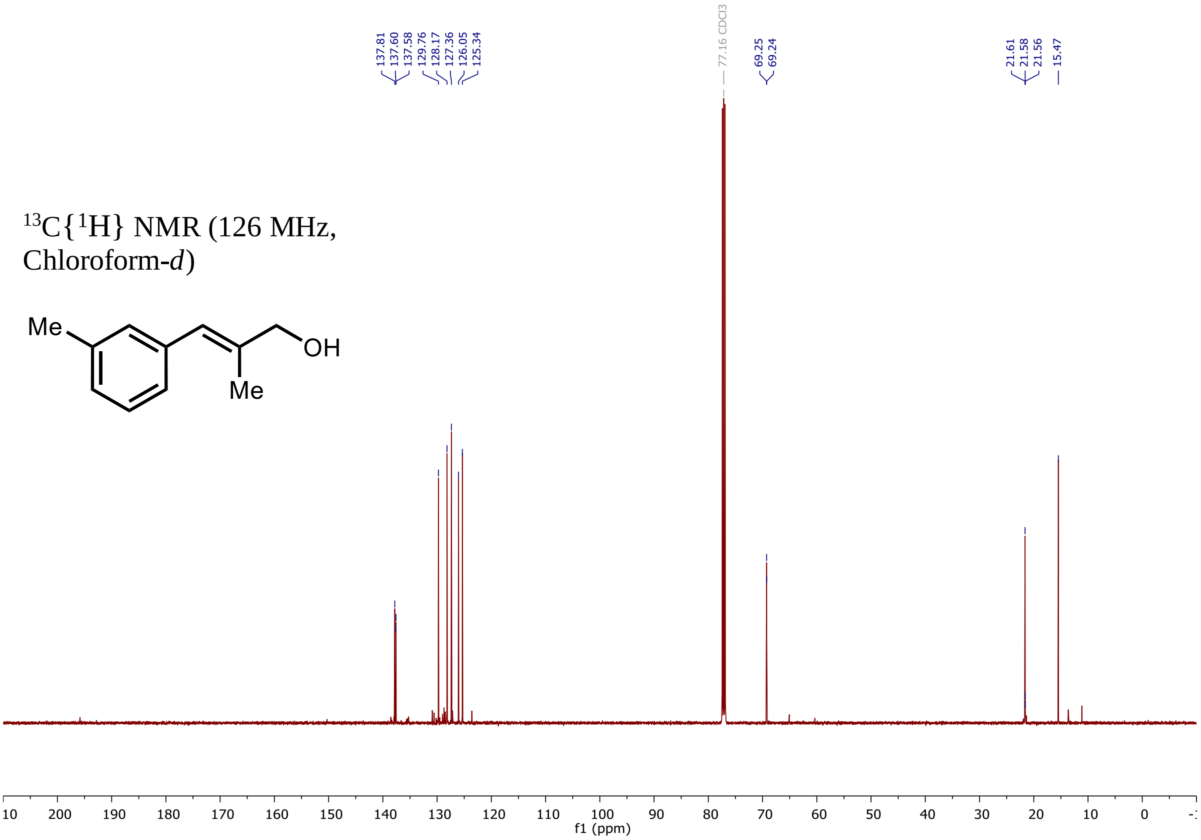

(*E*)-2-methyl-3-(o-tolyl)prop-2-en-1-ol

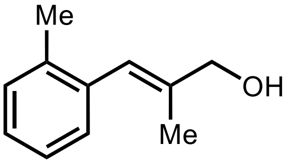

The title compound was prepared according to general procedure 4 using ethyl (*E*)-2-methyl-3-(o-tolyl)acrylate (1.27 g, 6.2 mmol). Purification by flash silica chromatography (eluent = 10-30% EtOAc in petroleum ether, 35 × 160 mm silica) gave the title compound as a clear oil (725 mg, 72%); R_f = 0.15 (eluent = 10% EtOAc in petroleum ether); ^{1}H NMR (500 MHz, Chloroform-*d*) δ 7.20 – 7.14 (m, 4H), 6.52 (s, 1H), 4.23 (s, 2H), 2.25 (s, 3H), 1.76 (s, 3H), 1.55 (s, 1H); ^{13}C{^{1}H} NMR (126 MHz, Chloroform-*d*) δ 137.9, 136.8, 136.6, 129.9, 129.3, 126.9, 125.5, 124.2, 68.8, 20.1, 15.2. Spectroscopic data in accordance with that stated in the literature.[5]

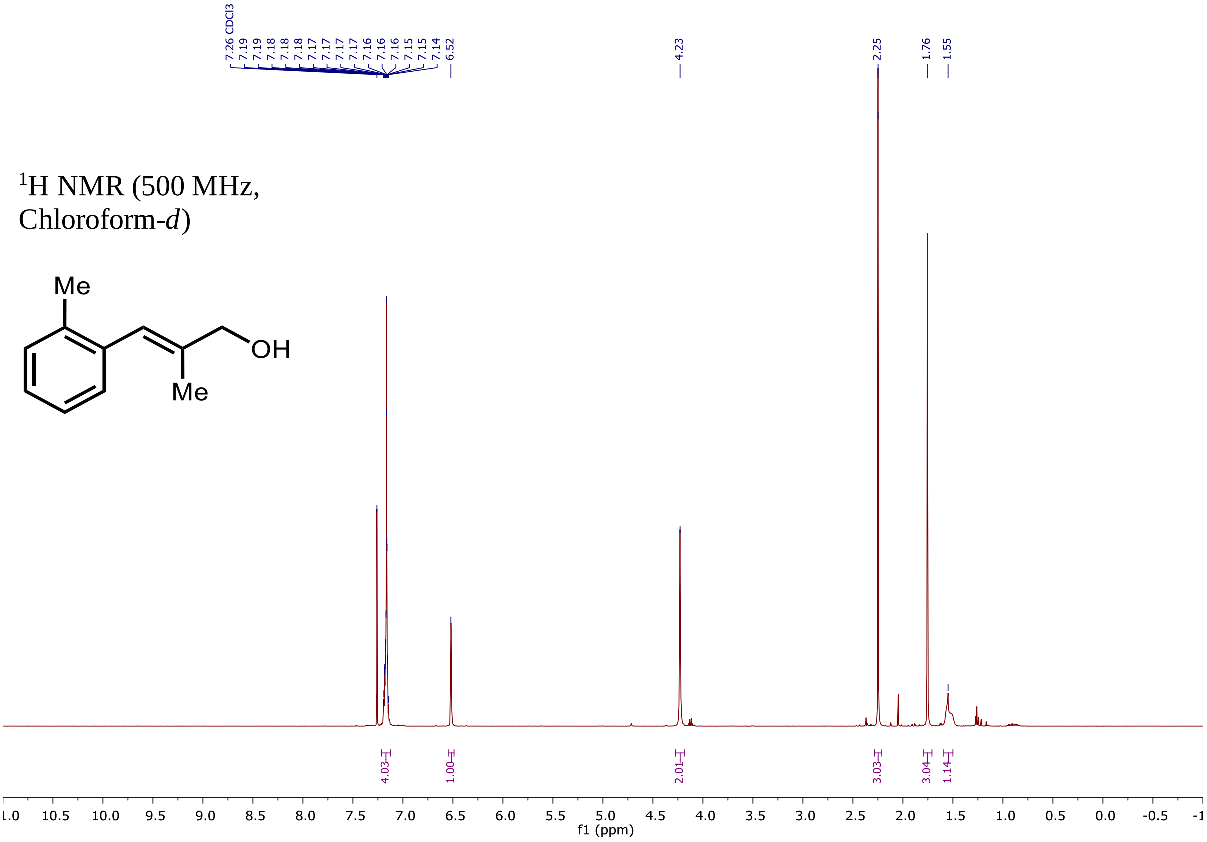

^{1}H NMR (500 MHz, Chloroform-*d*)
Me
OH
Me
f1 (ppm)

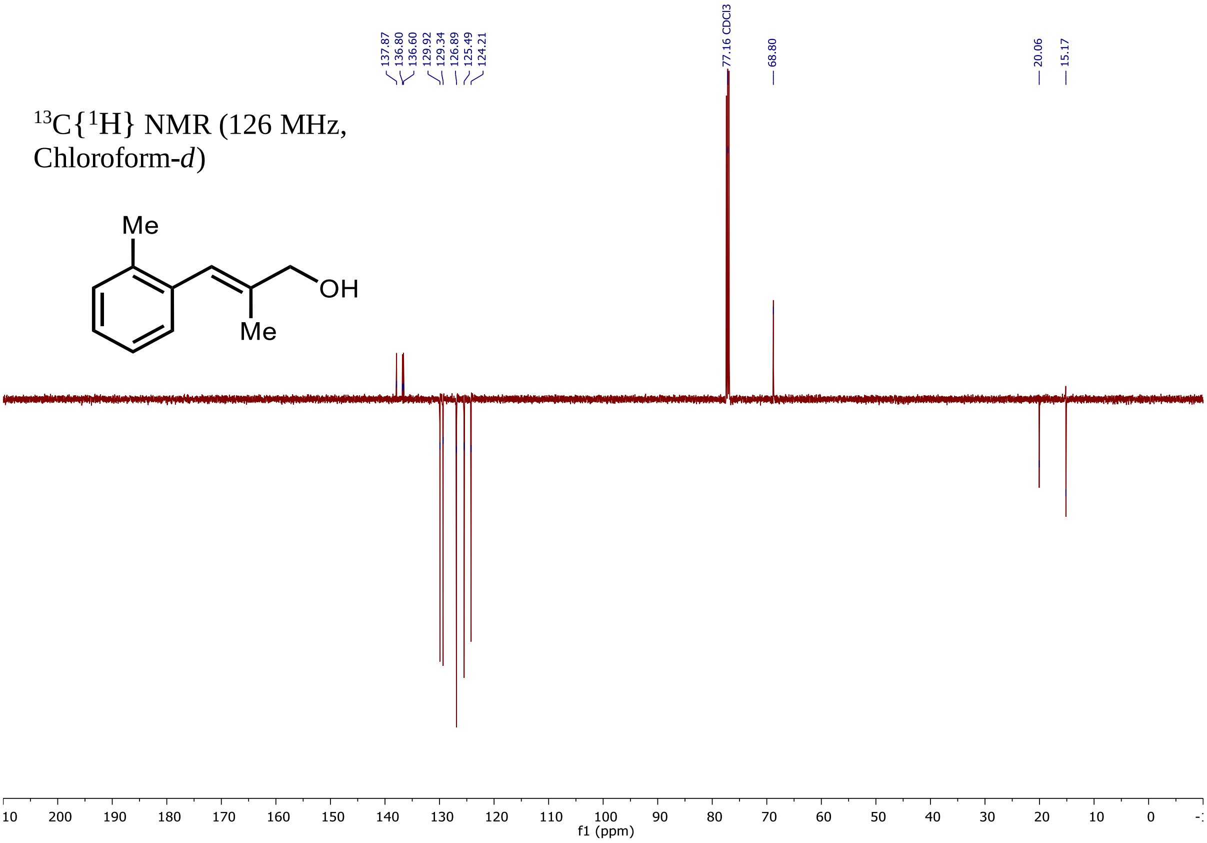

^{13}C{^{1}H} NMR (126 MHz, Chloroform-*d*)
Me
OH
Me
f1 (ppm)

(*E*)-3-(4-fluorophenyl)-2-methylprop-2-en-1-ol

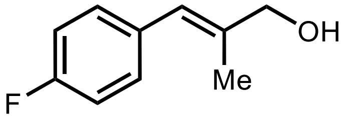

The title compound was prepared according to general procedure 4 using ethyl (*E*)-3-(4-fluorophenyl)-2-methylacrylate (1.5 g, 7.2 mmol). Purification by flash silica chromatography (eluent = 20% EtOAc in petroleum ether, 35 × 160 mm silica) gave the title compound as a colourless oil (1.1 g, 90%); R_f = 0.45 (eluent = 20% EtOAc in petroleum ether); ^{1}H NMR (500 MHz, Chloroform-*d*) δ 7.23 (dddd, *J* = 8.4, 5.3, 2.6, 1.5 Hz, 2H), 7.05 – 6.98 (m, 2H), 6.49 – 6.47 (m, 1H), 4.18 (d, *J* = 1.4 Hz, 2H), 1.87 (d, *J* = 1.4 Hz, 3H), 1.66 (s, 1H); ^{13}C{^{1}H} NMR (126 MHz, Chloroform-*d*) δ 161.5 (d, $J_{C\text{-}F}$ = 245.8 Hz), 137.7 (d, $J_{C\text{-}F}$ = 1.4 Hz), 133.7 (d, $J_{C\text{-}F}$ = 3.4 Hz), 130.5 (d, $J_{C\text{-}F}$ = 7.9 Hz), 124.1, 115.2 (d, $J_{C\text{-}F}$ = 21.3 Hz), 69.0, 15.3; ^{19}F{^{1}H} NMR (471 MHz, Chloroform-*d*) δ -115.9. Spectroscopic data in accordance with that stated in the literature.[6]

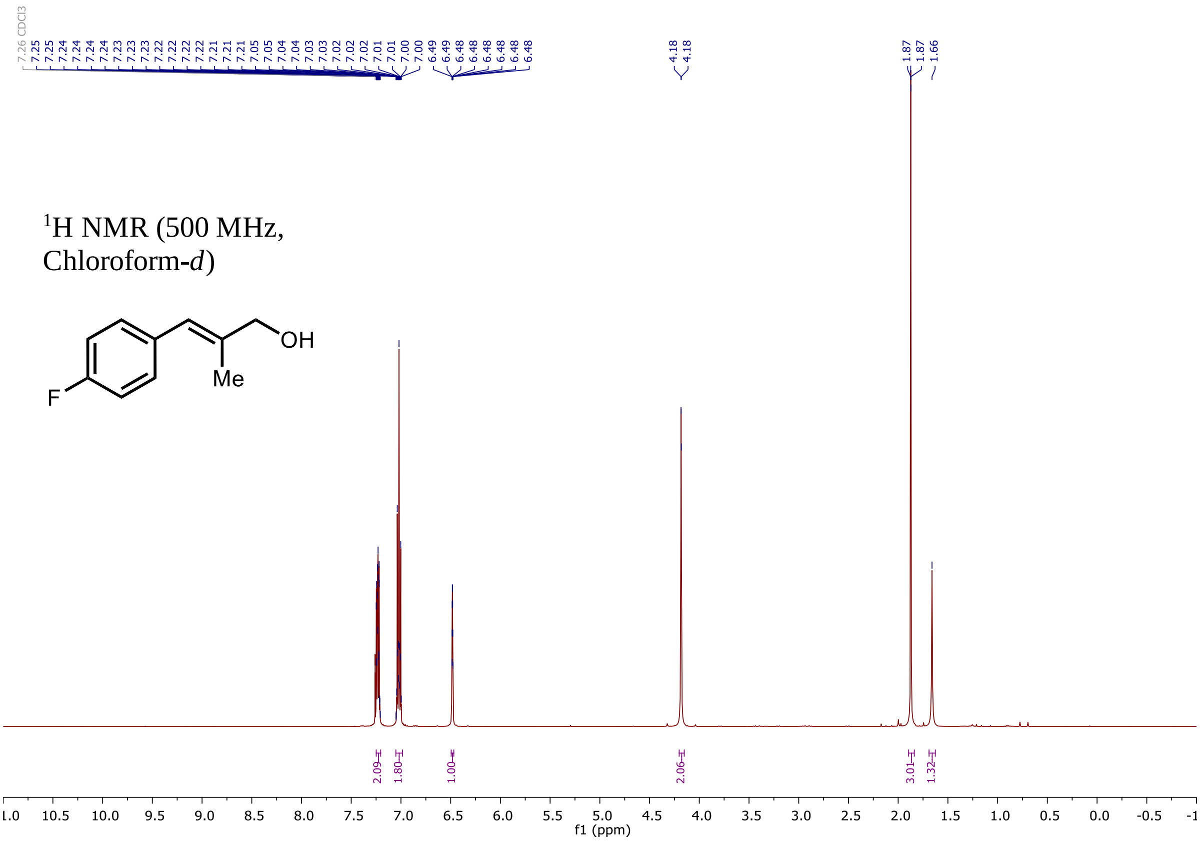

$^{13}C\{^{1}H\}$ NMR (126 MHz, Chloroform-*d*)

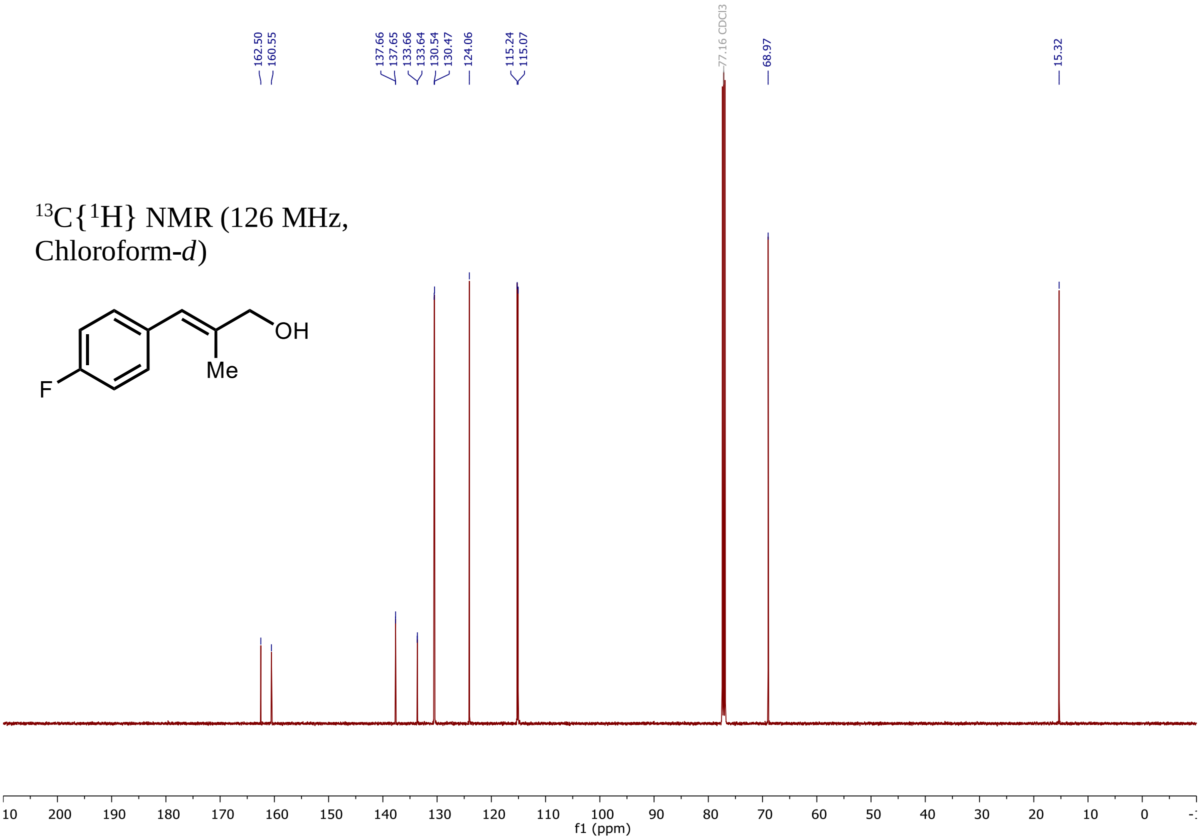

$^{19}F\{^{1}H\}$ NMR (471 MHz, Chloroform-*d*)

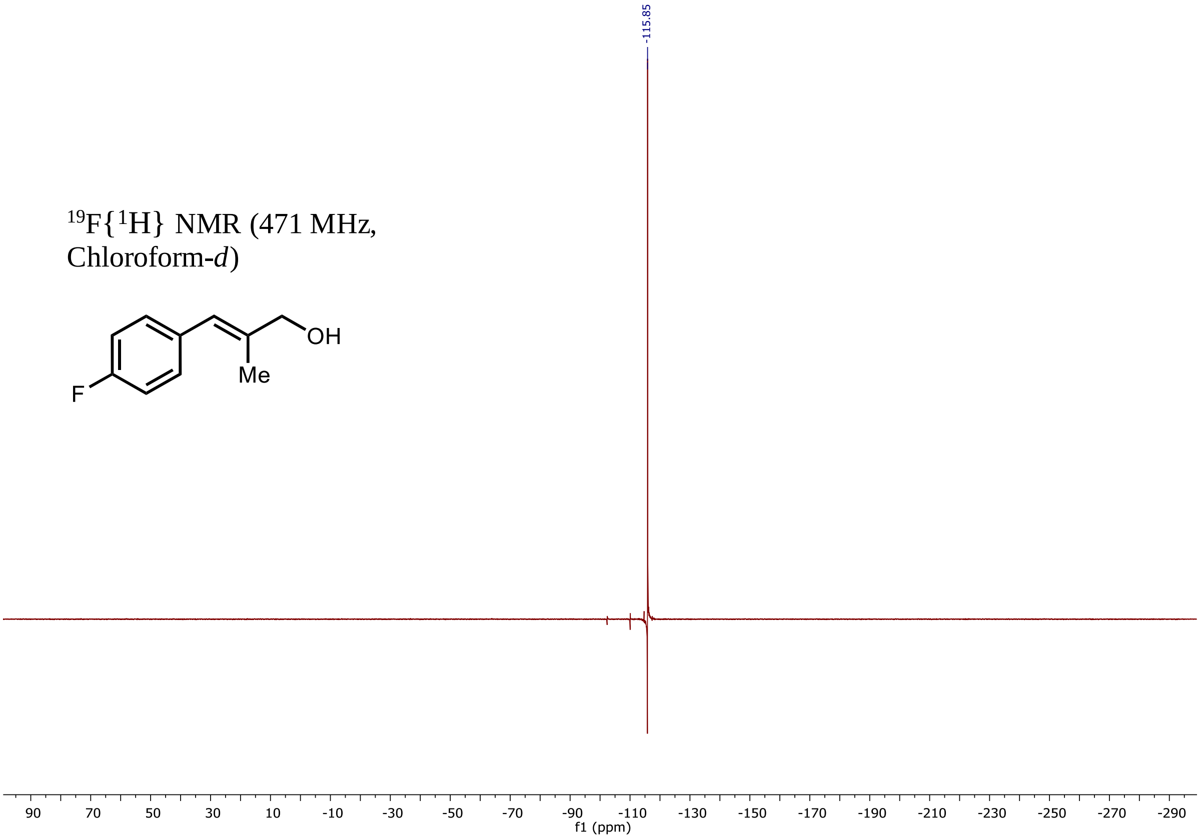

(*E*)-3-(4-chlorophenyl)-2-methylprop-2-en-1-ol

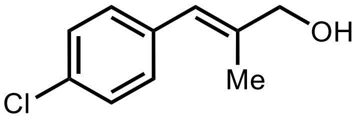

The title compound was prepared according to general procedure 4 using ethyl (*E*)-3-(4-chlorophenyl)-2-methylacrylate (1.5 g, 6.7 mmol). Purification by flash silica chromatography (eluent = 15-20% EtOAc in petroleum ether, 35 × 160 mm silica) gave the title compound as a white solid (638 mg, 52%); mp 63-65 °C (Lit 66-66.4 °C);[7] R_f = 0.62 (eluent = 20% EtOAc in petroleum ether); ^{1}H NMR (500 MHz, Chloroform-*d*) δ 7.33 – 7.26 (m, 2H), 7.24 – 7.16 (m, 2H), 6.48 (s, 1H), 4.19 (s, 2H), 1.88 (d, *J* = 1.5 Hz, 3H), 1.52 (s, 1H); ^{13}C{^{1}H} NMR (126 MHz, Chloroform-*d*) δ 138.5, 136.1, 132.3, 130.3, 128.5, 123.9, 68.9, 15.4. Spectroscopic data in accordance with that stated in the literature.[5]

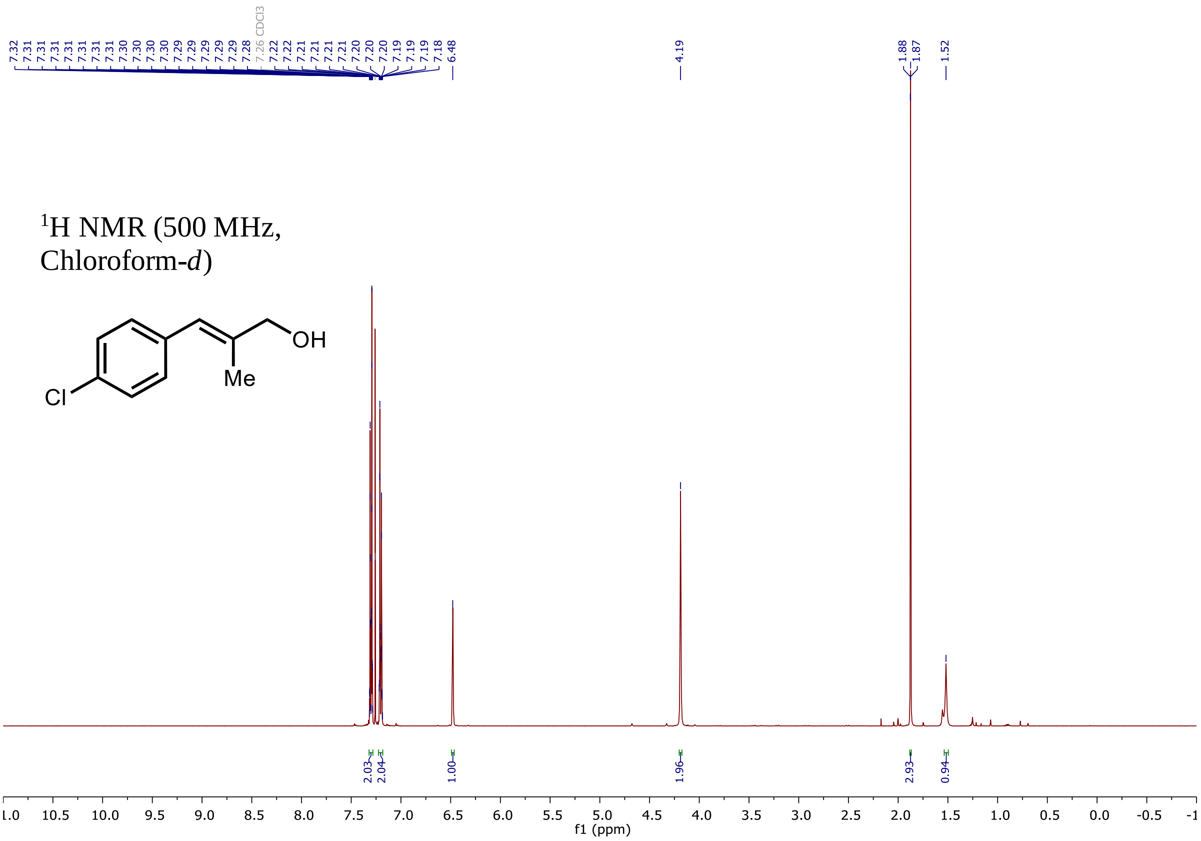

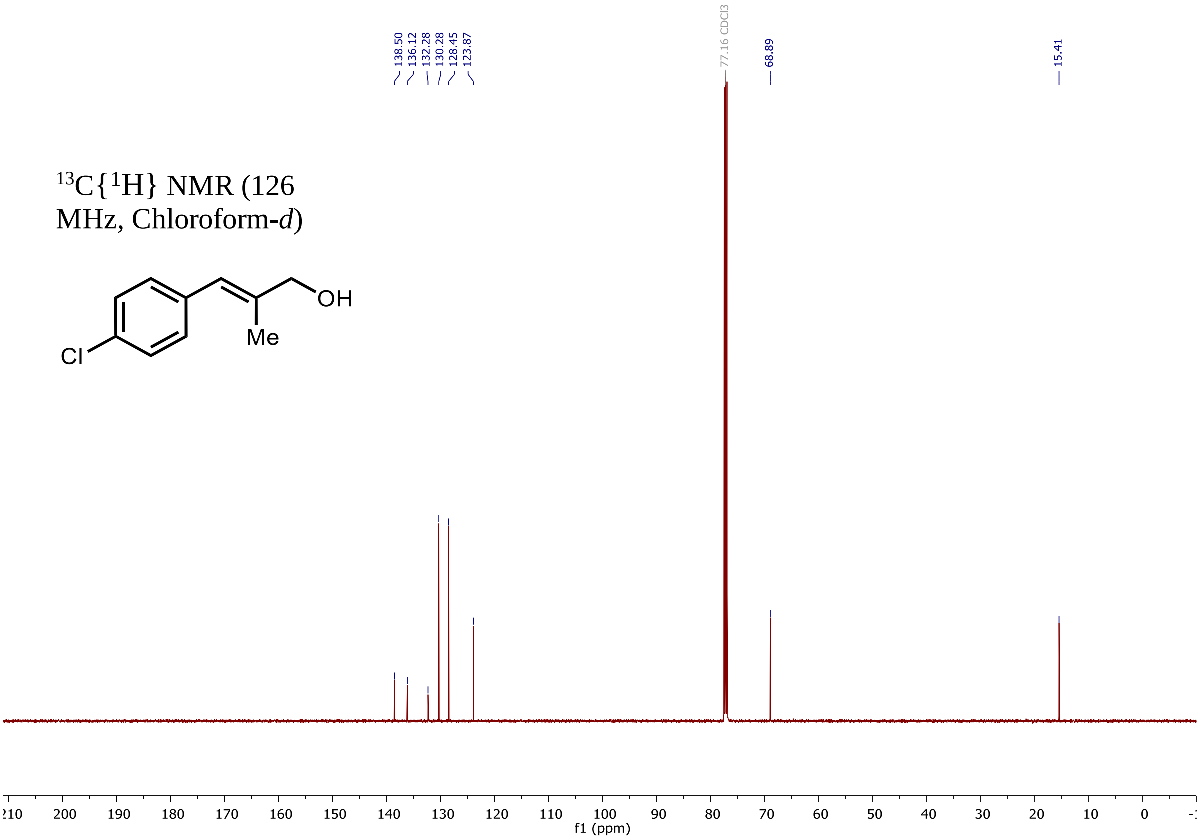

(*E*)-3-(4-bromophenyl)-2-methylprop-2-en-1-ol

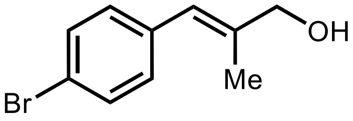

The title compound was prepared according to general procedure 4 using ethyl (*E*)-3-(4-bromophenyl)-2-methylacrylate (2 g, 7.7 mmol). Purification by flash silica chromatography (eluent = 30% EtOAc in petroleum ether, 35 × 160 mm silica) gave the title compound as white crystals (1.2 g, 69%); mp 81-83 °C (Lit 80.7-81.3 °C);[7] R_f = 0.44 (eluent = 30% EtOAc in petroleum ether); ^{1}H NMR (500 MHz, Chloroform-*d*) δ 7.49 – 7.42 (m, 2H), 7.18 – 7.10 (m, 2H), 6.46 (s, 1H), 4.18 (s, 2H), 1.87 (d, *J* = 1.5 Hz, 3H), 1.55 (s, 1H); ^{13}C{^{1}H} NMR (126 MHz, Chloroform-*d*) δ 138.6, 136.6, 131.4, 130.6, 123.9, 120.4, 68.9, 15.4. Spectroscopic data in accordance with that stated in the literature.[6]

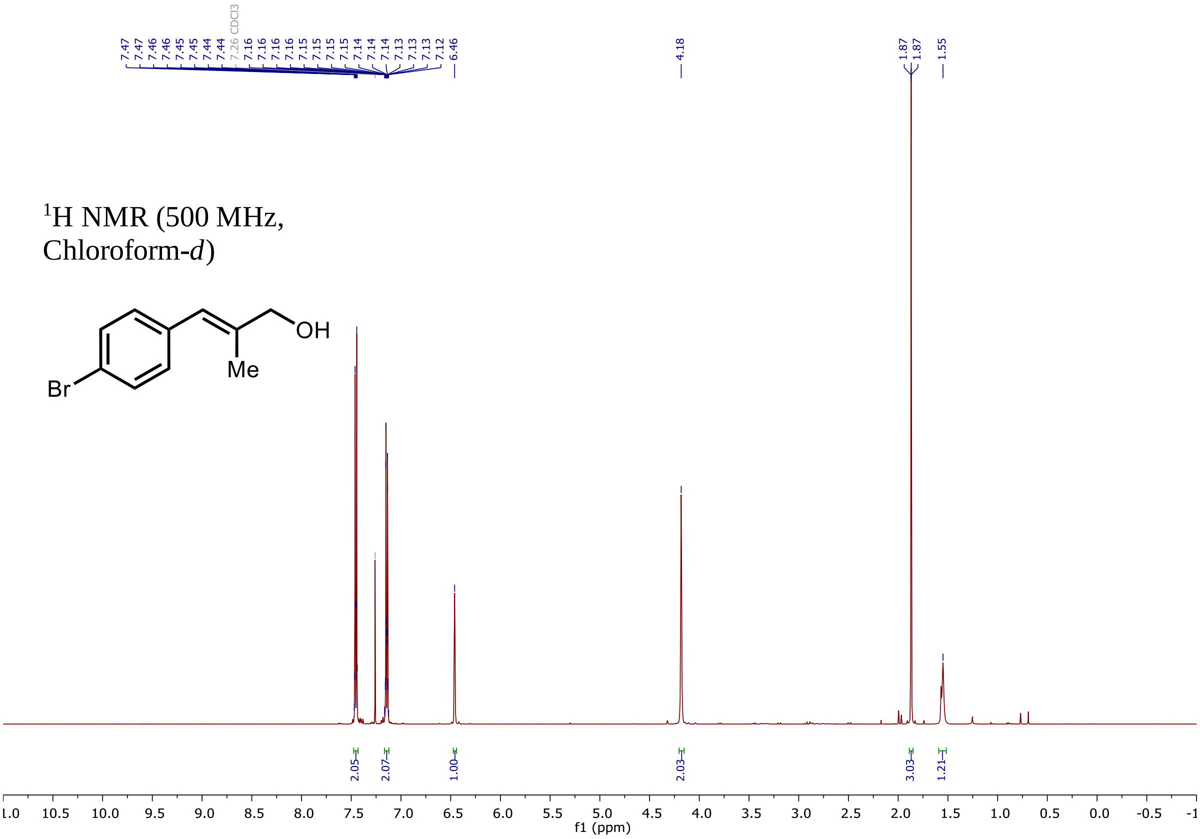
^{1}H NMR (500 MHz, Chloroform-*d*)
Br
Me
OH
7.47
7.47
7.46
7.46
7.45
7.45
7.44
7.44
7.26 CDCl3
7.16
7.16
7.16
7.16
7.15
7.15
7.15
7.15
7.14
7.14
7.14
7.13
7.13
7.13
7.12
6.46
4.18
1.87
1.87
1.55
2.05
2.07
1.00
2.03
3.03
1.21
f1 (ppm)

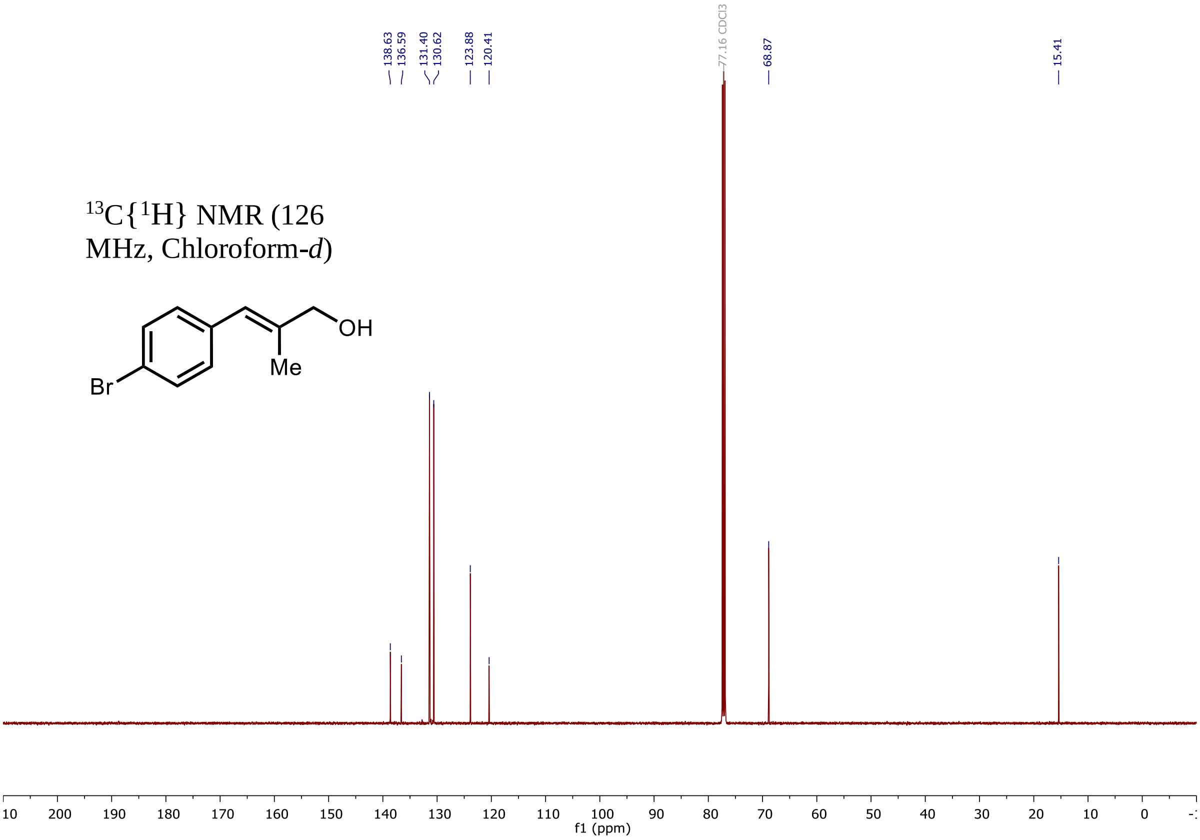
^{13}C{^{1}H} NMR (126 MHz, Chloroform-*d*)
Br
Me
OH
138.63
136.59
131.40
130.62
123.88
120.41
77.16 CDCl3
68.87
15.41
f1 (ppm)

(*E*)-3-(4-methoxyphenyl)-2-methylprop-2-en-1-ol

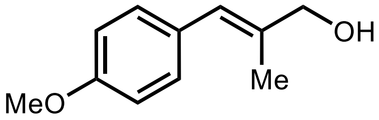

The title compound was prepared according to general procedure 4 using ethyl (*E*)-3-(4-methoxyphenyl)-2-methylacrylate (2.9 g, 13.2 mmol). Purification by flash silica chromatography (eluent = 10-30% EtOAc in petroleum ether, 35 × 160 mm silica) gave the title compound as a white solid (1.7 g, 68%); mp 45-47 °C (Lit. 39-41 °C);[8] R_f = 0.15 (eluent = 10% EtOAc in petroleum ether); ν_{max} / cm^{-1} (film) 3331, 2906, 2843, 1604, 1570, 1442, 1338, 1300, 1294, 1176, 1011, 875, 815, 551; ^{1}H NMR (500 MHz, Chloroform-*d*) δ 7.24 (d, *J* = 8.7 Hz, 2H), 6.89 (d, *J* = 8.8 Hz, 2H), 6.46 (s, 1H), 4.18 (s, 2H), 3.82 (s, 3H), 1.91 (s, 3H), 1.56 (s, 1H); ^{13}C{^{1}H} NMR (126 MHz, Chloroform-*d*) δ 158.3, 136.2, 130.3, 130.2, 125.0, 113.7, 69.5, 55.4, 15.5; HRMS (EI-quadrupole) m/z: ((M + H) - H_2O)$^+$ Calcd for $C_{11}H_{13}O$ 161.0966; Found 161.0966.

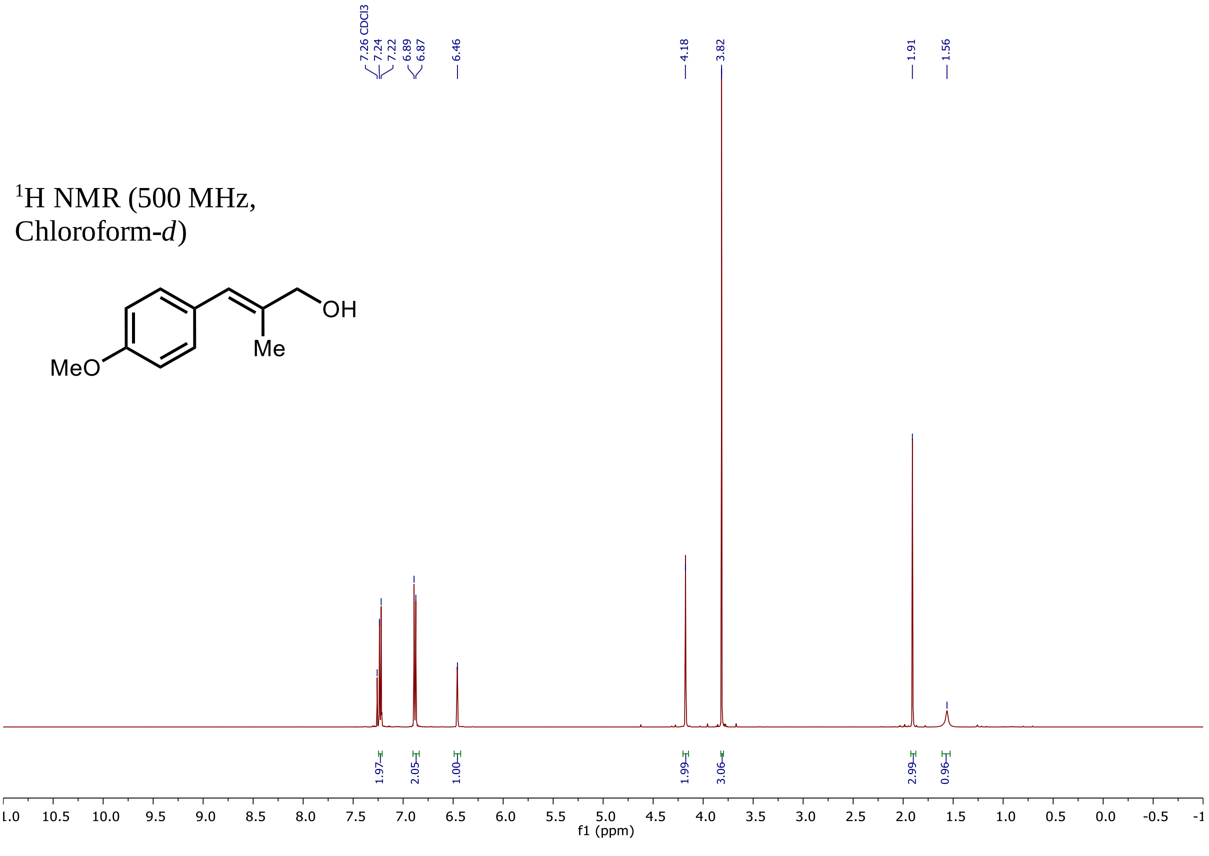

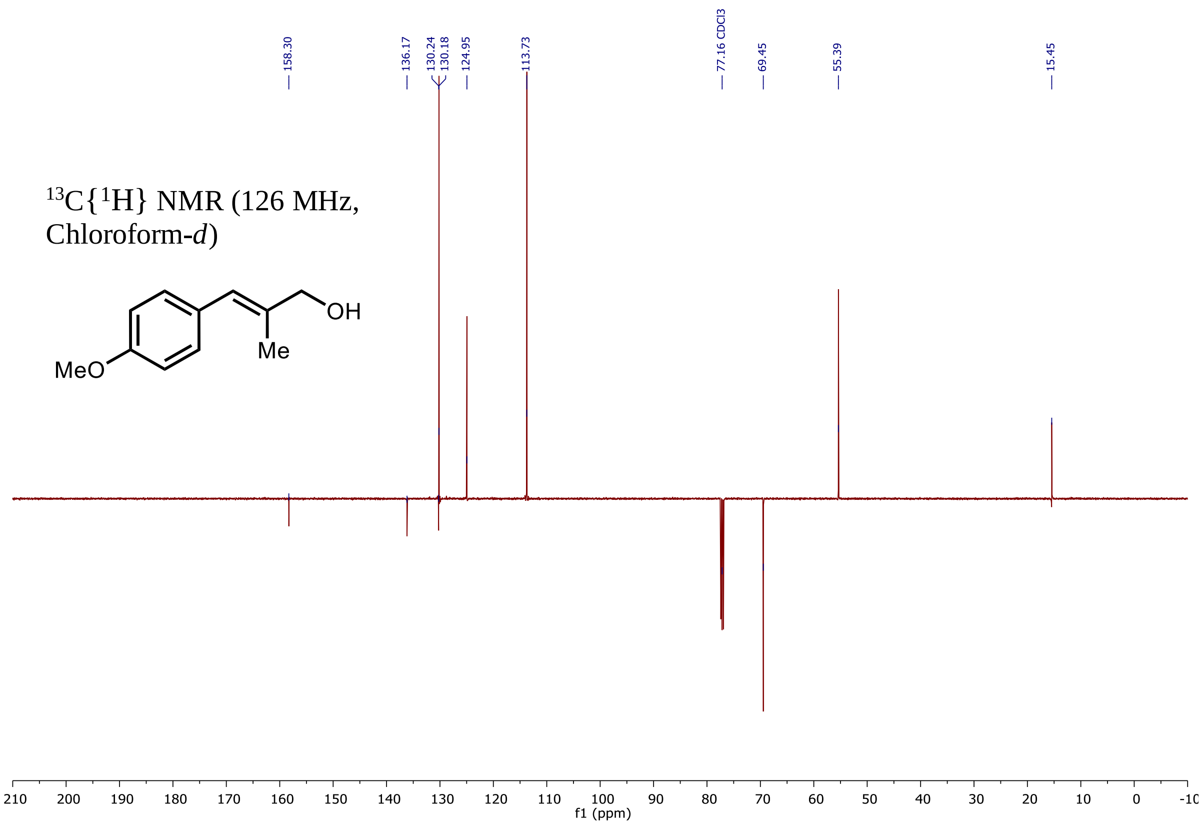

(*E*)-3-(4-(dimethylamino)phenyl)-2-methylprop-2-en-1-ol

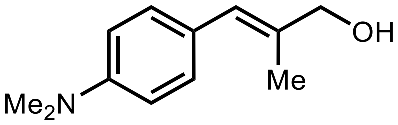

The title compound was prepared according to general procedure 4 using ethyl (*E*)-3-(4-(dimethylamino)phenyl)-2-methylacrylate (1.36 g, 5.8 mmol). Purification by flash silica chromatography (eluent = 20% EtOAc in petroleum ether, 35 × 160 mm silica) gave the title compound as a yellow oil (830 mg, 74%); R_f = 0.31 (eluent = 20% EtOAc in petroleum ether); ν_{max} / cm^{-1} (film) 3363, 2927, 2852, 2800, 1606, 1477, 1442, 1346, 1222, 1190, 1163, 1128, 1160, 1004, 945, 854, 817, 798, 752, 532, 439, 428, 416, 403; ^1H NMR (300 MHz, Chloroform-*d*) δ 7.24 – 7.18 (m, 2H), 6.76 – 6.68 (m, 2H), 6.41 (d, *J* = 1.8 Hz, 1H), 4.17 (s, 2H), 2.96 (s, 6H), 1.94 (d, *J* = 1.4 Hz, 3H), 1.48 (s, 1H); $^{13}C\{^1H\}$ NMR (126 MHz, Chloroform-*d*) δ 149.3, 134.4, 130.0, 126.0, 125.8, 112.3, 111.8, 70.0, 40.7, 15.6; HRMS (EI-quadrupole) $(M)^+$ Calcd for $C_{12}H_{17}NO$ 191.1305; Found 191.1301.

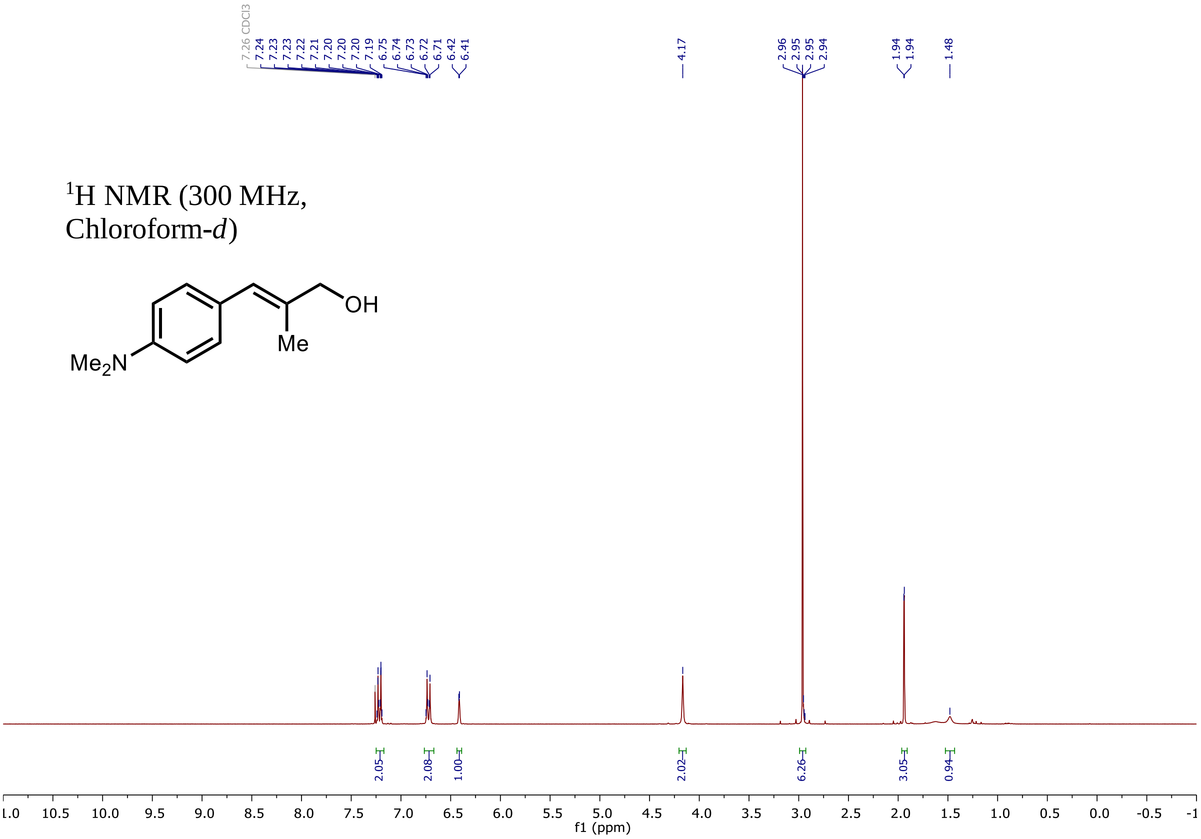

1H NMR (300 MHz, Chloroform-d)
OH
Me
Me2N
7.26 CDCl3
7.24
7.23
7.23
7.22
7.21
7.20
7.20
7.20
7.19
6.75
6.74
6.73
6.72
6.71
6.42
6.41
4.17
2.96
2.95
2.95
2.94
1.94
1.94
1.48
2.05
2.08
1.00
2.02
6.26
3.05
0.94
f1 (ppm)

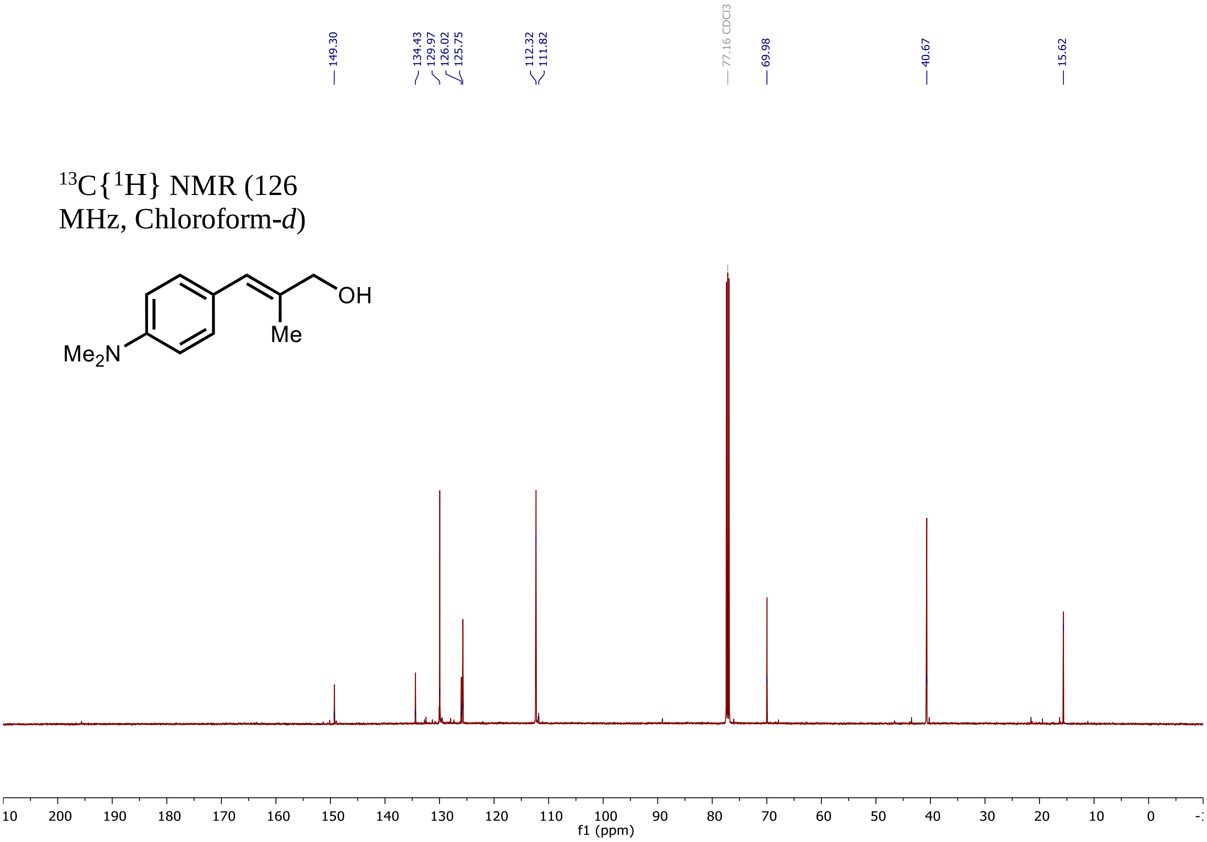

13C{1H} NMR (126 MHz, Chloroform-d)
OH
Me
Me2N
149.30
134.43
129.97
126.02
125.75
112.32
111.82
77.16 CDCl3
69.98
40.67
15.62
f1 (ppm)

(*E*)-2-methyl-3-(4-(trifluoromethyl)phenyl)prop-2-en-1-ol

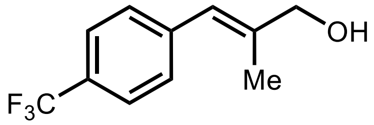

The title compound was prepared according to a general procedure 4 using ethyl (*E*)-2-methyl-3-(4-(trifluoromethyl)phenyl)acrylate (2.55 g, 9.9 mmol). Purification by flash silica chromatography (eluent = 10-30% EtOAc in petroleum ether, 35 × 160 mm silica) gave the title compound as a clear liquid (730 mg, 76%); R_f = 0.15 (eluent = 20% EtOAc in petroleum ether); ν_{max} / cm^{-1} (film) 3336, 2931, 2862, 1616, 1321, 1161, 1106, 1064, 1012, 862, 594, 514; ^{1}H NMR (300 MHz, Chloroform-*d*) δ 7.60 (d, J = 8.1 Hz, 2H), 7.39 (d, J = 8.4 Hz, 2H), 6.59 (s, 1H), 4.24 (s, 2H), 1.91 (s, 3H), 1.65 (s, 1H); ^{13}C{^{1}H} NMR (126 MHz, Chloroform-*d*) δ 168.2 (q, $J_{C\text{-}F}$ = 3.8 Hz), 139.7, 137.1, 133.9 (d, $J_{C\text{-}F}$ = 19.5 Hz), 129.8, 128.6, 128.6 (d, $J_{C\text{-}F}$ = 6.9 Hz), 125.4 (q, $J_{C\text{-}F}$ = 3.8 Hz), 61.3, 14.32 (d, J = 30.8 Hz); ^{19}F{^{1}H} NMR (471 MHz, Chloroform-*d*) δ -62.4; HRMS (EI-quadrupole) $(M)^+$ Calcd for $C_{11}H_{11}F_3O$ 216.0757; Found 216.0757.

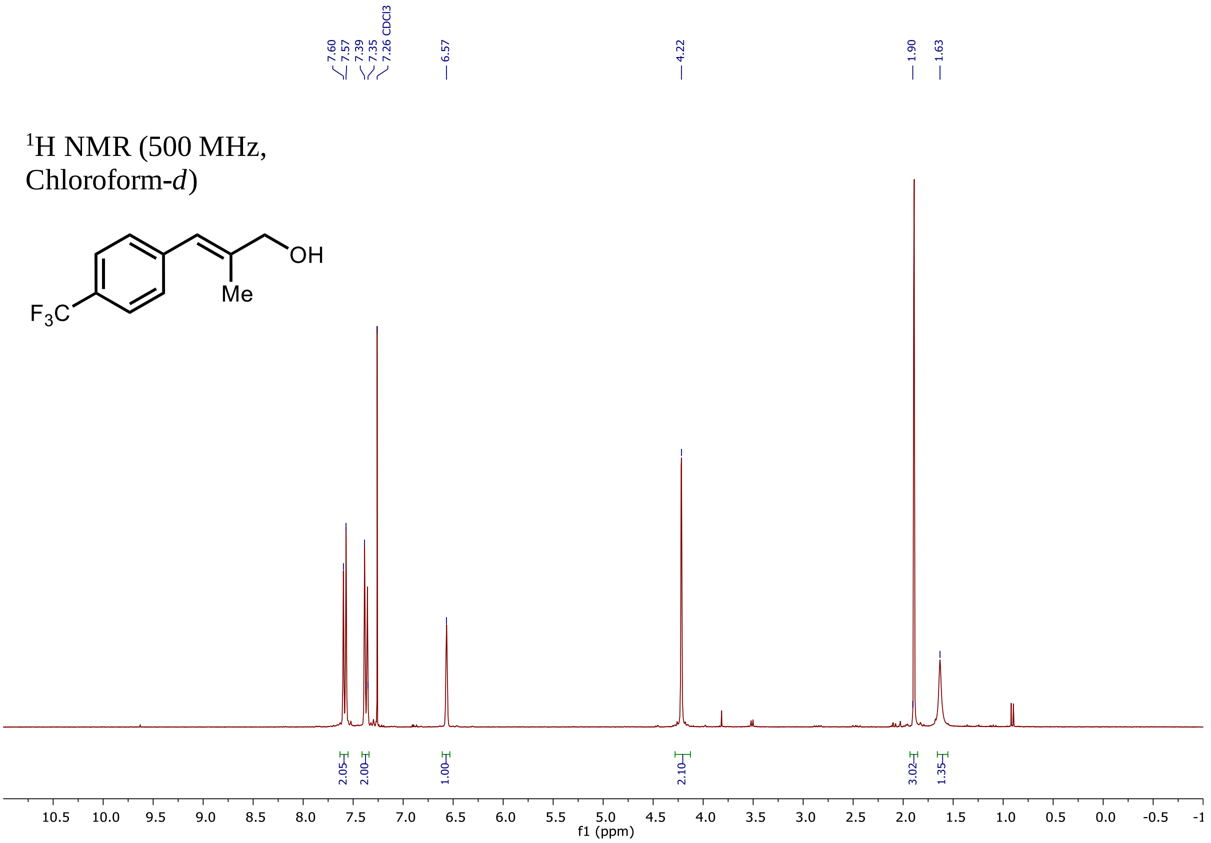

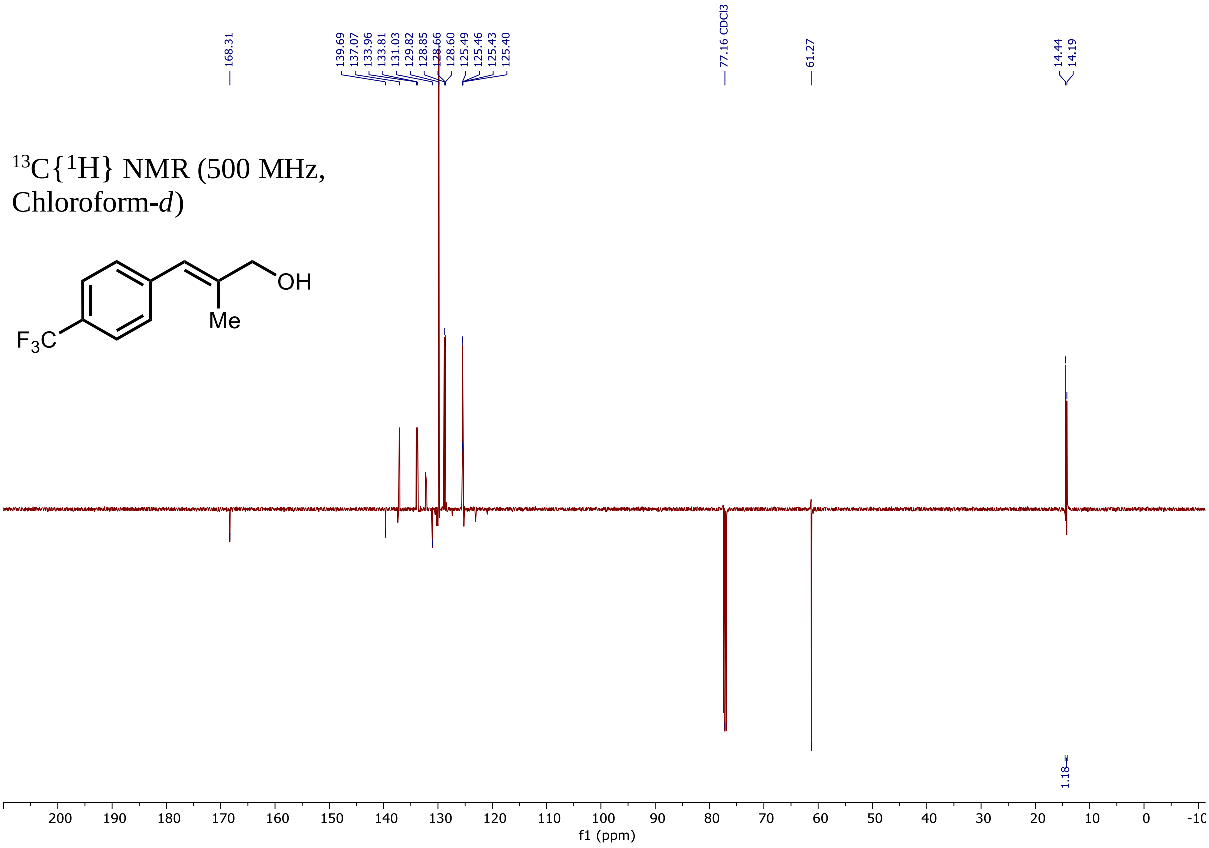
13C{1H} NMR (500 MHz, Chloroform-d)
OH
Me
F3C
168.31
139.69
137.07
133.96
133.81
131.03
129.82
128.85
128.66
128.60
125.49
125.46
125.43
125.40
77.16 CDCl3
61.27
14.44
14.19
1.18
f1 (ppm)

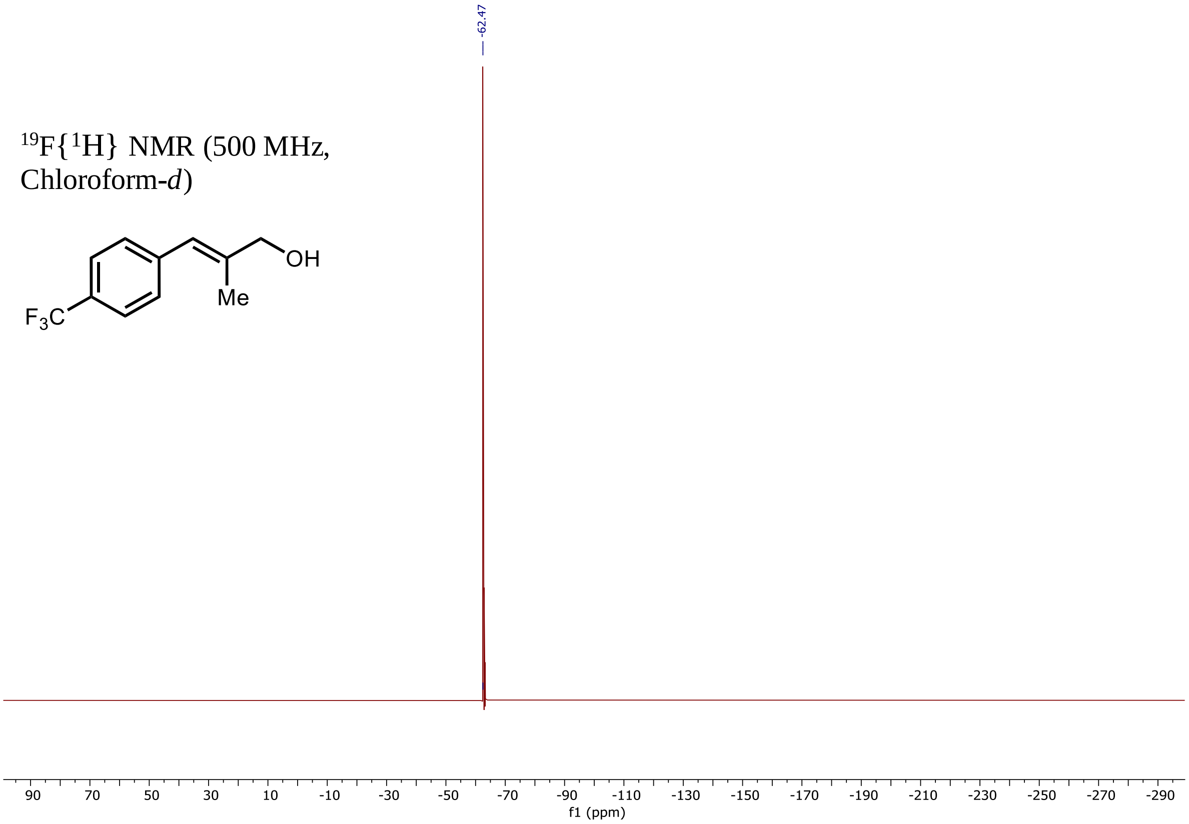
19F{1H} NMR (500 MHz, Chloroform-d)
OH
Me
F3C
-62.47
f1 (ppm)

(*E*)-3-(4-(hydroxymethyl)phenyl)-2-methylprop-2-en-1-ol

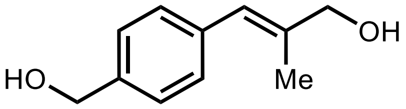

The title compound was prepared according to a general procedure 4 using ethyl (*E*)-3-(4-acetylphenyl)-2-methylacrylate (1.3 g, 6.0 mmol). The title compound was obtained as a white solid (800 mg, 75%); mp 63-64 °C; R_f = 0.15 (eluent = 30% EtOAc in petroleum ether); ν_{max} / cm^{-1} (film) 3213, 2904, 2852, 1440, 1421, 1354, 1001, 889, 815, 578, 518; ^{1}H NMR (500 MHz, Chloroform-*d*) δ 7.34 (d, *J* = 7.4 Hz, 2H), 7.28 (d, *J* = 7.5 Hz, 2H), 6.52 (s, 1H), 4.69 (s, 2H), 4.20 (s, 2H), 1.91 (s, 3H), 1.65 (s, 1H), 1.53 (s, 1H); ^{13}C{^{1}H} NMR (126 MHz, Chloroform-*d*) δ 139.1, 138.0, 137.2, 129.2, 127.1, 124.8, 69.1, 65.4, 15.5; HRMS (EI-quadrupole) $(M)^+$ Calcd for $C_{11}H_{14}O_2$ 178.0988; Found 178.0987.

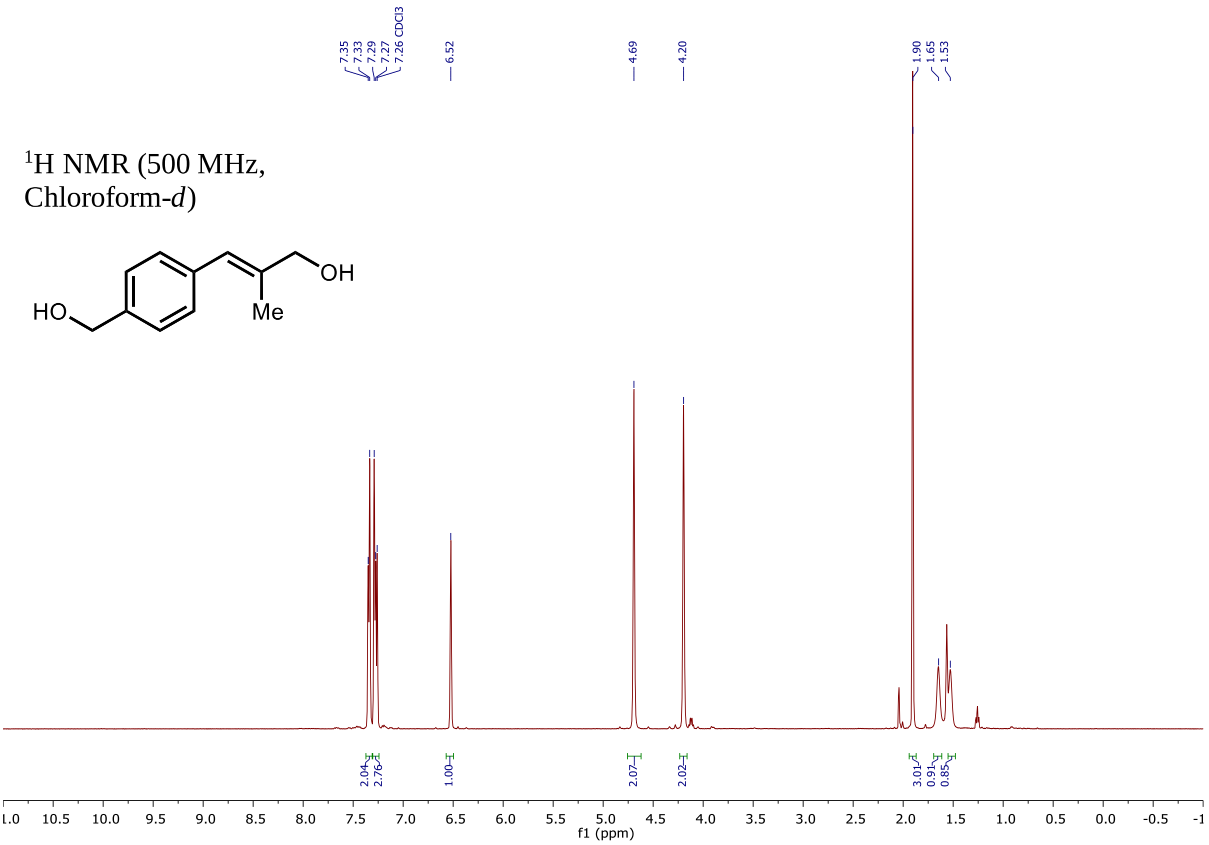

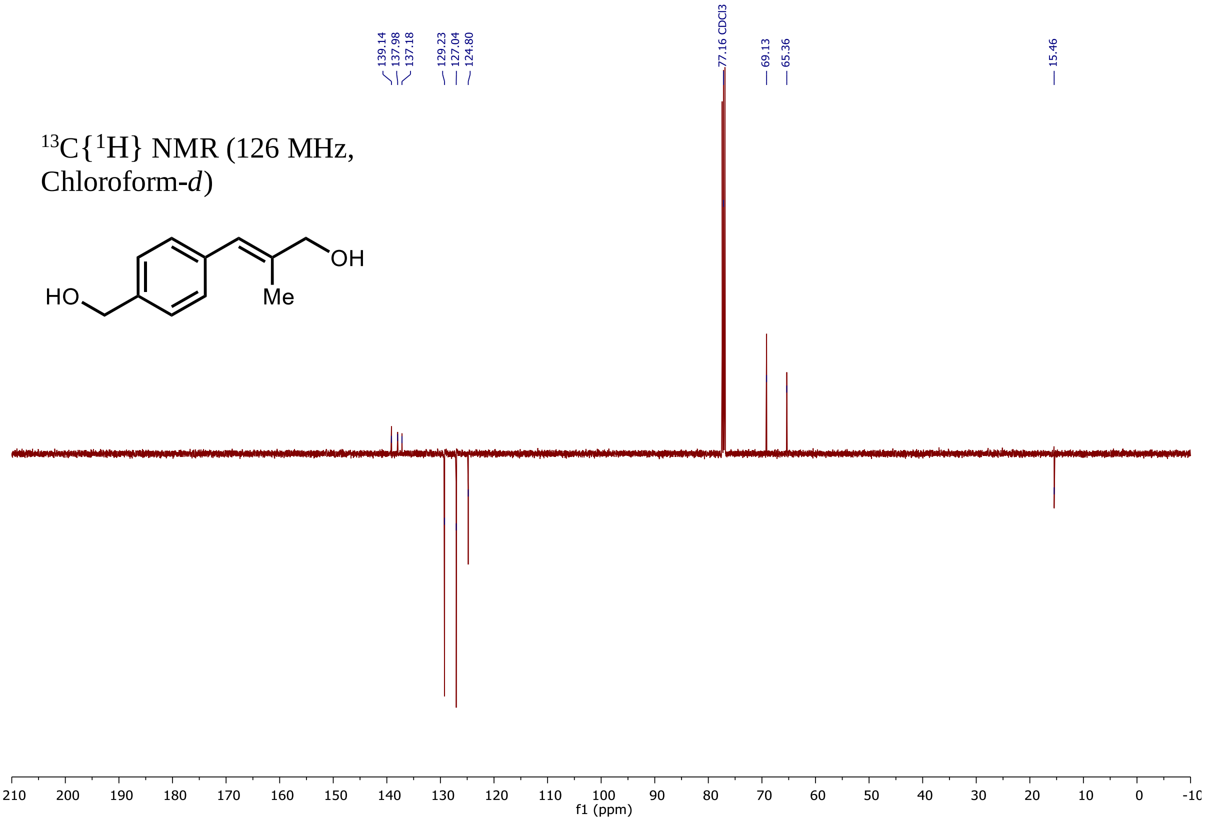

(*E*)-2-methyl-3-(4-vinylphenyl)prop-2-en-1-ol

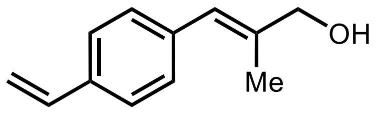

The title compound was prepared according to general procedure 4 using ethyl (*E*)-2-methyl-3-(4-vinylphenyl)acrylate (550 mg, 2.6 mmol). Purification by flash silica chromatography (eluent = 20% EtOAc in petroleum ether, 35 × 160 mm silica) gave the title compound as a white solid (330 mg, 74%); mp 53-55 °C; R_f = 0.42 (eluent = 20% EtOAc in petroleum ether); ν_{max} / cm^{-1} (film) 3333, 2918, 2853, 1504, 1487, 1072, 1009, 858; ^1H NMR (500 MHz, Chloroform-*d*) δ 7.42 – 7.35 (m, 2H), 7.29 – 7.22 (m, 2H), 6.71 (dd, *J* = 17.4, 10.6 Hz, 1H), 6.51 (s, 1H), 5.75 (dd, *J* = 17.6, 0.9 Hz, 1H), 5.24 (dd, *J* = 10.9, 0.9 Hz, 1H), 4.19 (d, *J* = 1.8 Hz, 2H), 1.92 (d, *J* = 1.5 Hz, 3H), 1.59 (s, 1H); $^{13}C\{^1H\}$ NMR (126 MHz, Chloroform-*d*) δ 137.9, 137.3, 136.7, 135.9, 129.2, 126.2, 124.9, 113.7, 69.2, 15.6; HRMS (CI-quadrupole) $(M + H)^+$ Calcd for $C_{12}H_{14}O$ 174.1039; Found 174.1040.

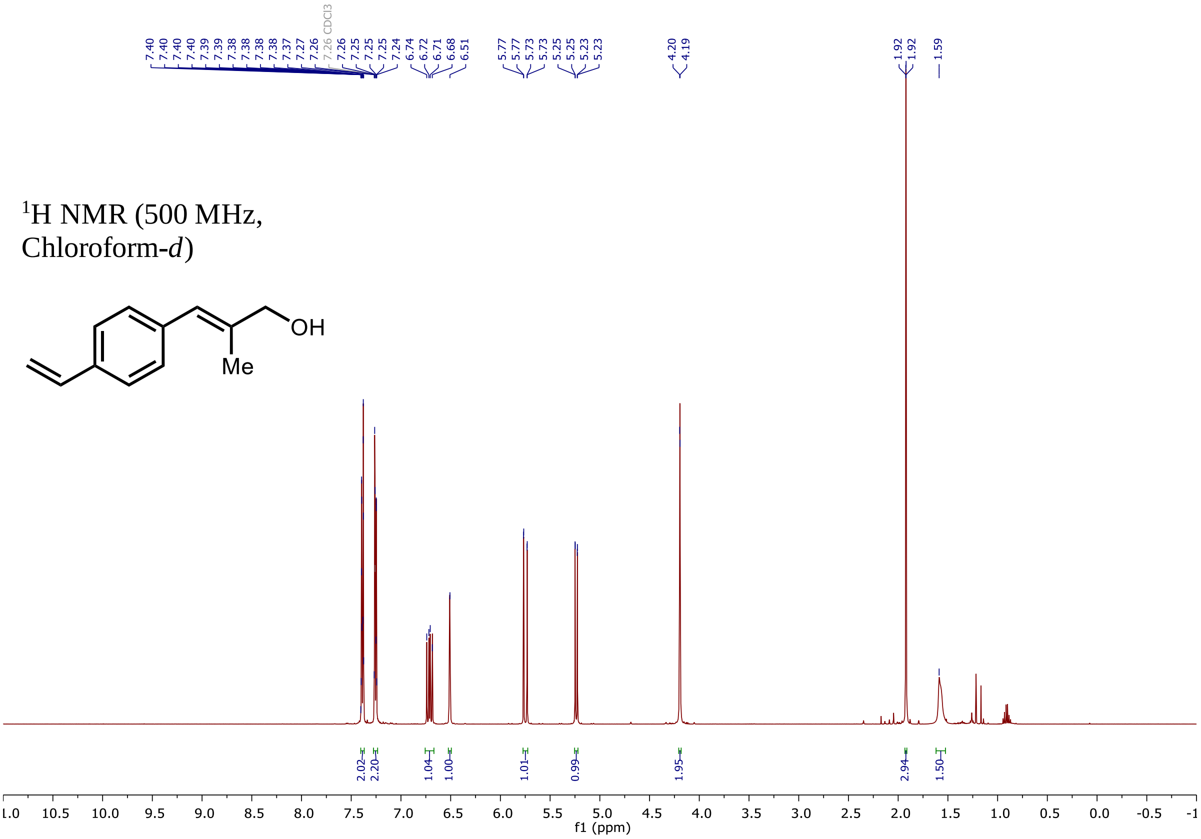
1H NMR (500 MHz, Chloroform-d)
OH
Me
7.40 7.40 7.40 7.39 7.39 7.38 7.38 7.38 7.38 7.37 7.27 7.26 7.26 CDCl3 7.26 7.25 7.25 7.25 7.25 7.24 6.74 6.72 6.71 6.68 6.51 5.77 5.77 5.73 5.73 5.25 5.25 5.23 5.23 4.20 4.19 1.92 1.92 1.59
2.02 2.20 1.04 1.00 1.01 0.99 1.95 2.94 1.50
f1 (ppm)

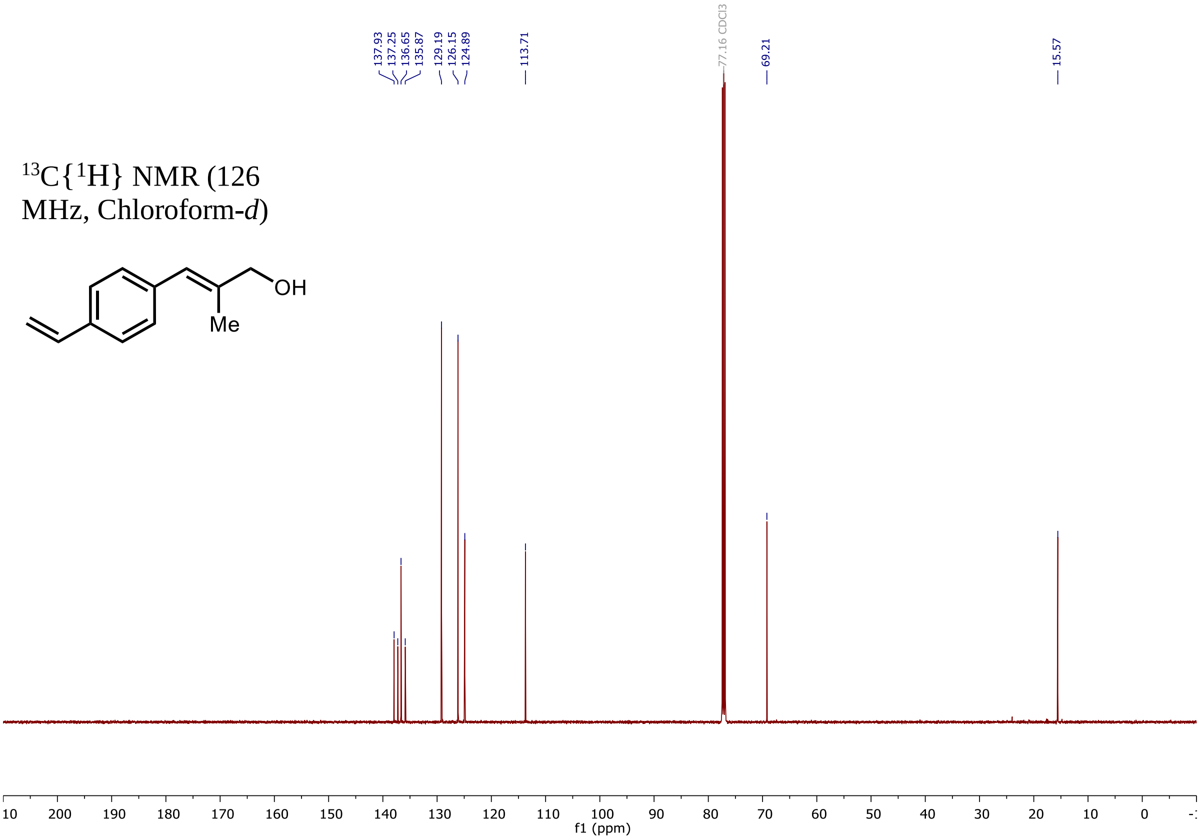
13C{1H} NMR (126 MHz, Chloroform-d)
OH
Me
137.93 137.25 136.65 135.87 129.19 126.15 124.89 113.71 77.16 CDCl3 69.21 15.57
f1 (ppm)

(*E*)-2-methyl-3-(naphthalen-1-yl)prop-2-en-1-ol

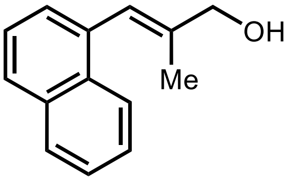

The title compound was prepared according to general procedure 4 using ethyl (*E*)-2-methyl-3-(naphthalen-1-yl)acrylate (1.285 g, 5.4 mmol). Purification by flash silica chromatography (eluent = 10% EtOAc in petroleum ether, 35 × 160 mm silica) gave the title compound as a white oil (573 mg, 54%); R_f = 0.43 (eluent = 10% EtOAc in petroleum ether); v_{max} / cm^{-1} (film) 3057, 2927, 2910, 2854, 1589, 1506, 1438, 1263, 1064, 867, 848, 796, 777, 732, 653, 578, 557, 420, 406; ^{1}H NMR (500 MHz, Chloroform-*d*) δ 8.00 – 7.95 (m, 1H), 7.89 – 7.83 (m, 1H), 7.77 (ddq, *J* = 8.2, 1.3, 0.6 Hz, 1H), 7.52 – 7.41 (m, 3H), 7.34 (dt, *J* = 7.1, 1.2 Hz, 1H), 6.98 (qt, *J* = 1.7, 0.8 Hz, 1H), 4.34 (dd, *J* = 1.4, 0.6 Hz, 2H), 1.77 (dd, *J* = 1.4, 0.6 Hz, 3H), 1.73 – 1.61 (m, 1H); ^{13}C{^{1}H} NMR (126 MHz, Chloroform-*d*) δ 139.6, 134.9, 133.7, 132.1, 128.5, 127.3, 126.8, 125.91, 125.87, 125.4, 125.1, 123.0, 68.7, 15.5; HRMS (EI-quadrupole) $(M)^+$ Calcd for $C_{14}H_{14}O$ 198.1039; Found 198.1036.

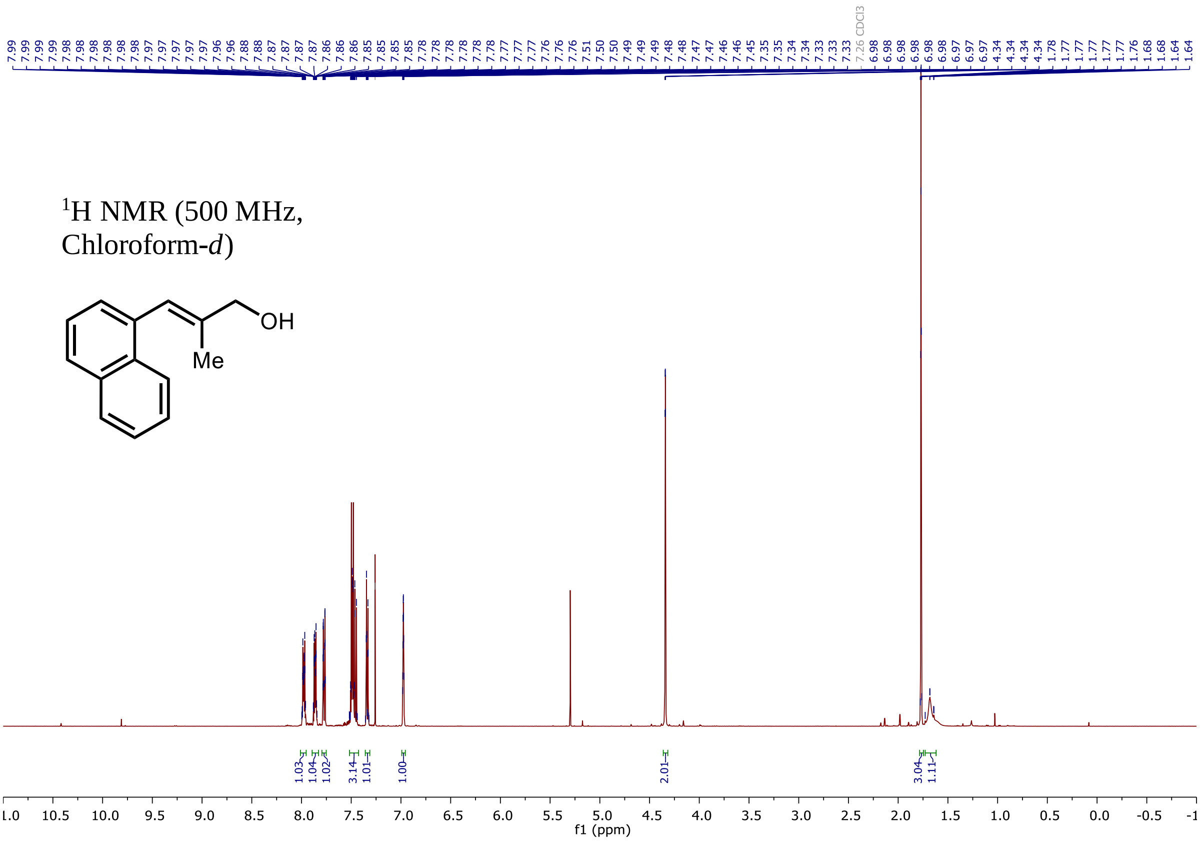

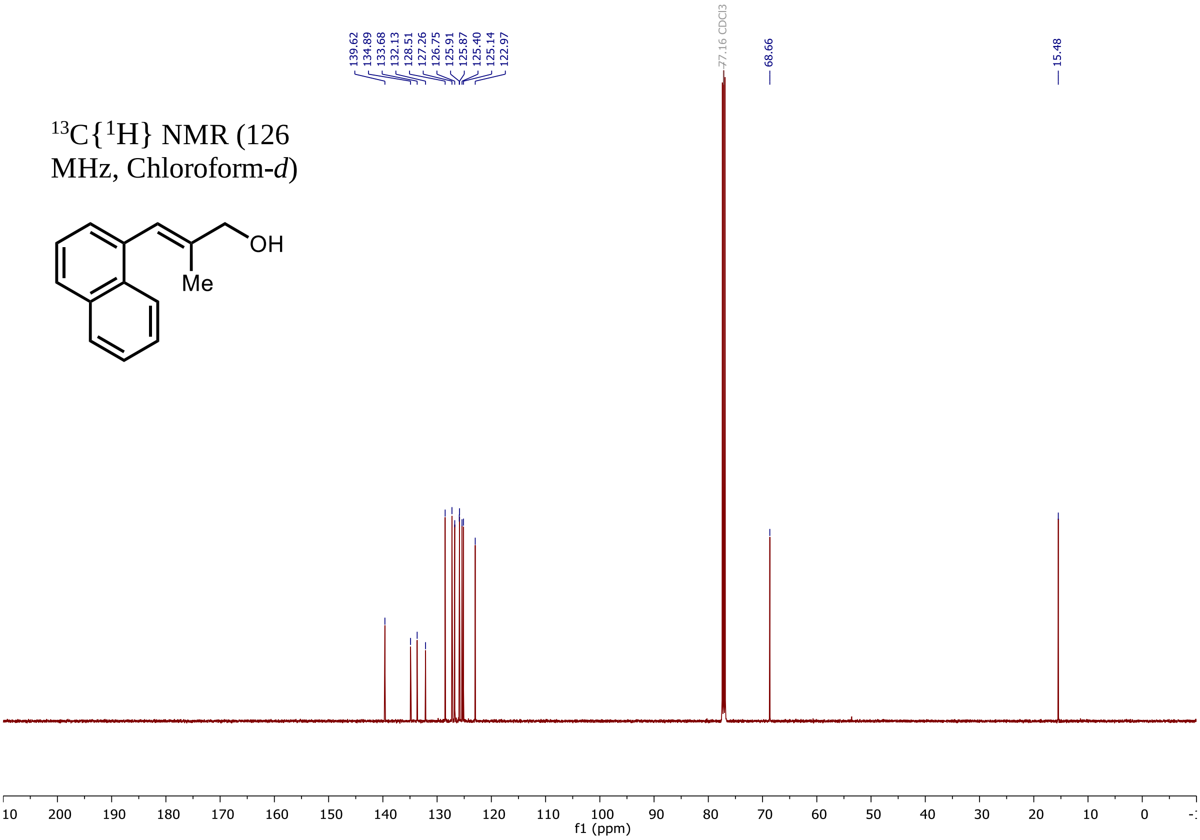

(*E*)-2-methyl-3-(naphthalen-2-yl)prop-2-en-1-ol

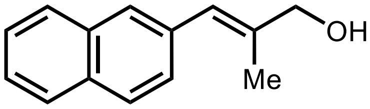

The title compound was prepared according to general procedure 4 using ethyl (*E*)-2-methyl-3-(naphthalen-2-yl)acrylate (2 g, 8.3 mmol). Purification by flash silica chromatography (eluent = 20% EtOAc in petroleum ether, 35 × 160 mm silica) gave the title compound as a white semi solid (954 mg, 58%); R_f = 0.52 (eluent = 20% EtOAc in petroleum ether); ν_{max} / cm^{-1} (film) 3230, 3049, 2833, 1591, 1500, 1375, 1352, 1273, 1215, 1184, 1159, 1124, 1066, 1006, 950, 866, 759, 742, 678, 648, 624, 596, 505, 416, 410; ^{1}H NMR (500 MHz, Chloroform-*d*) δ 7.83 – 7.80 (m, 4H), 7.48 – 7.41 (m, 3H), 6.69 (s, 1H), 4.26 (s, 2H), 1.99 (s, 3H), 1.56 (s, 1H); $^{13}C\{^{1}H\}$ NMR (126 MHz, Chloroform-*d*) δ 138.3, 135.2, 133.5, 132.2, 128.0, 127.8, 127.74, 127.66, 127.5, 126.2, 125.9, 125.2, 69.2, 15.6; HRMS (EI-quadrupole) $(M)^+$ Calcd for $C_{14}H_{14}O$ 198.1039; Found 198.1036.

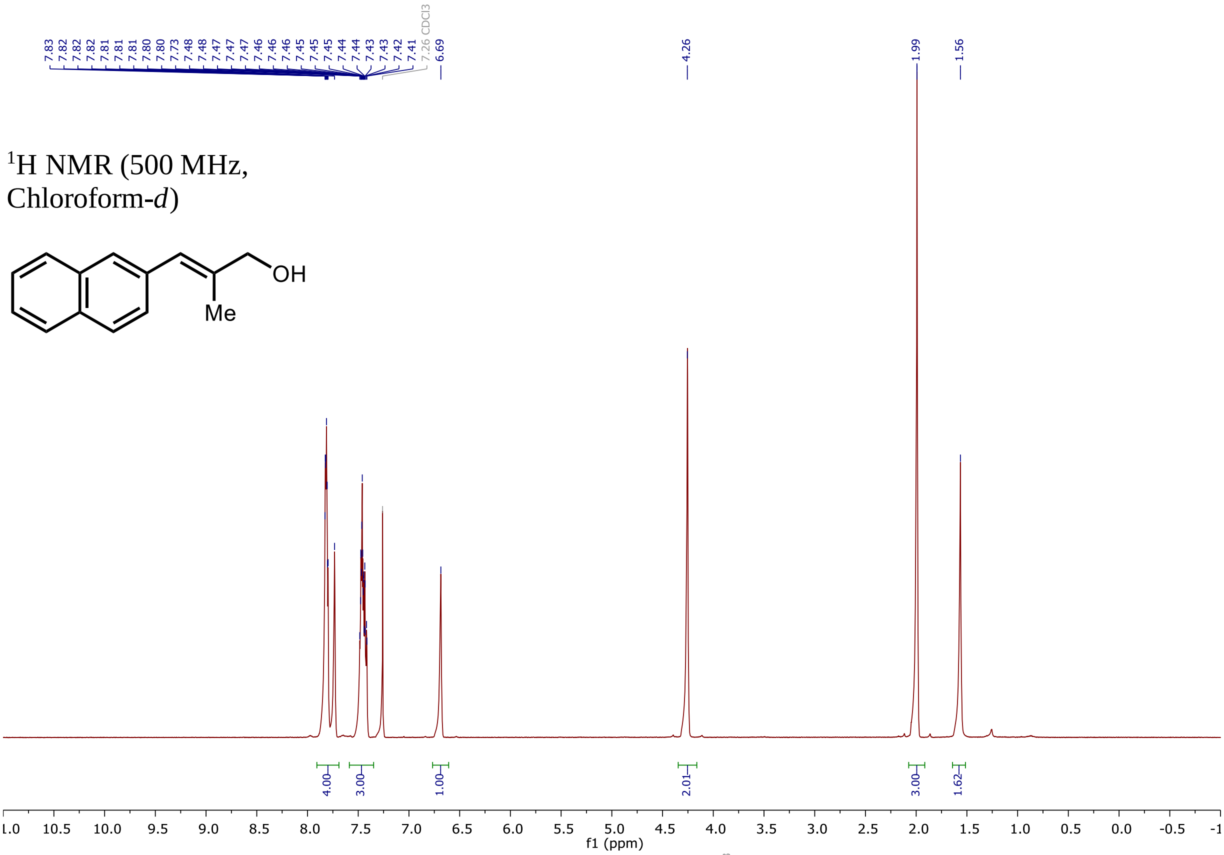

^{1}H NMR (500 MHz, Chloroform-*d*)
OH
Me
7.83 7.82 7.82 7.82 7.81 7.81 7.81 7.80 7.73 7.48 7.48 7.47 7.47 7.47 7.46 7.46 7.46 7.45 7.45 7.45 7.44 7.44 7.43 7.43 7.42 7.41 7.26 CDCl3 6.69
4.26
1.99
1.56
4.00 3.00 1.00 2.01 3.00 1.62
f1 (ppm)

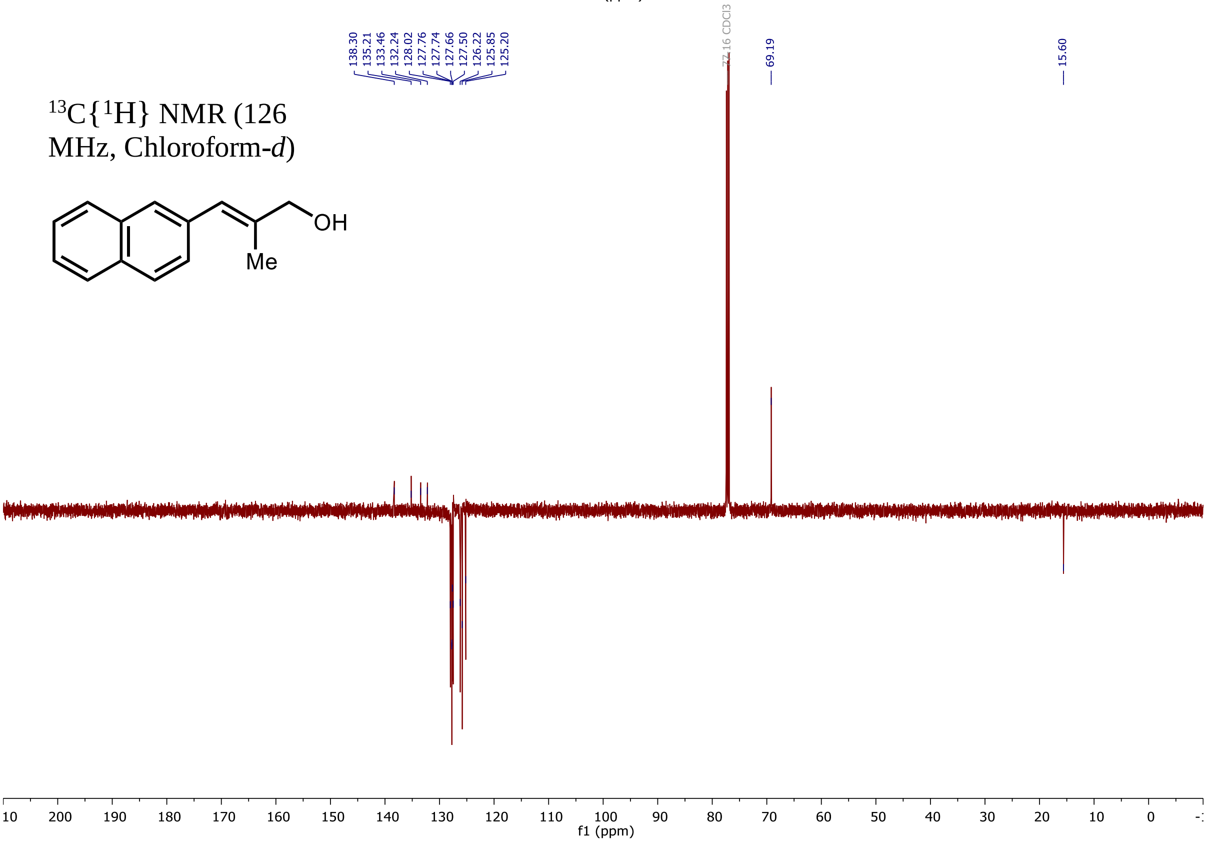

^{13}C{^{1}H} NMR (126 MHz, Chloroform-*d*)
OH
Me
138.30 135.21 133.46 132.24 128.02 127.76 127.74 127.66 127.50 126.22 125.85 125.20
77.16 CDCl3
69.19
15.60
f1 (ppm)

(*E*)-3-(furan-2-yl)-2-methylprop-2-en-1-ol

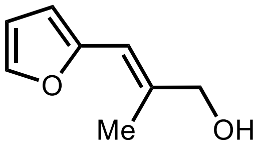

The title compound was prepared according to general procedure 4 using ethyl (*E*)-2-methyl-3-(furan-2-yl)acrylate (1 g, 5.5 mmol). Purification by flash silica chromatography (eluent = 20% EtOAc in petroleum ether, 35 × 160 mm silica) gave the title compound as an orange oil (422 mg, 56%); R_f = 0.61 (eluent = 20% EtOAc in petroleum ether); ^{1}H NMR (500 MHz, Chloroform-*d*) δ 7.38 (d, *J* = 1.2 Hz, 1H), 6.40 (ddd, *J* = 3.4, 1.8, 0.5 Hz, 1H), 6.37 – 6.31 (m, 1H), 6.28 (d, *J* = 3.4 Hz, 1H), 4.17 (s, 2H), 2.01 (s, 3H), 1.54 (s, 1H); ^{13}C{^{1}H} NMR (126 MHz, Chloroform-*d*) δ 153.2, 141.4, 136.4, 113.9, 111.3, 108.8, 68.8, 16.0. Spectroscopic data in accordance with that stated in the literature.[8]

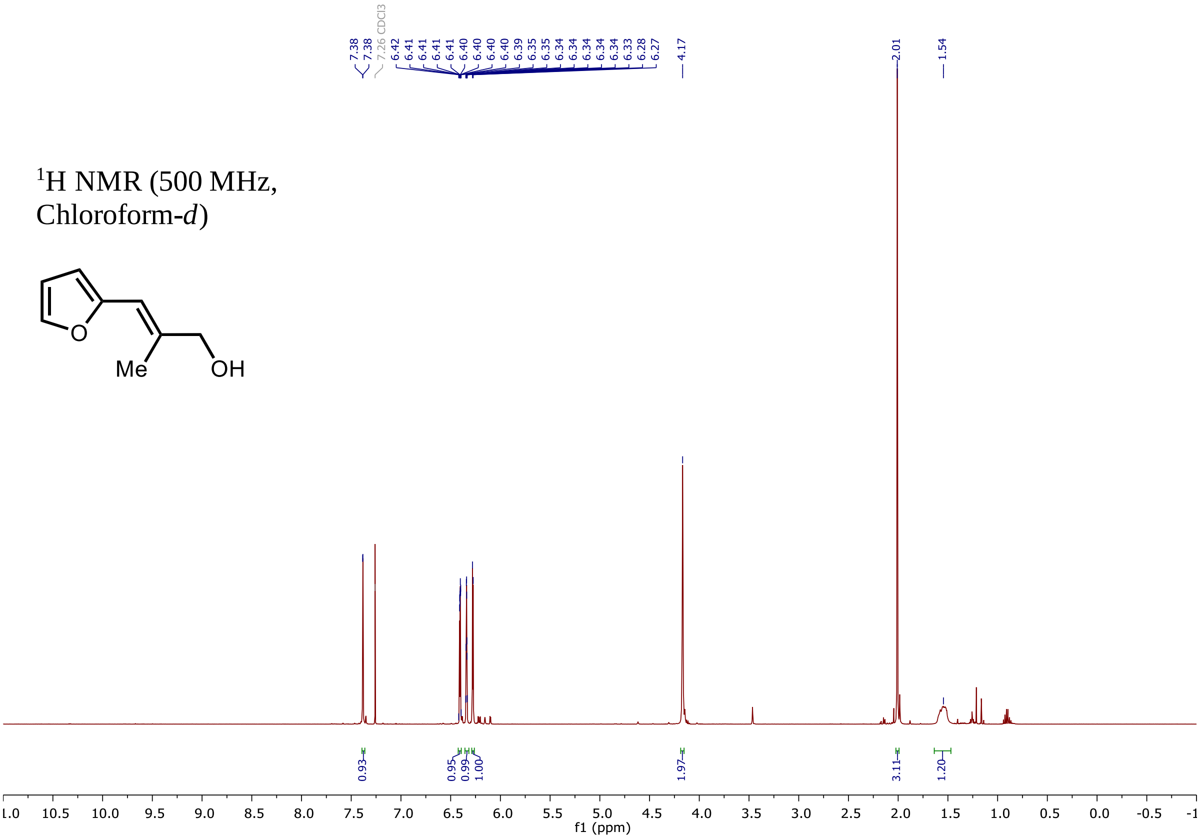

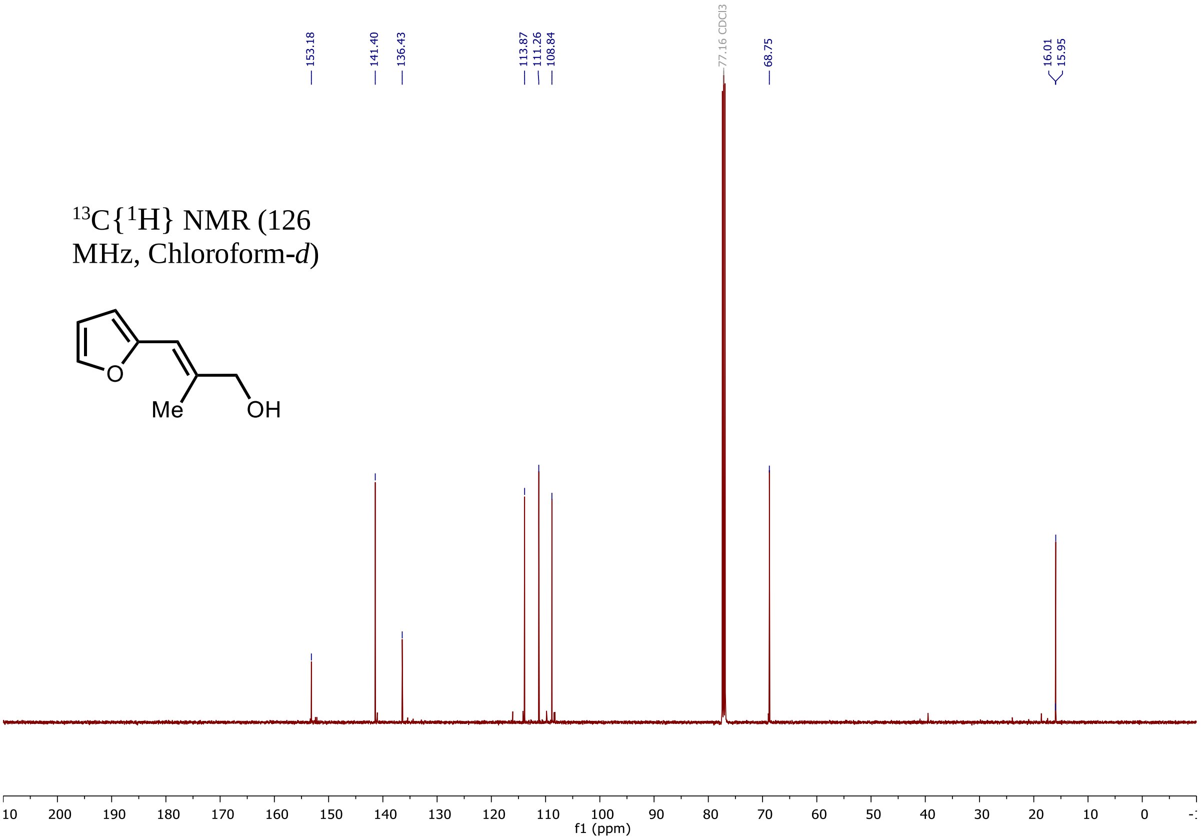

(*E*)-2-methyl-3-(thiophen-2-yl)prop-2-en-1-ol

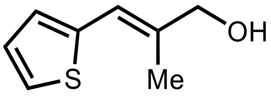

The title compound was prepared according to a general procedure 4 using ethyl (*E*)-2-methyl-3-(thiophen-2-yl)acrylate (1.27 g, 6.9 mmol). Purification by flash silica chromatography (eluent = 10-30% EtOAc in petroleum ether, 35 × 160 mm silica) gave the title compound as a yellow solid (750 mg, 70 %); mp 44-46 °C; R_f = 0.15 (eluent = 10 % EtOAc in petroleum ether); v_{max} / cm^{-1} (film) 3296, 3205, 2912, 2852, 1436, 1342, 1244, 1015, 873, 695, 513; ^1H NMR (500 MHz, Chloroform-*d*) δ 7.27 – 7.25 (m, 1H), 7.01 (ddd, *J* = 4.0, 3.2, 1.9 Hz, 2H), 6.67 (m, 1H), 4.19 (d, *J* = 4.7 Hz, 2H), 2.01 (s, 3H), 1.51 (s, 1H): $^{13}C\{^1H\}$ NMR (126 MHz, Chloroform-*d*) δ 140.8, 136.2, 127.2, 127.0, 124.9, 118.6, 69.1, 16.1; HRMS (EI-quadrupole) $(M)^+$ Calcd for $C_8H_{10}OS$ 154.0447; Found 154.0445.

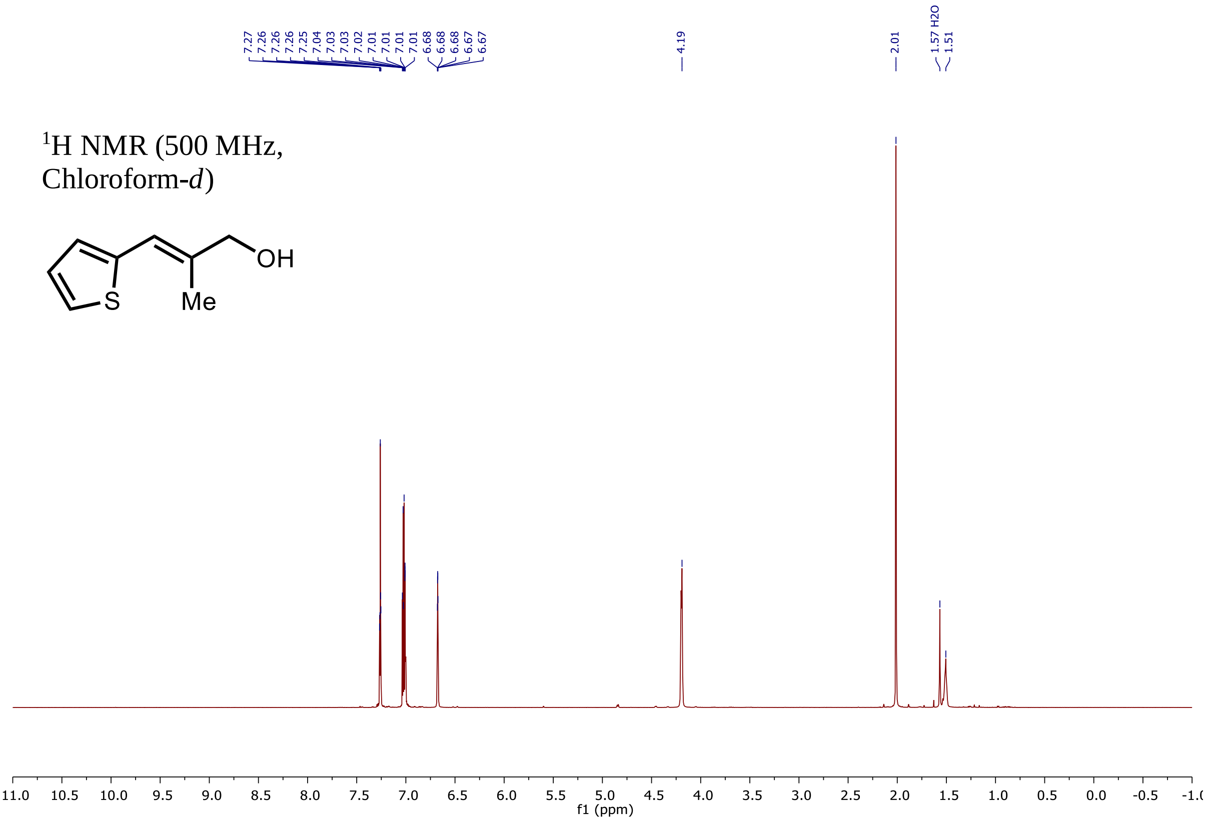
7.27
7.26
7.26
7.26
7.25
7.04
7.03
7.03
7.02
7.01
7.01
7.01
7.01
6.68
6.68
6.68
6.67
6.67
4.19
2.01
1.57 H2O
1.51
¹H NMR (500 MHz, Chloroform-d)
OH
S
Me
11.0 10.5 10.0 9.5 9.0 8.5 8.0 7.5 7.0 6.5 6.0 5.5 5.0 4.5 4.0 3.5 3.0 2.5 2.0 1.5 1.0 0.5 0.0 -0.5
f1 (ppm)

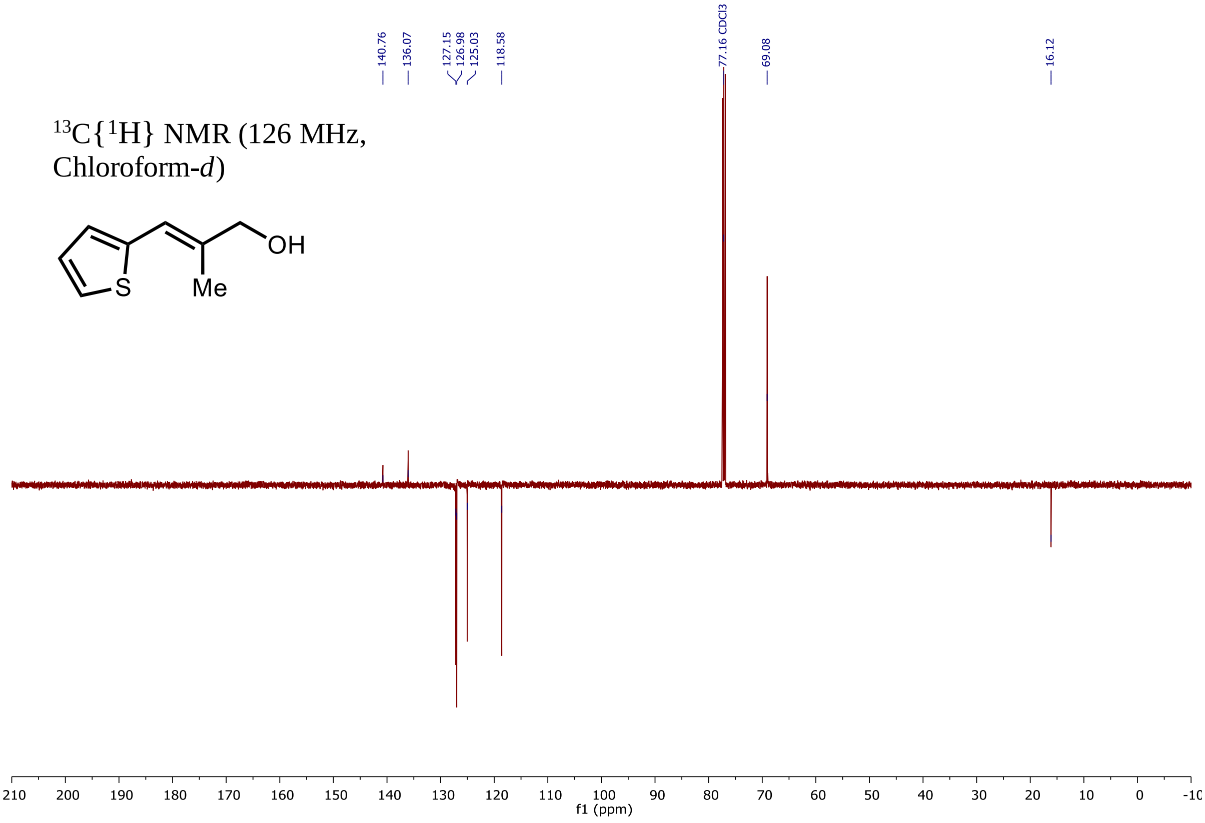
140.76
136.07
127.15
126.98
125.03
118.58
77.16 CDCl3
69.08
16.12
¹³C{¹H} NMR (126 MHz, Chloroform-d)
OH
S
Me
210 200 190 180 170 160 150 140 130 120 110 100 90 80 70 60 50 40 30 20 10 0
f1 (ppm)

(*E*)-3-(furan-3-yl)-2-methylprop-2-en-1-ol

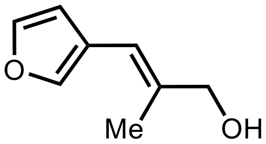

The title compound was prepared according to general procedure 4 using ethyl (*E*)-3-(furan-3-yl)-2-methylacrylate (3.15 g, 17.5 mmol). Purification by flash silica chromatography (eluent = 25% EtOAc in petroleum ether, 35 × 160 mm silica) gave the title compound as a yellow oil (1.72 g, 71%); R_f = 0.61 (eluent = 25% EtOAc in petroleum ether); ν_{max} / cm^{-1} (film) 3331, 2916, 2860, 1502, 1446, 1375, 1242, 1176, 1147, 1062, 999, 869, 823, 777, 754, 725, 638, 594, 467, 420, 403; ^{1}H NMR (500 MHz, Chloroform-*d*) δ 7.45 (s, 1H), 7.40 – 7.39 (m, 1H), 6.47 (dt, *J* = 1.9, 0.9 Hz, 1H), 6.25 (dtd, *J* = 2.1, 1.5, 0.8 Hz, 1H), 4.15 (s, 2H), 1.89 (s, 3H), 1.70 (bs, 1H); ^{13}C{^{1}H} NMR (126 MHz, Chloroform-*d*) δ 142.8, 140.9, 136.8, 122.6, 115.5, 111.1, 69.0, 15.9; HRMS (EI-quadrupole) (M)$^+$ Calcd for $C_8H_{10}O_2$ 138.0675; Found 138.0674.

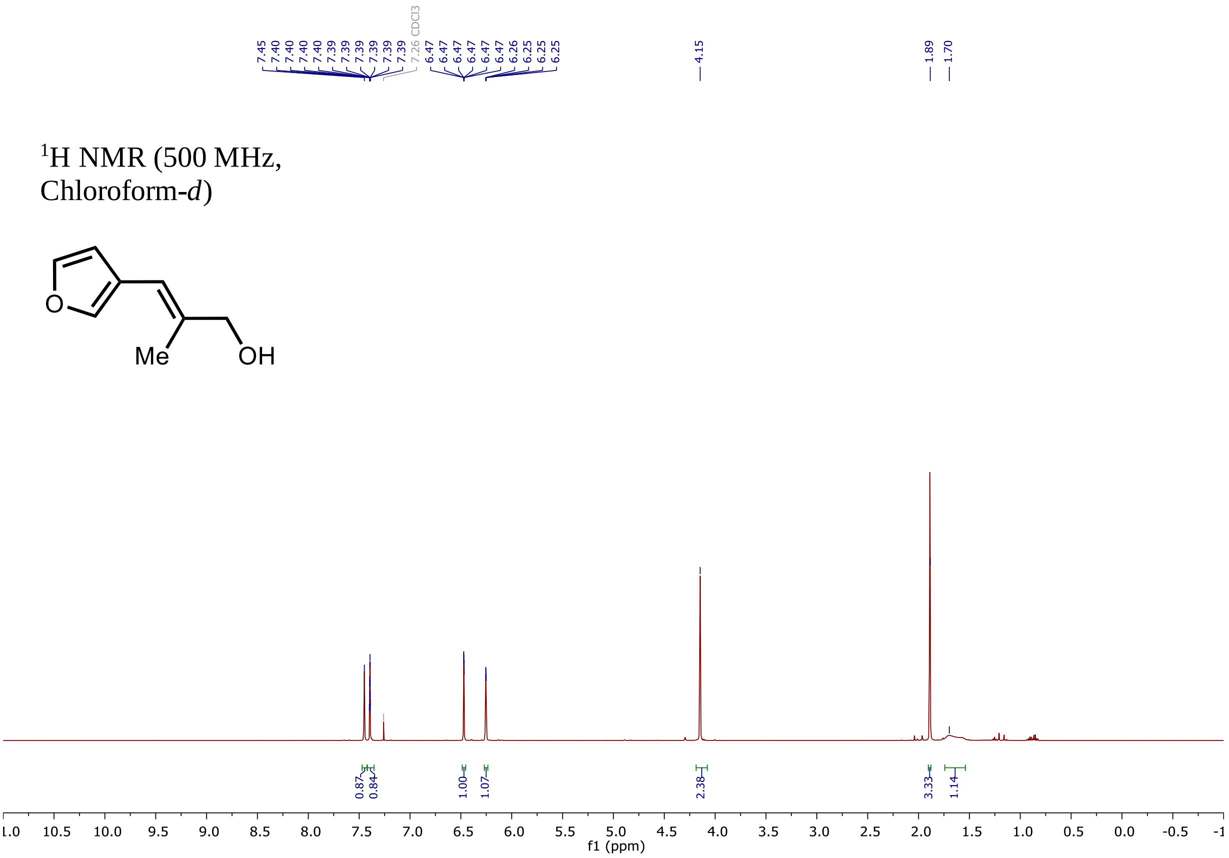

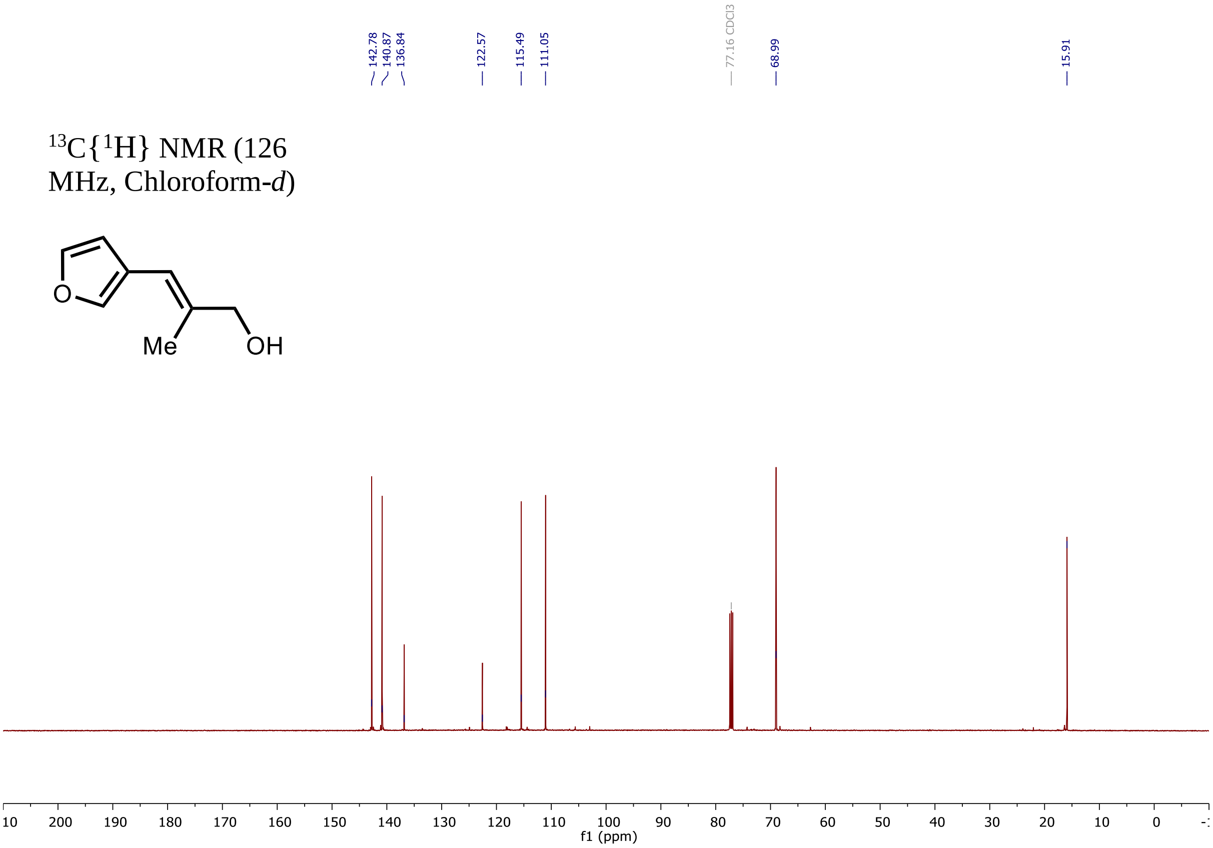

(*E*)-2-methyl-3-(thiophen-3-yl)prop-2-en-1-ol

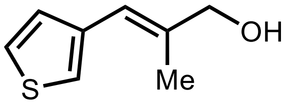

The title compound was prepared according to a general procedure 4 using ethyl (*E*)-2-methyl-3-(thiophen-2-yl)acrylate (1.27 g, 6.9 mmol). Purification by flash silica chromatography (eluent = 10-30% EtOAc in petroleum ether, 35 × 160 mm silica) gave the title compound as a clear liquid (525 mg, 50 %); R_f = 0.2 (eluent = 10 % EtOAc in petroleum ether); ν_{max} / cm^{-1} (film) 3296, 3205, 2912, 2852, 1436, 1342, 1244, 1015, 873, 695, 513; ^{1}H NMR (500 MHz, Chloroform-*d*) δ 7.29 (dd, *J* = 4.9, 2.9 Hz, 1H), 7.17 (d, *J* = 2.9 Hz, 1H), 7.11 (dd, *J* = 4.6, 1.3 Hz, 1H), 6.48 (s, 1H), 4.18 (s, 2H), 1.95 (s, 3H), 1.55 (s, 1H); $^{13}C\{^{1}H\}$ NMR (126 MHz, Chloroform-*d*) δ 138.7, 137.0, 128.8, 125.1, 122.7, 119.6, 69.2, 15.9; HRMS (EI-quadrupole) $(M)^+$ Calcd for $C_8H_{10}OS$ 154.0446; Found 154.0447.

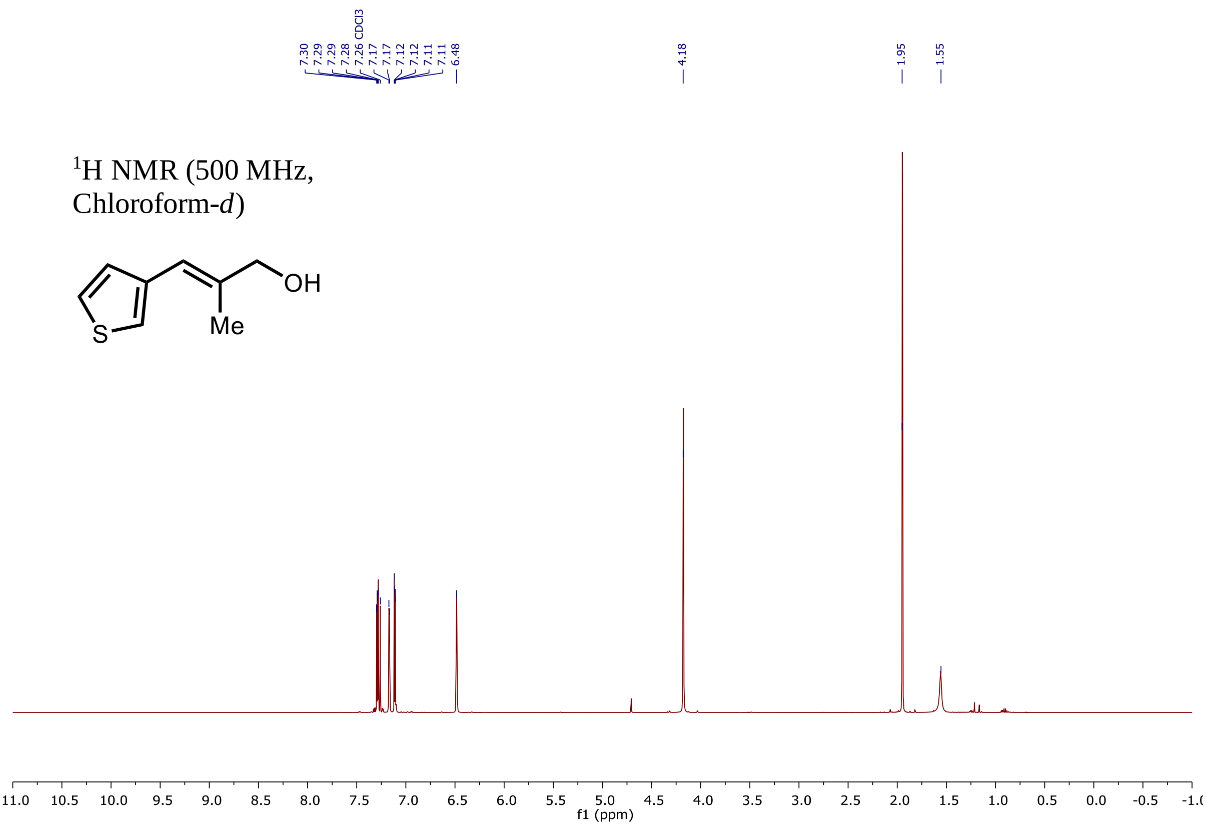
7.30
7.29
7.29
7.28
7.26 CDCl3
7.17
7.17
7.12
7.12
7.11
7.11
6.48
4.18
1.95
1.55
¹H NMR (500 MHz, Chloroform-d)
OH
Me
S
11.0 10.5 10.0 9.5 9.0 8.5 8.0 7.5 7.0 6.5 6.0 5.5 5.0 4.5 4.0 3.5 3.0 2.5 2.0 1.5 1.0 0.5 0.0 -0.5 -1.0
f1 (ppm)

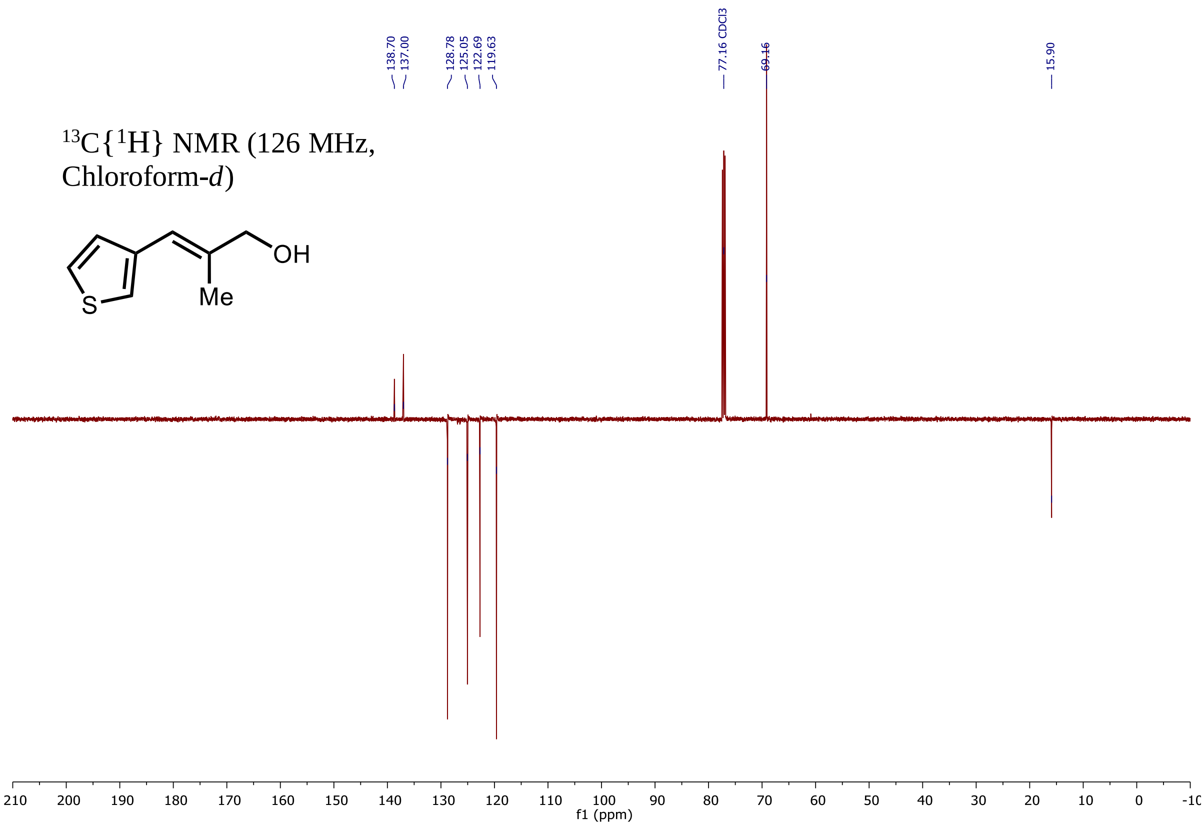
138.70
137.00
128.78
125.05
122.69
119.63
77.16 CDCl3
69.16
15.90
¹³C{¹H} NMR (126 MHz, Chloroform-d)
OH
Me
S
210 200 190 180 170 160 150 140 130 120 110 100 90 80 70 60 50 40 30 20 10 0 -10
f1 (ppm)

(*E*)-2-methyl-3-(pyridin-3-yl)prop-2-en-1-ol

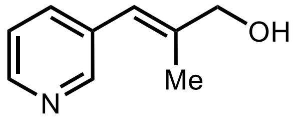

The title compound was prepared according to general procedure 4 using ethyl (*E*)-2-methyl-3-(pyridin-3-yl)acrylate (1.68 g, 8.8 mmol). Purification by flash silica chromatography (eluent = 80% EtOAc in petroleum ether, 35 × 160 mm silica) gave the title compound as a yellow oil (421 mg, 31%); R_f = 0.22 (eluent = 80% EtOAc in petroleum ether); ν_{max} / cm^{-1} (film) 3232, 2912, 2852, 1589, 1570, 1477, 1444, 1413, 1226, 1193, 1105, 1074, 952, 842, 786, 709, 628, 576, 414. 408, 401; ^{1}H NMR (500 MHz, Chloroform-*d*) δ 8.51 – 8.50 (m, 1H), 8.43 (dd, *J* = 4.8, 1.7 Hz, 1H), 7.59 (dddd, *J* = 7.9, 2.3, 1.6, 0.7 Hz, 1H), 7.26 -7.25 (m, 1H), 6.50 (s, 1H), 4.21 (s, 2H), 2.58 (bs, 1H), 1.88 (s, 3H); ^{13}C{^{1}H} NMR (126 MHz, Chloroform-*d*) δ 150.0, 147.3, 140.8, 136.1, 133.6, 123.3, 120.8, 68.3, 15.4; HRMS (ES-TOF) $(M + H)^+$ Calcd for $C_9H_{12}NO$ 150.0919; Found 150.0924.

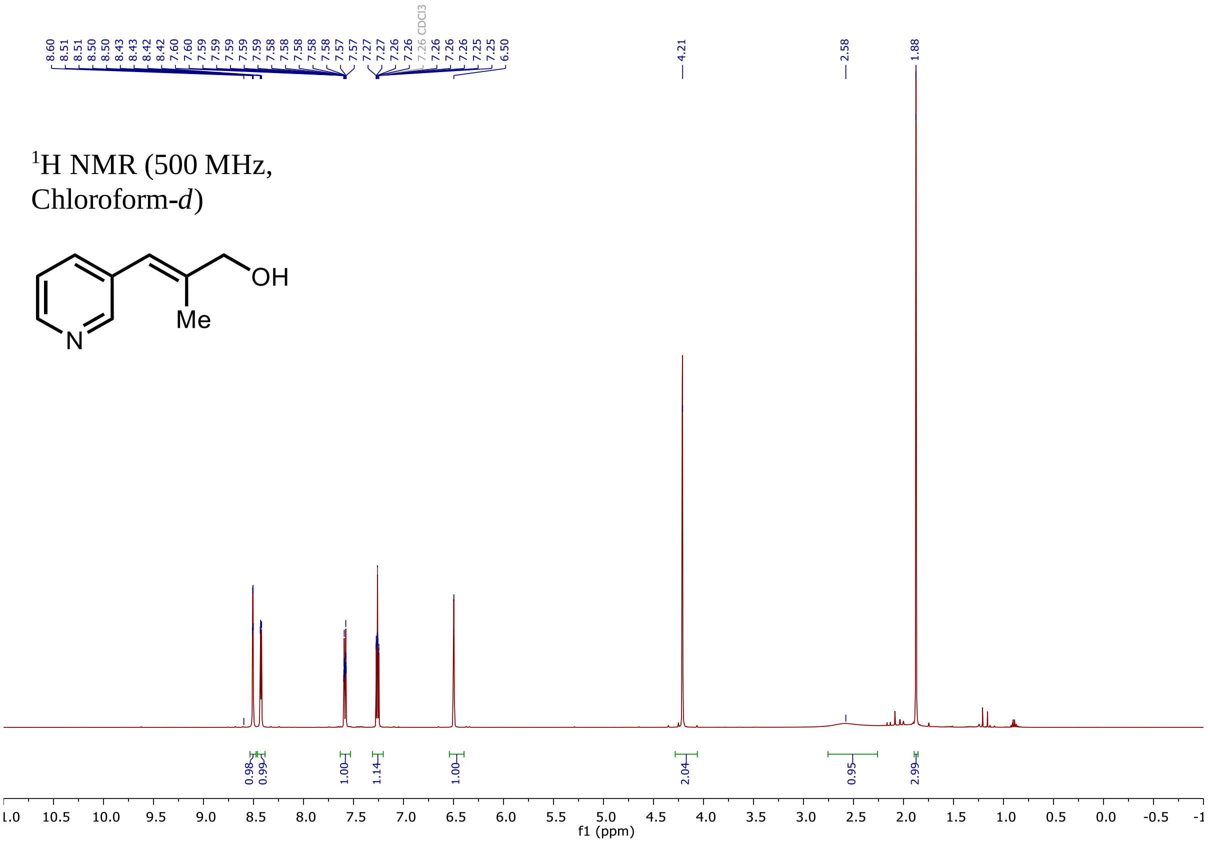

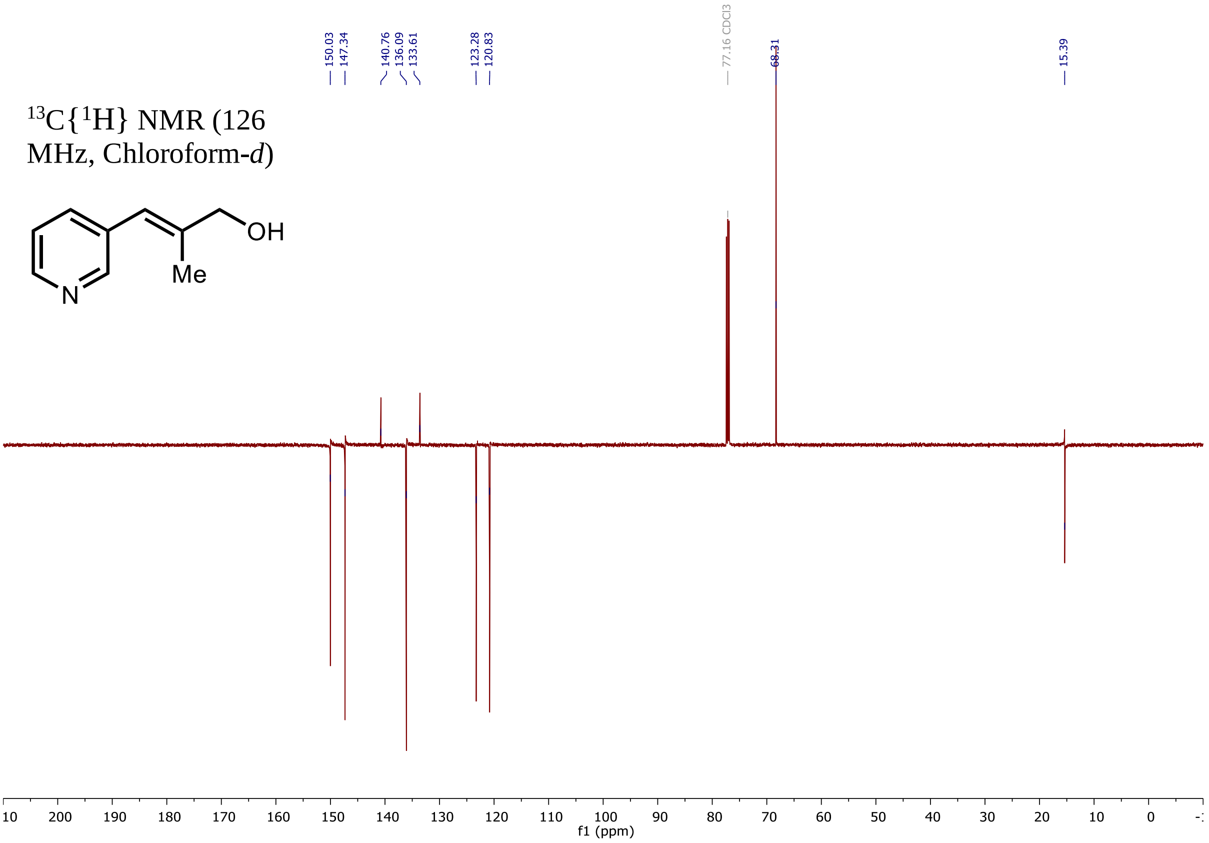

(*E*)-2-methyl-4-phenylbut-2-en-1-ol

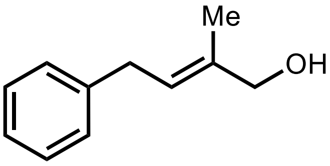

The title compound was prepared according to general procedure 4 using ethyl (*E*)-2-methyl-4-phenylbut-2-enoate (700 mg, 3.4 mmol). Purification by flash silica chromatography (eluent = 10-30% EtOAc in petroleum ether, 35 × 160 mm silica) gave the title compound as a colourless oil (218 mg, 39%); R_f = 0.27 (eluent = 10% EtOAc in petroleum ether); ^{1}H NMR (500 MHz, Chloroform-*d*) δ 7.22 – 7.15 (m, 2H), 7.13 – 7.05 (m, 3H), 5.51 (tp, *J* = 7.3, 1.3 Hz, 1H), 3.93 (s, 2H), 3.30 (d, *J* = 7.5 Hz, 2H), 1.87 (bs, 1H), 1.68 (s, 3H); $^{13}C\{^{1}H\}$ NMR (126 MHz, Chloroform-*d*) δ 141.1, 135.7, 128.5, 128.4, 126.0, 124.7, 68.7, 34.0, 13.9. Spectroscopic data in accordance with that stated in the literature.[6]

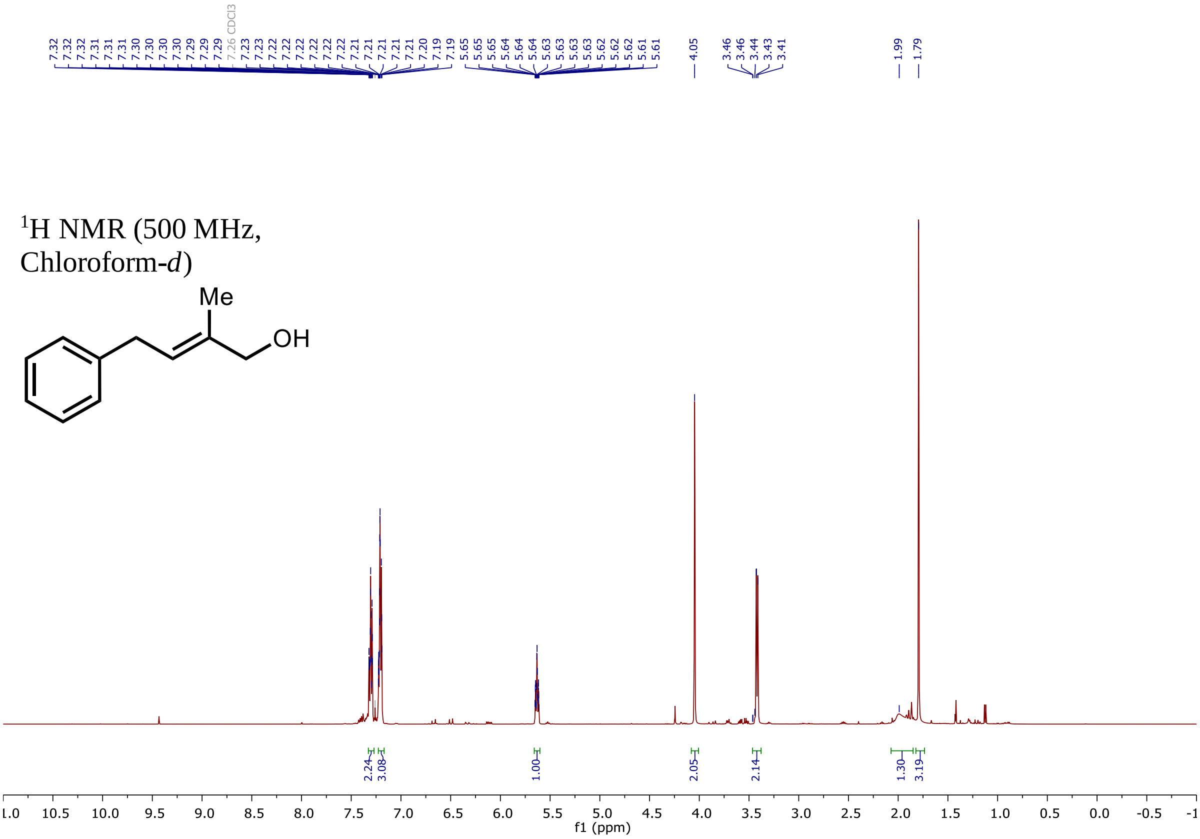
1H NMR (500 MHz, Chloroform-d)
Me
OH
7.32 7.32 7.32 7.31 7.31 7.31 7.30 7.30 7.30 7.30 7.29 7.29 7.29 7.26 CDCl3 7.23 7.23 7.22 7.22 7.22 7.22 7.22 7.22 7.21 7.21 7.21 7.21 7.21 7.20 7.19 7.19 5.65 5.65 5.65 5.64 5.64 5.64 5.63 5.63 5.63 5.63 5.62 5.62 5.62 5.61 5.61
4.05
3.46 3.46 3.44 3.43 3.41
1.99
1.79
2.24 3.08 1.00 2.05 2.14 1.30 3.19
f1 (ppm)

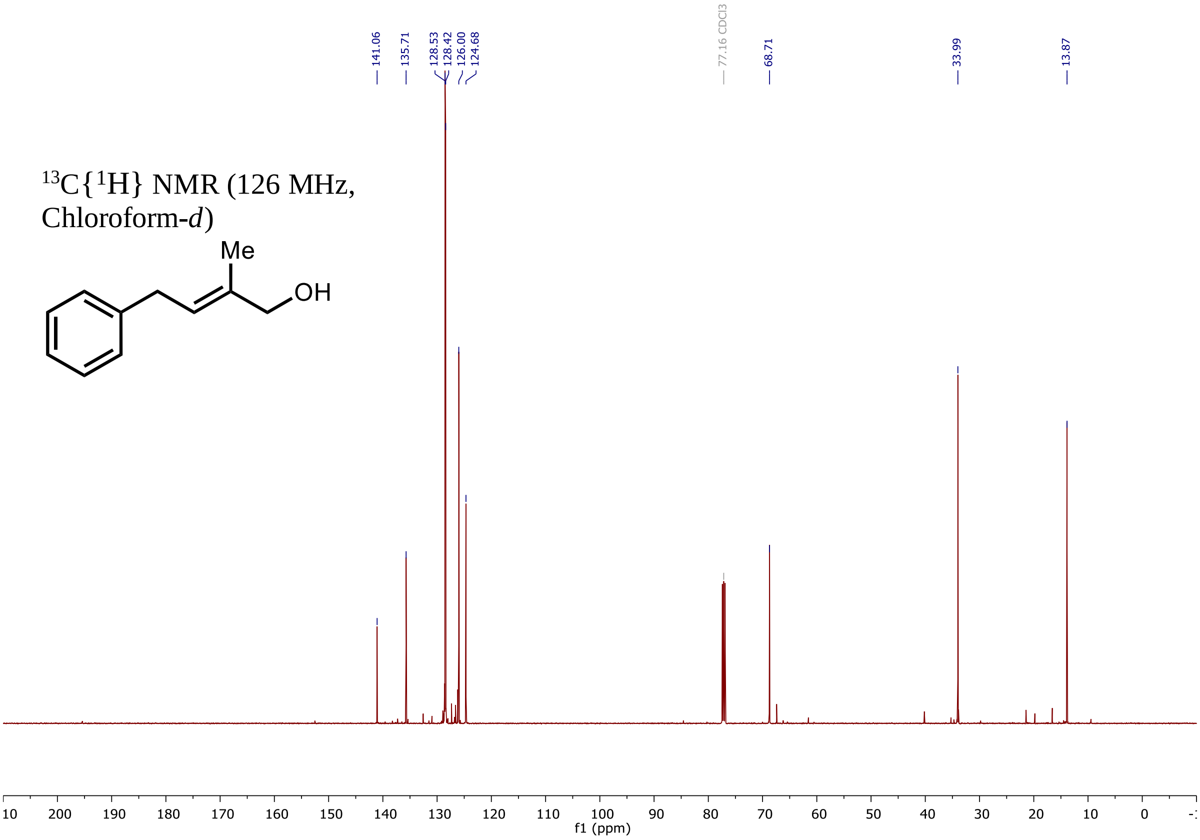
13C{1H} NMR (126 MHz, Chloroform-d)
Me
OH
141.06
135.71
128.53 128.42 126.00 124.68
77.16 CDCl3
68.71
33.99
13.87
f1 (ppm)

(*E*)-3-phenylbut-2-en-1-ol

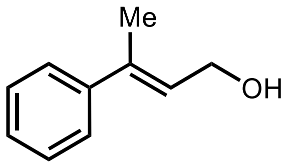

The title compound was prepared according to general procedure 4 using ethyl (E)-3-phenylbut-2-enoate (1.4 g, 7.368 mmol). Purification by flash silica chromatography (eluent = 25% EtOAc in petroleum ether, 35 × 160 mm silica) gave the title compound as a colourless oil (0.334 g, 30%); R_f = 0.31 (eluent = 25% EtOAc in petroleum ether); ν_{max} / cm^{-1} (film) 3369, 3055, 2980, 2873, 1598, 1492, 1468, 1444, 1377, 1265, 1145, 756, 696, 630, 588, 453, 437, 408, 401; ^{1}H NMR (300 MHz, Chloroform-*d*) δ 7.46 – 7.43 (m, 2H), 7.39 – 7.27 (m, 3H), 6.01 (t, *J* = 7.0 Hz, 1H), 4.39 (d, *J* = 6.7 Hz, 2H), 2.11 (s, 4H); ^{13}C{^{1}H} NMR (126 MHz, Chloroform-*d*) δ 143.0, 138.0, 133.3, 128.7, 128.4, 127.4, 126.6, 125.9, 60.1, 16.1; HRMS (ES-TOF) $(M - H)^+$ Calcd for $C_{10}H_{11}O$ 147.0810; Found 147.0812.

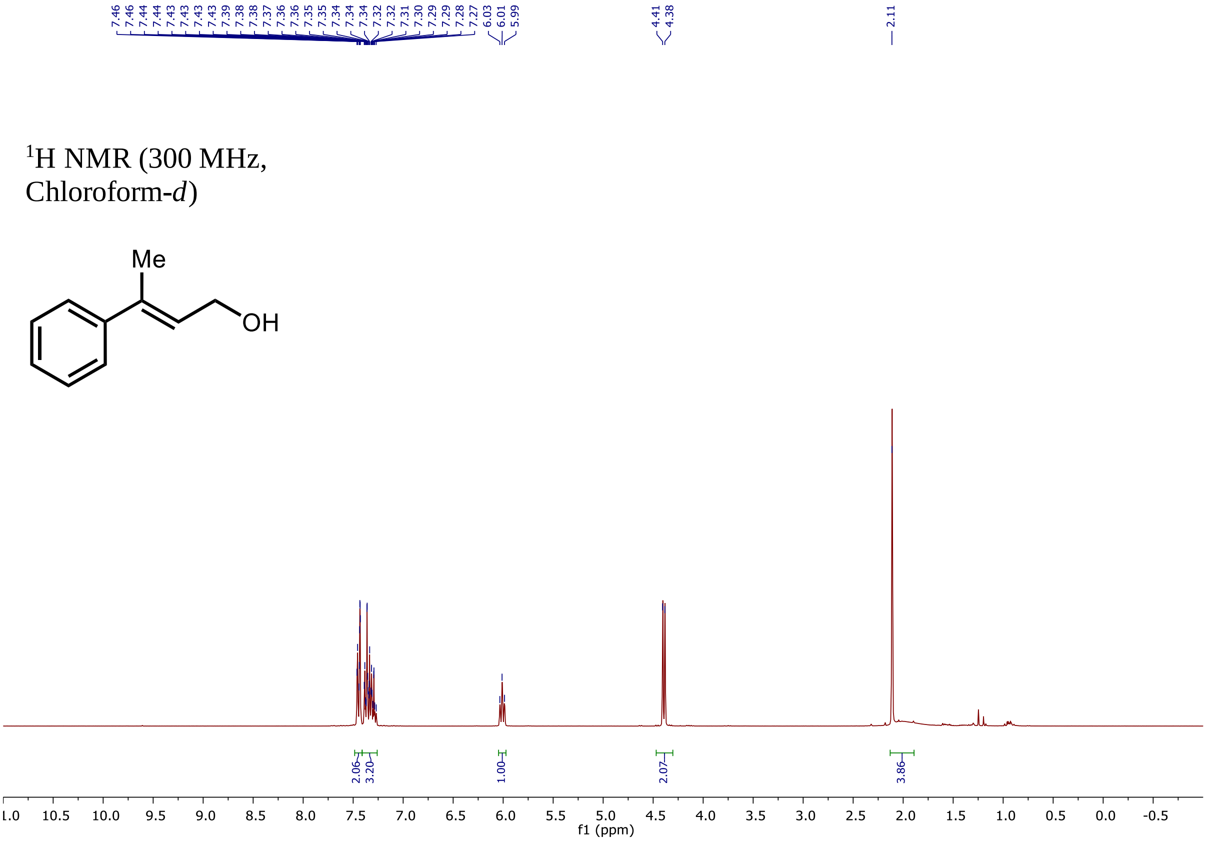

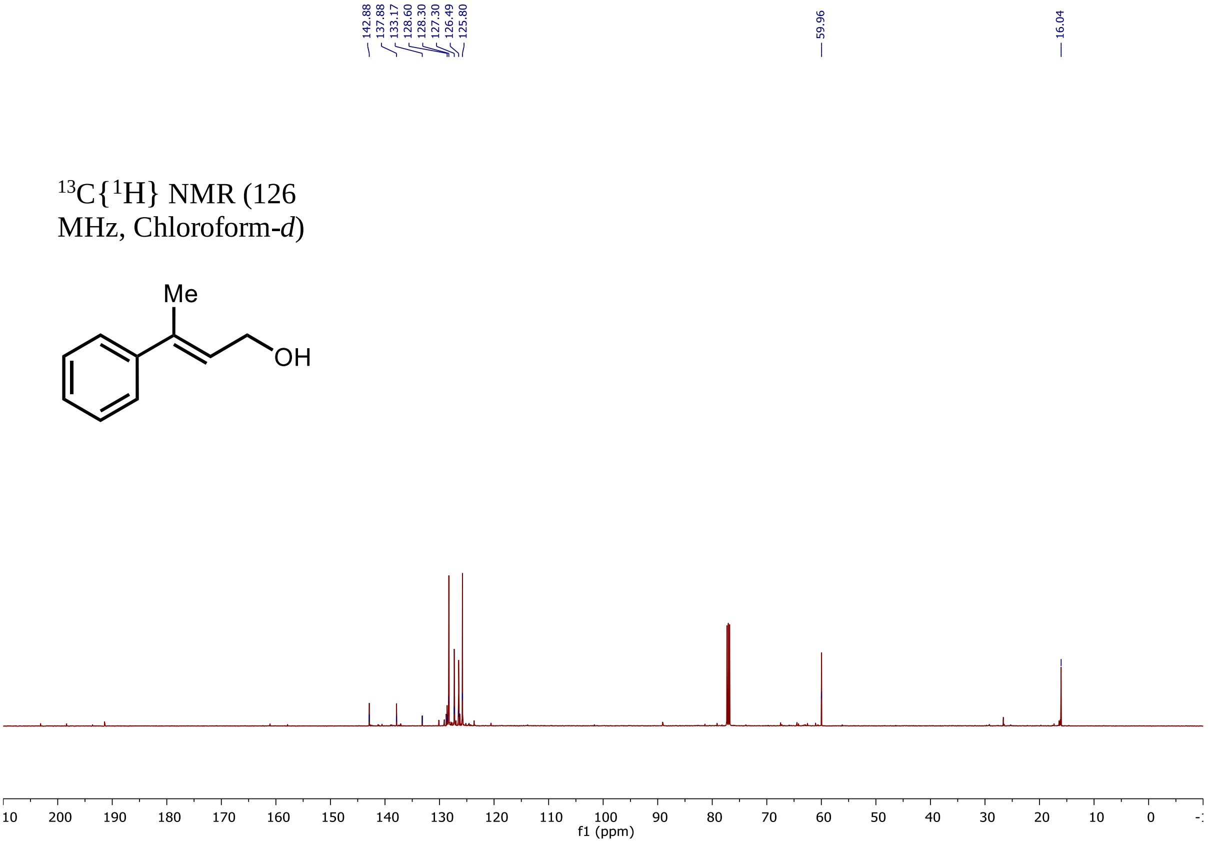

(*E*)-3-phenylprop-2-en-1-ol

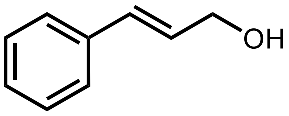

The title compound was prepared according to general procedure 4 using ethyl cinnamate (3 g, 17 mmol). Purification by flash silica chromatography (eluent = 30% EtOAc in petroleum ether, 35 × 160 mm silica) gave the title compound as a low melting point solid (835 mg, 37%); R_f = 0.28 (eluent = 30% EtOAc in petroleum ether); ^{1}H NMR (500 MHz, Chloroform-*d*) δ 7.39 (d, *J* = 7.2 Hz, 1H), 7.32 (t, *J* = 7.6 Hz, 2H), 7.29 – 7.21 (m, 1H), 6.63 (d, *J* = 15.9 Hz, 1H), 6.38 (dt, *J* = 15.9, 5.7 Hz, 1H), 4.33 (dd, *J* = 5.8, 1.6 Hz, 2H), 1.46 (s, 1H); ^{13}C{^{1}H} NMR (126 MHz, Chloroform-*d*) δ 136.8, 131.3, 128.7, 128.6, 127.9, 126.6, 63.9. Spectroscopic data in accordance with that stated in the literature.[9]

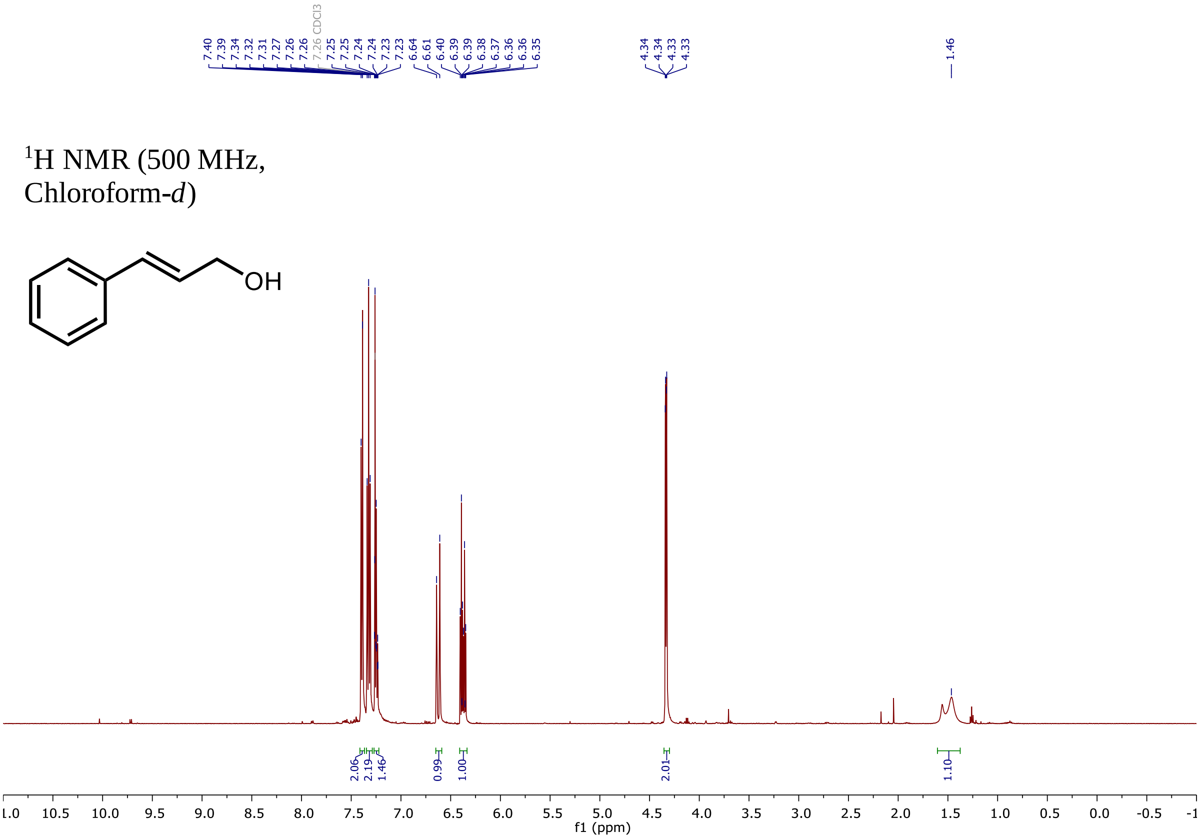

7.40
7.39
7.34
7.32
7.31
7.27
7.26
7.26 CDCl3
7.25
7.25
7.24
7.24
7.23
7.23
6.64
6.61
6.40
6.39
6.39
6.38
6.37
6.36
6.36
6.35
4.34
4.34
4.33
4.33
1.46
1H NMR (500 MHz, Chloroform-d)
OH
2.06
2.19
1.46
0.99
1.00
2.01
1.10
f1 (ppm)

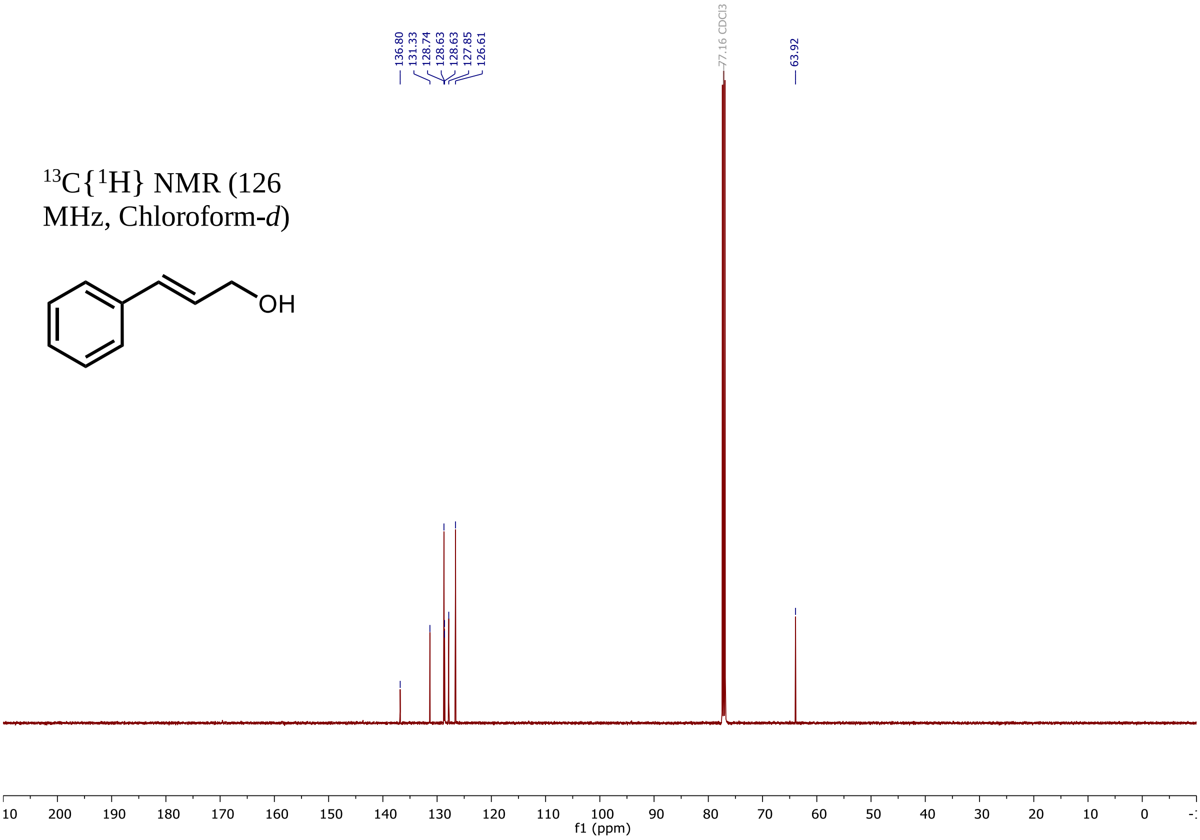

136.80
131.33
128.74
128.63
128.63
127.85
126.61
77.16 CDCl3
63.92
13C{1H} NMR (126 MHz, Chloroform-d)
OH
f1 (ppm)

(*E*)-3-(4-methoxyphenyl)prop-2-en-1-ol

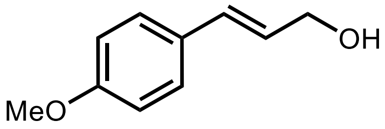

The title compound was prepared according to general procedure 4 using ethyl (*E*)-3-(4-methoxyphenyl)acrylate (2.0 g, 9.7 mmol). Purification by flash silica chromatography (eluent = 10-30% EtOAc in petroleum ether) gave the title compound as a white solid (1.79 g, 79 % yield); mp 60-62 °C; R_f = 0.11 (eluent = 20% EtOAc in petroleum ether); vmax /cm^{-1} (film) 3352, 2906, 2841, 1508, 1604, 1458, 1442, 1224, 1172, 1023, 1024, 1004, 885, 775, 518; ^{1}H NMR (500 MHz, Chloroform-*d*) δ 7.33 (d, *J* = 8.7 Hz, 2H), 6.86 (d, *J* = 8.8 Hz, 2H), 6.56 (d, *J* = 15.9 Hz, 1H), 6.24 (dt, *J* = 15.8, 6.0 Hz, 1H), 4.30 (dd, *J* = 6.0, 1.4 Hz, 2H), 3.81 (s, 3H), 1.49 (s, 1H); ^{13}C{^{1}H} NMR (126 MHz, Chloroform-*d*) δ 159.5, 131.1, 129.6, 127.8, 126.3, 114.2, 64.2, 55.3; HRMS (EI-quadrupole) (M)$^+$ Calcd for $C_{10}H_{12}O_2$ 164.0832; Found 164.0827.

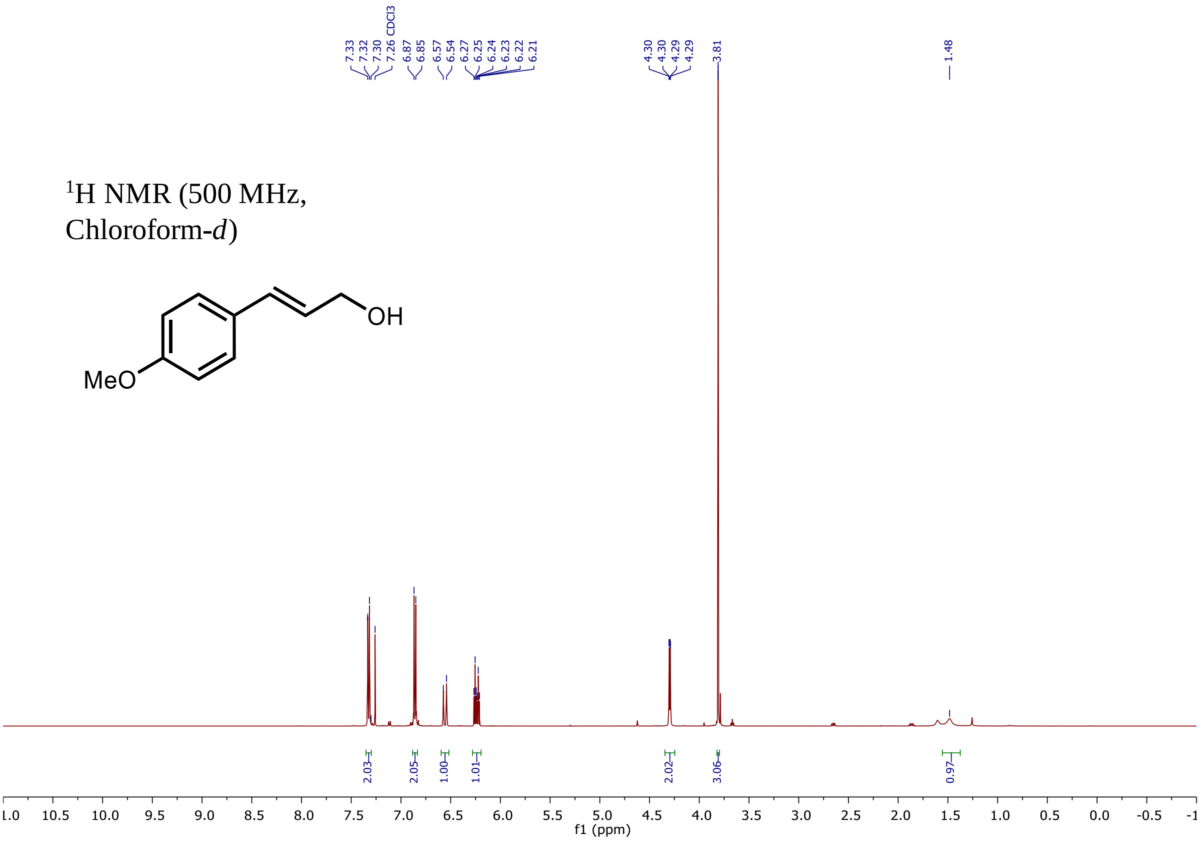

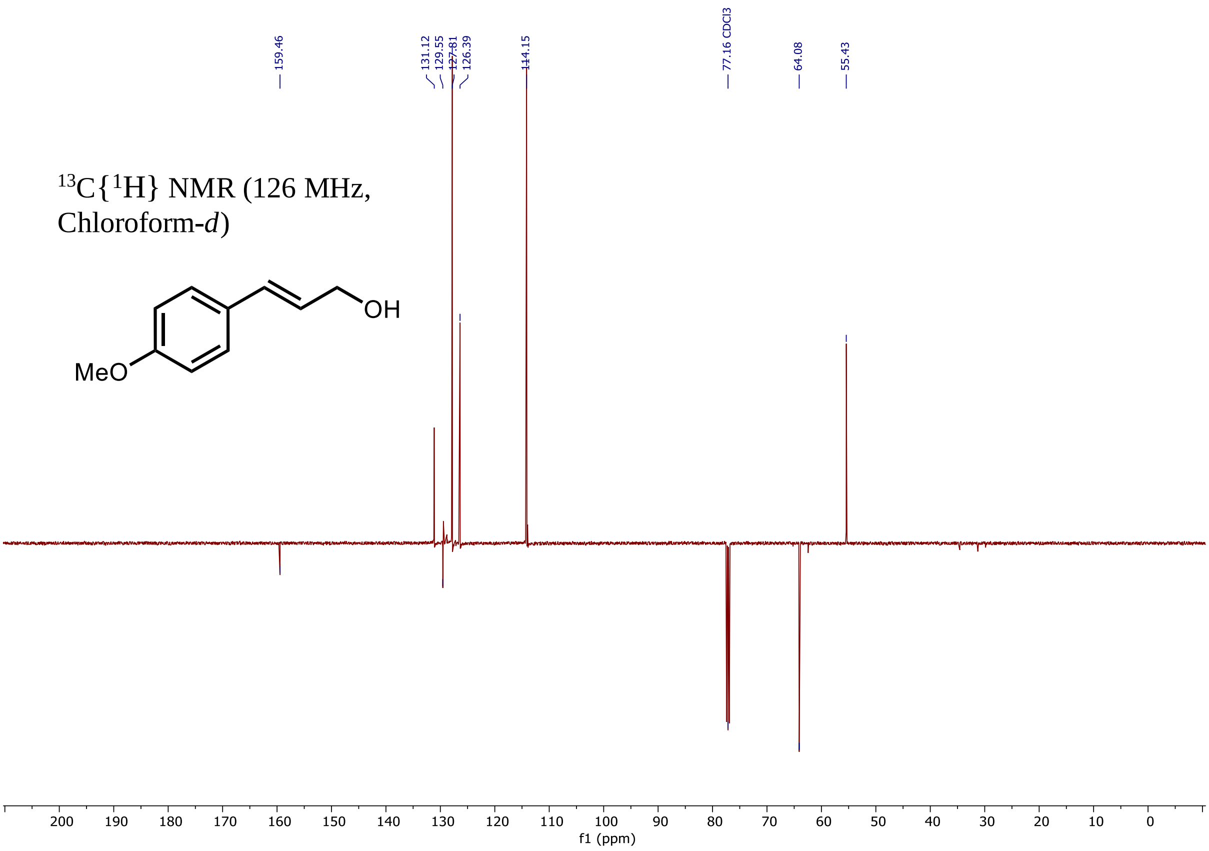

(*E*)-3-(4-fluorophenyl)prop-2-en-1-ol

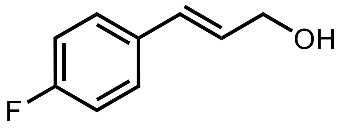

The title compound was prepared according to general procedure 4 using ethyl (*E*)-4-fluorocinnamate (1 g, 5.1 mmol). Purification by flash silica chromatography (eluent = 10-30% EtOAc in petroleum ether, 35 × 160 mm silica) gave the title compound as a white solid (258 mg, 33%); mp 55-57 °C (Lit 54-56 °C);[10] R_f = 0.16 (eluent = 30% EtOAc in petroleum ether); v_{max} / cm^{-1} (film) 3294, 2918, 2851, 1601, 1508, 1231, 1159, 1086, 1007, 966, 845; ^1H NMR (500 MHz, Chloroform-*d*) δ 7.39 – 7.31 (m, 2H), 7.05 – 6.97 (m, 2H), 6.59 (dt, *J* = 15.9, 1.6 Hz, 1H), 6.29 (dtd, *J* = 15.9, 5.8, 0.5 Hz, 1H), 4.32 (d, *J* = 5.2 Hz, 2H), 1.44 (s, 1H); $^{13}C\{^1H\}$ NMR (126 MHz, Chloroform-*d*) δ 162.6 (d, J_{C-F} = 246.9 Hz), 133.1 (d, J_{C-F} = 3.4 Hz), 130.2, 128.4 (d, J_{C-F} = 2.2 Hz), 128.2 (d, J_{C-F} = 8.0 Hz), 115.7 (d, J_{C-F} = 21.6 Hz), 63.9 (d, J_{C-F} = 1.2 Hz); $^{19}F\{^1H\}$ NMR (471 MHz, Chloroform-*d*) δ -114.4; HRMS (CI-quadrupole) $(M)^+$ Calcd for C_9H_9OF 152.0632; Found 152.0630.

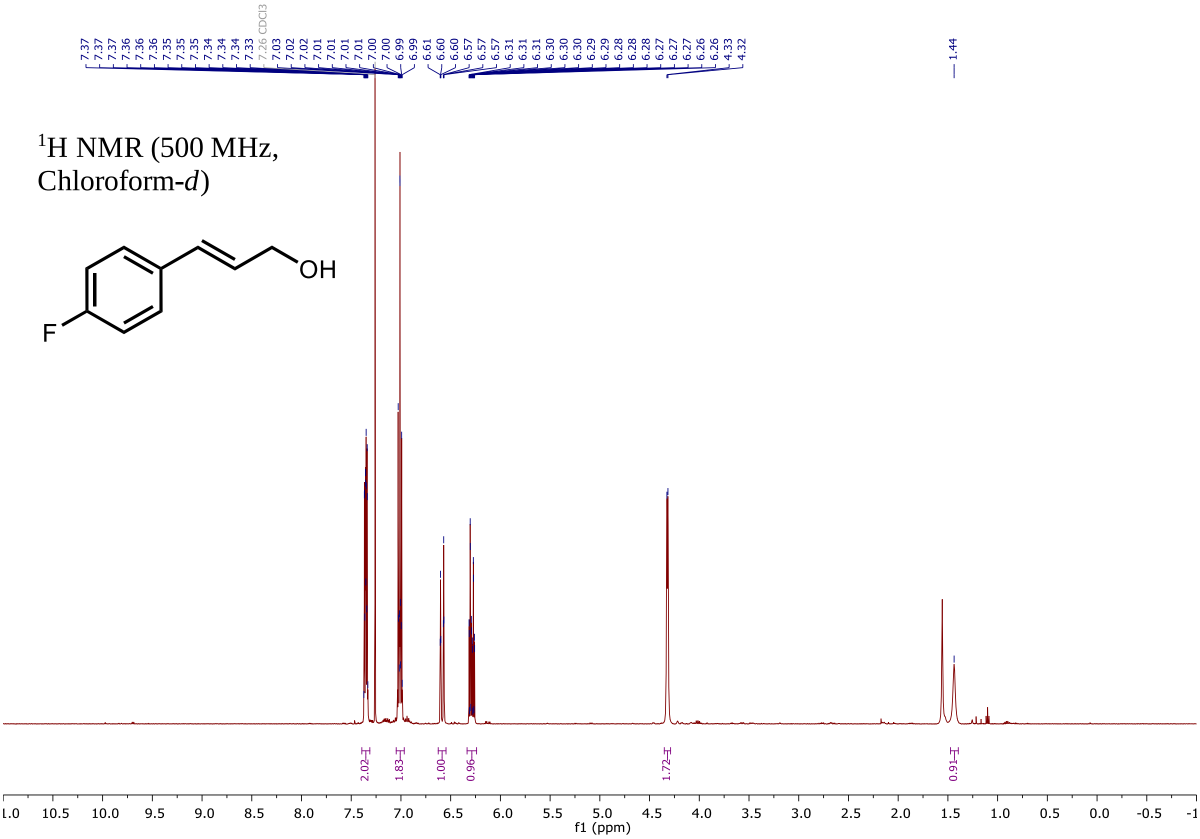

^{1}H NMR (500 MHz, Chloroform-*d*)
OH
F
f1 (ppm)

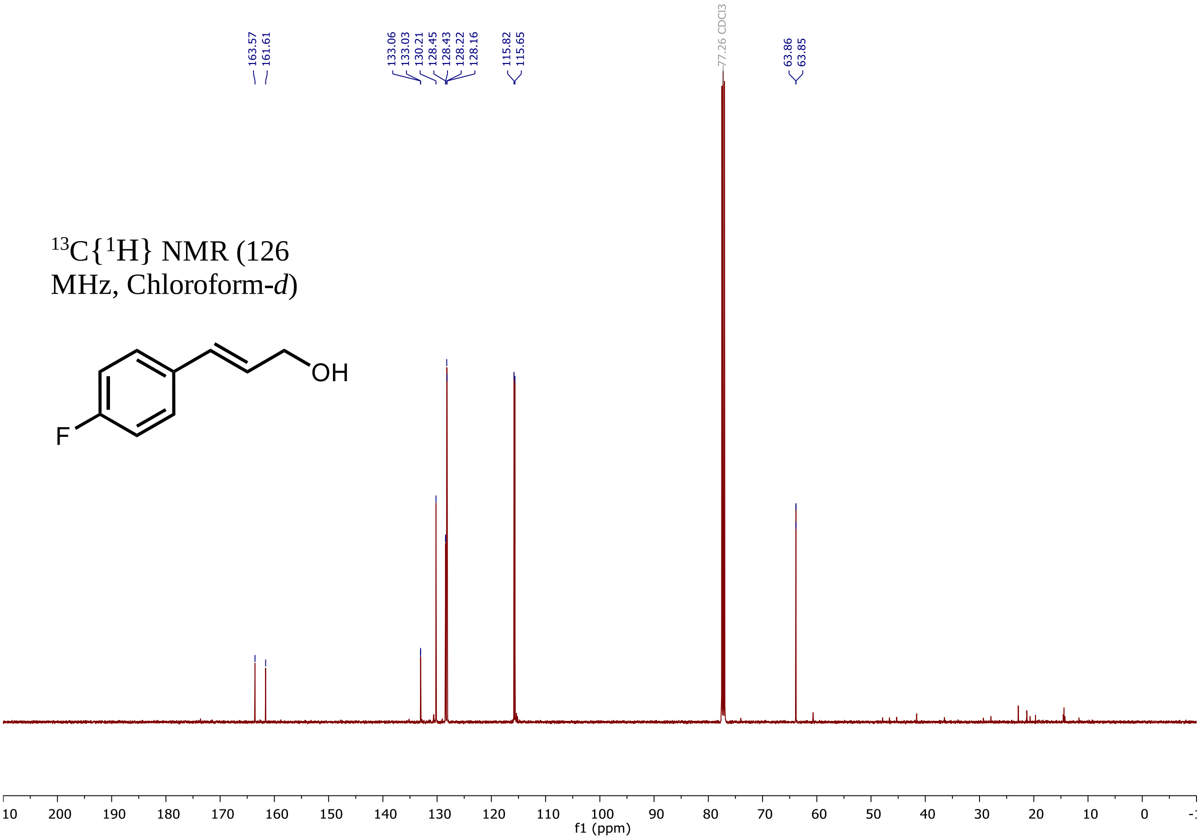

^{13}C{^{1}H} NMR (126 MHz, Chloroform-*d*)
OH
F
f1 (ppm)

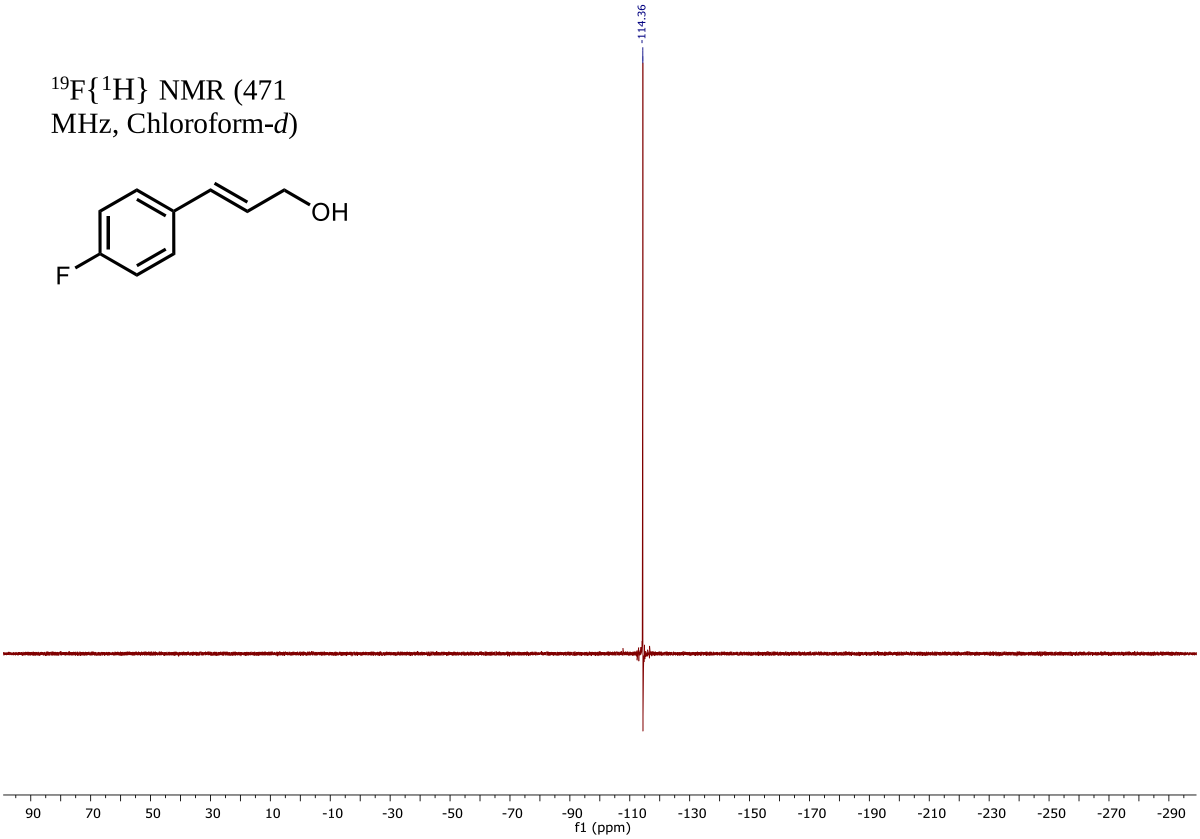

(E)-2-methyl-3-phenylbut-2-en-1-ol

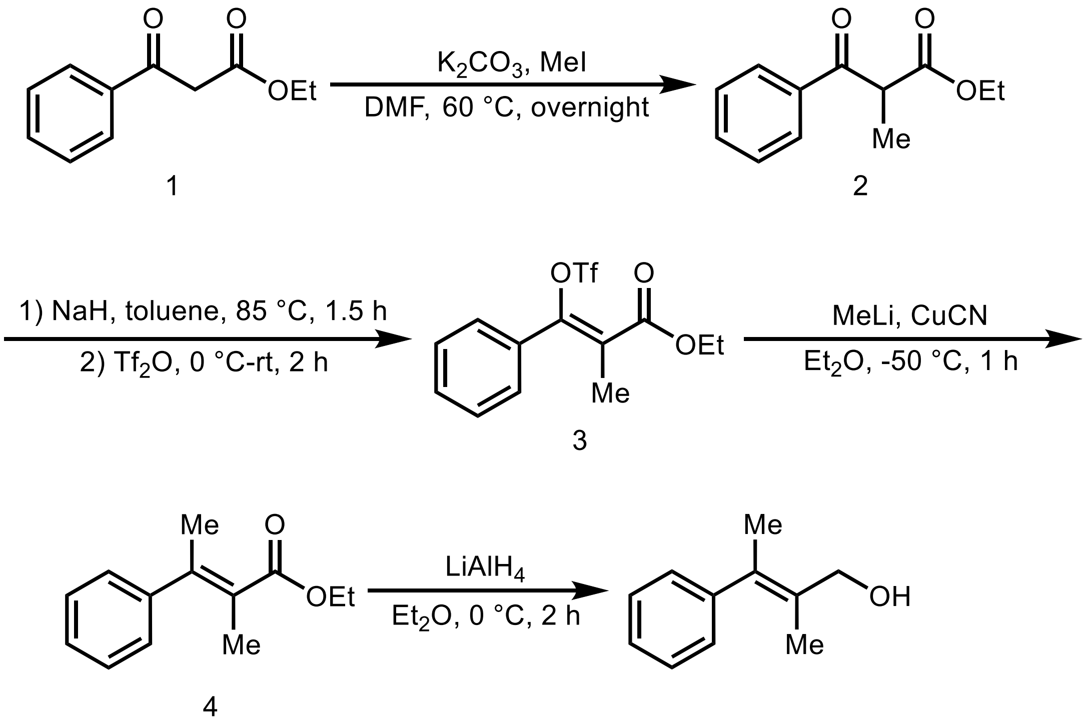

A mixture of ethyl benzoyl acetate (4 g, 21 mmol, 1 equiv.), K_2CO_3 (4.3 g, 31.2 mmol, 1.5 equiv.) and iodomethane (2.14 mL, 34.3 mmol, 1.1 equiv.) in dry DMF (26 mL) was heated at 60 °C overnight. The mixture was then allowed to cool, poured into water and extracted with EtOAc (3×). The combined organic layers were washed with water and brine, dried over

anhydrous $MgSO_4$, filtered and concentrated in vacuo and used in the next step without further purification.

To a stirred suspension of NaH (60% dispersion in mineral oil, 700 mg, 17.5 mmol, 1.8 equiv.) in dry toluene (10 mL) at rt was added **2** (2 g, 9.7 mmol, 1 equiv.). The mixture was stirred at 85 °C for 1.5 h, cooled to 0 °C and Tf_2O (2.45 mL, 14.55 mmol, 1.5 equiv.) was added dropwise. The mixture was stirred at 0 °C for 2 h. The reaction was quenched with water at 0 °C and the aqueous layer was extracted with ethyl acetate (3×). The combined organic layers were washed with water, brine and dried over anhydrous $MgSO_4$, filtered and concentrated in vacuo and used in the next step without further purification.

To a stirred suspension of CuCN (670 mg, 7.5 mmol, 1.4 equiv.) in dry Et_2O (26 mL) at −50 °C was added a solution of methyl lithium (in dry Et_2O, 4.7 mL, 7.5 mmol, 1.4 equiv.) dropwise and the mixture was stirred at −50 °C for 30 min. A solution of **3** (1.7 g, 5 mmol, 1 equiv.) in dry Et_2O (5 mL) was then added at –50 °C and the solution was stirred at −50 °C for another 30 min. The reaction mixture was quenched with a saturated aqueous solution of NH_4Cl and filtered through Celite and the Celite pad was washed with EtOAc. The organic layer was separated and the aqueous layer was further extracted with EtOAc (3×). The combined organic layers were washed with water, brine and dried over anhydrous $MgSO_4$, filtered and concentrated in vacuo and used in the next step without further purification.

The title compound was prepared according to general procedure 4 using ethyl (E)-2-methyl-3-phenylbut-2-enoate (850 mg, 4.16 mmol). Purification by flash silica chromatography (eluent = 20% EtOAc in petroleum ether, 35 × 160 mm silica) gave the title compound as a yellow oil (178 mg, 26%); R_f = 0.15 (eluent = 10% EtOAc in petroleum ether); ^{1}H NMR (500 MHz, Chloroform-*d*) δ 7.37 – 7.29 (m, 2H), 7.27 – 7.19 (m, 1H), 7.17 – 7.10 (m, 2H), 4.31 (s, 2H), 2.04 (dtd, *J* = 2.1, 1.5, 0.5 Hz, 3H), 1.68 (q, *J* = 1.5 Hz, 3H), 1.36 (s, 1H); ^{13}C{^{1}H} NMR (126 MHz, Chloroform-*d*) δ 144.5, 134.5, 130.4, 128.3, 128.1, 126.4, 64.0, 20.5, 18.1. Spectroscopic data in accordance with that stated in the literature.[11]

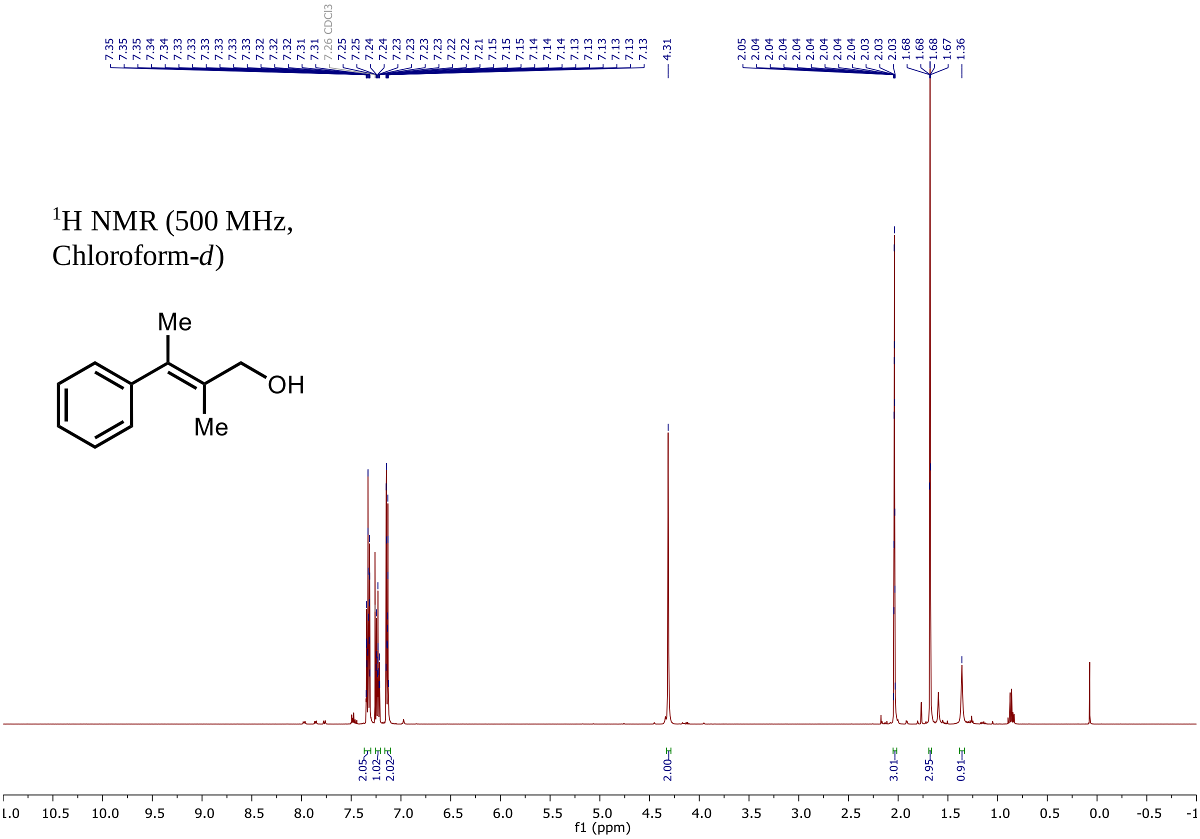
1H NMR (500 MHz, Chloroform-d)
Me
OH
Me
f1 (ppm)

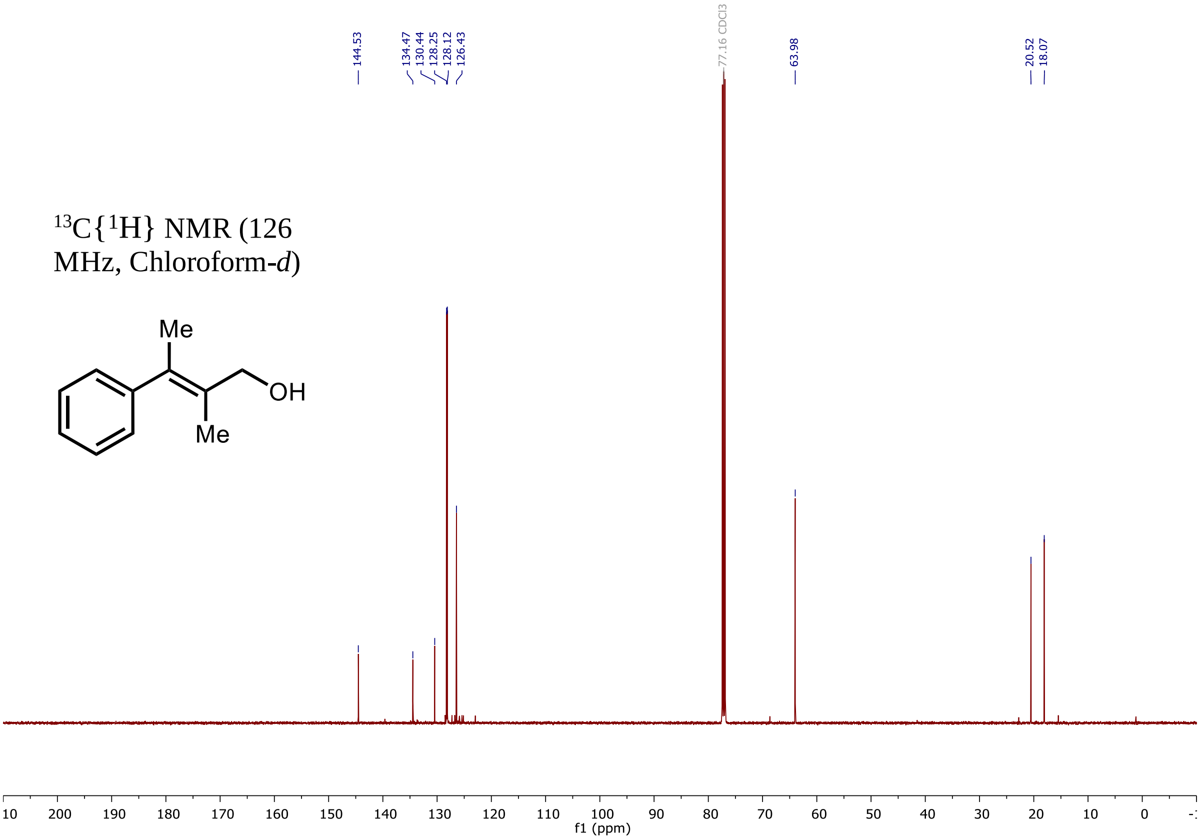
13C{1H} NMR (126 MHz, Chloroform-d)
Me
OH
Me
144.53
134.47
130.44
128.25
128.12
126.43
77.16 CDCl3
63.98
20.52
18.07
f1 (ppm)

2.1.5. General procedure 5

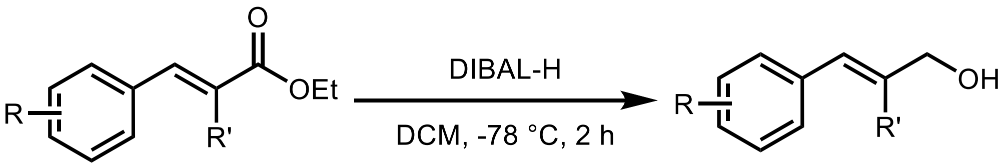

To a stirred solution of the acrylic ester (1 equiv.) in dry DCM (2 M) at -78 °C under N_2 was added DIBAL-H (1 M in toluene, 2.2 equiv.) dropwise over 20 min. The reaction mixture was stirred for 2 h and then quenched with 1 M NaOH at the same temperature. The reaction mixture was allowed to warm to rt and stirred for additional 2 h. The layers were separated and the aqueous layer was extracted with DCM (2×). The combined organics were washed with brine (1×), dried over anhydrous $MgSO_4$, filtered and concentrated in vacuo.

(*E*)-3-(4-(trifluoromethyl)phenyl)prop-2-en-1-ol

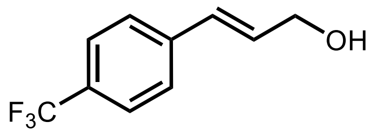

The title compound was prepared according to general procedure 5 using ethyl (*E*)-3-(4-(trifluoromethyl)phenyl)acrylate (570 mg, 2.3 mmol). Purification by flash silica chromatography (eluent = 20-30% EtOAc in petroleum ether, 35 × 160 mm silica) gave the title compound as a white solid (223 mg, 48%); mp 45-47 °C (Lit 49-50 °C);[12] R_f = 0.4 (eluent = 30% EtOAc in petroleum ether); ν_{max} / cm^{-1} (film) 3329, 2930, 2859, 1614, 1414, 1323, 1161, 1067, 1016, 856; ^1H NMR (500 MHz, Chloroform-*d*) δ 7.60 – 7.54 (m, 2H), 7.50 – 7.45 (m, 2H), 6.67 (dt, *J* = 15.9, 1.7 Hz, 1H), 6.46 (dt, *J* = 16.0, 5.4 Hz, 1H), 4.37 (td, *J* = 5.5, 1.6 Hz, 2H), 1.50 (t, *J* = 5.8 Hz, 1H); $^{13}C\{^1H\}$ NMR (126 MHz, Chloroform-*d*) δ 140.3, 131.4, 129.7, 129.5, 126.7, 125.7 (q, J_{C-F} = 3.8 Hz), 124.3 (q, J_{C-F} = 272 Hz), 63.5; $^{19}F\{^1H\}$ NMR (471 MHz, Chloroform-*d*) δ -62.5; HRMS (CI-quadrupole) $(M)^+$ Calcd for $C_{10}H_9OF_3$ 202.0600; Found 202.0599.

^{1}H NMR (500 MHz, Chloroform-*d*)

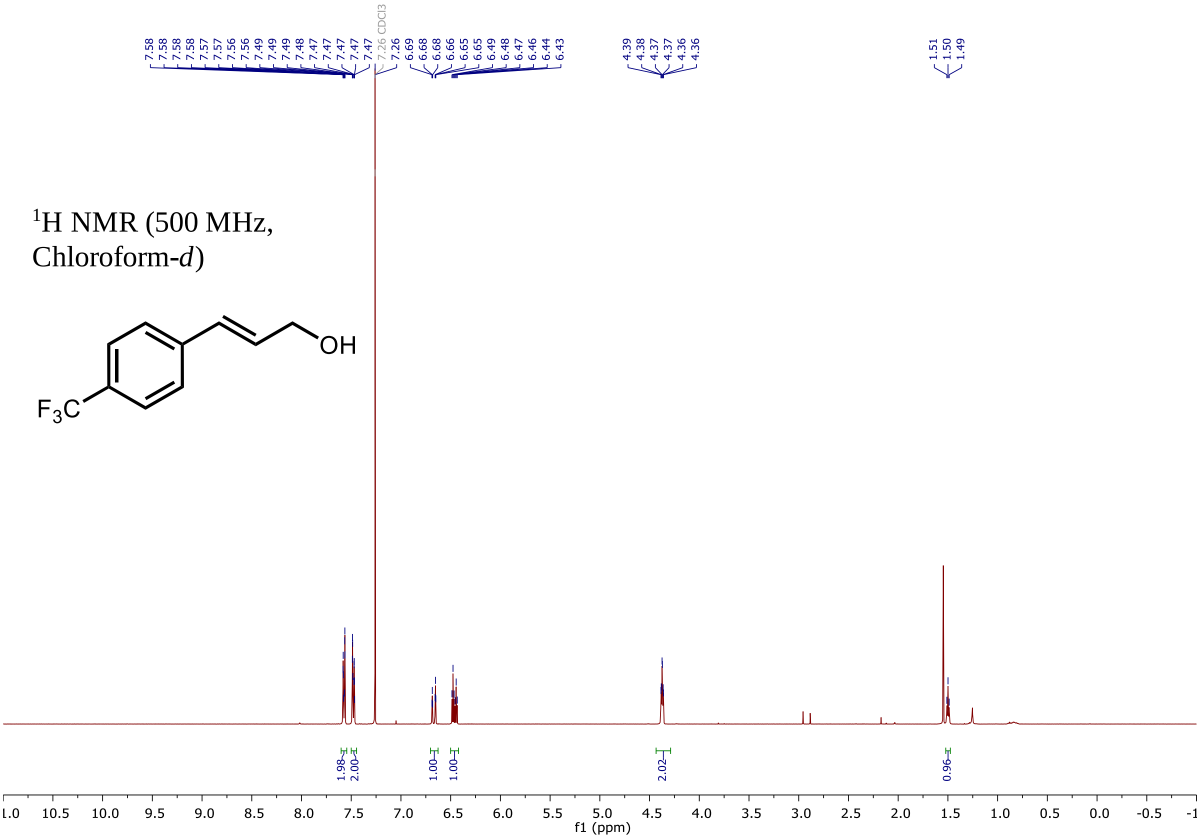

^{13}C{^{1}H} NMR (126 MHz, Chloroform-*d*)

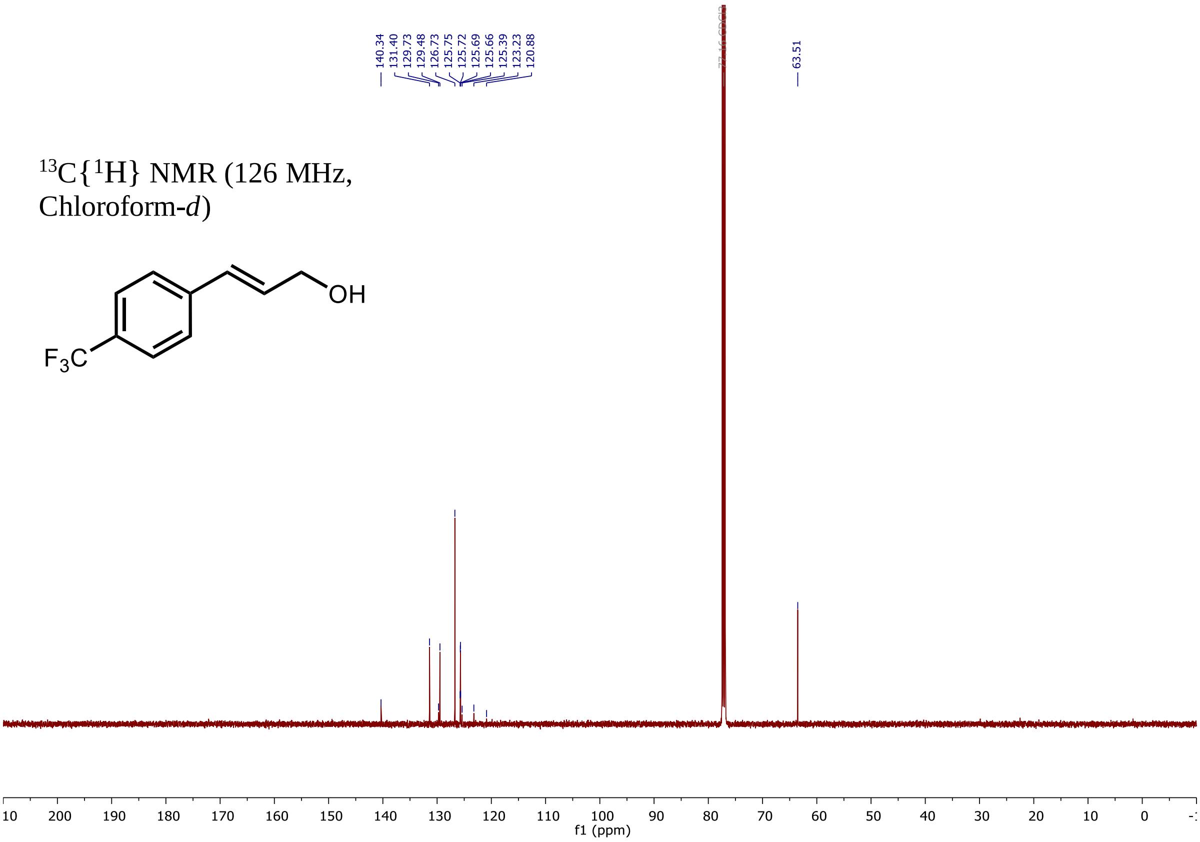

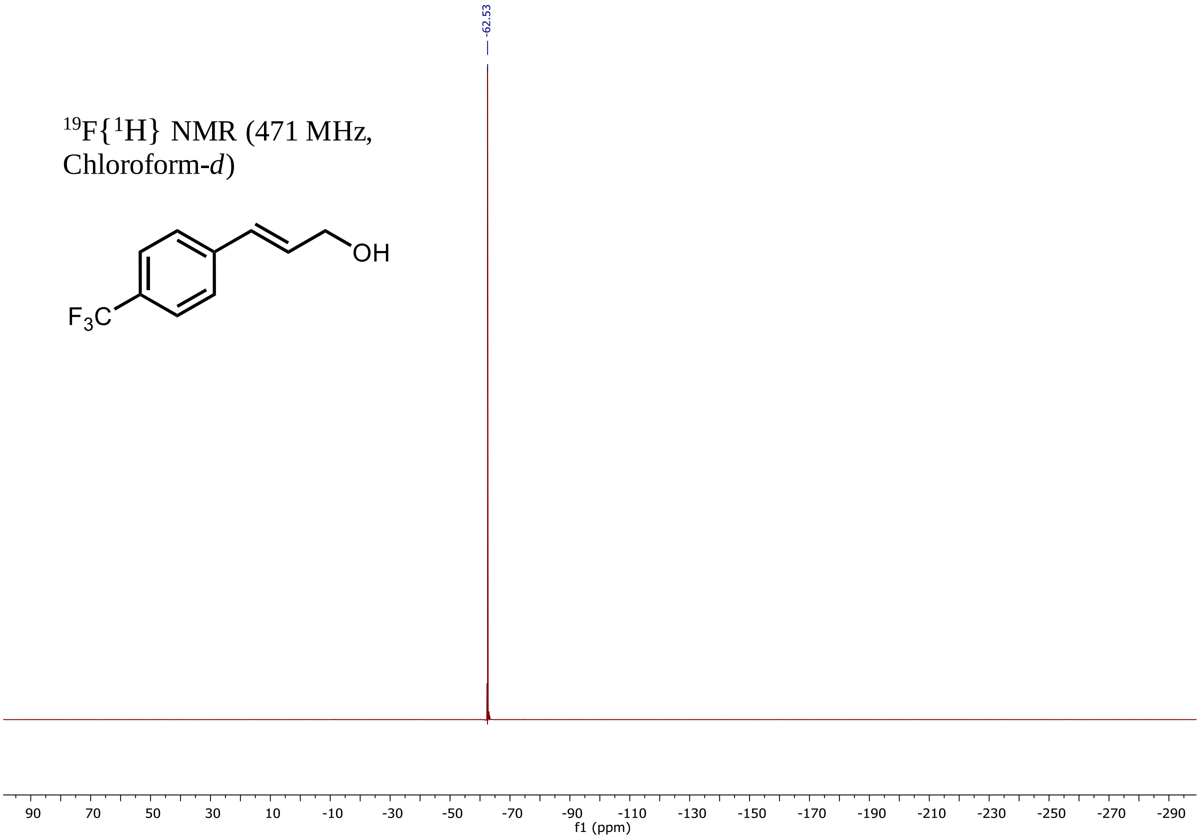

(*E*)-2-methyl-3-(1-methyl-1H-indol-3-yl)prop-2-en-1-ol

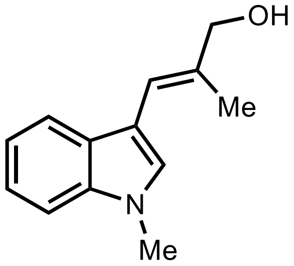

The title compound was prepared according to general procedure 5 using ethyl (*E*)-2-methyl-3-(1-methyl-1H-indol-3-yl)acrylate (900 mg, 3.7 mmol). Upon purification by flash silica chromatography (eluent = 20-30% EtOAc in petroleum ether, 35 × 160 mm silica), the title compound was obtained as a yellow oil. The compound is unstable for storage and degradation was triggered while running NMR sample. However, partial evaporation of EtOAc maintained the compound for analysis and the next step was done directly after separation and concentration; R_f = 0.25 (eluent = 20% EtOAc in petroleum ether); ν_{max} / cm^{-1} (film) 3400, 3049, 2930, 2876, 1533, 1474, 1371, 1331, 1244, 1013, 906, 731; ^{1}H NMR (500 MHz, Chloroform-*d*) δ 7.71 – 7.67 (m, 1H), 7.32 (dt, *J* = 8.2, 1.0 Hz, 1H), 7.30 – 7.23 (m, 1H), 7.16 (ddd, *J* = 8.0, 6.9, 1.1 Hz, 1H), 7.14 (d, *J* = 0.7 Hz, 1H), 6.73 – 6.68 (m, 1H), 4.27 (d, *J* = 1.2 Hz, 2H), 3.81 (s, 3H), 2.00 (d, *J* = 1.3 Hz, 3H), 1.55 (s, 1H); $^{13}C\{^{1}H\}$ NMR (126 MHz,

Chloroform-*d*) δ 136.4, 133.7, 128.1, 127.4, 122.2, 119.5, 119.1, 116.9, 112.3, 109.3, 70.0, 33.0, 16.7; HRMS (APCI-TOF) (M - H)$^+$ Calcd for $C_{13}H_{14}NO$ 200.1075; Found 200.1070.

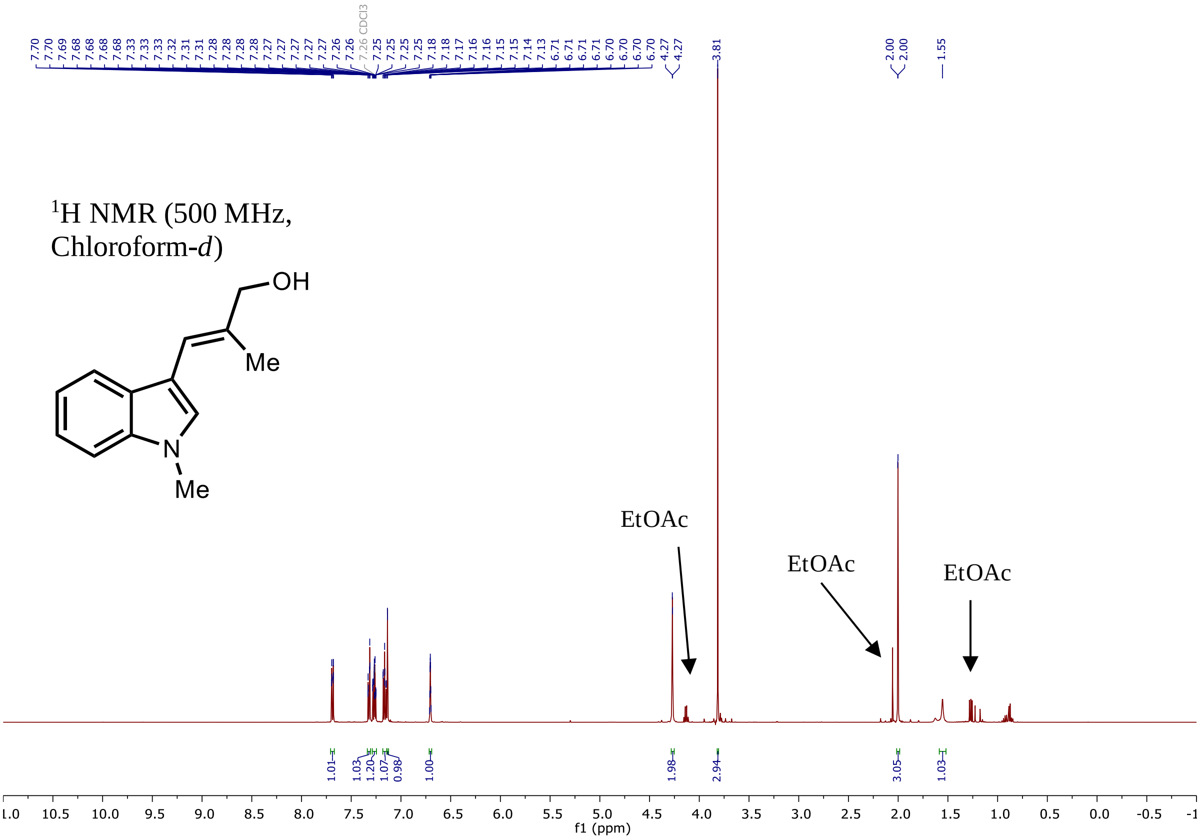

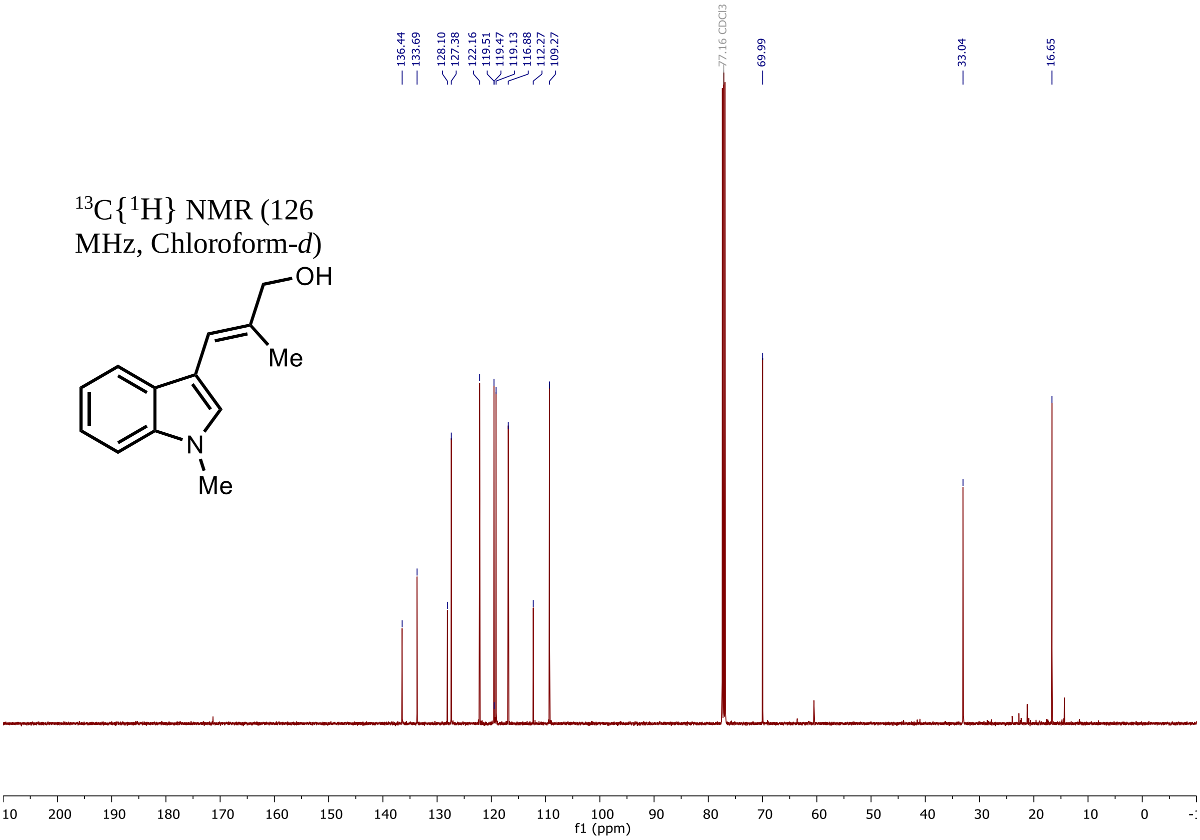

(*E*)-2-methyl-3-(pyridin-4-yl)prop-2-en-1-ol

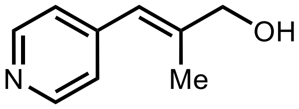

The title compound was prepared according to general procedure 5 using ethyl (*E*)-2-methyl-3-(pyridine-4-yl)acrylate (900 mg, 4.7 mmol). Purification by flash silica chromatography (eluent = 100% EtOAc, 35 × 160 mm silica) gave the title compound as white crystals (437 mg, 62%); mp 101-104 °C; R_f = 0.22 (eluent = 100% EtOAc); ν_{max} / cm^{-1} (film) 3138, 2858, 2818, 1601, 1416, 1371, 1082, 1007, 959, 872, 542; ^1H NMR (500 MHz, Chloroform-*d*) δ 8.53 (d, *J* = 4.5 Hz, 2H), 7.17 (d, *J* = 5.5 Hz, 2H), 6.50 (s, 1H), 4.22 (s, 2H), 2.52 (s, 1H), 1.91 (s, 3H); $^{13}C\{^1H\}$ NMR (126 MHz, Chloroform-*d*) δ 149.7 (d, *J* = 3.1 Hz), 145.6, 142.6, 123.8, 121.9, 68.1, 15.6; HRMS (ES-TOF) $(M + H)^+$ Calcd for $C_9H_{12}NO$ 150.0918; Found 150.0919.

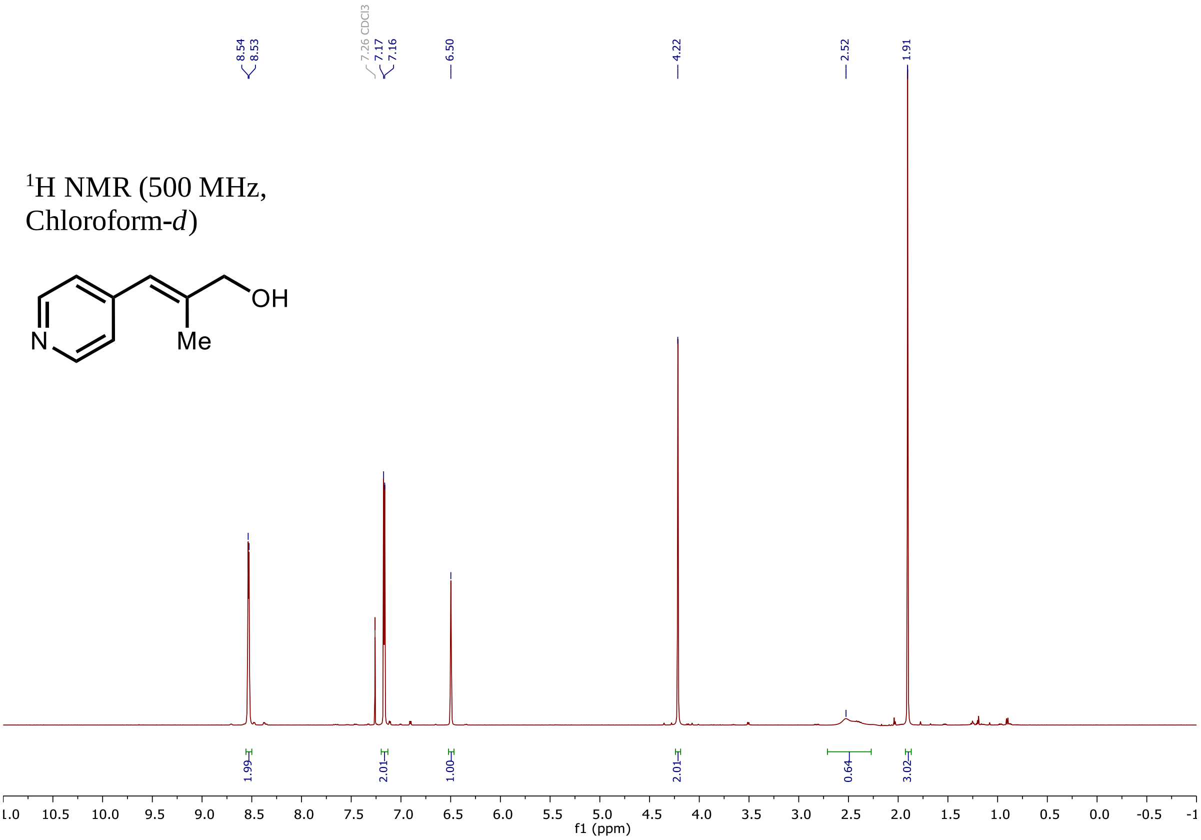

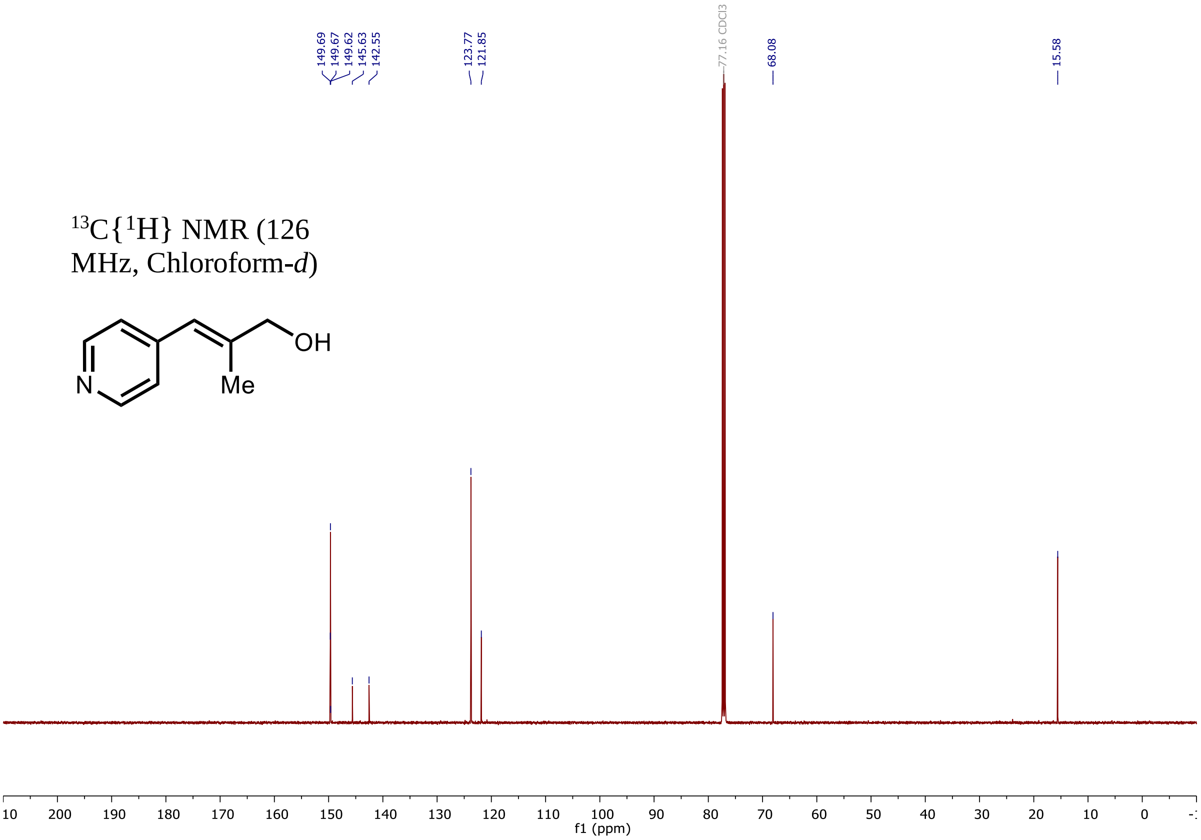

(*E*)-2-methyl-5-phenylpent-2-en-1-ol

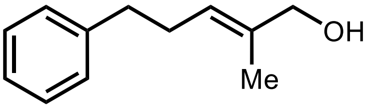

The title compound was prepared according to general procedure 5 using ethyl (*E*)-2-methyl-5-phenylpent-2-enoate (2.0 g, 9.2 mmol). The resulting crude was stirred with bromine water at 0 °C for 20 minutes and transferred to a separatory funnel and then aqueous phase was extracted with EtOAc (2 × 25 mL). Purification by flash silica chromatography (eluent = 20-40% EtOAc in petroleum ether) gave the title compound as a clear liquid (110 mg, 61% yield); R_f = 0..25 (eluent = 20% EtOAc in petroleum ether); vmax /cm^{-1} (film) 3298, 2918, 2856, 1494, 1452, 1001, 696; ^{1}H NMR (500 MHz, Chloroform-*d*) δ 7.33 – 7.28 (m, 2H), 7.24 – 7.18 (m, 3H), 5.48 (tq, *J* = 7.1, 1.3 Hz, 1H), 3.99 (s, 2H), 2.74 – 2.67 (m, 2H), 2.39 (q, *J* = 7.5 Hz, 2H), 1.72 (s, 1H), 1.64 (s, 3H);^{13}C{^{1}H} NMR (126 MHz, Chloroform-*d*) δ 142.1, 135.6, 128.5, 128.4, 125.9, 125.2, 68.8, 35.8, 29.6, 13.7; HRMS (EI-quadrupole) (M - H)$^+$ Calcd for $C_{12}H_{15}O$ 175.1117; Found 175.1114.

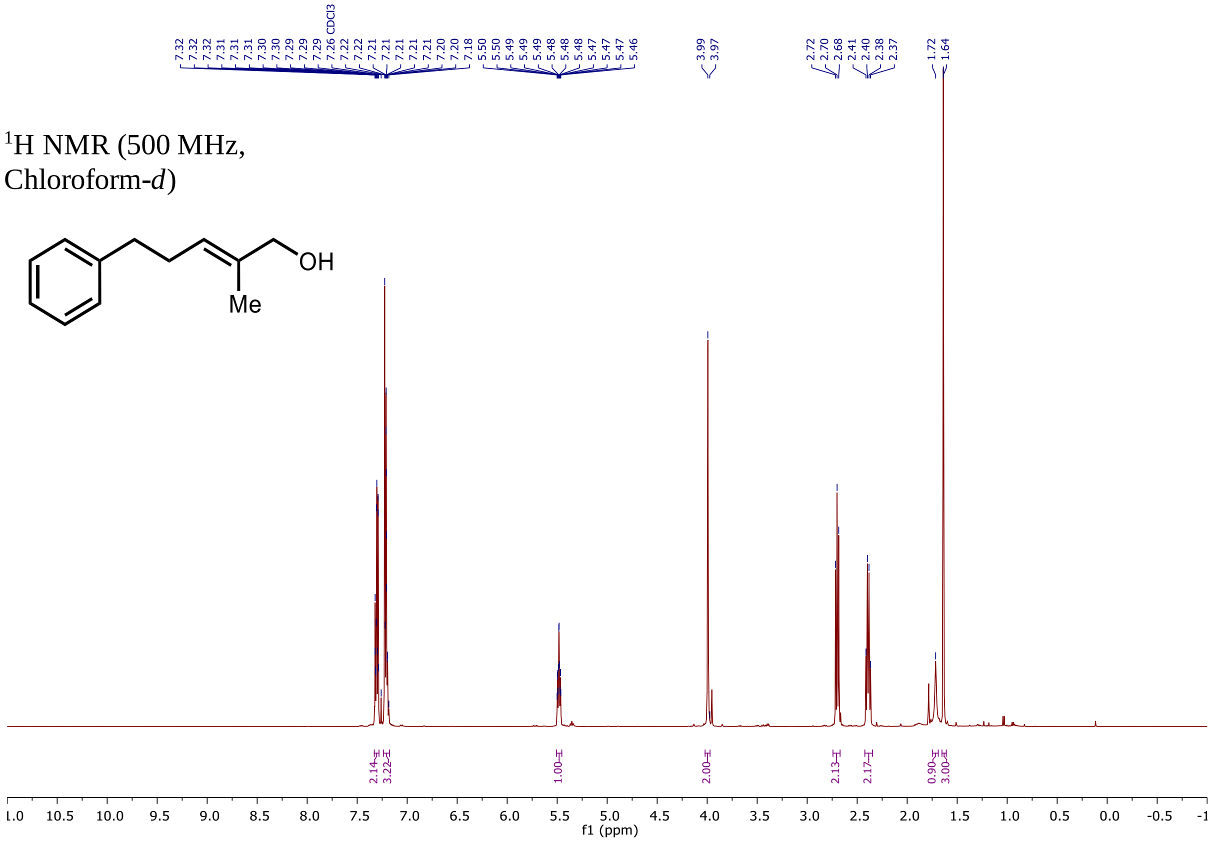

1H NMR (500 MHz, Chloroform-d)
OH
Me
7.32 7.32 7.32 7.31 7.31 7.30 7.30 7.29 7.29 7.29 7.26 CDCl3 7.22 7.22 7.21 7.21 7.21 7.21 7.21 7.20 7.18 5.50 5.50 5.49 5.49 5.49 5.48 5.48 5.48 5.47 5.47 5.47 5.46 3.99 3.97 2.72 2.70 2.68 2.41 2.40 2.38 2.37 1.72 1.64
2.14 3.22 1.00 2.00 2.13 2.17 0.90 3.00
f1 (ppm)

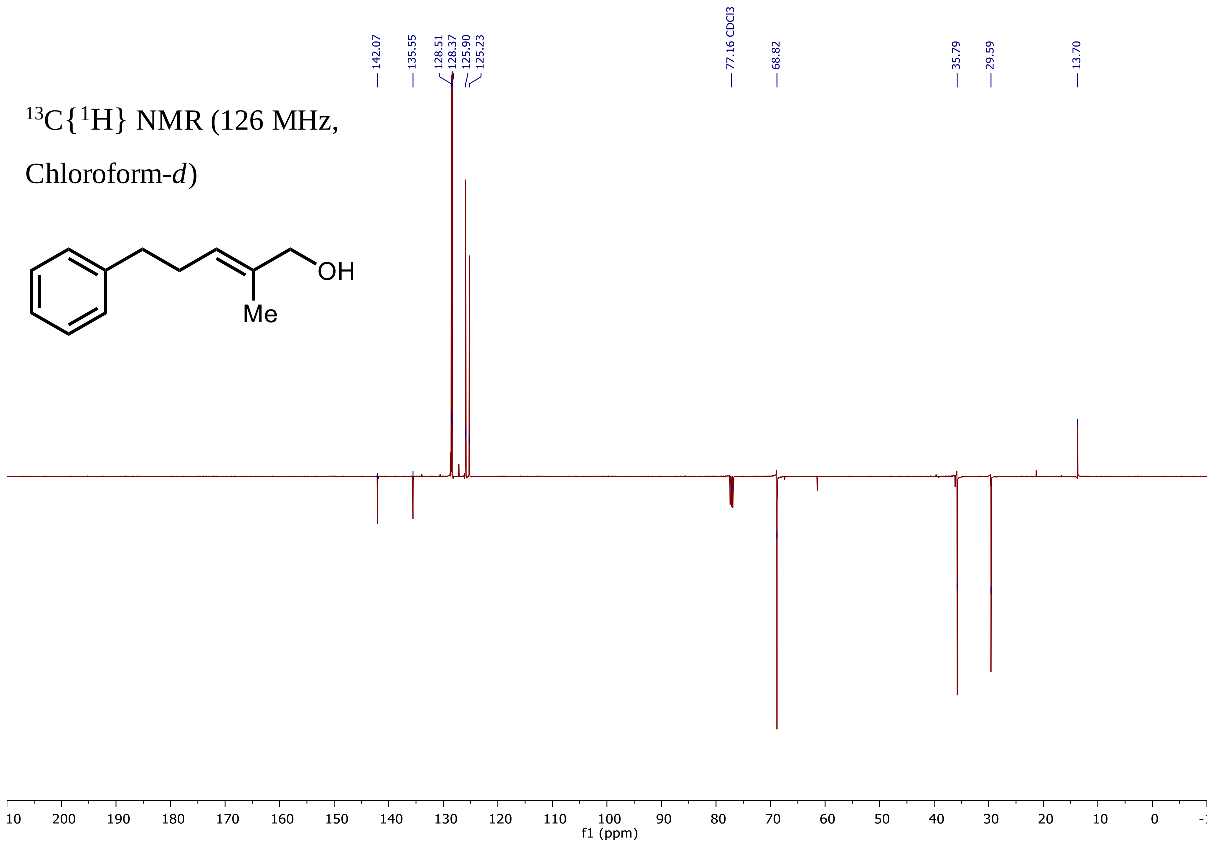

13C{1H} NMR (126 MHz, Chloroform-d)
OH
Me
142.07 135.55 128.51 128.37 125.90 125.23 77.16 CDCl3 68.82 35.79 29.59 13.70
f1 (ppm)

(*E*)-3-cyclohexyl-2-methylprop-2-en-1-ol

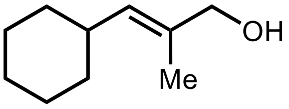

The title compound was prepared according to a general procedure 5 using ethyl (*E*)-3-cyclohexyl-2-methylacrylate (3.7 g, 18.8 mmol). Purification by flash silica chromatography (eluent = 5% EtOAc in petroleum ether, 35 × 260 mm silica) gave the mixture of title compound and Z isomer as a clear oil (1.4 g, 48%); R_f = 0.25 (eluent = 10 % EtOA in petroleum ether), v_{max} / cm^{-1} (film) 3292, 2920, 2848, 1446, 1004, 893; ^1H NMR (500 MHz, Chloroform-*d*) δ 5.23 (dd, *J* = 9.1, 1.4 Hz, 1H), 5.14 (dd, *J* = 9.4, 1.6 Hz, 0.15H (Z isomer)), 4.12 (s, 0.32H (Z isomer)), 3.97 (d, *J* = 1.3 Hz, 2H), 2.26 – 2.14 (m, 1H), 1.77 (d, *J* = 1.5 Hz, 0.46H (Z isomer)), 1.70 (ddd, *J* = 12.8, 5.5, 2.2 Hz, 2H), 1.67 (d, *J* = 1.4 Hz, 3H), 1.64 – 1.58 (m, 3H), 1.35 (s, 1H), 1.27 (tt, *J* = 12.4, 3.3 Hz, 2H), 1.18 (tt, *J* = 12.5, 3.2 Hz, 1H), 1.09 – 1.00 (m, 2H); $^{13}C\{^1H\}$ NMR (126 MHz, Chloroform-*d*) δ 135.3 (Z isomer), 133.0, 132.6, 69.2, 62.1 (Z isomer) 36.8, 33.2, 33.9 (Z isomer), 26.2, 26.1, 26.1 (Z isomer), 26.0 (Z isomer), 21.4 (Z isomer), 13.9; HRMS (EI-quadrupole) $(M)^+$ Calcd for $C_{10}H_{18}O$ 154.1352; Found 154.1349.

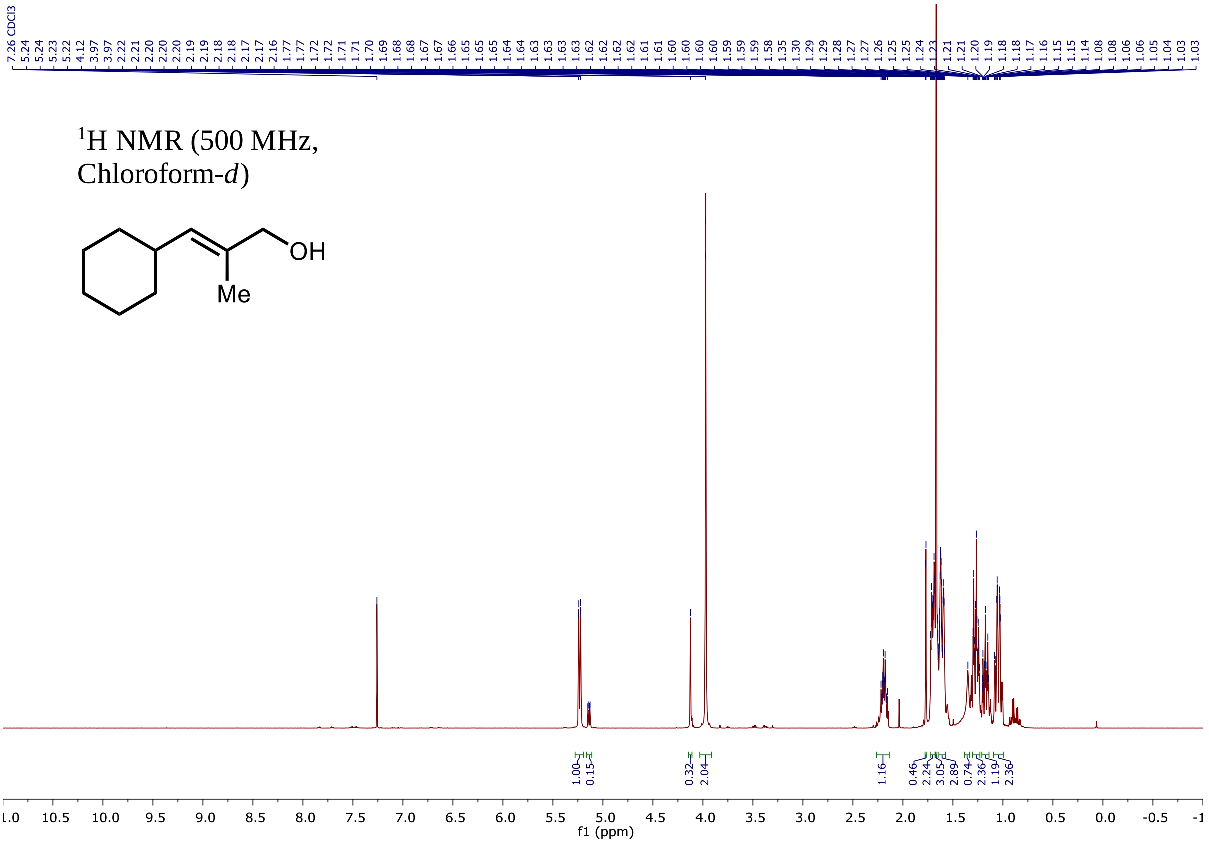

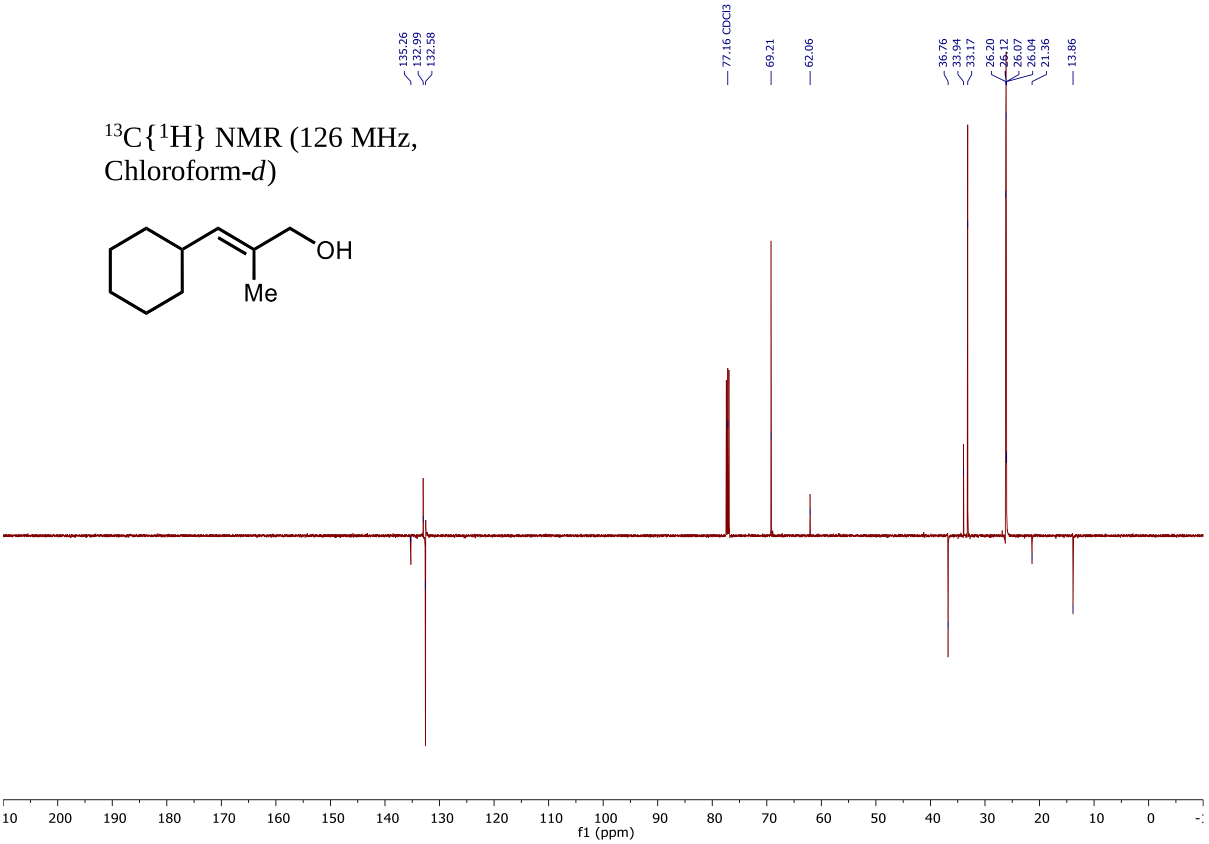

2.1.6. General Procedure 6

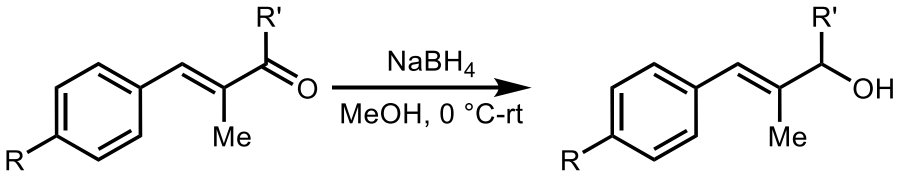

To a solution of the carbonyl compound (1 equiv.) in dry MeOH (1.7 mL/mmol), $NaBH_4$ (1.3 equiv.) was added at 0 °C, stirred at rt and monitored with TLC. After completion, the solvent was removed and the residue was dissolved in H_2O, extracted with DCM (2×) and the combined organic layers were washed with brine (1×), dried over anhydrous $MgSO_4$, filtered and concentrated in vacuo.

Methyl (*E*)-4-(3-hydroxy-2-methylprop-1-en-1-yl)benzoate

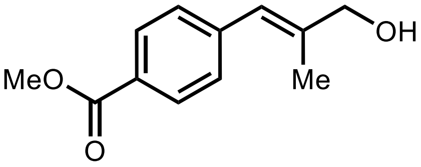

The title compound was prepared according to general procedure 6 using methyl (*E*)-4-(2-methyl-3-oxoprop-1-en-1-yl)benzoate (260 mg, 1.35 mmol). After workup, the title compound was obtained as a white solid (165 mg, 63%); mp 46-49 °C; R_f = 0.41 (eluent = 30% EtOAc in

petroleum ether); ν_{max} / cm^{-1} (film) 3406, 2953, 1720, 1605, 1440, 1280, 1183, 1107, 1018, 761; ^{1}H NMR (500 MHz, Chloroform-*d*) δ 8.04 – 7.97 (m, 2H), 7.37 – 7.31 (m, 2H), 6.57 (d, *J* = 2.1 Hz, 1H), 4.22 (d, *J* = 5.6 Hz, 2H), 3.92 (s, 3H), 1.91 (d, *J* = 0.8 Hz, 3H), 1.59 – 1.55 (m, 1H); $^{13}C\{^{1}H\}$ NMR (126 MHz, Chloroform-*d*) δ 167.1, 142.5, 140.1, 129.6, 128.9, 128.1, 124.1, 68.8, 52.2, 15.6; HRMS (CI-quadrupole) $(M + H)^{+}$ Calcd for $C_{12}H_{15}O_3$ 207.1016; Found 207.1015.

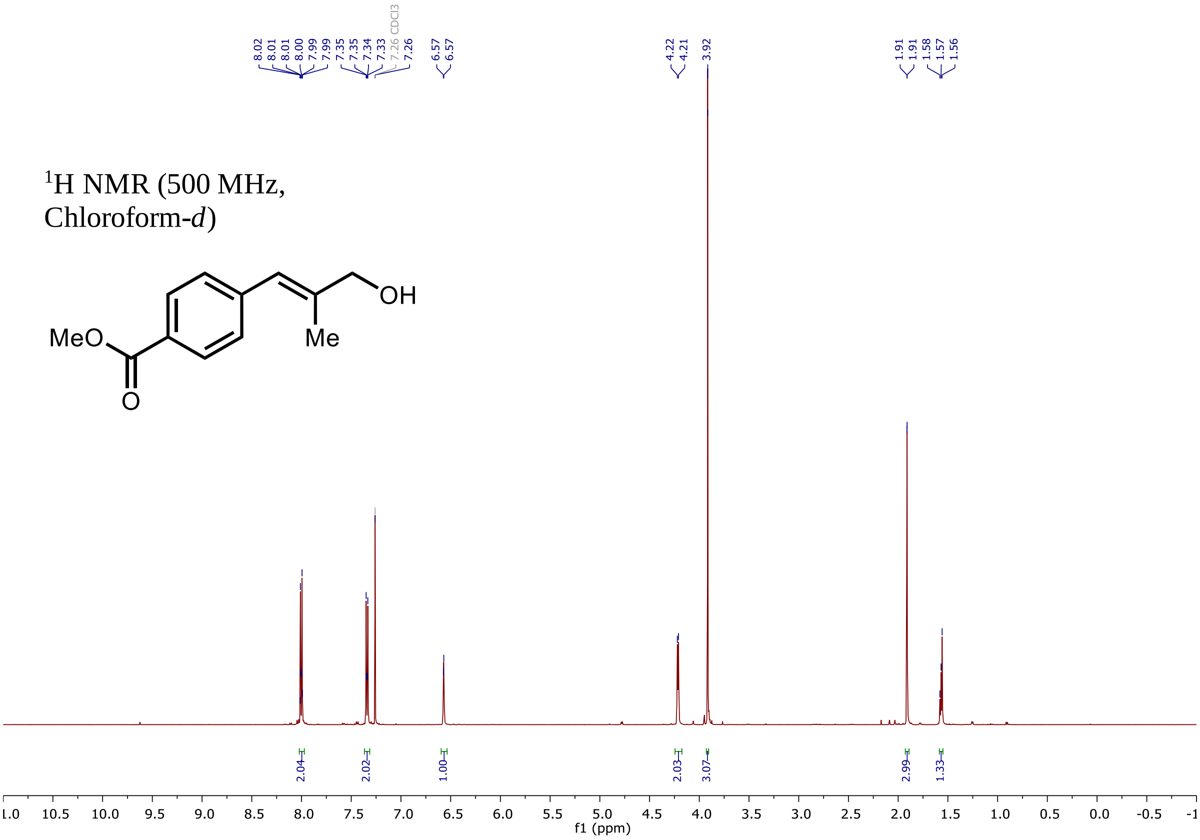

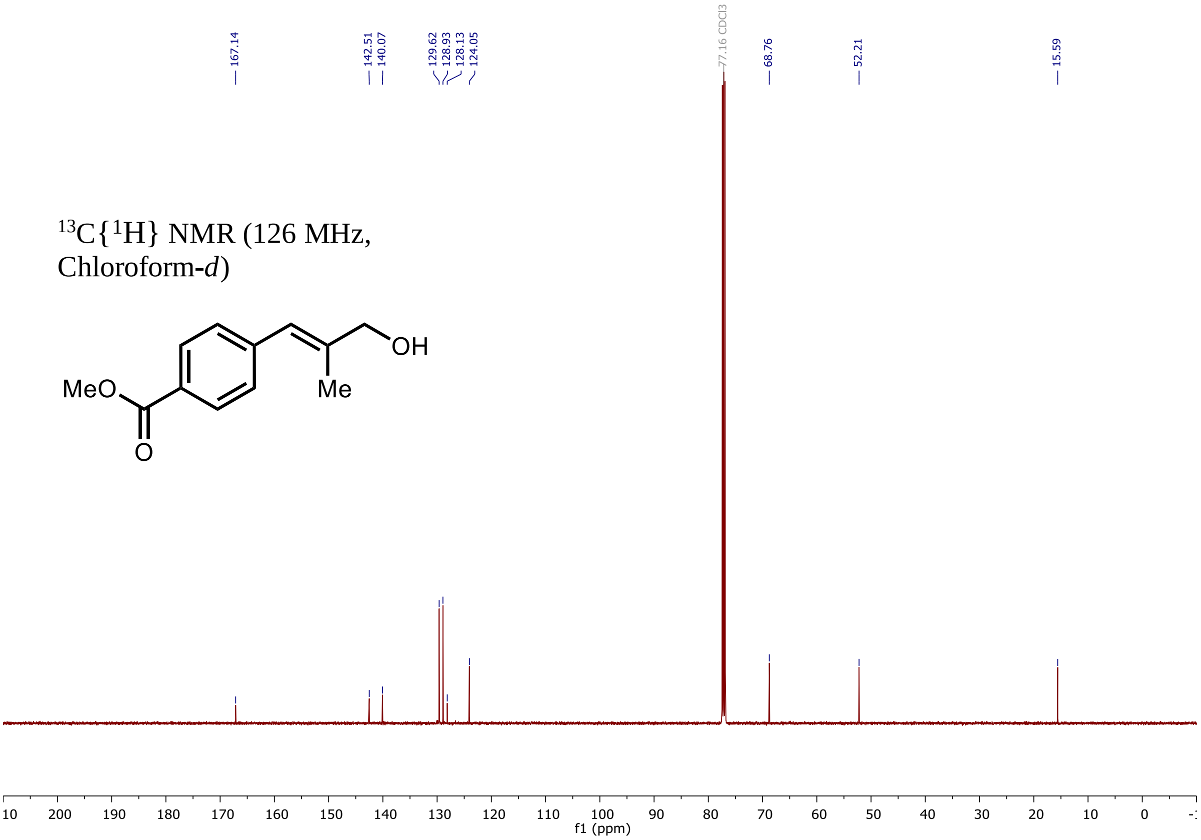

(*E*)-4-phenylbut-3-en-2-ol

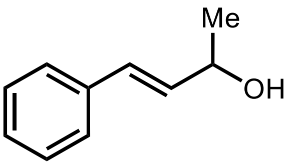

The title compound was prepared according to general procedure 6 using (*E*)-4-phenylbut-3-en-2-one (2 g, 14 mmol). Purification by flash silica chromatography (eluent = 20% EtOAc in petroleum ether, 35 × 160 mm silica) gave the title compound as a light yellow solid (1.55 g, 76%); mp 31-33 °C; R_f = 0.35 (eluent = 20% EtOAc in petroleum ether); ν_{max} / cm^{-1} (film) 3421, 2980, 2966, 2891, 1490, 1446, 1359, 1305, 1141, 1058, 962, 933, 875, 746, 690, 603, 547, 520, 455; ^{1}H NMR (500 MHz, Chloroform-*d*) δ 7.38 (ddd, *J* = 7.8, 1.4, 0.6 Hz, 2H), 7.32 (ddd, *J* = 7.8, 6.8, 1.2 Hz, 2H), 7.27 – 7.22 (m, 1H), 6.60 – 6.54 (m, 1H), 6.27 (dd, *J* = 15.9, 6.4 Hz, 1H), 4.50 (pd, *J* = 6.4, 1.3 Hz, 1H), 1.66 (bs, 1H), 1.38 (d, *J* = 6.4 Hz, 3H); ^{13}C{^{1}H} NMR (126 MHz, Chloroform-*d*) δ 136.8, 133.7, 129.6, 128.7, 127.8, 126.6, 69.1, 23.6; HRMS (ES-TOF) $(M - H)^+$ Calcd for $C_{10}H_{11}O$ 147.0810; Found 147.0808.

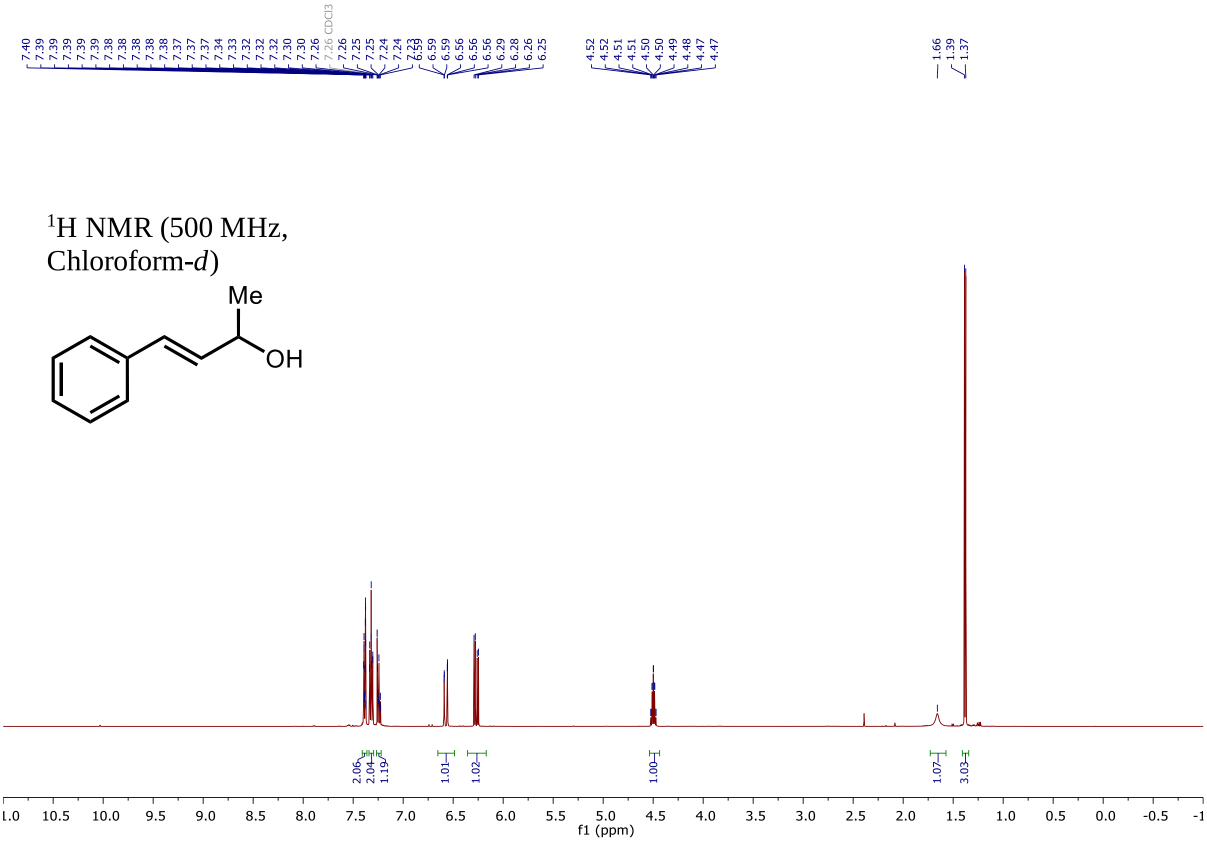

¹H NMR (500 MHz, Chloroform-d)
Me
OH
f1 (ppm)

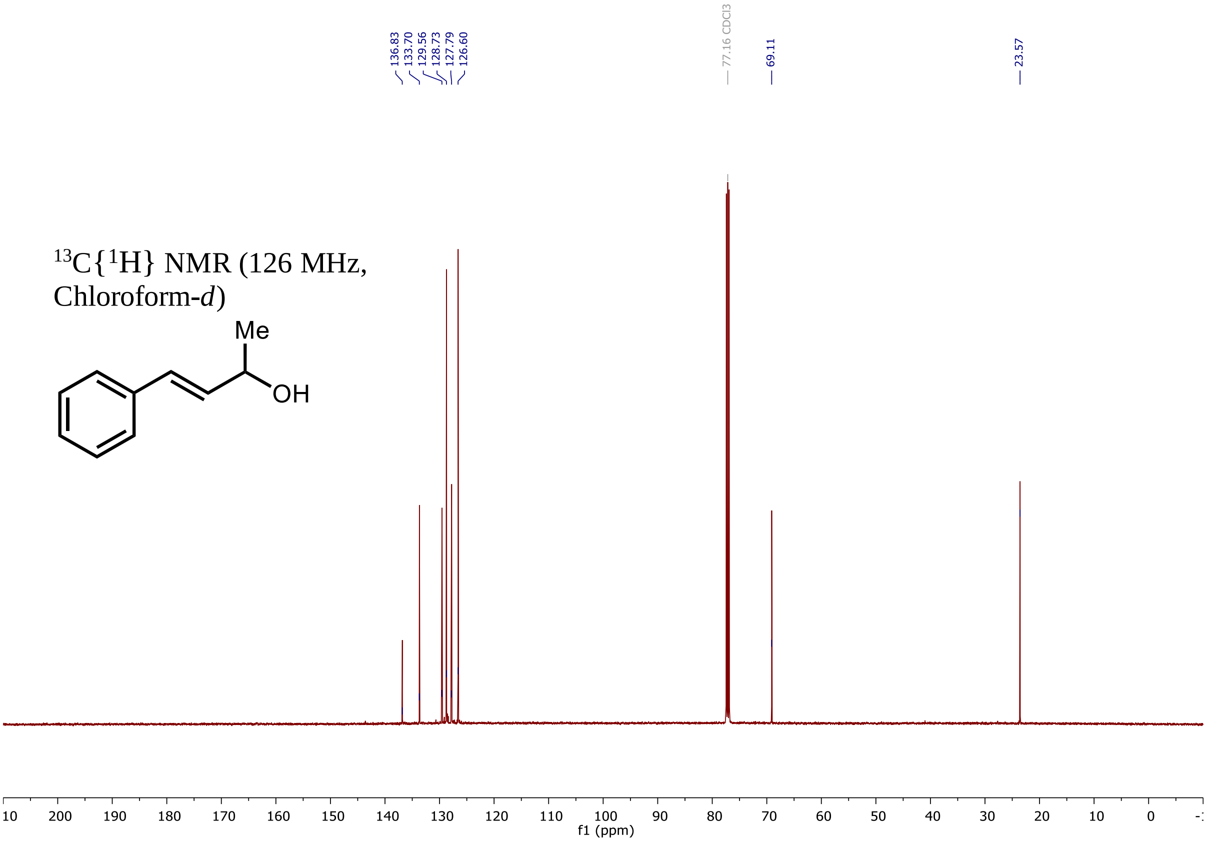

¹³C{¹H} NMR (126 MHz, Chloroform-d)
Me
OH
136.83
133.70
129.56
128.73
127.79
126.60
77.16 CDCl3
69.11
23.57
f1 (ppm)

(*E*)-4-phenylpent-3-en-2-ol

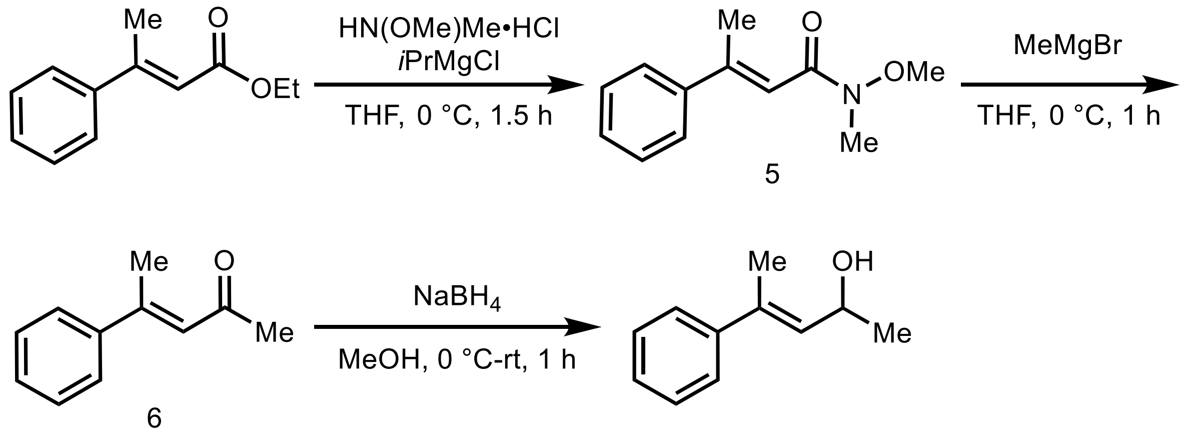

To a solution of ethyl (E)-3-phenylbut-2-enoate (3 g, 15.8 mmol, 1 equiv.) and N,O-dimethylhydroxyamine hydrochloride (3.1 g, 31.5 mmol, 2 equiv.) in dry THF (32 mL), *i*PrMgCl (2.0 M in THF, 23.7 mL, 47.4 mmol, 3 equiv.) was added at 0 °C dropwise over a period of 30 min. The mixture was stirred at 0 °C for 1 h, then quenched with saturated aqueous NH_4Cl. The solvent was evaporated under reduced pressure, and the aqueous layer was extracted with EtOAc. The organic layer was washed with water and brine, dried over anhydrous $MgSO_4$, filtered and concentrated in vacuo and used in the next step without further purification.

MeMgBr (27 mL, 1.0 M in THF) was added dropwise to a solution of **5** (1 g, 4.9 mmol, 1 equiv.) in THF (10 mL) at 0 °C and the mixture was stirred at 0 °C for1 h, then quenched with a saturated aqueous NH_4Cl (5 mL). The solvent was removed under reduced pressure, and the aqueous layer was extracted with EtOAc. The organic layer was washed with brine and dried over anhydrous $MgSO_4$, filtered and concentrated in vacuo and used in the next step without further purification.

$NaBH_4$ (170 mg, 4.5 mmol, 3 equiv.) was added to a solution of **6** (238 mg, 1.5 mmol, 1 equiv.) in MeOH (4.5 mL) at 0 °C and the mixture was stirred at rt for 1 h. The solvent was removed under reduced pressure and the residue was dissolved in DCM and washed with brine. The aqueous layer was extracted with DCM (3×). The organic layer was dried over anhydrous $MgSO_4$, filtered and concentrated in vacuo. Purification by flash silica chromatography (eluent = 20% EtOAc in petroleum ether, 35 × 160 mm silica) gave the title compound as a white solid (183 mg, 75%); mp 56-58 °C; R_f = 0.35 (eluent = 20% EtOAc in petroleum ether); ^{1}H NMR (500 MHz, Chloroform-*d*) δ 7.44 – 7.36 (m, 2H), 7.36 – 7.29 (m, 2H), 7.27 (m, 1H), 5.80 (dq, *J* = 8.3, 1.4 Hz, 1H), 4.76 (dq, *J* = 8.3, 6.3 Hz, 1H), 2.11 (d, *J* = 1.4 Hz, 3H), 1.54 (s, 1H), 1.35 (d, *J* = 6.3 Hz, 3H); ^{13}C{^{1}H} NMR (126 MHz, Chloroform-*d*) δ 143.0, 136.4, 132.0, 128.4,

127.4, 125.9, 65.4, 23.7, 16.3. Spectroscopic data in accordance with that stated in the literature.[13]

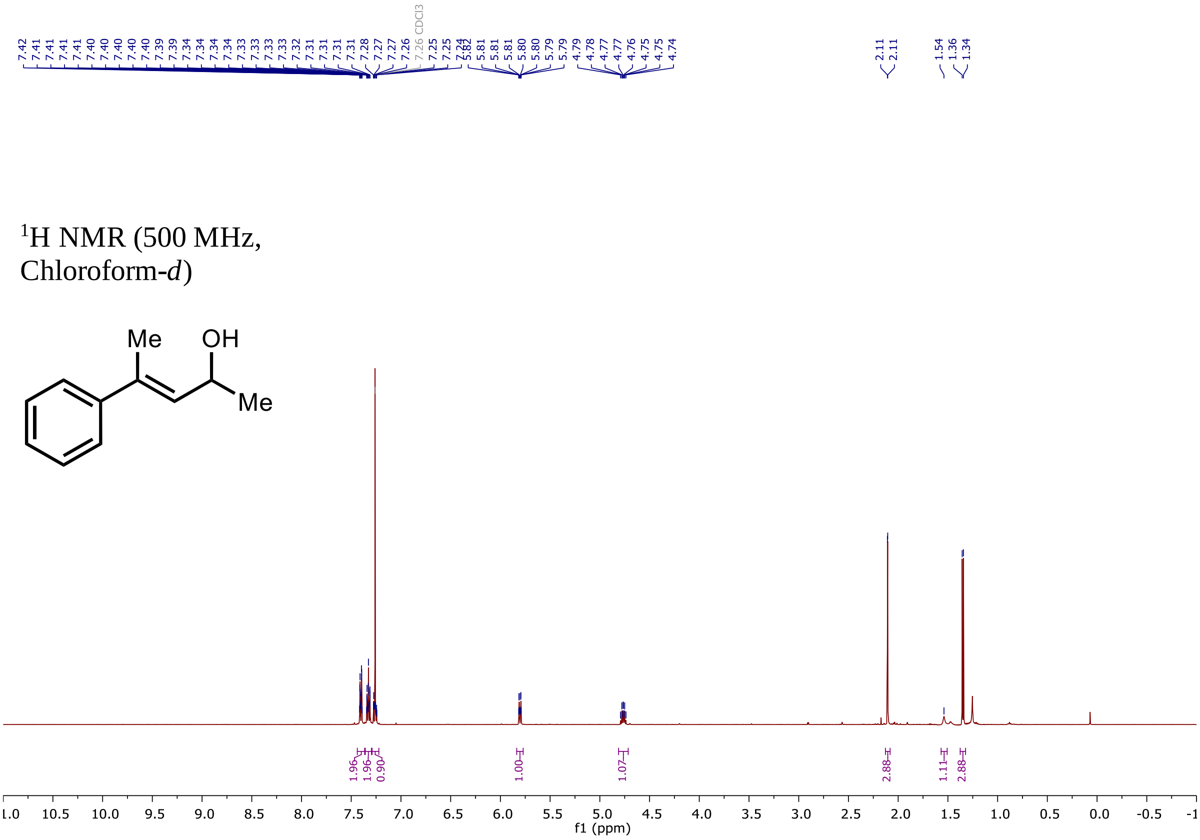

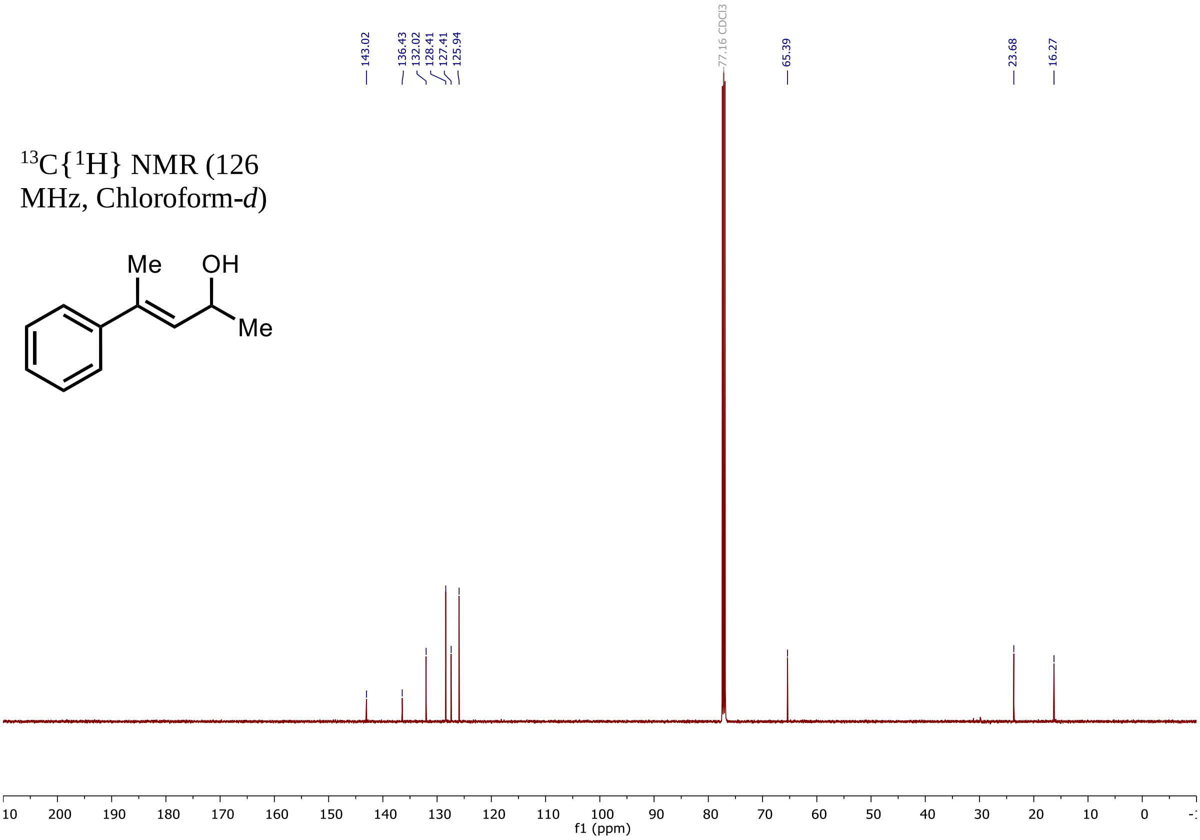

(*E*)-3-methyl-4-phenylbut-3-en-2-ol

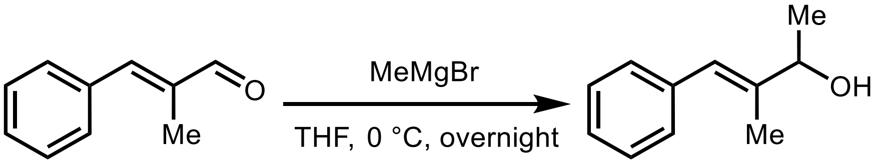

To a solution of (*E*)-2-methyl-3-phenylacrylaldehyde (2.0 mL, 14.0 mmol, 1 equiv.) in THF (20 mL) at 0 °C was added methyl magnesium bromide (3.00 equiv.) dropwise. The reaction mixture was allowed to warm to room temperature and left to stir overnight. The mixture was quenched with H_2O/saturated aqueous NH_4Cl solution (4:1) and then extracted with EtOAc (2×). The layers were separated, and the organics were dried over MgSO4, filtered and concentrated in vacuo. Purification by flash silica chromatography (eluent = 10-30% EtOAc in petroleum ether, 35 × 160 mm silica) to give the title compound as a yellowish oil (1.73 g, 76%); R_f = 0.12 (eluent = 10% EtOAc in petroleum ether); ν_{max} / cm^{-1} (film) 3329, 2972, 1600, 1510, 1244, 1117, 1072, 1026, 964, 742, 695, 516; ^{1}H NMR (300 MHz, Chloroform-*d*) δ 7.36 – 7.31 (m, 2H), 7.30 – 7.26 (m, 2H), 7.24 – 7.20 (m, 1H), 6.52 (s, 1H), 4.39 (q, *J* = 6.4 Hz, 1H), 1.89 (d, *J* = 1.4 Hz, 3H), 1.56 (s, 1H), 1.37 (d, *J* = 6.4 Hz, 3H); ^{13}C{^{1}H} NMR (126 MHz, Chloroform-*d*) δ 141.6, 137.5, 128.9, 128.0, 126.2, 124.1, 73.3, 21.7, 13.3; HRMS (EI-quadrupole) ((M + H - $H_2O)^+$ Calcd for $C_{11}H_{13}$ 145.1017; Found 145.1017.

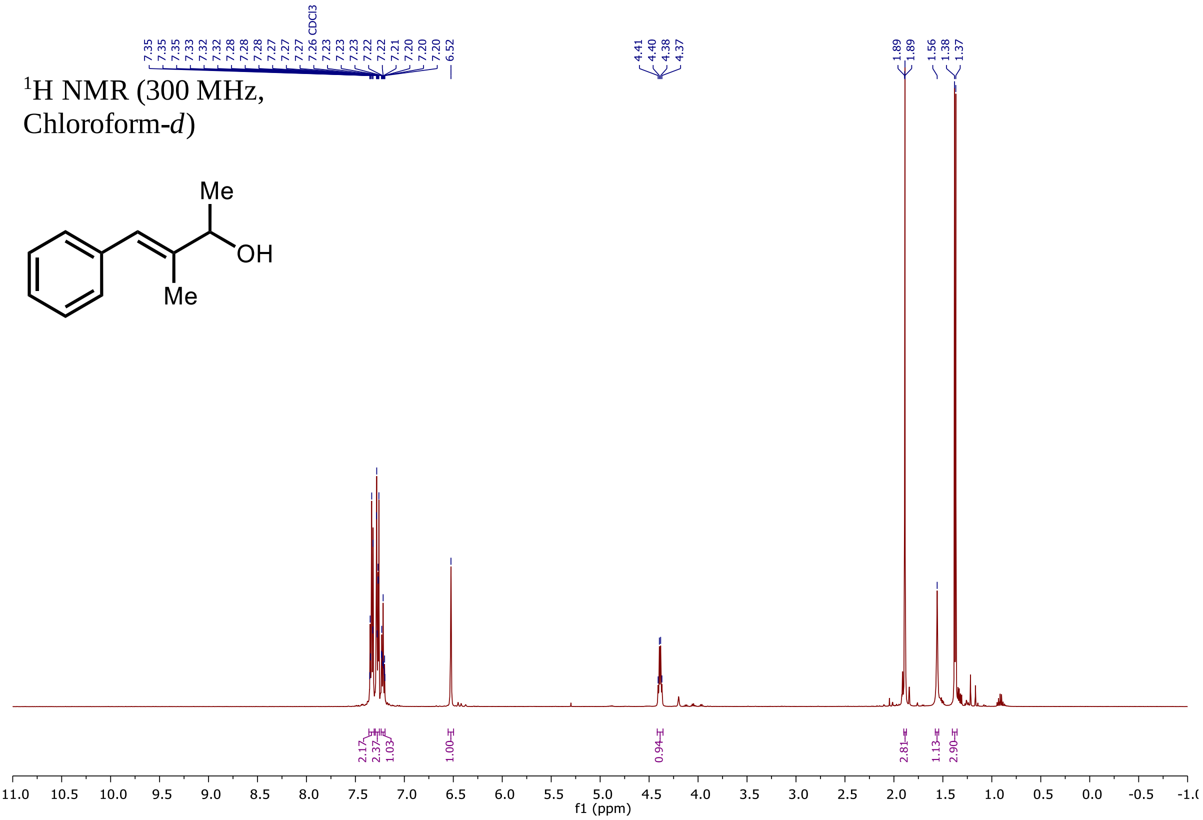

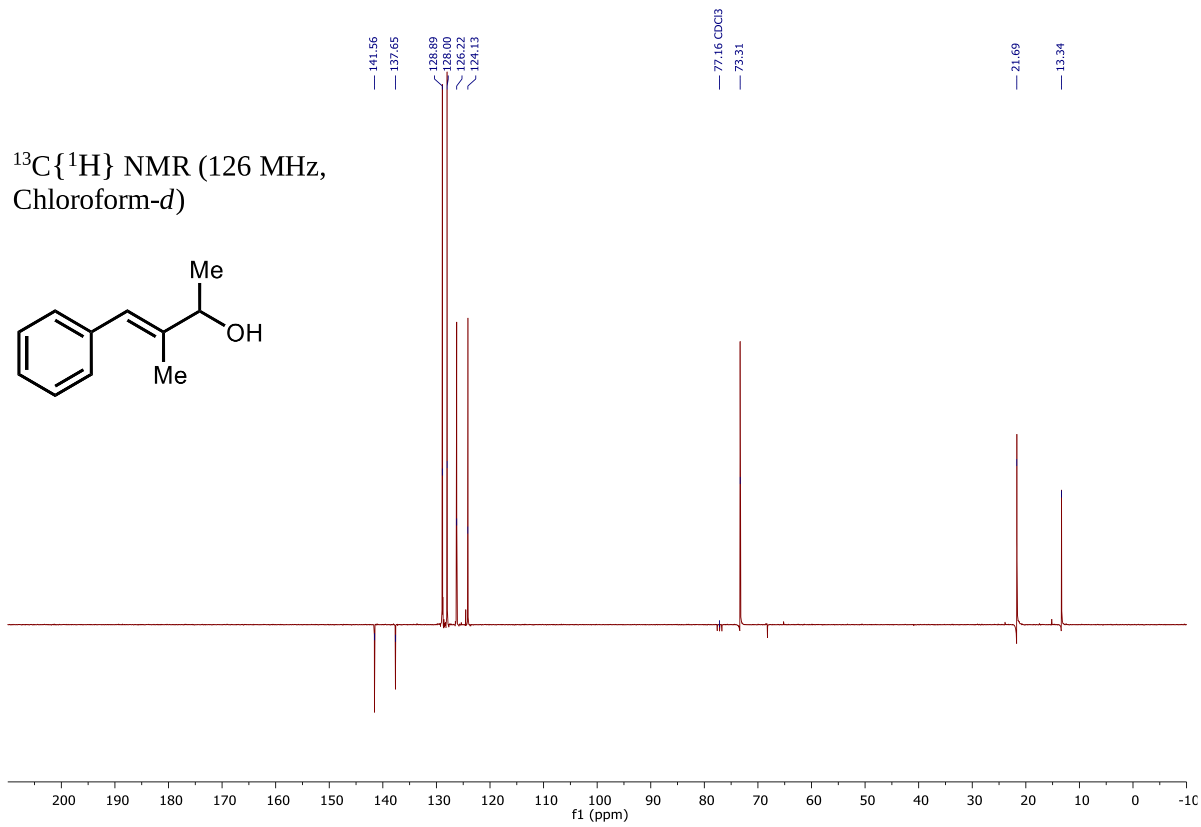

(*E*)-2,3-dimethyl-4-phenylbut-3-en-2-ol (41)

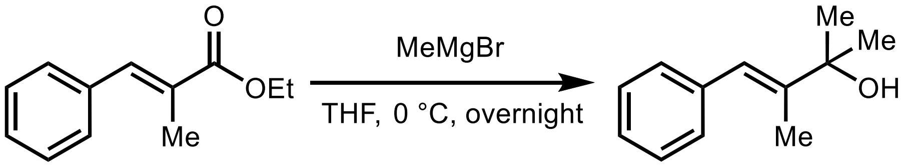

To a solution of ethyl (*E*)-2-methyl-3-phenylacrylate (2 g, 10.5 mmol) in THF (20 mL) at 0 °C was added methyl magnesium bromide (3.00 equiv.) dropwise. The reaction mixture was allowed to warm to room temperature and left to stir overnight. The mixture was quenched with H_2O/saturated aqueous NH_4Cl solution (4:1) and then extracted with EtOAc (2×). The layers were separated, and the organics were dried over MgSO4, filtered and concentrated in vacuo. Purification by flash silica chromatography (eluent = 20% EtOAc in petroleum ether, 35 × 160 mm silica) to give the title compound as a white solid (1.39 g, 70%); mp 45-46 °C, R_f = 0.15 (eluent = 20 % EtOA in petroleum ether); ν_{max} / cm^{-1} (film) 3331, 2976, 1598, 1440, 1361, 1106, 696, 517; ^{1}H NMR (500 MHz, Chloroform-*d*) δ 7.35 – 7.31 (m, 2H), 7.26 – 7.23 (m, 2H), 7.23 – 7.18 (m, 1H), 6.67 (s, 1H), 1.90 (d, *J* = 1.3 Hz, 3H), 1.50 (s, 1H), 1.45 (s, 6H); ^{13}C{^{1}H} NMR (126 MHz, Chloroform-*d*) δ 144.8, 138.5, 129.2, 128.2, 126.3, 122.5, 74.1, 29.2, 14.6; HRMS (EI-quadrupole) (M + H - H_2O)$^+$ Calcd for $C_{12}H_{15}$ 159.1174; Found 159.1172.

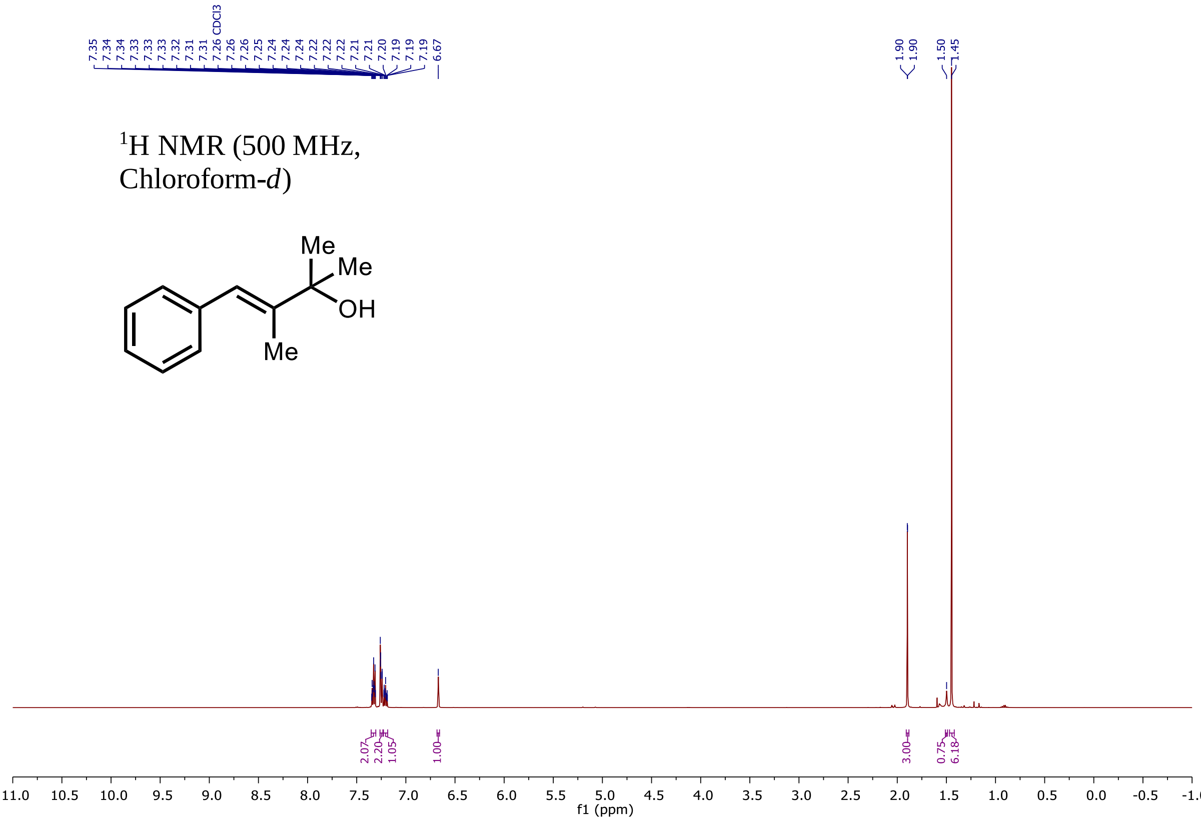

7.35 7.34 7.34 7.33 7.33 7.33 7.32 7.31 7.31 7.26 CDCl3 7.26 7.26 7.25 7.24 7.24 7.24 7.22 7.22 7.22 7.21 7.21 7.20 7.19 7.19 7.19 6.67
1.90 1.90 1.50 1.45
^{1}H NMR (500 MHz, Chloroform-*d*)
Me
Me
OH
Me
2.07 2.20 1.05 1.00 3.00 0.75 6.18
11.0 10.5 10.0 9.5 9.0 8.5 8.0 7.5 7.0 6.5 6.0 5.5 5.0 4.5 4.0 3.5 3.0 2.5 2.0 1.5 1.0 0.5 0.0 -0.5
f1 (ppm)

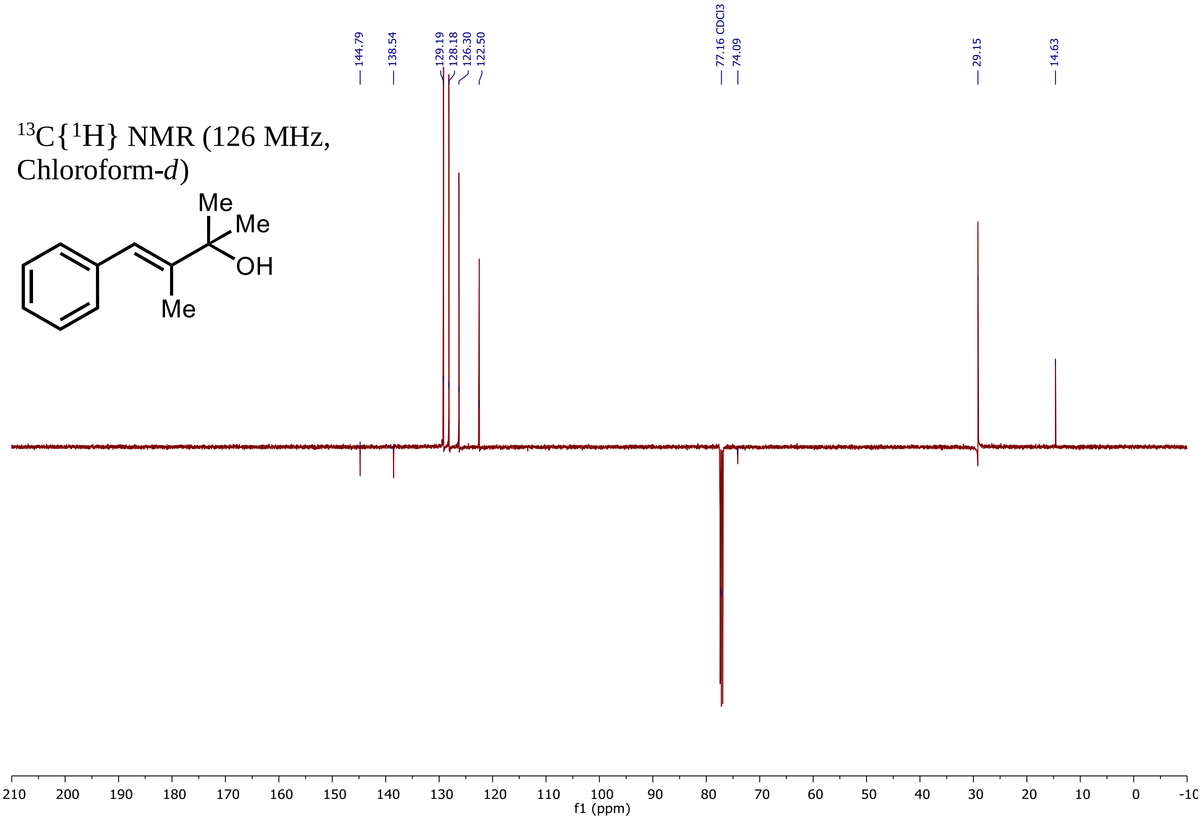

144.79 138.54 129.19 128.18 126.30 122.50 77.16 CDCl3 74.09 29.15 14.63
^{13}C{^{1}H} NMR (126 MHz, Chloroform-*d*)
Me
Me
OH
Me
210 200 190 180 170 160 150 140 130 120 110 100 90 80 70 60 50 40 30 20 10 0
f1 (ppm)

2-phenylprop-2-en-1-ol

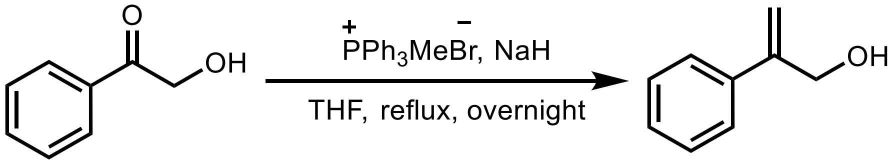

NaH (60% dispersion in mineral oil) (880 mg, 22 mmol, 2 equiv.) was added to a solution of methyltriphenylphosphonium bromide (6.3 g, 17.6 mmol, 1.6 equiv.) in dry THF (35 mL) at 0 °C and the mixture was stirred at reflux for 2 h. Then a solution of 2-hydroxyacetophenone (1.5 g, 11 mmol, 1 equiv.) in dry THF (8 mL) was added dropwise over 10 min at 0 °C and the mixture was stirred at reflux overnight. After cooling, the reaction mixture was quenched with water and the organic layer was separated. The aqueous layer was extracted with EtOAc (3×) and the combined organics were washed with brine (1×), dried over anhydrous $MgSO_4$, filtered and concentrated in vacuo. Purification by flash silica chromatography (eluent = 20% EtOAc in petroleum ether, 35 × 160 mm silica) gave the title compound as a yellow oil (246 mg, 12%); R_f = 0.45 (eluent = 20% EtOAc in petroleum ether); ^{1}H NMR (500 MHz, Chloroform-*d*) δ 7.49 – 7.42 (m, 2H), 7.40 – 7.32 (m, 2H), 7.34 – 7.27 (m, 1H), 5.48 (q, *J* = 0.9 Hz, 1H), 5.36 (q, *J* = 1.4 Hz, 1H), 4.55 (s, 2H), 1.60 (s, 1H); ^{13}C{^{1}H} NMR (126 MHz, Chloroform-*d*) δ 147.4, 138.6, 128.7, 128.1, 126.2, 112.8, 65.2. Spectroscopic data in accordance with that stated in the literature.[14]

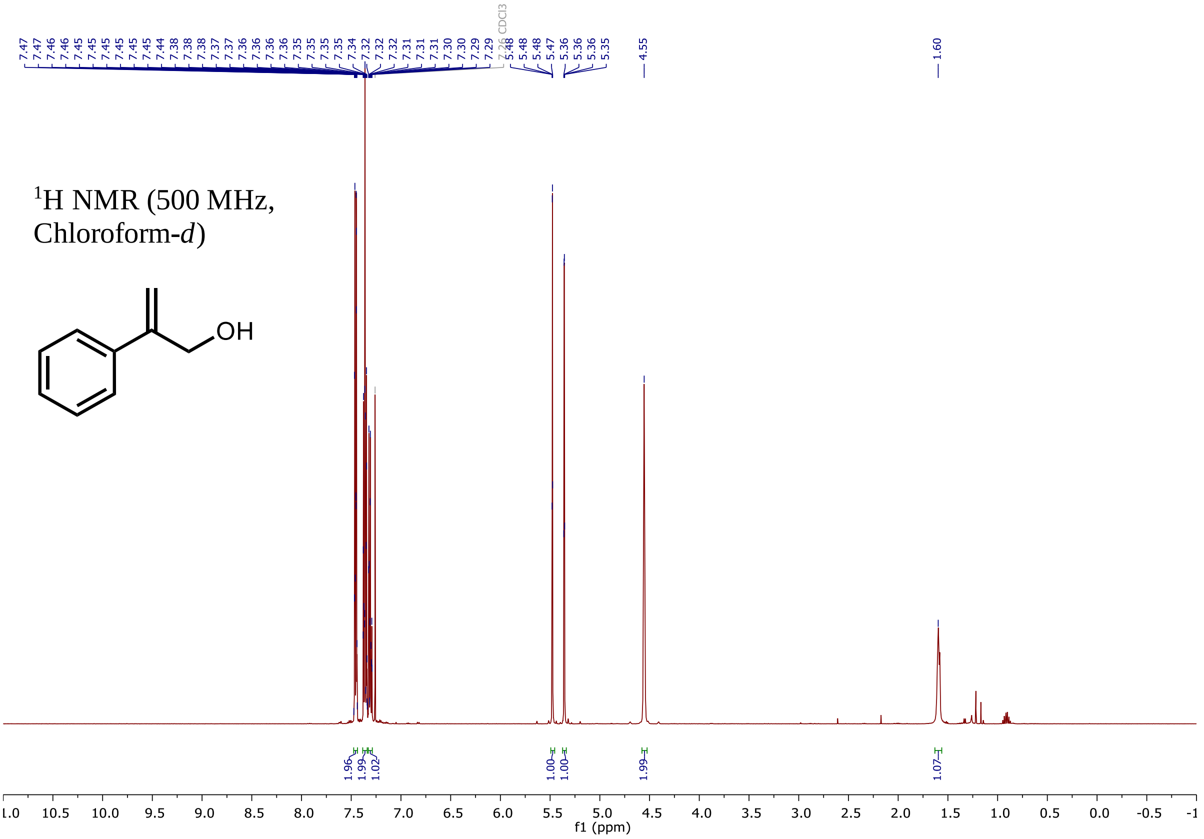

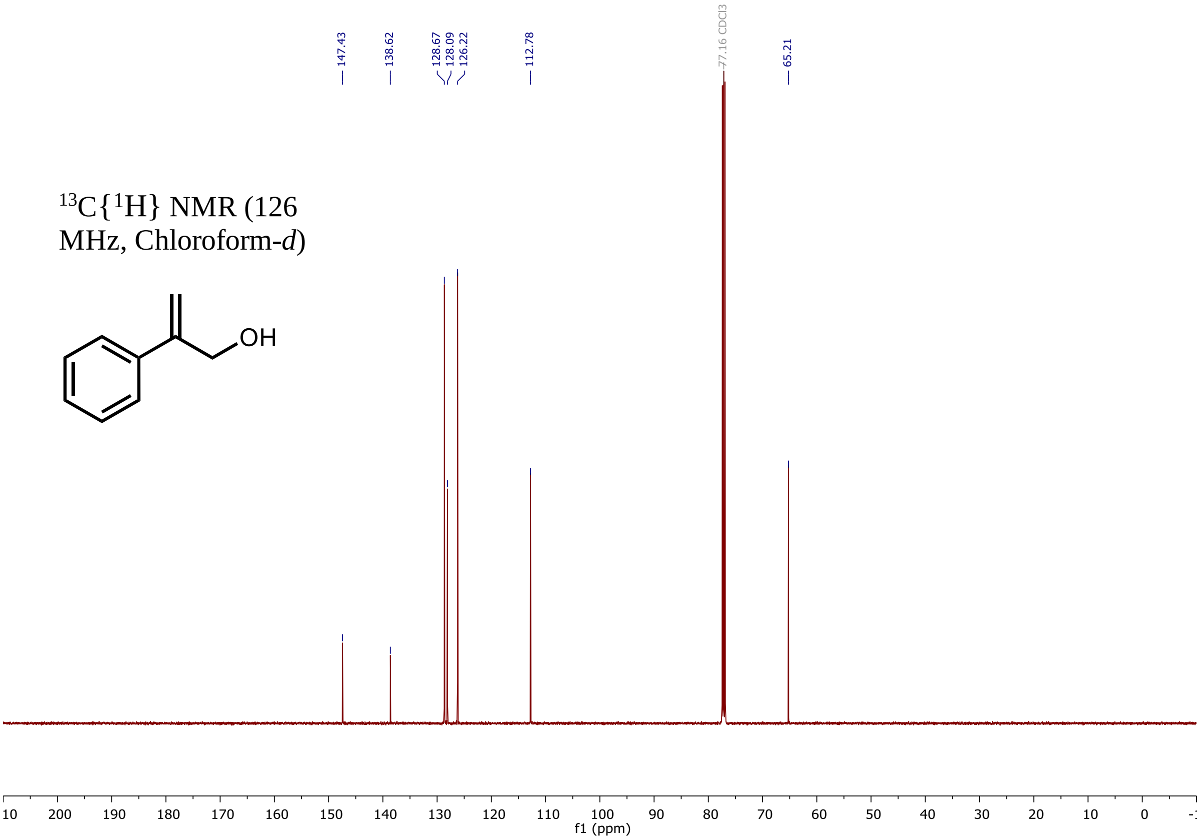

(*E*)-2-methyl-3-phenylprop-2-en-1,1-*d*₂-1-ol (47)

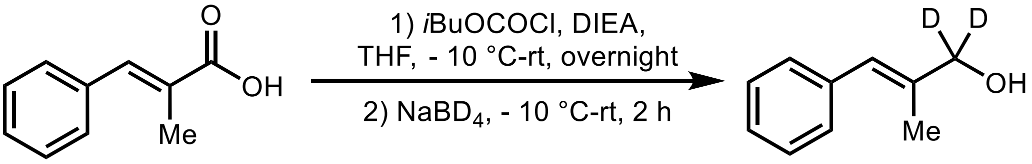

A solution of isobutyl chloroformate (1.9 mL, 14.8 mmol, 1.2 equiv.) in THF (4 mL) was added dropwise to a solution of (E)-2-methyl-3-phenylacrylic acid (2 g, 12.3 mmol, 1 equiv.) and diisopropylethylamine (4.3 mL, 24.6 mmol, 2 equiv.) in THF (45 mL). The mixture was stirred at –10 ° C for 1 h and then at rt overnight. $NaBD_4$ was then added to the mixture portion wise at –10 ° C and stirred for 1 h at the same temperature and for 1 h at rt. The reaction mixture was quenched with H_2O at –10 ° C and stirred at rt for 1 h and neutralized with 10% aqueous citric acid solution and extracted with EtOAc (2×). The combined organic layer was dried over anhydrous $MgSO_4$, filtered and concentrated in vacuo. Purification by flash silica chromatography (eluent = 15-20% EtOAc in petroleum ether, 35 × 160 mm silica) gave the title compound as a yellow oil (457 mg, 25%); R_f = 0.38 (eluent = 30% EtOAc in petroleum ether); ν_{max} / cm^{-1} (film) 3337, 3055, 3022, 2980, 1543, 1508, 1489, 1105, 1070, 957, 920, 739, 698, 418; ^1H NMR (500 MHz, Chloroform-*d*) δ 7.38 – 7.30 (m, 2H), 7.32 – 7.25 (m, 2H), 7.26 – 7.19 (m, 1H), 6.53 (s, 1H), 4.18 (q, *J* = 1.7 Hz, 0.25H), 1.91 (d, *J* = 1.5 Hz, 3H), 1.64 (s, 1H);

$^{13}C\{^{1}H\}$ NMR (126 MHz, Chloroform-*d*) δ 137.8 – 137.6 (m), 129.0, 128.3, 126.6, 125.3 (d, *J* = 8.8 Hz), 69.4 – 68.2 (m), 15.4 (d, *J* = 1.5 Hz); HRMS (CI-quadrupole) $(M)^+$ Calcd for $C_{10}H_{10}{}^{2}H_2O$ 150.1008; Found 150.1009.

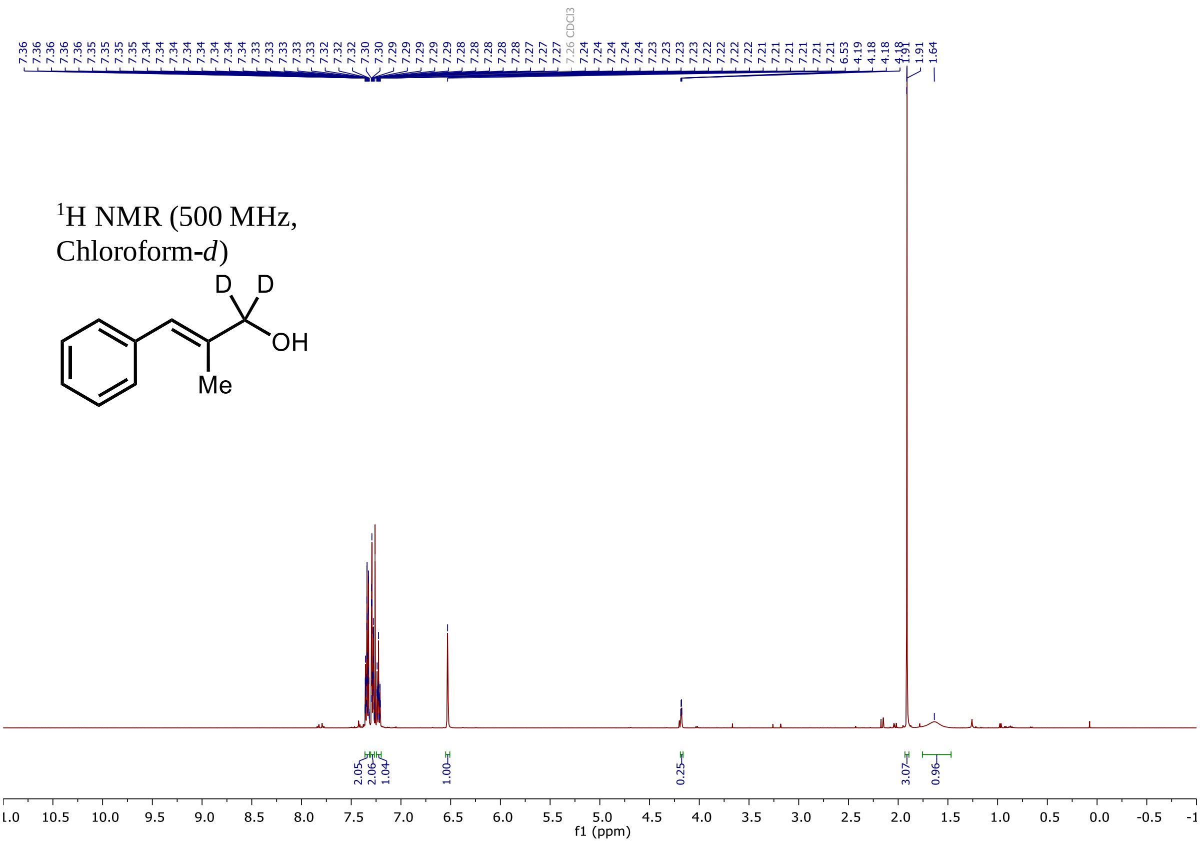

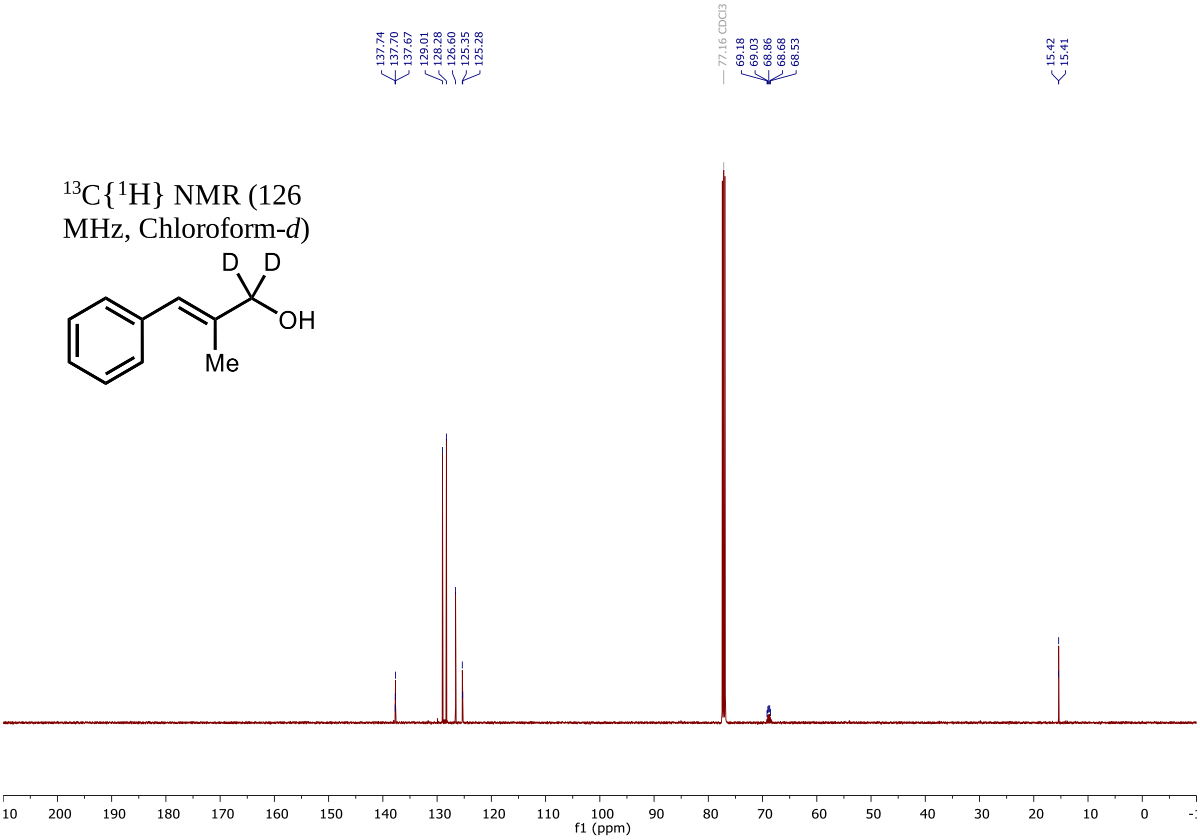

2.2. Optimization of reaction conditions

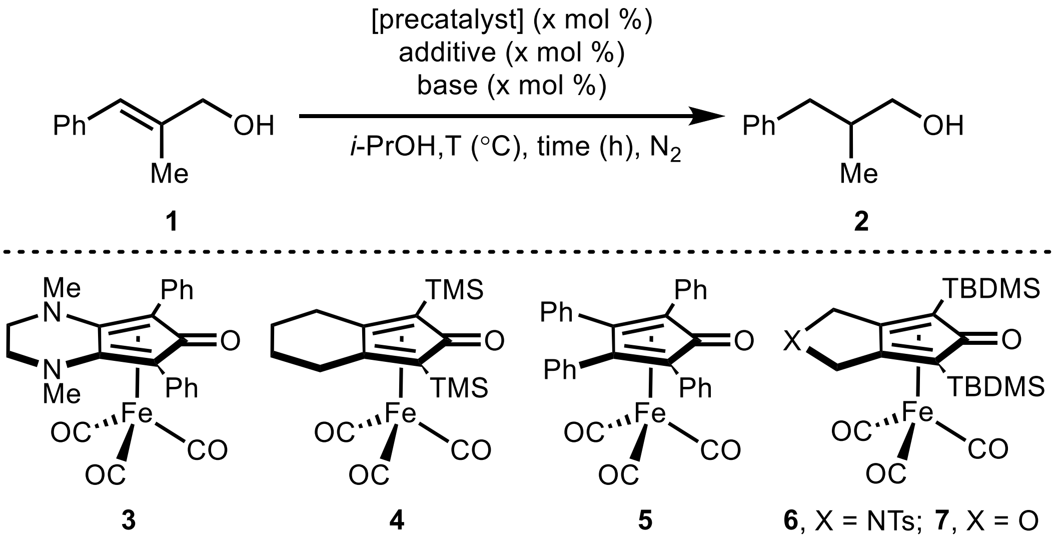

A 20 mL microwave vial with a magnetic stirrer bar was charged with 2-methyl-3-phenylpropan-1-ol (144 µL, 148 mg, 1 mmol), base (x mol%), additive (x mol%), and precatalyst (x mol%). The vial was firmly sealed with a cap and was placed under vacuum. After 5 minutes, the vial was flushed with nitrogen and the cycle was repeated three times. Under nitrogen, the vial was then charged with dry isopropanol (x mL), placed on a preheated heating block at the specified temperature (°C) and time (h). It was then cooled and the cap was removed followed by the addition of mesitylene (139 µL, 120 mg, 1 mmol) and it was stirred for 2 minutes. Saturated aqueous NH_4Cl (2 mL) was then added and the mixture was stirred for further 2 minutes, followed by the addition of EtOAc (2 mL) and stirring for 2 minutes. Then the mixture was left to settle for 5 minutes, the top layer was sampled and analysed using ^{1}H NMR.

Entry[a]	Precat. loading (mol%)	Additive (mol%)	Base (mol%)	Solvent [**1**]	T (°C)	time (h)	**2** (%)[b]
1	**[Fe] 3 (4)**	$Me_3NO.2H_2O$ (8)	K_2CO_3 (4)	IPA (0.5M)	130	18	**93**
2	**[Fe] 4 (4)**	$Me_3NO.2H_2O$ (8)	K_2CO_3 (4)	IPA (0.5M)	130	18	72
3	**[Fe] 5 (4)**	$Me_3NO.2H_2O$ (8)	K_2CO_3 (4)	IPA (0.5M)	130	18	<2
4	**[Fe] 6 (4)**	$Me_3NO.2H_2O$ (8)	K_2CO_3 (4)	IPA (0.5M)	130	18	6
5	**[Fe] 7 (4)**	$Me_3NO.2H_2O$ (8)	K_2CO_3 (4)	IPA (0.5M)	130	18	5

6	[Fe] 3 (4)	$Me_3NO.2H_2O$ (**4**)	K_2CO_3 (4)	IPA (0.5M)	130	18	86
7	[Fe] 3 (2)	$Me_3NO.2H_2O$ (8)	K_2CO_3 (4)	IPA (0.5M)	130	18	69
8	[Fe] 3 (2)	$Me_3NO.2H_2O$ (**4**)	K_2CO_3 (4)	IPA (0.5M)	130	18	78
9	[Fe] 2 (4)	$Me_3NO.2H_2O$ (8)	K_2CO_3 (**2**)	IPA (0.5M)	130	18	92
10	[Fe] 3 (4)	$Me_3NO.2H_2O$ (8)	**NaO^tBu** (4)	IPA (0.5M)	130	18	61
11	[Fe] 3 (4)	$Me_3NO.2H_2O$ (8)	**KO^tBu** (4)	IPA (0.5M)	130	18	92
12	[Fe] 3 (4)	$Me_3NO.2H_2O$ (8)	**KOH** (4)	IPA (0.5M)	130	18	44
13	[Fe] 3 (4)	$Me_3NO.2H_2O$ (8)	**NaOH** (4)	IPA (0.5M)	130	18	43
14	[Fe] 3 (4)	$Me_3NO.2H_2O$ (8)	**Na_2CO_3** (4)	IPA (0.5M)	130	18	86
15	[Fe] 3 (4)	$Me_3NO.2H_2O$ (8)	**Cs_2CO_3** (4)	IPA (0.5M)	130	18	81
16	[Fe] 3 (4)	$Me_3NO.2H_2O$ (8)	K_2CO_3 (4)	IPA (0.5M)	**110**	18	36
17	[Fe] 3 (4)	$Me_3NO.2H_2O$ (8)	K_2CO_3 (4)	IPA (0.5M)	**90**	18	2
18	[Fe] 3 (4)	$Me_3NO.2H_2O$ (8)	K_2CO_3 (4)	IPA (0.5M)	130	**8**	86
19	[Fe] 3 (4)	$Me_3NO.2H_2O$ (8)	K_2CO_3 (4)	**IPA (1M)**	130	18	79
20	[Fe] 3 (4)	$Me_3NO.2H_2O$ (8)	K_2CO_3 (4)	**IPA (0.25M)**	130	18	80
21	-	$Me_3NO.2H_2O$ (8)	K_2CO_3 (4)	IPA (0.5M)	130	18	<2
22	[Fe] 3 (4)	-	K_2CO_3 (4)	IPA (0.5M)	130	18	94
23	[Fe] 3 (4)	-	**KOH** (4)	IPA (0.5M)	130	18	89
24	[Fe] 3 (4)	$Me_3NO.2H_2O$ (8)	-	IPA (0.5M)	130	18	<2
25	[Fe] 3 (4)	**-**	K_2CO_3 (4)	IPA (0.5M)	130	**24**	**99**

[a] Reactions performed using **1** (1 mmol) and reagent grade *i*-PrOH. [**1**] = 0.5 M. [b] Yield after 18 h as determined by ^{1}H NMR analysis of the crude reaction mixture with 1,3,5-trimethylbenzene as the internal standard.

2.3. Scope of the iron-catalyzed TH protocol

2.3.1 General procedure 7

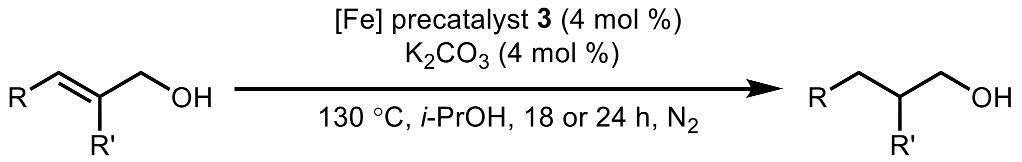

A 20 mL microwave vial with a magnetic stirrer bar was charged with potassium carbonate (4 mol%), allylic alcohol (1 mmol) and [Fe] precatalyst **3** (4 mol %). The vial was firmly sealed with a cap and was placed under vacuum. After 5 minutes, the vial was flushed with nitrogen and the cycle was repeated three times. Under nitrogen, the vial was then charged with dry isopropanol (2 mL), placed on a preheated heating block at 130 °C and stirred for 18 or 24 h. The mixture was then cooled and the cap was removed followed by the addition of mesitylene (139 µL, 120 mg, 1 mmol) and it was stirred for 2 minutes. Saturated aqueous NH_4Cl (2 mL) was then added and the mixture was stirred for further 2 minutes, followed by the addition of EtOAc (2 mL) and stirring for 2 minutes. Then the mixture was left to settle for 5 minutes, the top layer was sampled and analysed using ^{1}H NMR. The mixture was diluted with EtOAc (25 mL), and transferred to a separatory funnel containing brine (25 mL). The aqueous phase was extracted with EtOAc (2 × 25 mL). The combined organic layers were dried over $MgSO_4$, filtered and concentrated in vacuo.

2-methyl-3-phenylpropan-1-ol (2)

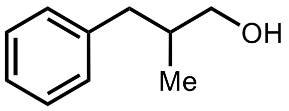

The title compound was prepared according to general procedure 7 using (*E*)-2-methyl-3-phenylprop-2-en-1-ol (148 mg, 1 mmol) for 24 h and purified by flash silica chromatography (20% EtOAc in petroleum ether, 35 × 160 mm silica) to give the title compound as a beige coloured viscous oily liquid (120 mg, 80% isolated yield, >98% NMR yield); R_f = 0.15 (10% EtOAc in petroleum ether); ^{1}H NMR (500 MHz, Chloroform-*d*) δ 7.29 (t, *J* = 7.5 Hz, 2H), 7.19 (dd, *J* = 13.8, 6.9 Hz, 3H), 3.54 (dd, *J* = 10.6, 5.8 Hz, 1H), 3.48 (dd, *J* = 10.5, 6.1 Hz, 1H), 2.76 (dd, *J* = 13.5, 6.3 Hz, 1H), 2.43 (dd, *J* = 13.5, 8.1 Hz, 1H), 1.95 (dp, *J* = 7.8, 6.4 Hz, 1H), 1.46 (s, 1H), 0.92 (d, *J* = 6.7 Hz, 3H); ^{13}C{^{1}H} NMR (126 MHz, Chloroform-*d*) δ 140.8, 129.3, 128.4, 126.0, 67.8, 39.9, 37.9, 16.6. Spectroscopic data are in accordance with that stated in the literature.[15]

5.5 mmol scale

An 110 mL ACE pressure tube rated at 150 PSI with a magnetic stirrer bar was charged with dry potassium carbonate (30 mg, 0.22 mmol, 4 mol%), dry 2-methylcinnamyl alcohol (814 mg, 792 µL, 5.5 mmol) and [Fe] precatalyst **3** (99 mg, 0.22 mmol, 4 mol%). It was flushed with nitrogen and under the running nitrogen, the tube was then charged with dry isopropanol (11 mL). It was sealed with the appropriate screw top cap, placed in a preheated oil bath at 130 °C behind a blast shield and the mixture was stirred for 24 h. It was then cooled at room temperature, followed by the addition of mesitylene (764.5 µL, 5.5 mmol), saturated NH_4Cl (11 mL) and EtOAc (11 mL). The mixture was stirred for 5 minutes, left to settle for a further 5 minutes, the top layer was sampled and analysed using ^1H NMR. The mixture was diluted with EtOAc (100 mL), and transferred to a separatory funnel containing brine (100 mL). The organic layer was collected, and the aqueous phase washed with EtOAc (2 x 100 mL). The organic layers were combined, dried over $MgSO_4$, filtered and concentrated in vacuo. Purification by flash silica chromatography (eluent = 10-20% EtOAc in petroleum ether, 35 × 160 mm silica) gave a beige coloured viscous oily liquid (660 mg, 80% isolated yield, 88% NMR yield). Spectroscopic data in accordance with that reported previously.

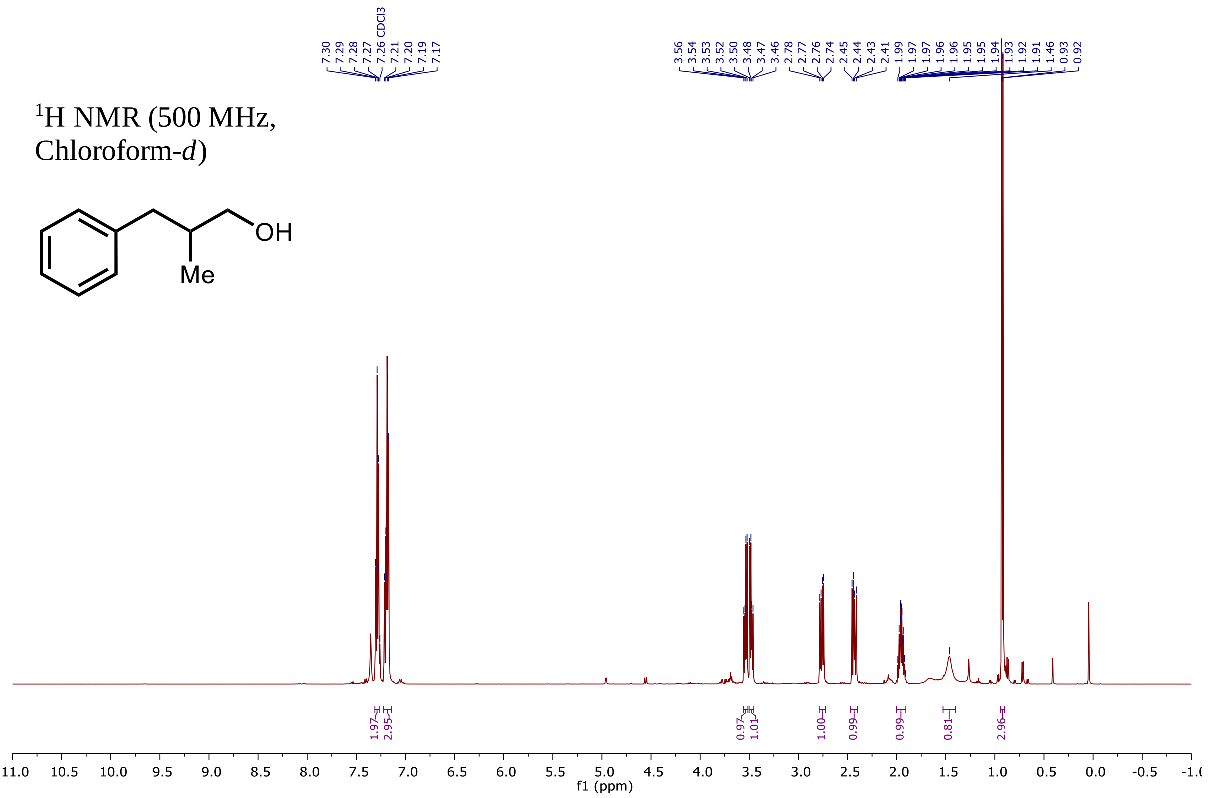

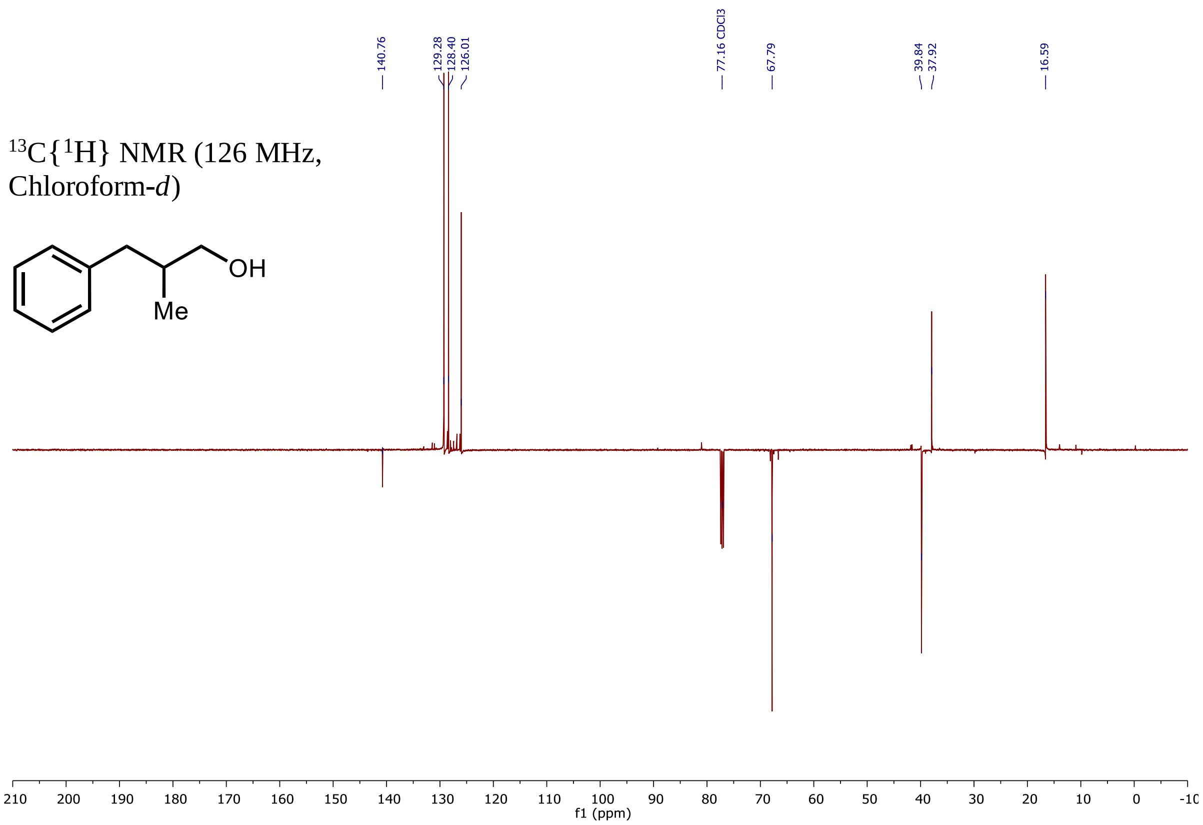

2-methyl-3-(p-tolyl)propan-1-ol (8)

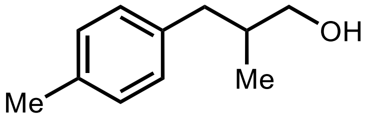

The title compound was prepared according to general procedure 7 using (*E*)-2-methyl-3-(*p*-tolyl)prop-2-en-1-ol (162 mg, 1 mmol) for 24 h and purified by flash silica chromatography (5% EtOAc in petroleum ether, 35 × 160 mm silica) to give the title compound as yellowish oil (136 mg, 83% isolated yield, 95% NMR yield); R_f = 0.22 (20% EtOAc in petroleum ether); ^{1}H NMR (500 MHz, Chloroform-*d*) δ 7.10 (d, *J* = 7.9 Hz, 2H), 7.06 (d, *J* = 8.1 Hz, 2H), 3.54 (dd, *J* = 10.6, 5.9 Hz, 1H), 3.47 (dd, *J* = 10.6, 6.0 Hz, 1H), 2.71 (dd, *J* = 13.5, 6.4 Hz, 1H), 2.40 (dd, *J* = 13.5, 8.0 Hz, 1H), 2.32 (s, 3H), 2.00 – 1.87 (m, 1H), 1.27 (s, 1H), 0.92 (d, *J* = 6.7 Hz, 3H); ^{13}C{^{1}H} NMR (126 MHz, Chloroform-*d*) δ 137.6, 135.5, 129.2, 129.1, 67.9, 39.4, 38.0, 21.2, 16.6. Spectroscopic data are in accordance with that stated in the literature.[16]

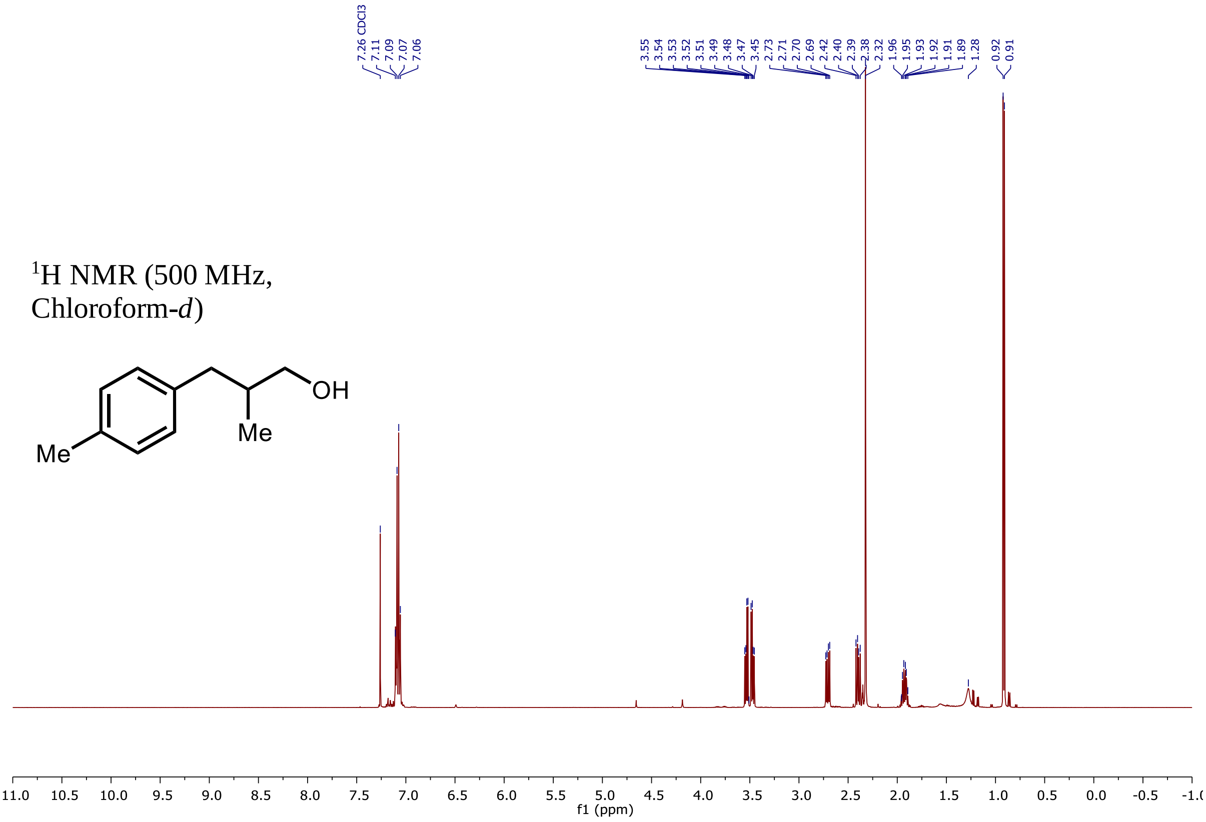
7.26 CDCl3
7.11
7.09
7.07
7.06
3.55
3.54
3.53
3.52
3.51
3.49
3.48
3.47
3.45
2.73
2.71
2.70
2.69
2.42
2.40
2.39
2.38
2.32
1.96
1.95
1.93
1.92
1.91
1.89
1.28
0.92
0.91
¹H NMR (500 MHz, Chloroform-d)
OH
Me
Me
f1 (ppm)

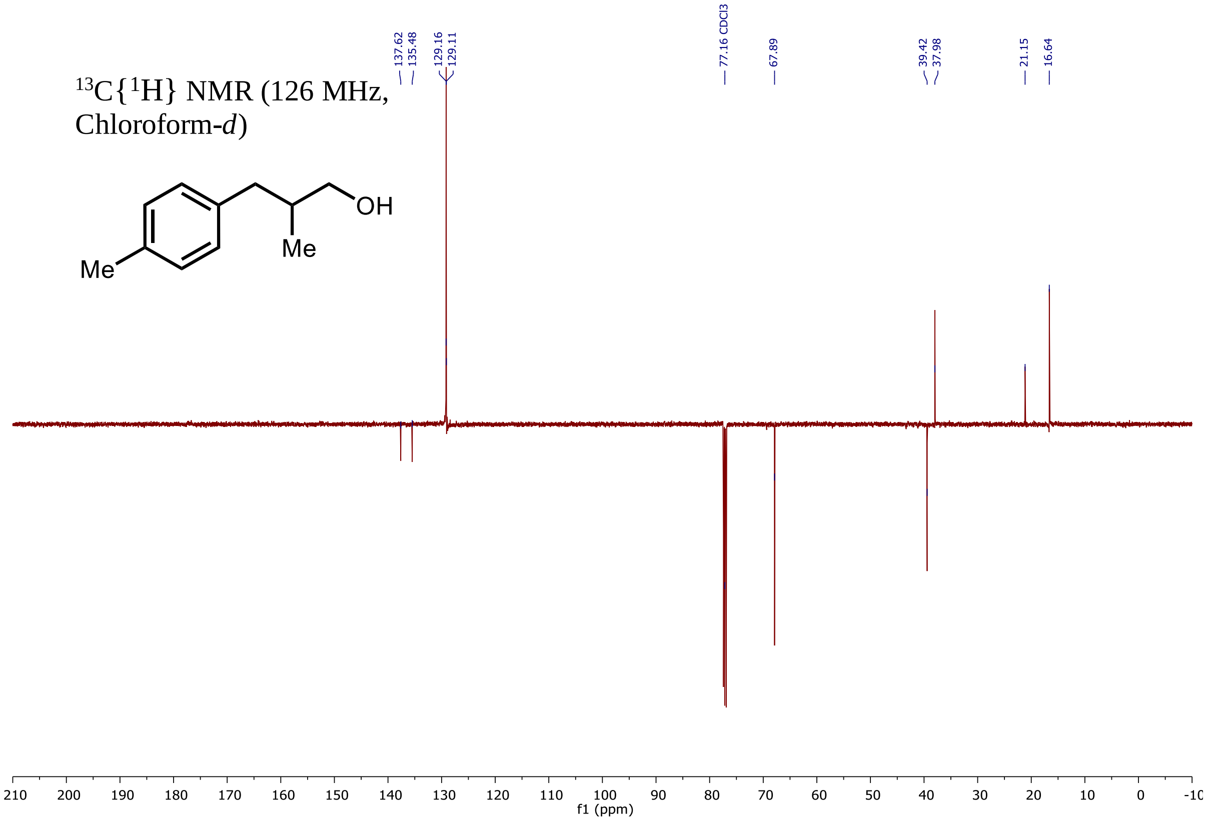
137.62
135.48
129.16
129.11
77.16 CDCl3
67.89
39.42
37.98
21.15
16.64
¹³C{¹H} NMR (126 MHz, Chloroform-d)
OH
Me
Me
f1 (ppm)

2-methyl-3-(*m*-tolyl)propan-1-ol (9)

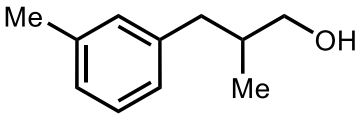

The title compound was prepared according to general procedure 7 using (*E*)-2-methyl-3-(*m*-tolyl)prop-2-en-1-ol (162 mg, 1 mmol) for 24 h and purified by flash silica chromatography (15-20% EtOAc in petroleum ether, 35 × 160 mm silica) to give the title compound as a colourless oil (116 mg, 71% isolated yield, 89% NMR yield); R_f = 0.38 (20% EtOAc in petroleum ether); ν_{max} / cm^{-1} (film) 3327, 2955, 2916, 2870, 1607, 1456, 1034, 777, 741, 698, 419; ^{1}H NMR (500 MHz, Chloroform-*d*) δ 7.18 (t, *J* = 7.5 Hz, 1H), 7.04 – 6.94 (m, 3H), 3.54 (dd, *J* = 10.6, 5.8 Hz, 1H), 3.48 (dd, *J* = 10.6, 6.0 Hz, 1H), 2.71 (dd, *J* = 13.4, 6.4 Hz, 1H), 2.44 – 2.34 (m, 1H), 2.33 (d, *J* = 0.8 Hz, 3H), 2.00 – 1.89 (m, 1H), 1.33 (bs, 1H), 0.92 (d, *J* = 6.8 Hz, 3H); ^{13}C{^{1}H} NMR (126 MHz, Chloroform-*d*) δ 140.7, 138.0, 130.1, 128.3, 126.8, 126.3, 67.9, 39.8, 38.0, 21.6, 16.7; HRMS (EI-quadrupole) $(M)^+$ Calcd for $C_{11}H_{16}O$ 164.1196; Found 164.1194.

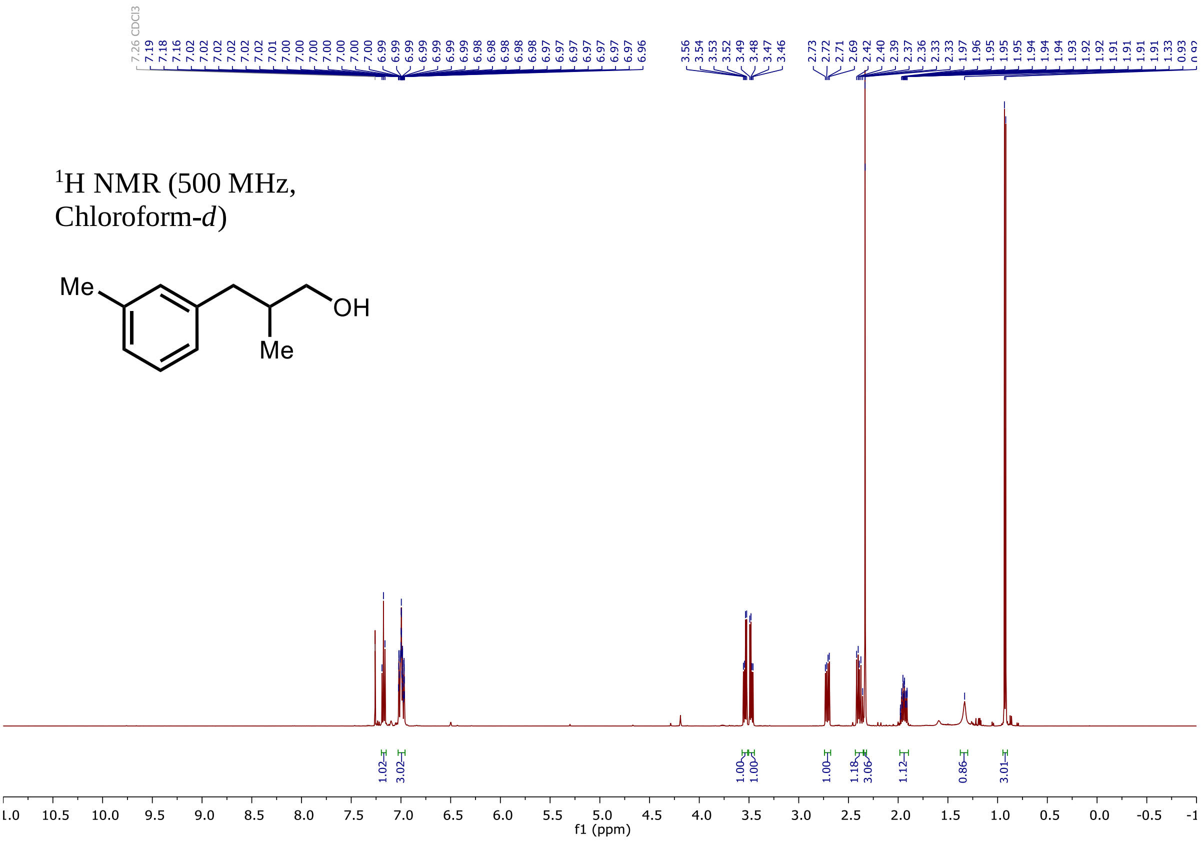

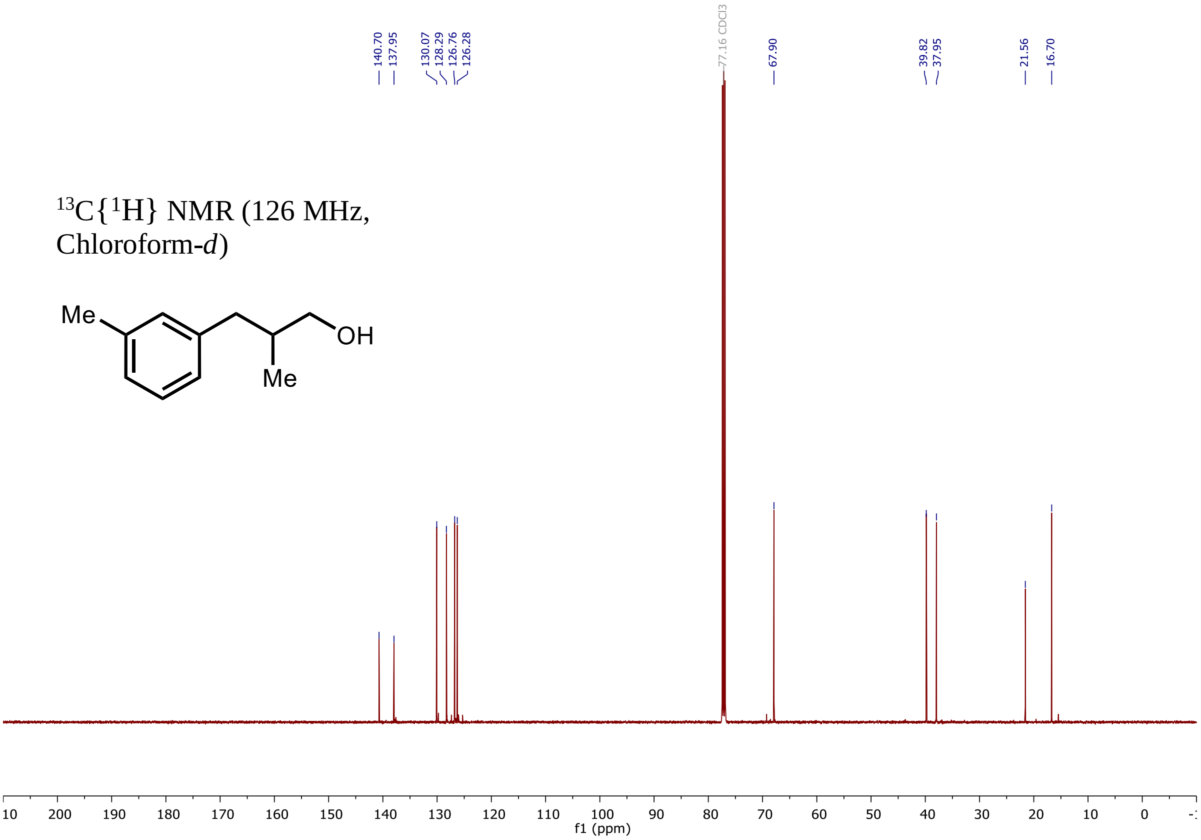

2-methyl-3-(*o*-tolyl)propan-1-ol (10)

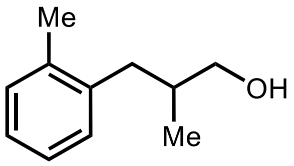

The title compound was prepared according to general procedure 7 using (*E*)-2-methyl-3-(*o*-tolyl)prop-2-en-1-ol (162 mg, 1 mmol) for 24 hand purified by flash silica chromatography (10-20% EtOAc in petroleum ether, 35 × 160 mm silica) to give the title compound as a colourless oil (135 mg, 82% isolated yield, 91% NMR yield); R_f = 0.30 (20% EtOAc in petroleum ether; ^{1}H NMR (500 MHz, Chloroform-*d*) δ 7.15 (dd, *J* = 7.7, 3.1 Hz, 1H), 7.13 – 7.09 (m, 3H), 3.57 (dd, *J* = 10.5, 5.7 Hz, 1H), 3.50 (dd, *J* = 10.5, 6.1 Hz, 1H), 2.77 (dd, *J* = 13.7, 6.5 Hz, 1H), 2.41 (dd, *J* = 13.7, 8.1 Hz, 1H), 2.32 (s, 3H), 1.93 (tt, *J* = 12.7, 6.4 Hz, 1H), 1.40 (s, 1H), 0.95 (d, *J* = 6.7 Hz, 3H); ^{13}C{^{1}H} NMR (126 MHz, Chloroform-*d*) δ 139.1, 136.4, 130.5, 130.1, 126.2, 125.9, 68.1, 37.5, 36.8, 19.2, 16.8. Spectroscopic data are in accordance with that stated in the literature.[17]

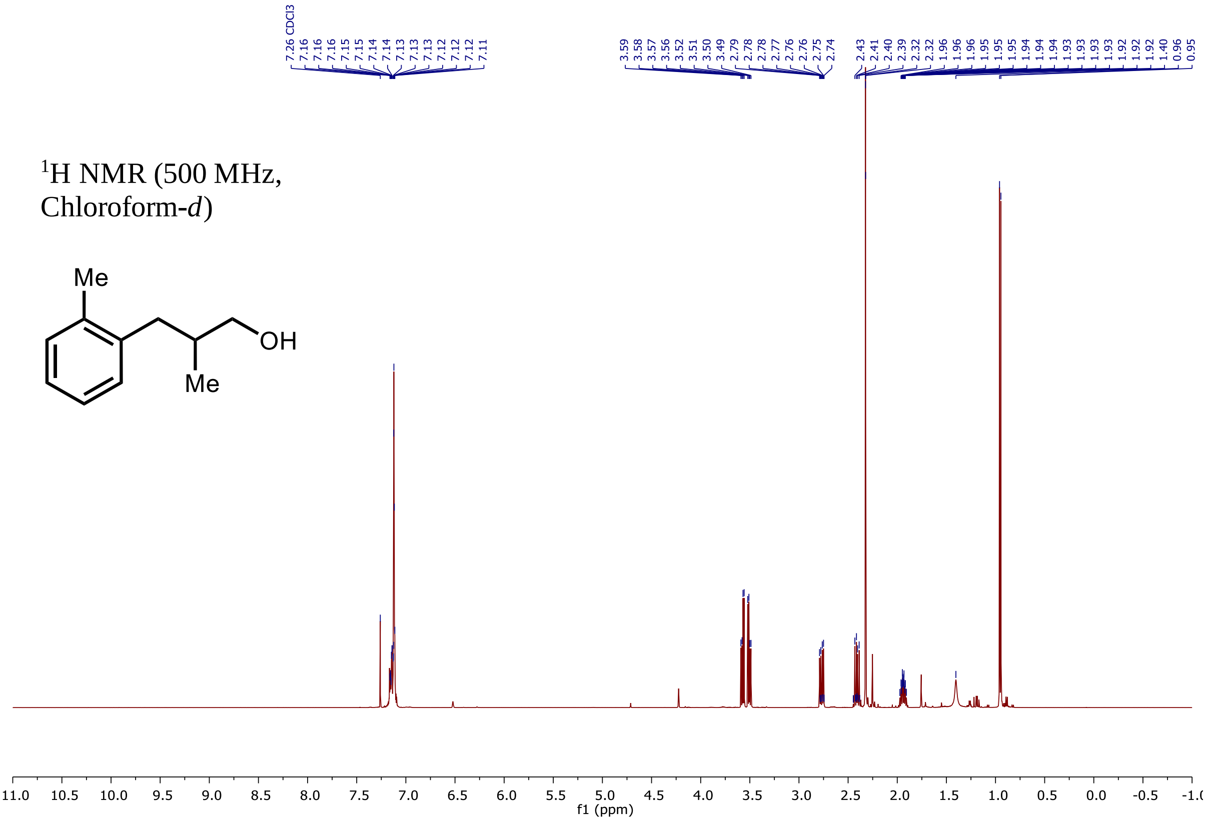
7.26 CDCl3
7.16 7.16 7.16 7.15 7.15 7.14 7.14 7.13 7.13 7.13 7.12 7.12 7.12 7.11
3.59 3.58 3.57 3.56 3.52 3.51 3.50 3.49 2.79 2.78 2.78 2.77 2.76 2.76 2.75 2.74
2.43 2.41 2.40 2.39 2.32 2.32 1.96 1.96 1.96 1.95 1.95 1.95 1.94 1.94 1.94 1.93 1.93 1.93 1.93 1.92 1.92 1.40 0.96 0.95
1H NMR (500 MHz, Chloroform-d)
Me
OH
Me
11.0 10.5 10.0 9.5 9.0 8.5 8.0 7.5 7.0 6.5 6.0 5.5 5.0 4.5 4.0 3.5 3.0 2.5 2.0 1.5 1.0 0.5 0.0 -0.5
f1 (ppm)

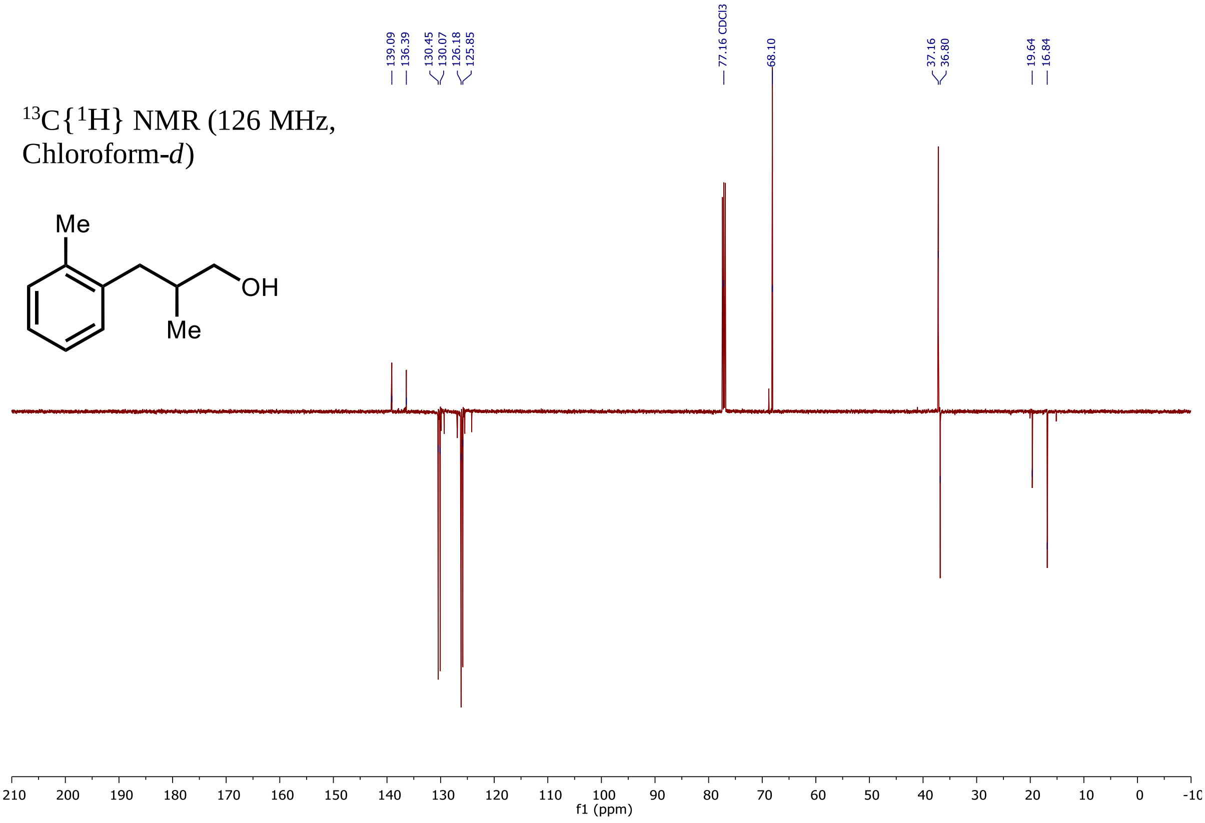
139.09
136.39
130.45
130.07
126.18
125.85
77.16 CDCl3
68.10
37.16
36.80
19.64
16.84
13C{1H} NMR (126 MHz, Chloroform-d)
Me
OH
Me
210 200 190 180 170 160 150 140 130 120 110 100 90 80 70 60 50 40 30 20 10 0
f1 (ppm)

3-(4-fluorophenyl)-2-methylpropan-1-ol (11)

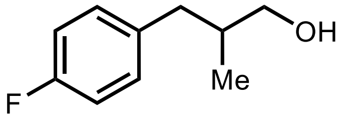

The title compound was prepared according to general procedure 7 using (*E*)-3-(4-fluorophenyl)-2-methylprop-2-en-1-ol (166 mg, 1 mmol) for 24 h and purified by flash silica chromatography (15-20% EtOAc in petroleum ether, 35 × 160 mm silica) to give the title compound as a yellow oil (102 mg, 61% isolated yield, 92% NMR yield); ν_{max} / cm^{-1} (film) 3337, 2957, 2922, 2874, 1601, 1508, 1456, 1219, 1157, 1032, 986, 841, 808, 762, 557, 503; R_f = 0.58 (20% EtOAc in petroleum ether); ^{1}H NMR (500 MHz, Chloroform-*d*) δ 7.14 – 7.09 (m, 2H), 6.99 – 6.93 (m, 2H), 3.54 – 3.42 (m, 2H), 2.74 (dd, *J* = 13.4, 6.4 Hz, 1H), 2.39 (dd, *J* = 13.6, 8.1 Hz, 1H), 1.94 – 1.86 (m, 1H), 1.64 (s, 1H), 0.90 (d, *J* = 6.8 Hz, 3H); ^{13}C{^{1}H} NMR (126 MHz, Chloroform-*d*) δ 161.4 (d, $J_{C\text{-}F}$ = 243.4 Hz), 136.3 (d, $J_{C\text{-}F}$ = 3.2 Hz), 130.6 (d, $J_{C\text{-}F}$ = 7.7 Hz), 115.1 (d, $J_{C\text{-}F}$ = 21.1 Hz), 67.5, 38.9, 37.9 (d, $J_{C\text{-}F}$ = 1.0 Hz), 16.4; ^{19}F{^{1}H} NMR (471 MHz, Chloroform-*d*) δ -117.7; HRMS (EI-quadrupole) $(M)^{+}$ Calcd for $C_{10}H_{13}OF$ 168.0945; Found 168.0943.

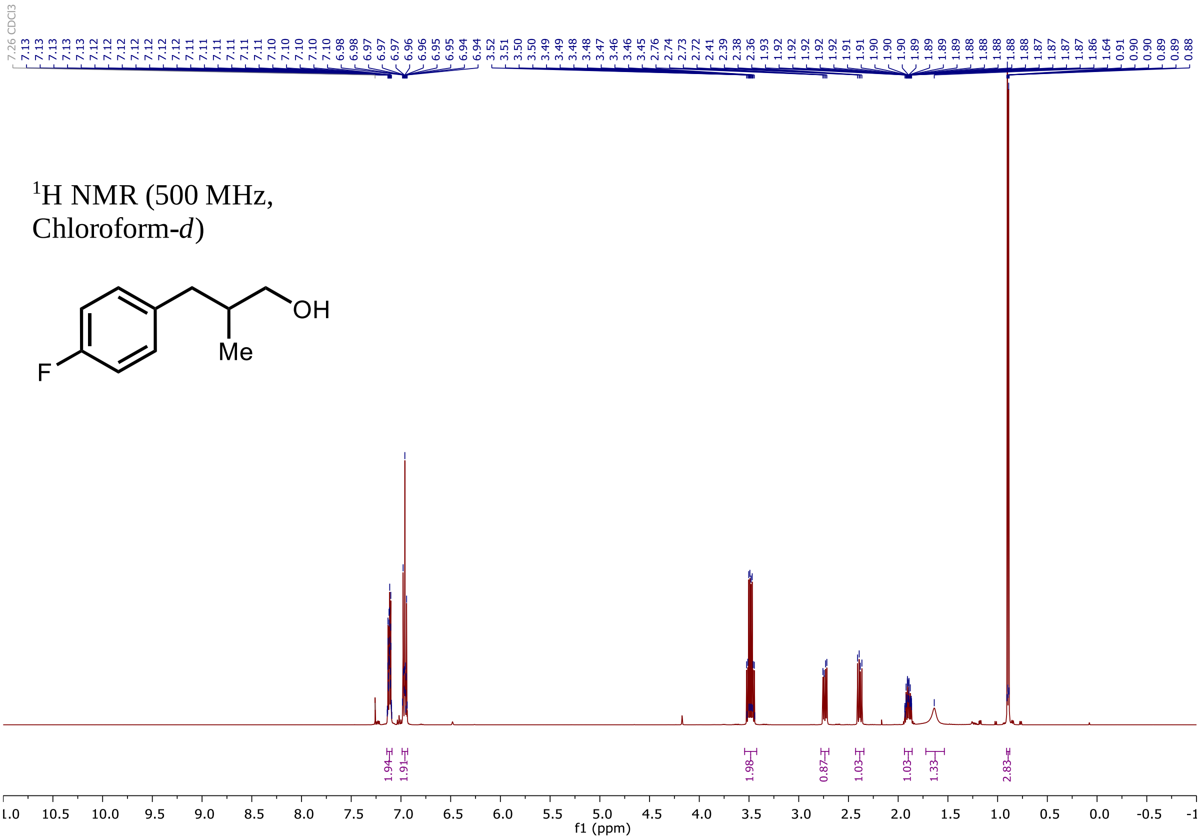

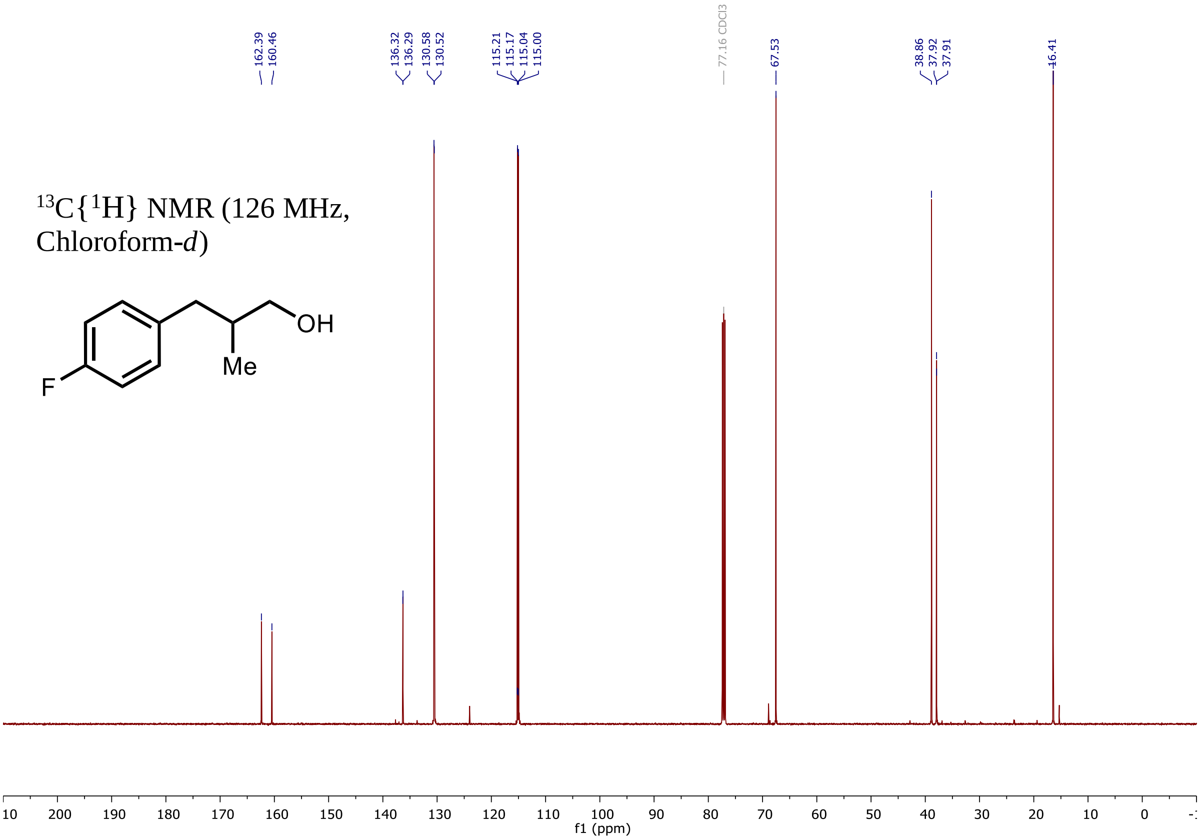

$^{13}C\{^1H\}$ NMR (126 MHz, Chloroform-*d*)
OH
Me
F
162.39
160.46
136.32
136.29
130.58
130.52
115.21
115.17
115.04
115.00
77.16 CDCl3
67.53
38.86
37.92
37.91
16.41
f1 (ppm)

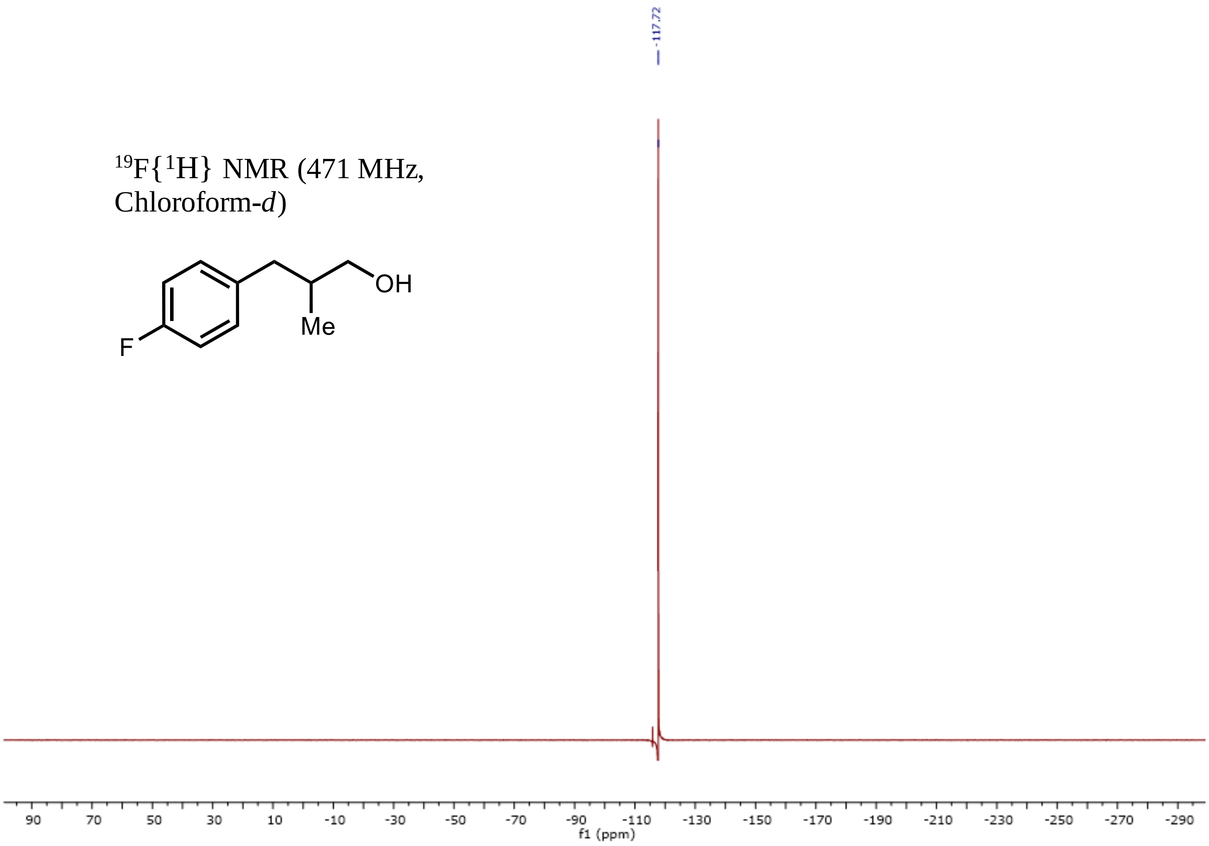

$^{19}F\{^1H\}$ NMR (471 MHz, Chloroform-*d*)
OH
Me
F
-117.72
f1 (ppm)

3-(4-chlorophenyl)-2-methylpropan-1-ol (12)

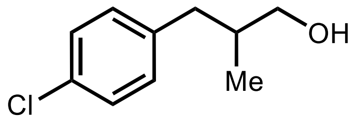

The title compound was prepared according to general procedure 7 using (*E*)-3-(4-chlorophenyl)-2-methylprop-2-en-1-ol (182 mg, 1 mmol) for 24 h and purified by flash silica chromatography (15-20% EtOAc in petroleum ether, 35 × 160 mm silica) to give the title compound as a colourless oil (140 mg, 76% isolated yield, 90% NMR yield); R_f = 0.37 (20% EtOAc in petroleum ether); v_{max} / cm^{-1} (film) 3335, 2980, 2928, 2883, 1491, 1456, 1406, 1088, 1032, 1006, 841, 791, 661, 548, 517; ^{1}H NMR (500 MHz, Chloroform-*d*) δ 7.27 – 7.21 (m, 2H), 7.13 – 7.06 (m, 2H), 3.55 – 3.44 (m, 2H), 2.74 (dd, *J* = 13.5, 6.2 Hz, 1H), 2.39 (dd, *J* = 13.5, 8.2 Hz, 1H), 1.97 – 1.84 (m, 1H), 1.47 (bs, 1H), 0.90 (d, *J* = 6.8 Hz, 3H); ^{13}C{^{1}H} NMR (126 MHz, Chloroform-*d*) δ 139.2, 131.8, 130.6, 128.5, 67.5, 39.1, 37.8, 16.4; HRMS (EI-quadrupole) $(M)^+$ Calcd for $C_{10}H_{13}O^{35}Cl$ 184.0649; Found 184.0643.

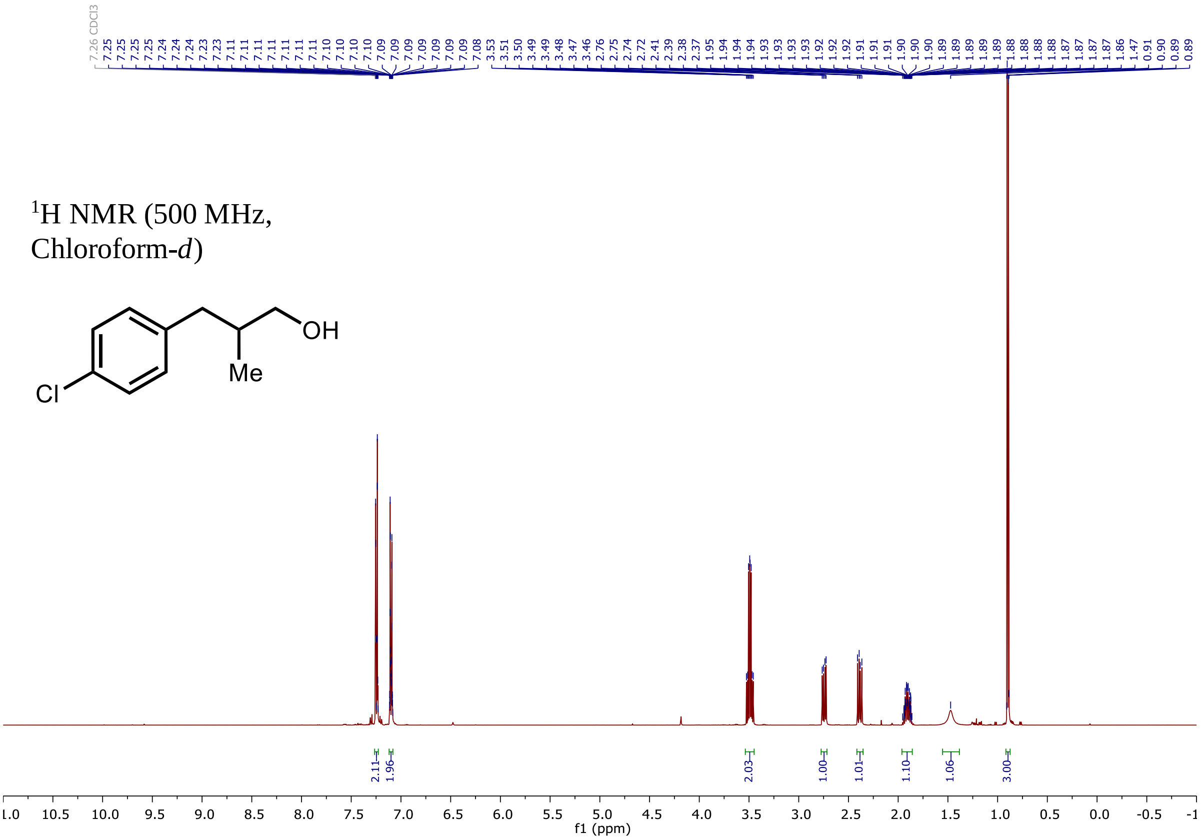

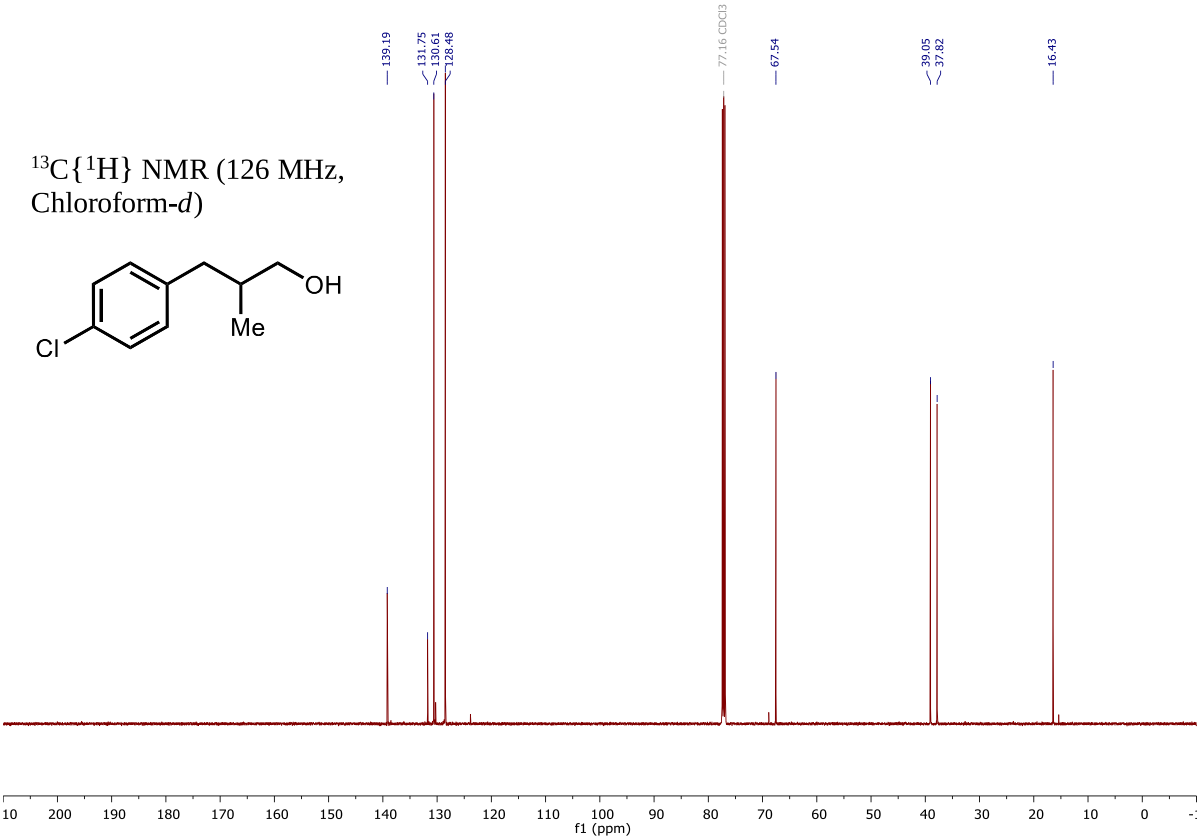

3-(4-bromophenyl)-2-methylpropan-1-ol (13)

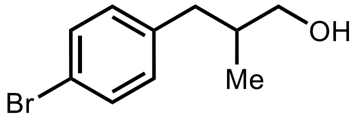

The title compound was prepared according to general procedure 7 using (*E*)-3-(4-bromophenyl)-2-methylprop-2-en-1-ol (227 mg, 1 mmol) for 48 h. Yield determined by crude ^{1}H NMR using 1,3,5-trimethylbenzene as internal standard: 33%

Resolved signals of 3-(4-bromophenyl)-2-methylpropan-1-ol:[18]

^{1}H NMR (300 MHz, Chloroform-*d*) δ 7.37 (d, *J* = 7.9 Hz, 2H), 7.02 (d, *J* = 8.0 Hz, 2H), 3.52 – 3.46 (m, 2H), 2.79 – 2.64 (m, 1H), 0.89 – 0.83 (t, *J* = 6.8, 3H).

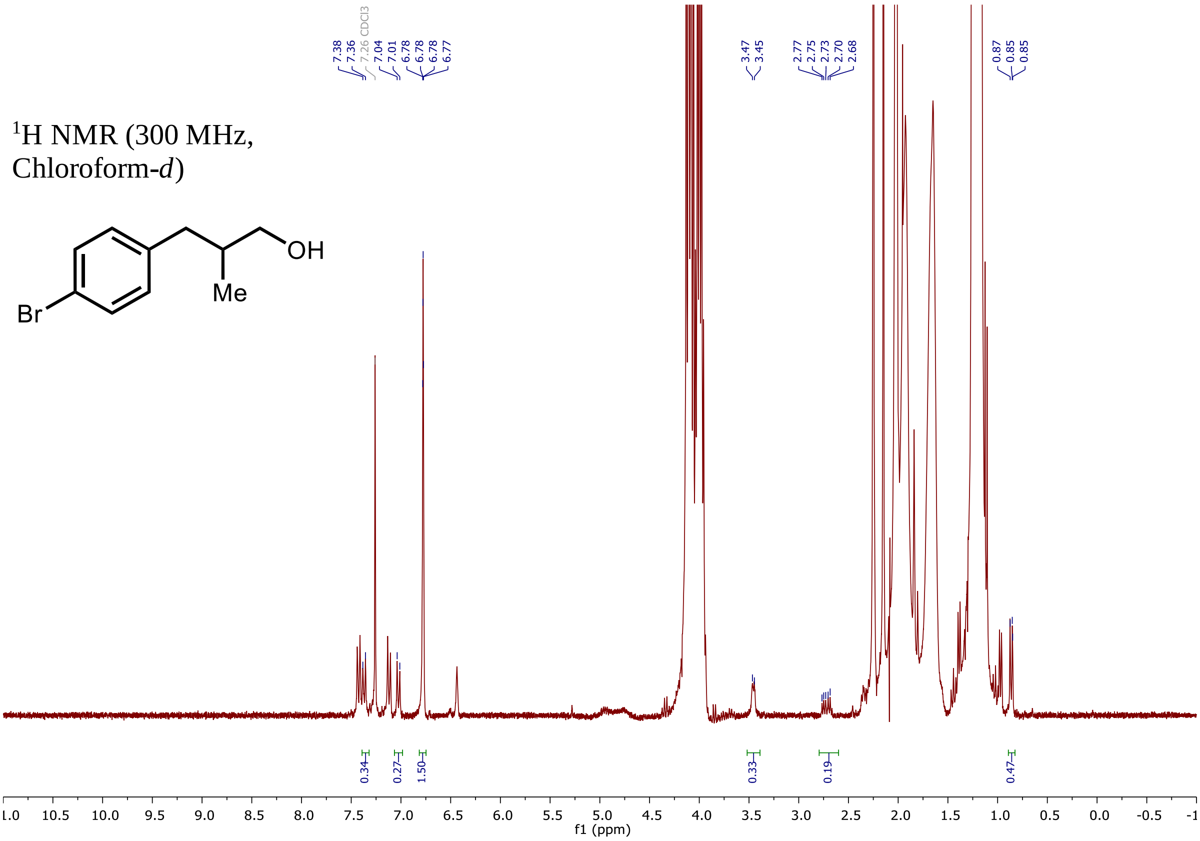

3-(4-methoxyphenyl)-2-methylpropan-1-ol (14)

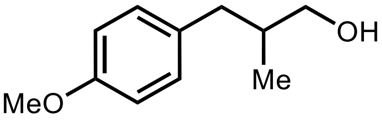

The title compound was prepared according to general procedure 7 using (*E*)-3-(4-methoxyphenyl)-2-methylprop-2-en-1-ol (178 mg, 1 mmol) for 24 h and purified by flash silica chromatography (5-10% EtOAc in petroleum ether, 35 × 160 mm silica) to give the title compound as a white solid (130 mg, 72% isolated yield, 88% NMR yield); mp 39-40 °C; R_f = 0.35 (20% EtOAc in petroleum ether); ^{1}H NMR (500 MHz, Chloroform-*d*) δ 7.09 (d, *J* = 8.3 Hz, 2H), 6.83 (d, *J* = 8.6 Hz, 2H), 3.79 (s, 3H), 3.53 (dd, *J* = 10.6, 5.9 Hz, 1H), 3.47 (dd, *J* = 10.5, 6.0 Hz, 1H), 2.69 (dd, *J* = 13.6, 6.4 Hz, 1H), 2.38 (dd, *J* = 13.6, 7.9 Hz, 1H), 1.90 (dq, *J* = 13.0, 6.5 Hz, 1H), 1.29 (s, 1H), 0.91 (d, *J* = 6.7 Hz, 3H);^{13}C{^{1}H} NMR (126 MHz, Chloroform-*d*) δ 158.0, 132.8, 130.2, 113.8, 67.8, 55.4, 38.9, 38.0, 16.6. Spectroscopic data are in accordance with that stated in the literature.[19]

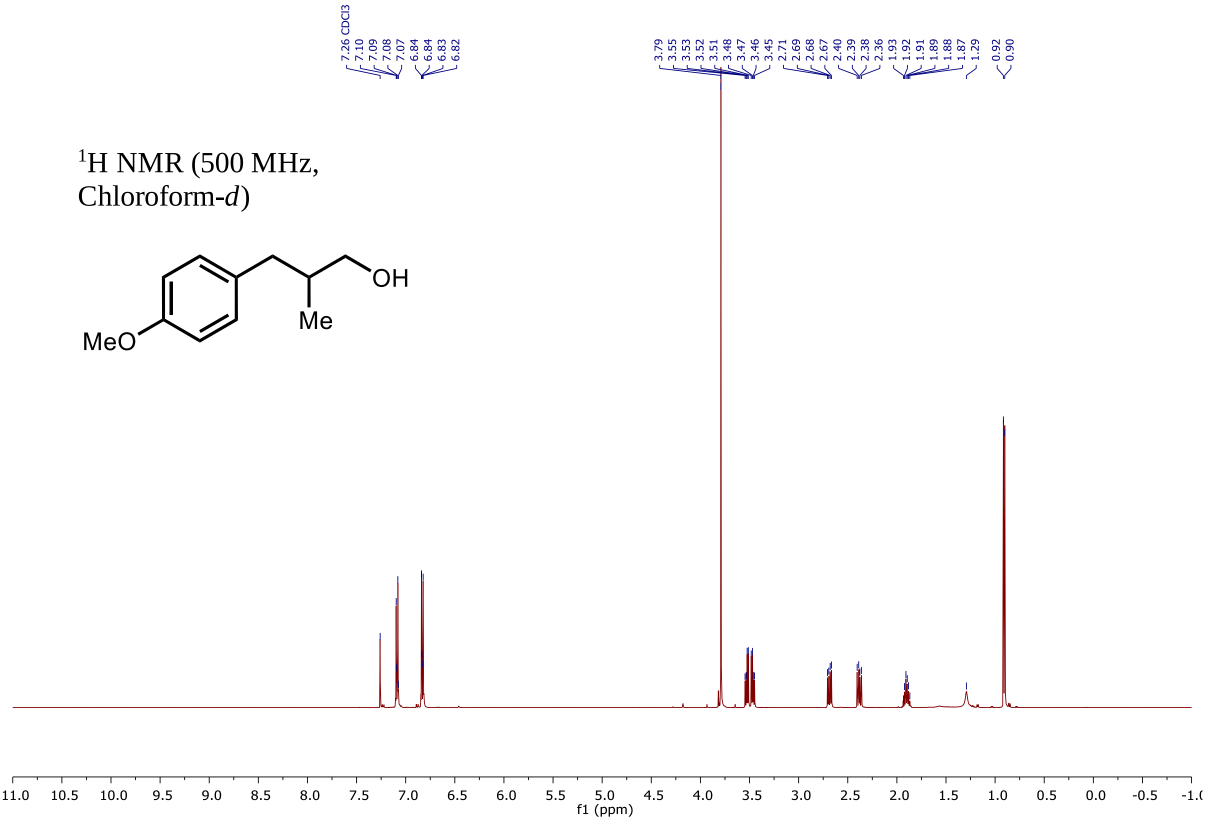

7.26 CDCl3
7.10
7.09
7.08
7.07
6.84
6.84
6.83
6.82
3.79
3.55
3.53
3.53
3.52
3.51
3.48
3.47
3.46
3.45
2.71
2.69
2.68
2.67
2.40
2.39
2.38
2.36
1.93
1.92
1.91
1.89
1.88
1.87
1.29
0.92
0.90
1H NMR (500 MHz, Chloroform-d)
OH
Me
MeO
f1 (ppm)

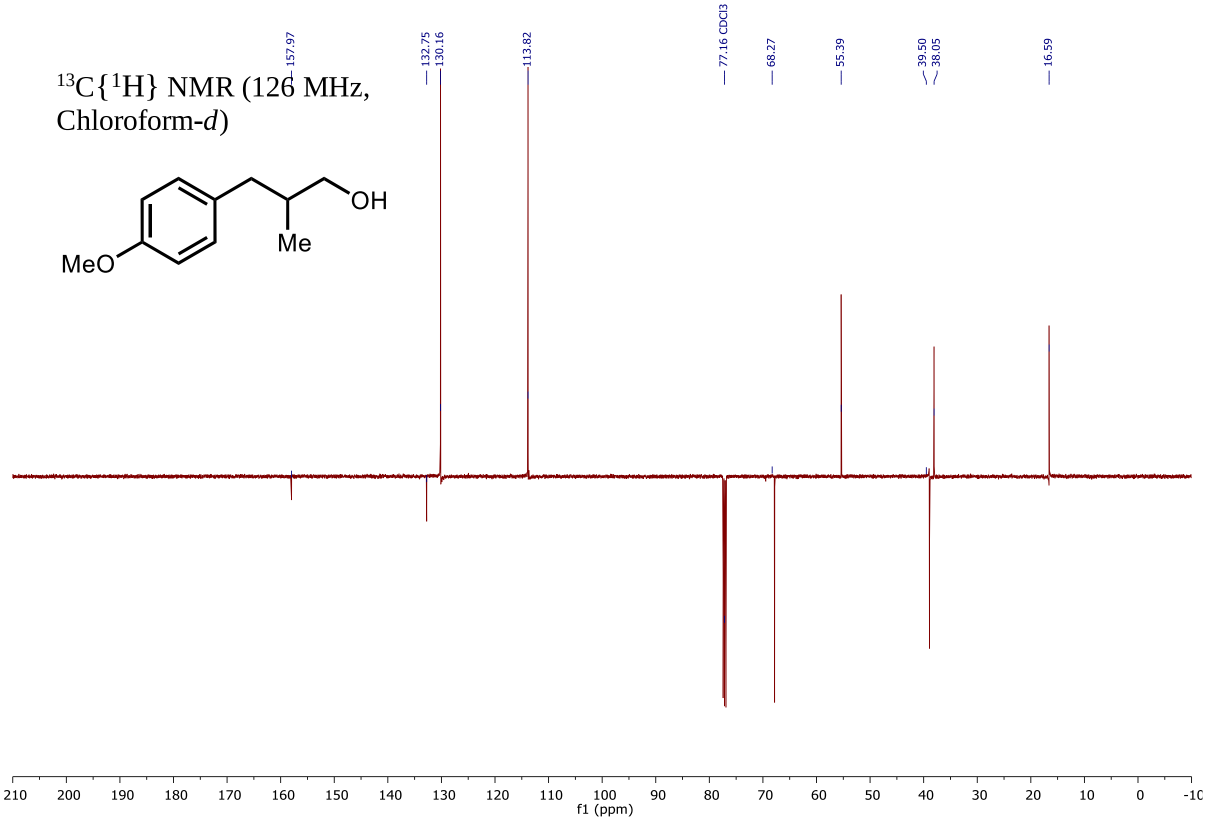

157.97
132.75
130.16
113.82
77.16 CDCl3
68.27
55.39
39.50
38.05
16.59
13C{1H} NMR (126 MHz, Chloroform-d)
OH
Me
MeO
f1 (ppm)

3-(4-(dimethylamino)phenyl)-2-methylpropan-1-ol (15)

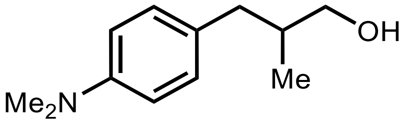

The title compound was prepared according to general procedure 7 using (*E*)-3-(4-(dimethylamino)phenyl)-2-methylprop-2-en-1-ol (191.27 mg, 1 mmol) for 48 h and purified by flash silica chromatography (20% EtOAc in petroleum ether, 35 × 160 mm silica) to give the title compound as a yellowish brown oil (156 mg, 80% isolated yield, 94% NMR yield); R_f = 0.30 (20% EtOAc in petroleum ether); ν_{max} / cm^{-1} (film) 3369, 2951, 2908, 2870, 2796, 1612, 1517, 1342, 1224, 1192, 1161, 1130, 1031, 983, 945, 821, 794, 732, 700, 559, 516, 451,437, 420, 408; ^1H NMR (500 MHz, Chloroform-*d*) δ 7.05 (d, *J* = 8.8 Hz, 2H), 6.69 (d, *J* = 8.6 Hz, 2H), 3.58 – 3.42 (m, 2H), 2.92 (s, 6H), 2.63 (dd, *J* = 13.6, 6.5 Hz, 1H), 2.36 (dd, *J* = 13.6, 7.8 Hz, 1H), 1.90 (dddd, *J* = 12.7, 7.8, 6.8, 6.0 Hz, 1H), 1.24 (s, 1H), 0.91 (d, *J* = 6.7 Hz, 3H); $^{13}C\{^1H\}$ NMR (126 MHz, Chloroform-*d*) δ 149.2, 129.9, 128.8, 113.0, 68.0, 41.0, 38.9, 38.1, 16.7; HRMS (CI-quadrupole) $(M)^+$ Calcd for $C_{12}H_{19}NO$ 193.1461; Found 193.1462.

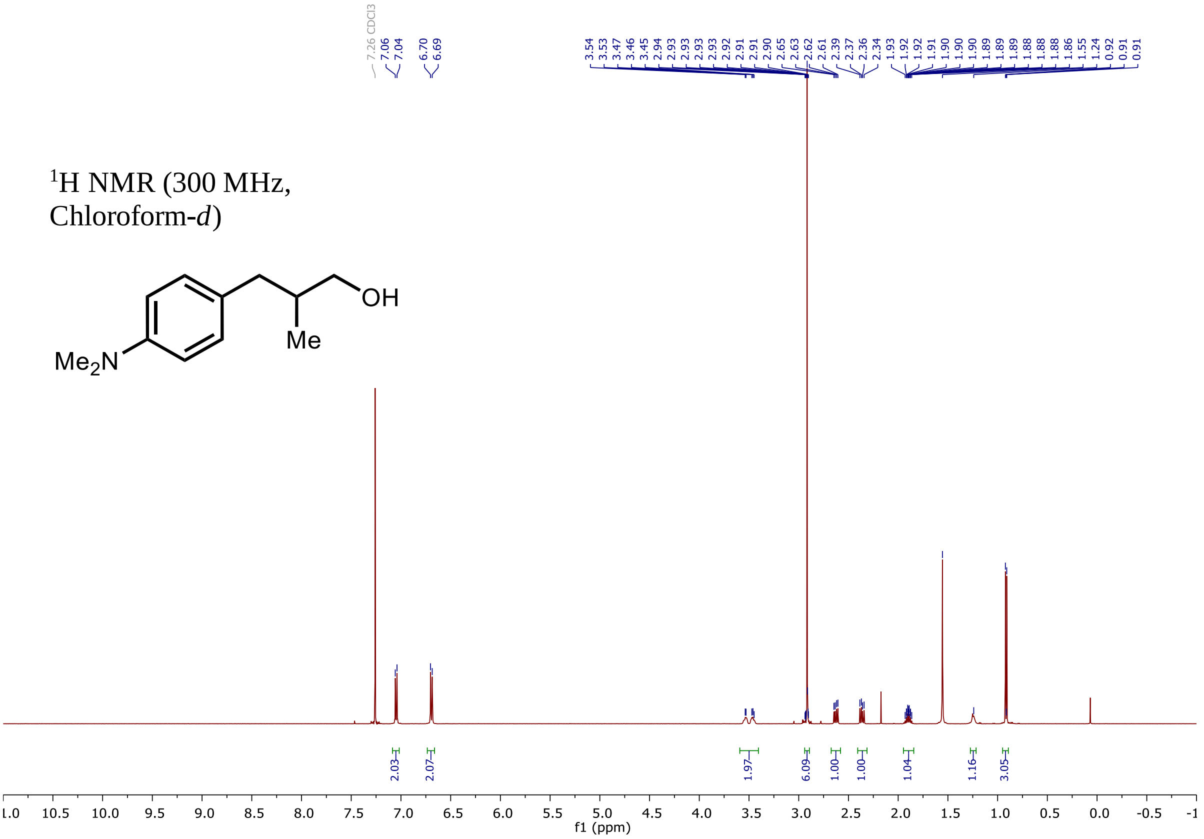

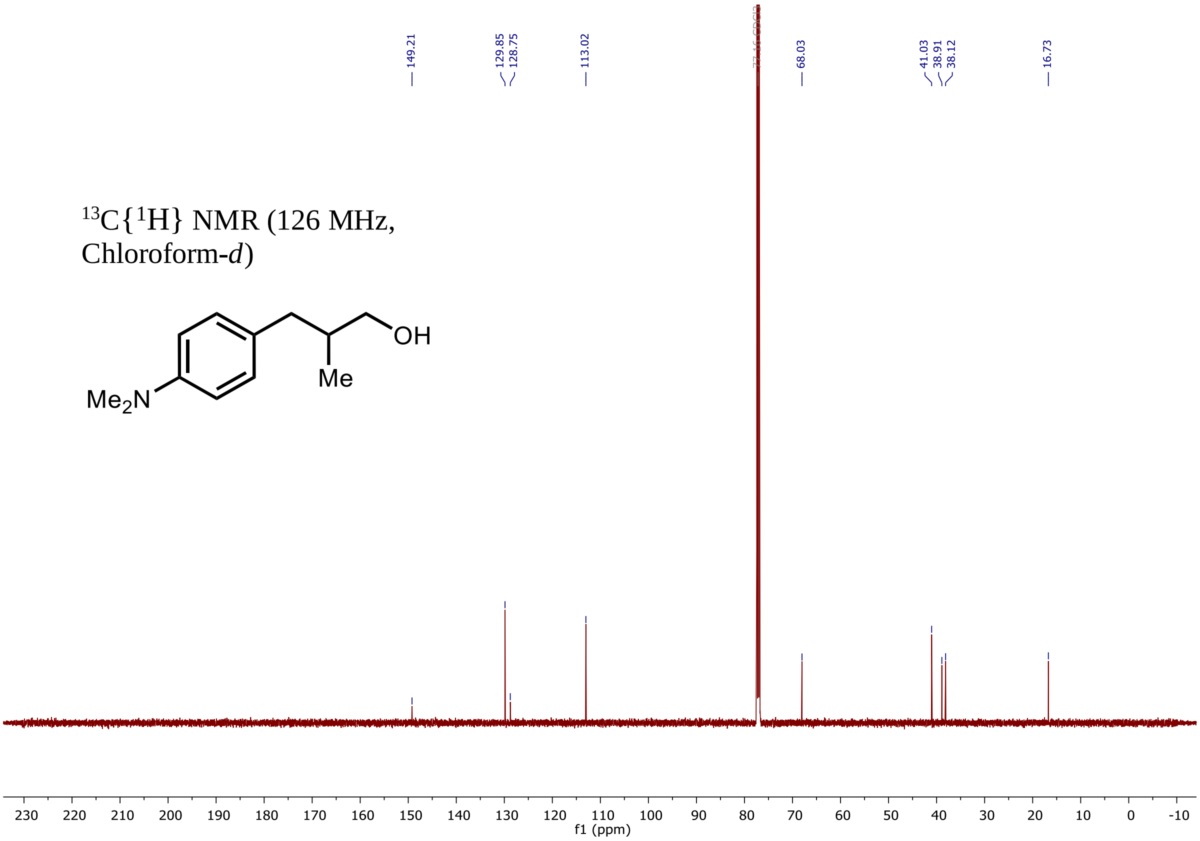

2-methyl-3-(4-(trifluoromethyl)phenyl)propan-1-ol (16)

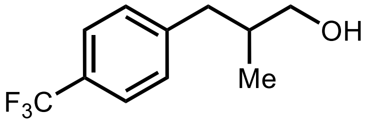

The title compound was prepared according to general procedure 7 using (*E*)-2-methyl-3-(4-(trifluoromethyl)phenyl)propan-1-ol (202 mg, 1 mmol) for 48 h. Yield determined by crude ^{1}H NMR using 1,3,5-trimethylbenzene as internal standard: 62%

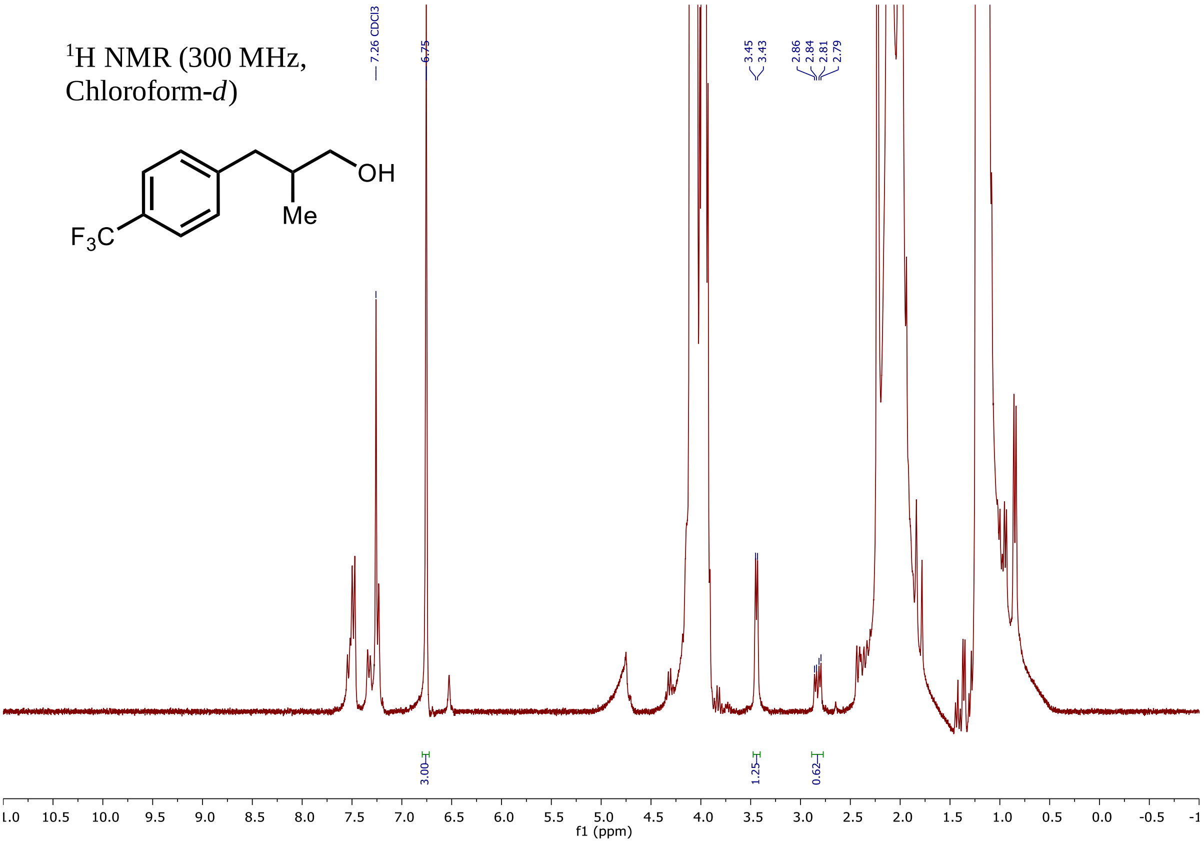

3-(4-(hydroxymethyl)phenyl)-2-methylpropan-1-ol (17)

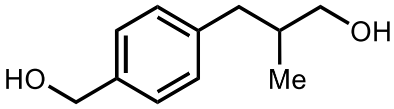

The title compound was prepared according to general procedure 7 using (*E*)-3-(4-(hydroxymethyl)phenyl)-2-methylprop-2-en-1-ol (178 mg, 1 mmol) for 24 h and purified by flash silica chromatography (10% EtOAc in petroleum ether, 35 × 160 mm silica) to give the title compound as a clear oil (127 mg, 71% isolated yield, 84% NMR yield); R_f = 0.25 (30% EtOAc in petroleum ether); ν_{max} / cm^{-1} (film) 3306, 2917, 2864, 1411, 1031, 849, 686; ^{1}H NMR (500 MHz, Chloroform-*d*) δ 7.24 (d, *J* = 8.1 Hz, 2H), 7.12 (d, *J* = 8.0 Hz, 2H), 4.59 (s, 2H), 3.46 (dd, *J* = 10.6, 6.0 Hz, 1H), 3.41 (dd, *J* = 10.6, 6.1 Hz, 1H), 2.72 (dd, *J* = 13.5, 6.1 Hz, 1H), 2.36 (dd, *J* = 13.5, 8.2 Hz, 1H), 2.25 (s, 1H), 1.92 – 1.84 (m, 1H), 0.86 (d, *J* = 6.8 Hz, 3H); ^{13}C{^{1}H} NMR (126 MHz, Chloroform-*d*) δ 140.1, 138.5, 129.4, 127.1, 67.5, 65.0, 39.4, 37.8, 16.5; HRMS (ES-TOF) $(M)^+$ Calcd for $C_{11}H_{16}O_2$ 180.1145; Found 180.1140.

^{1}H NMR (300 MHz, Chloroform-*d*)

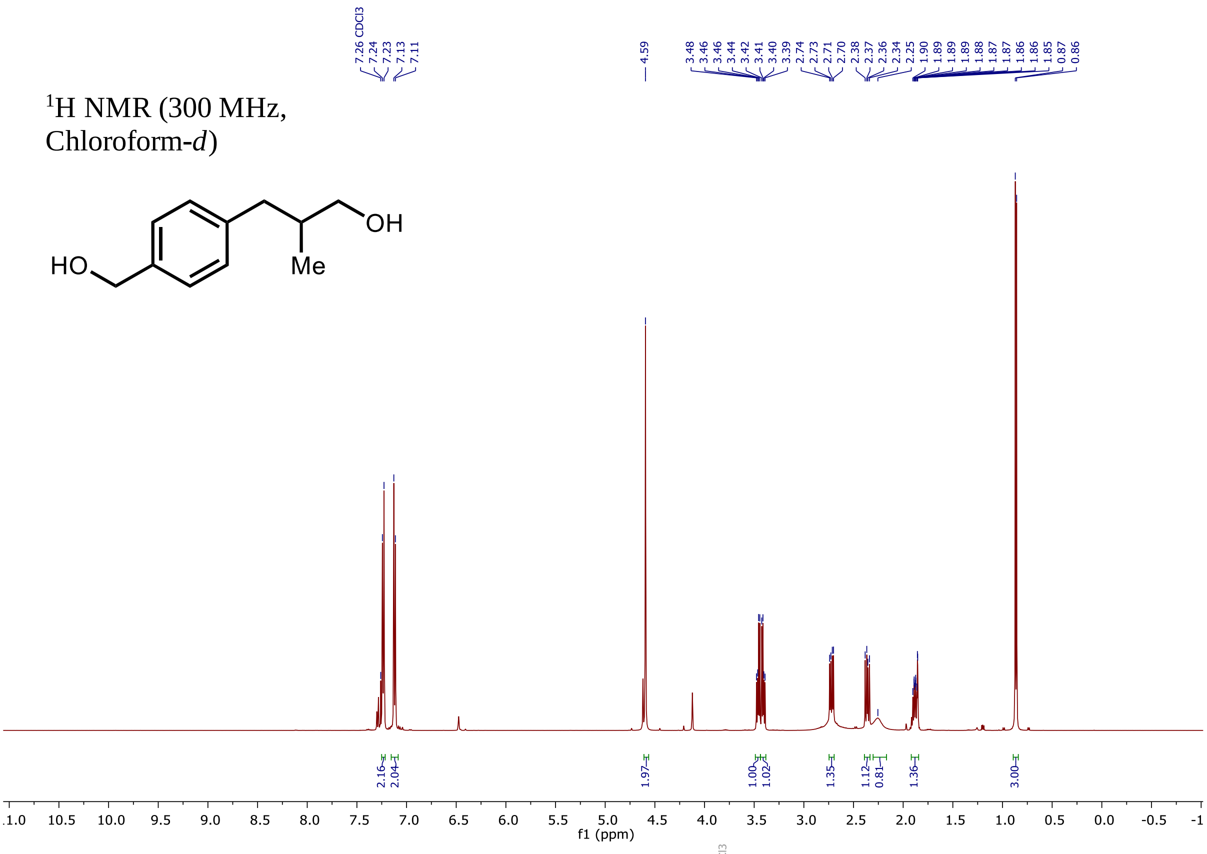

^{13}C{^{1}H} NMR (75 MHz, Chloroform-*d*)

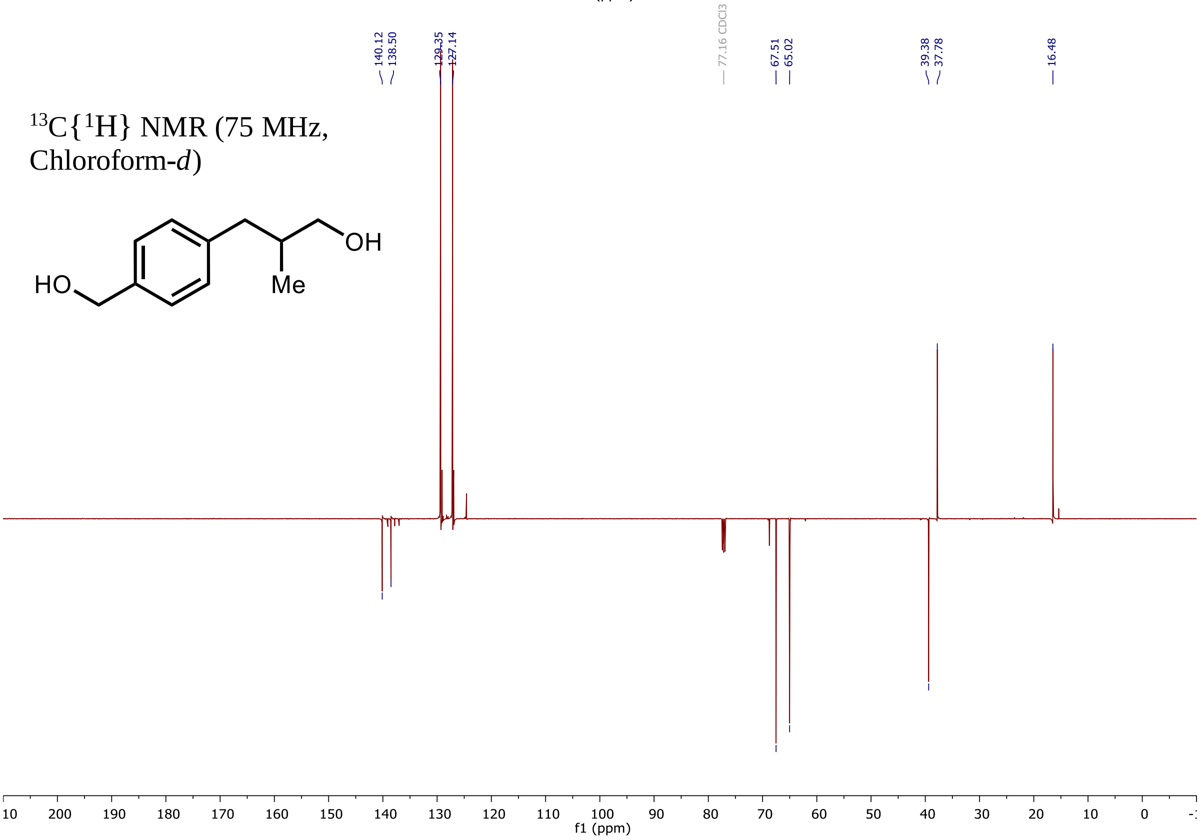

2-methyl-3-(4-vinylphenyl)propan-1-ol (18)

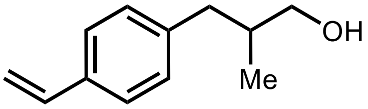

The title compound was prepared according to general procedure 7 using (*E*)-2-methyl-3-(4-vinylphenyl)prop-2-en-1-ol (174 mg, 1 mmol) for 48 h. Yield determined by crude ^{1}H NMR using 1,3,5-trimethylbenzene as internal standard: 71%

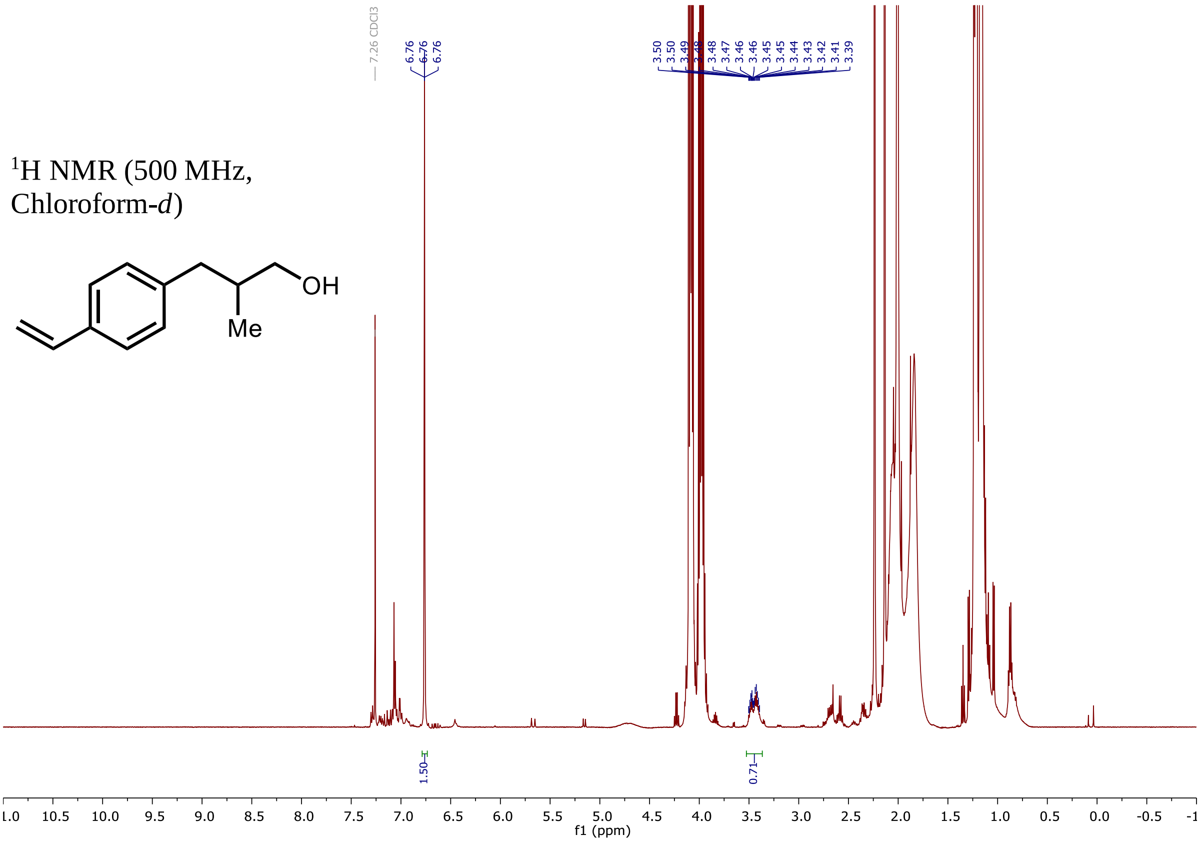

2-methyl-3-(naphthalen-1-yl)propan-1-ol (19)

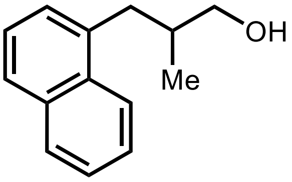

The title compound was prepared according to general procedure 7 using (*E*)-2-methyl-3-(naphthalen-1-yl)prop-2-en-1-ol (198 mg, 1 mmol) for 24 h and purified by flash silica chromatography (10% EtOAc in petroleum ether, 35 × 160 mm silica) to give the title compound as a colourless oil (139 mg, 69% isolated yield, 71 % NMR yield); R_f = 0.45 (10% EtOAc in petroleum ether)); ν_{max} / cm^{-1} (film) 3348, 3057, 2927, 2926, 2868, 1595, 1508, 1458, 1394, 1259,1165, 1072, 983, 910, 864, 788, 773, 732, 597, 553, 489, 428, 406; ^{1}H NMR

(500 MHz, Chloroform-*d*) δ 8.07 (dd, *J* = 8.3, 1.3 Hz, 1H), 7.86 (dd, *J* = 7.9, 1.7 Hz, 1H), 7.73 (d, *J* = 8.2 Hz, 1H), 7.53 – 7.45 (m, 2H), 7.40 (dd, *J* = 8.2, 7.0 Hz, 1H), 7.33 – 7.30 (m, 1H), 3.67 – 3.52 (m, 2H), 3.31 (dd, *J* = 13.7, 6.2 Hz, 1H), 2.80 (dd, *J* = 13.7, 8.1 Hz, 1H), 2.20 – 2.09 (m, 1H), 1.37 (s, 1H), 0.98 (d, *J* = 6.7 Hz, 3H); $^{13}C\{^1H\}$ NMR (126 MHz, Chloroform-*d*) δ 137.0, 134.1, 132.2, 128.9, 127.3, 126.9, 125.9, 125.6, 125.5, 124.2, 68.2, 37.2, 37.1, 17.1; HRMS (CI-quadrupole) $(M)^+$ Calcd for $C_{14}H_{16}O$ 200.1196; Found 200.1197.

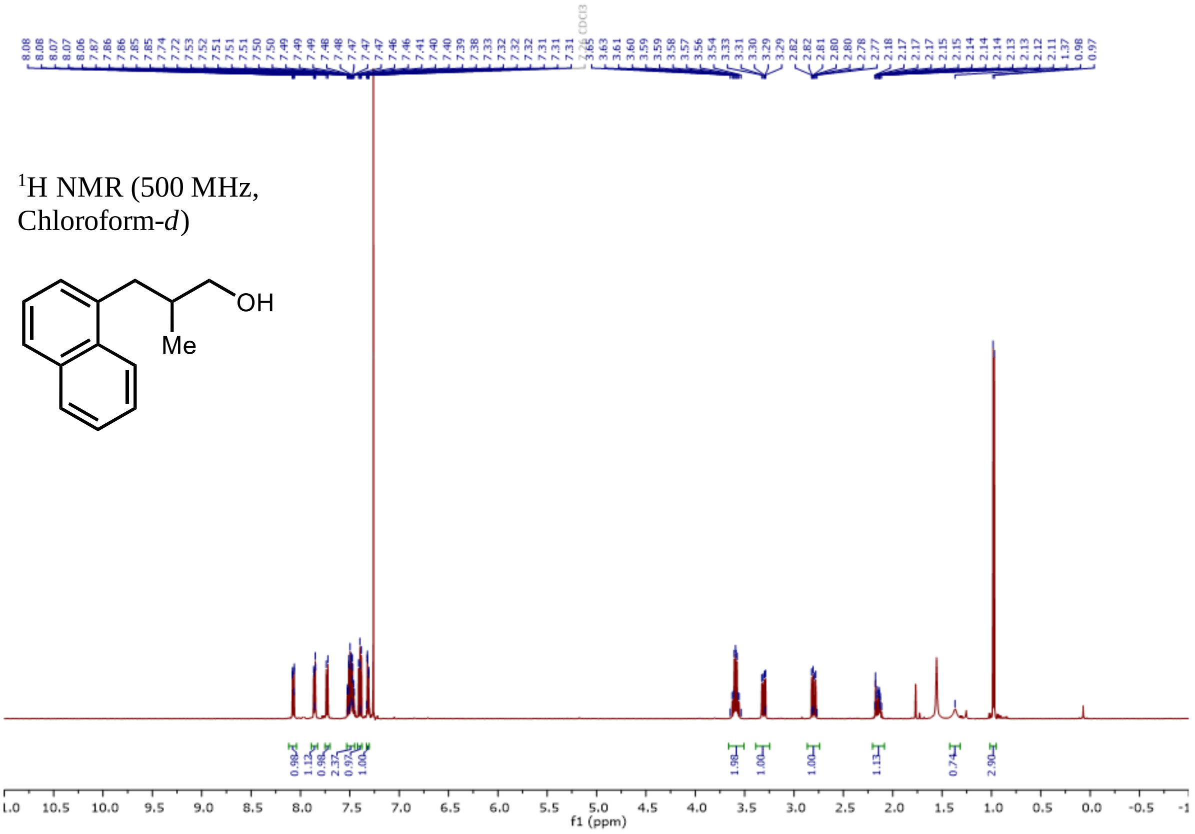

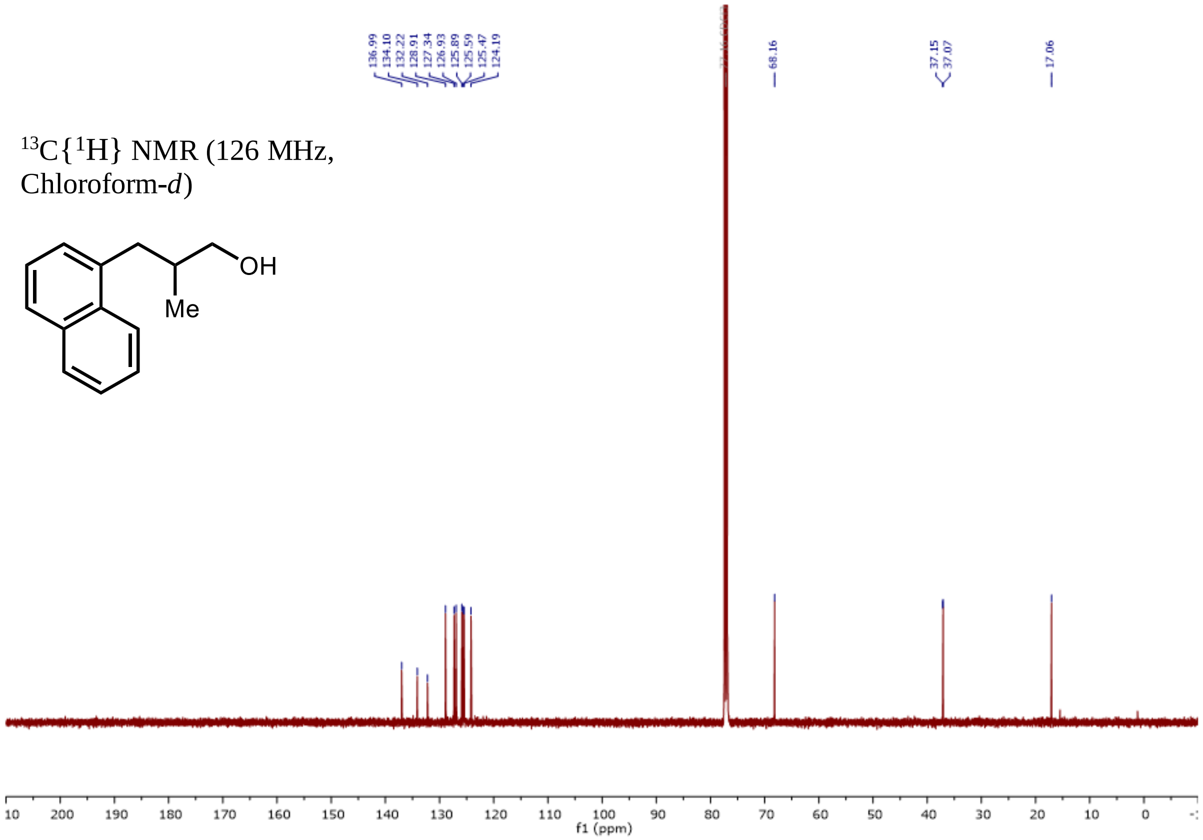

2-methyl-3-(naphthalen-2-yl)propan-1-ol (20)

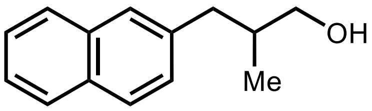

The title compound was prepared according to general procedure 7 using (*E*)-2-methyl-3-(naphthalen-2-yl)prop-2-en-1-ol (198 mg, 1 mmol) for 24 h and purified by flash silica chromatography (20% EtOAc in petroleum ether, 35 × 160 mm silica) to give the title compound as a colourless oil (102 mg, 51% isolated yield, 89% NMR yield); R_f = 0.55 (20% EtOAc in petroleum ether); ν_{max} / cm^{-1} (film) 3298, 3053, 3014, 2914, 2854, 1595, 1504, 1363, 1271, 1192, 1170, 1155, 1124, 1068, 1012, 987, 893, 813, 773, 729, 648, 621, 555, 503, 478, 426, 406; ^{1}H NMR (500 MHz, Chloroform-*d*) δ 7.85 – 7.73 (m, 3H), 7.62 (s, 1H), 7.49 – 7.39 (m, 2H), 7.33 (dt, J = 8.4, 1.9 Hz, 1H), 3.61 – 3.47 (m, 2H), 2.93 (ddd, J = 13.5, 6.3, 1.9 Hz, 1H), 2.64 – 2.60 (m, 1H), 2.12 – 2.05 (m, 1H), 1.45 (bs, 1H), 0.96 (dd, J = 6.8, 1.9 Hz, 3H); ^{13}C{^{1}H} NMR (126 MHz, Chloroform-*d*) δ 138.3, 133.7, 132.2, 127.97, 127.95, 127.8, 127.6, 127.5, 67.8, 40.0, 37.9, 16.7; HRMS (EI-quadrupole) $(M)^+$ Calcd for $C_{14}H_{16}O$ 200.1196; Found 200.1192.

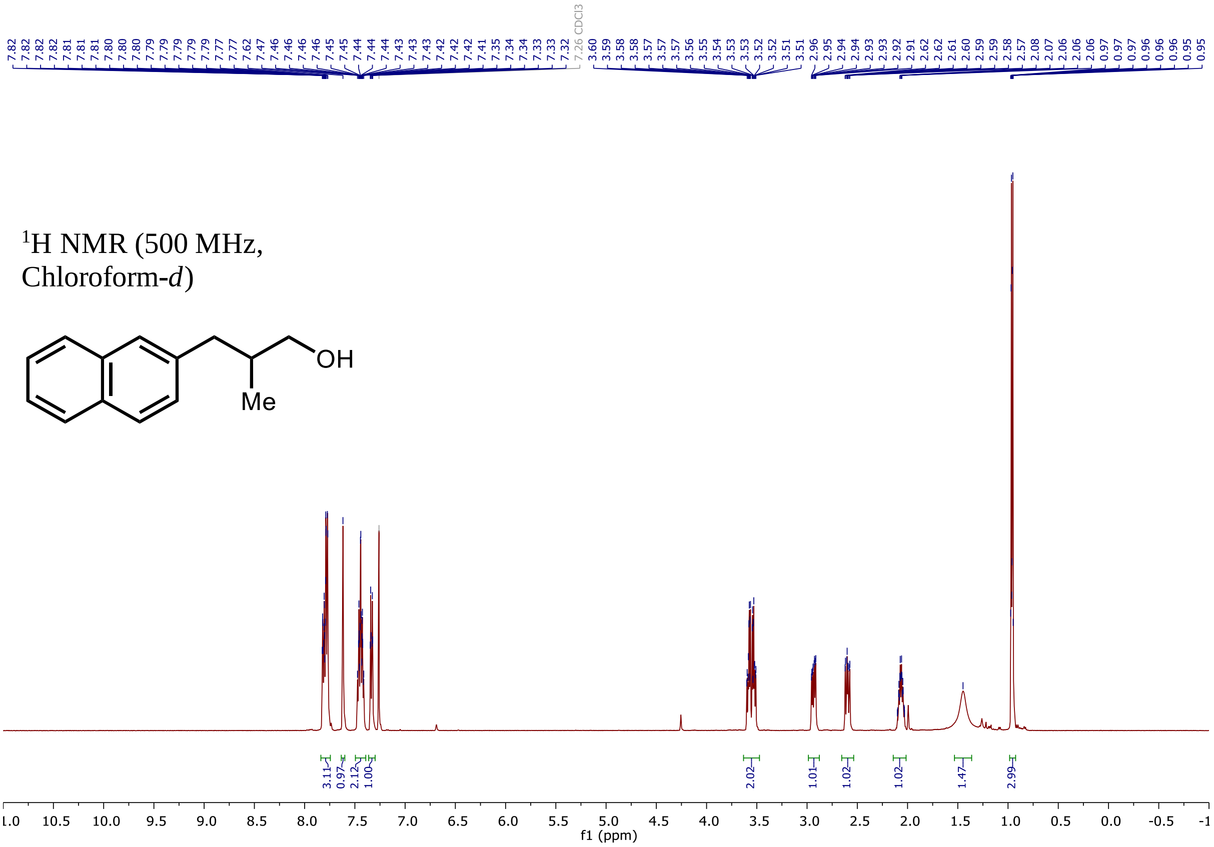
7.82 7.82 7.82 7.81 7.81 7.81 7.80 7.80 7.79 7.79 7.79 7.79 7.79 7.77 7.62 7.47 7.46 7.46 7.46 7.45 7.45 7.44 7.44 7.43 7.43 7.43 7.42 7.42 7.41 7.35 7.34 7.34 7.33 7.32 7.26 CDCl3 3.60 3.59 3.58 3.58 3.57 3.57 3.56 3.55 3.54 3.53 3.53 3.52 3.52 3.51 3.51 2.96 2.95 2.94 2.94 2.93 2.93 2.92 2.91 2.62 2.62 2.61 2.60 2.59 2.59 2.58 2.57 2.08 2.07 2.06 2.06 2.06 0.97 0.97 0.97 0.96 0.96 0.96 0.95 0.95
¹H NMR (500 MHz, Chloroform-d)
OH
Me
3.11 0.97 2.12 1.00 2.02 1.01 1.02 1.02 1.47 2.99
f1 (ppm)

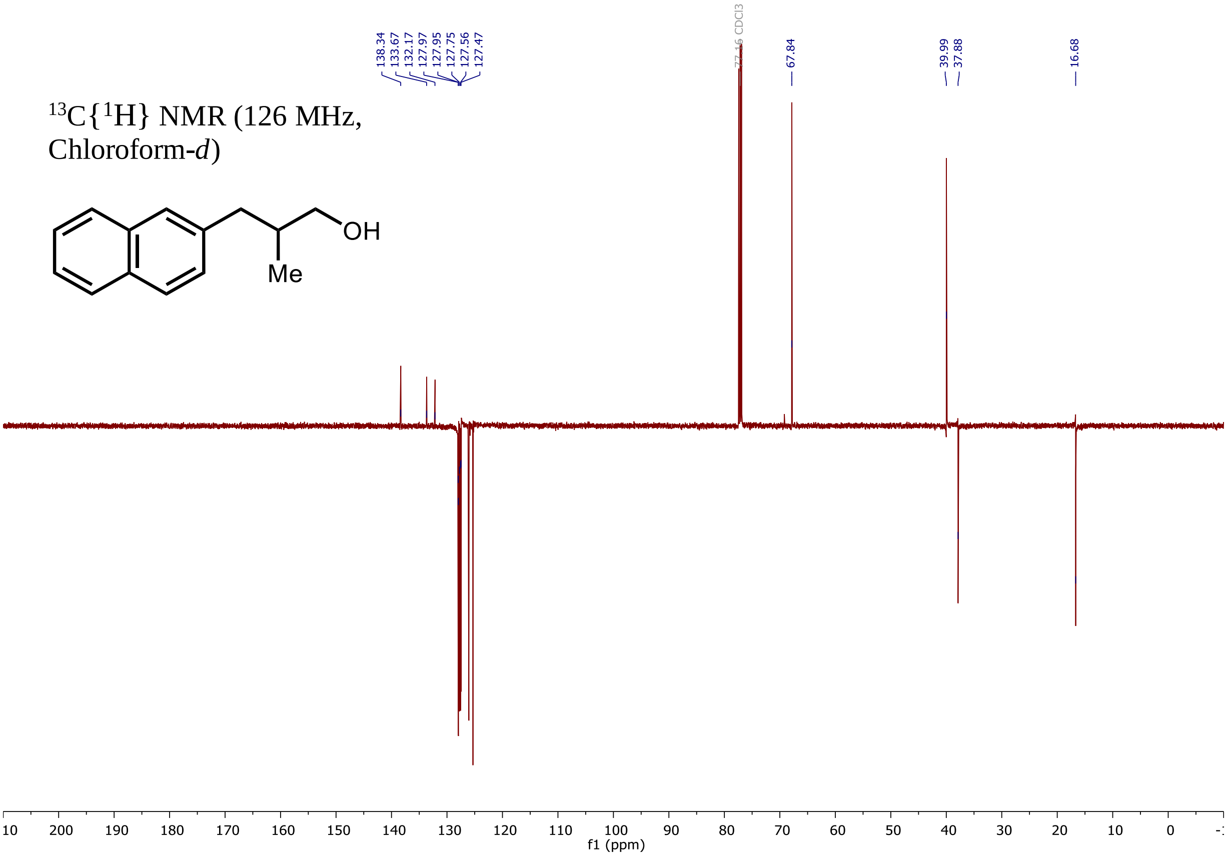
138.34 133.67 132.17 127.97 127.95 127.75 127.56 127.47
77.16 CDCl3
67.84
39.99 37.88
16.68
¹³C{¹H} NMR (126 MHz, Chloroform-d)
OH
Me
f1 (ppm)

2-methyl-3-(1-methyl-1H-indol-3-yl)propan-1-ol (21)

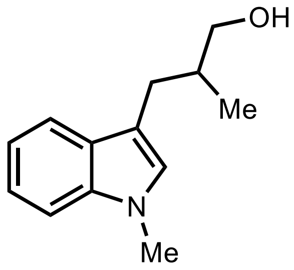

The title compound was prepared according to general procedure 7 using (*E*)-2-methyl-3-(1-methyl-1H-indol-3-yl)prop-2-en-1-ol (201 mg, 1 mmol) for 48 h and purified by flash silica chromatography (15-45% EtOAc in petroleum ether, 35 × 160 mm silica) to give the title compound as a yellow oil (125 mg, 61% isolated yield, 82% NMR yield); R_f = 0.59 (50% EtOAc in petroleum ether); v_{max} / cm^{-1} (film) 3348, 2953, 2910, 1472, 1423, 1375, 1325, 1250, 1157, 1130, 1030, 739, 420; ^{1}H NMR (500 MHz, Chloroform-*d*) δ 7.62 – 7.56 (m, 1H), 7.27 (dq, *J* = 8.2, 1.1 Hz, 1H), 7.20 (dddd, *J* = 9.1, 7.0, 2.1, 1.3 Hz, 1H), 7.09 (dddd, *J* = 8.0, 6.9, 2.5, 1.0 Hz, 1H), 6.82 (d, *J* = 1.0 Hz, 1H), 3.70 (s, 3H), 3.55 (ddd, *J* = 10.6, 5.9, 1.1 Hz, 1H), 3.47 (ddd, *J* = 10.6, 6.1, 1.1 Hz, 1H), 2.83 (ddt, *J* = 14.4, 6.5, 1.3 Hz, 1H), 2.59 (ddt, *J* = 14.4, 7.5, 0.9 Hz, 1H), 2.07 – 1.99 (m, 1H), 1.64 (s, 1H), 0.96 (dd, *J* = 6.7, 1.6 Hz, 3H); ^{13}C{^{1}H} NMR (126 MHz, Chloroform-*d*) δ 137.1, 128.3, 127.1, 121.5, 119.2, 118.7, 113.2, 109.2, 68.1, 37.0, 32.6, 28.9, 17.1; HRMS (EI-quadrupole) $(M)^+$ Calcd for $C_{13}H_{17}ON$ 203.1305; Found 203.1302.

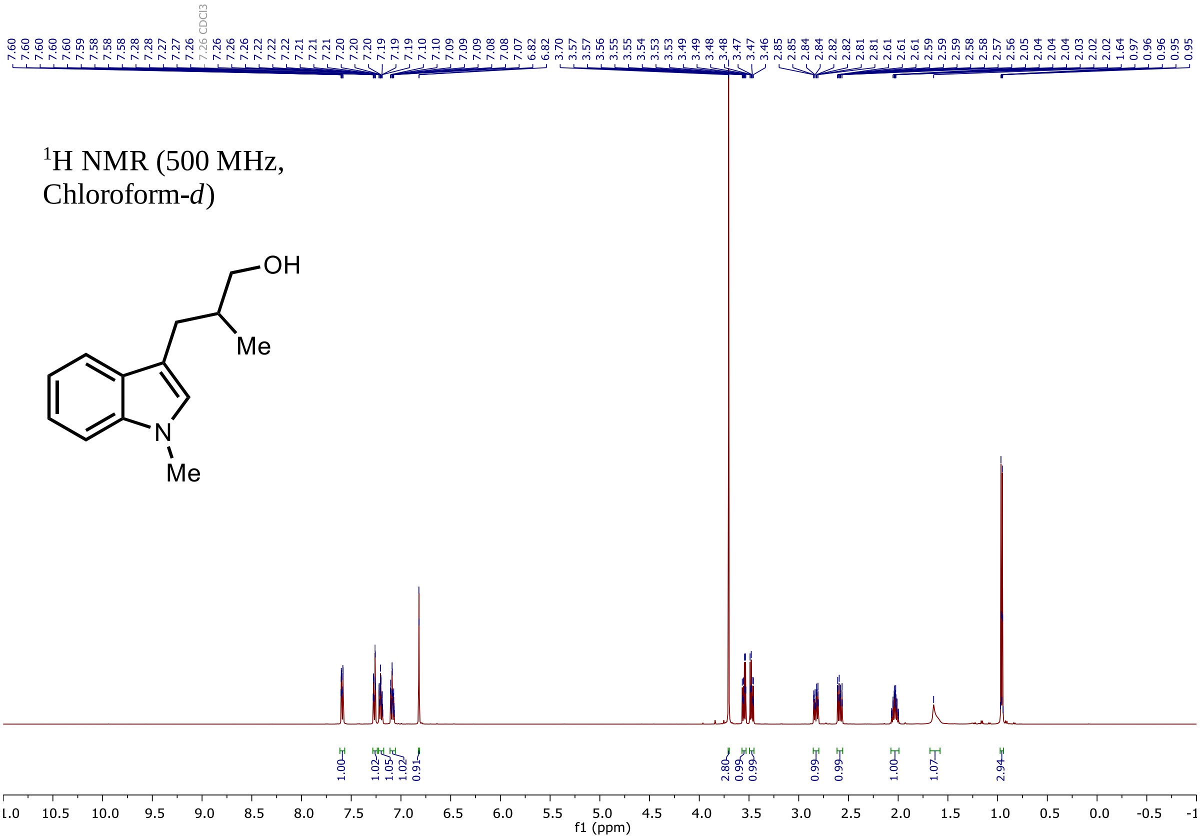

1H NMR (500 MHz, Chloroform-d)
OH
Me
N
Me
f1 (ppm)

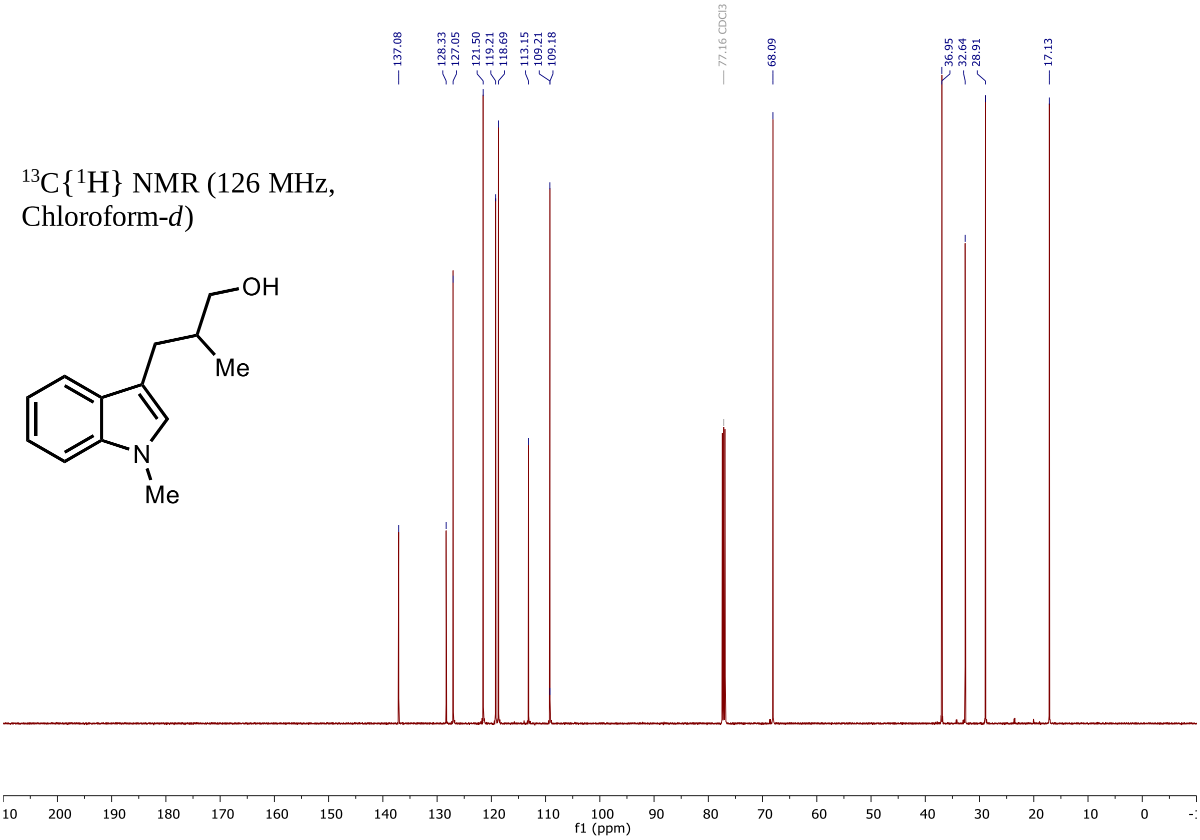

13C{1H} NMR (126 MHz, Chloroform-d)
137.08
128.33
127.05
121.50
119.21
118.69
113.15
109.21
109.18
77.16 CDCl3
68.09
36.95
32.64
28.91
17.13
OH
Me
N
Me
f1 (ppm)

3-(furan-2-yl)-2-methylpropan-1-ol (22)

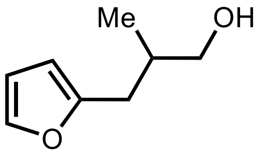

The title compound was prepared according to general procedure 7 using (*E*)-3-(furan-2-yl)-2-methylprop-2-en-1-ol (138 mg, 1 mmol) for 24 h and purified by flash silica chromatography (10–20% EtOAc in petroleum ether, 35 × 160 mm silica) to give the title compound as an orange oil (78 mg, 56% isolated yield, 85% NMR yield); R_f = 0.6 (20% EtOAc in petroleum ether); ν_{max} / cm^{-1} (film) 3397, 2980, 2970, 2887, 1379, 1252, 1148, 1080, 1007, 957, 800, 727; ^1H NMR (500 MHz, Chloroform-*d*) δ 7.31 (dd, *J* = 1.9, 0.9 Hz, 1H), 6.28 (ddt, *J* = 3.1, 1.8, 0.4 Hz, 1H), 6.02 (dq, *J* = 3.1, 0.8 Hz, 1H), 3.55 – 3.45 (m, 2H), 2.73 (ddt, *J* = 14.9, 6.3, 0.5 Hz, 1H), 2.54 (ddt, *J* = 15.0, 7.3, 0.5 Hz, 1H), 2.08 – 1.97 (m, 1H), 1.52 (bs, 1H), 0.95 (d, *J* = 6.8 Hz, 3H); $^{13}C\{^1H\}$ NMR (126 MHz, Chloroform-*d*) δ 154.7, 141.2, 110.3, 106.3, 67.6, 35.6, 31.7, 16.7; HRMS (EI-quadrupole) $(M)^+$ Calcd for $C_8H_{12}O_2$ 140.0832; Found 140.0828.

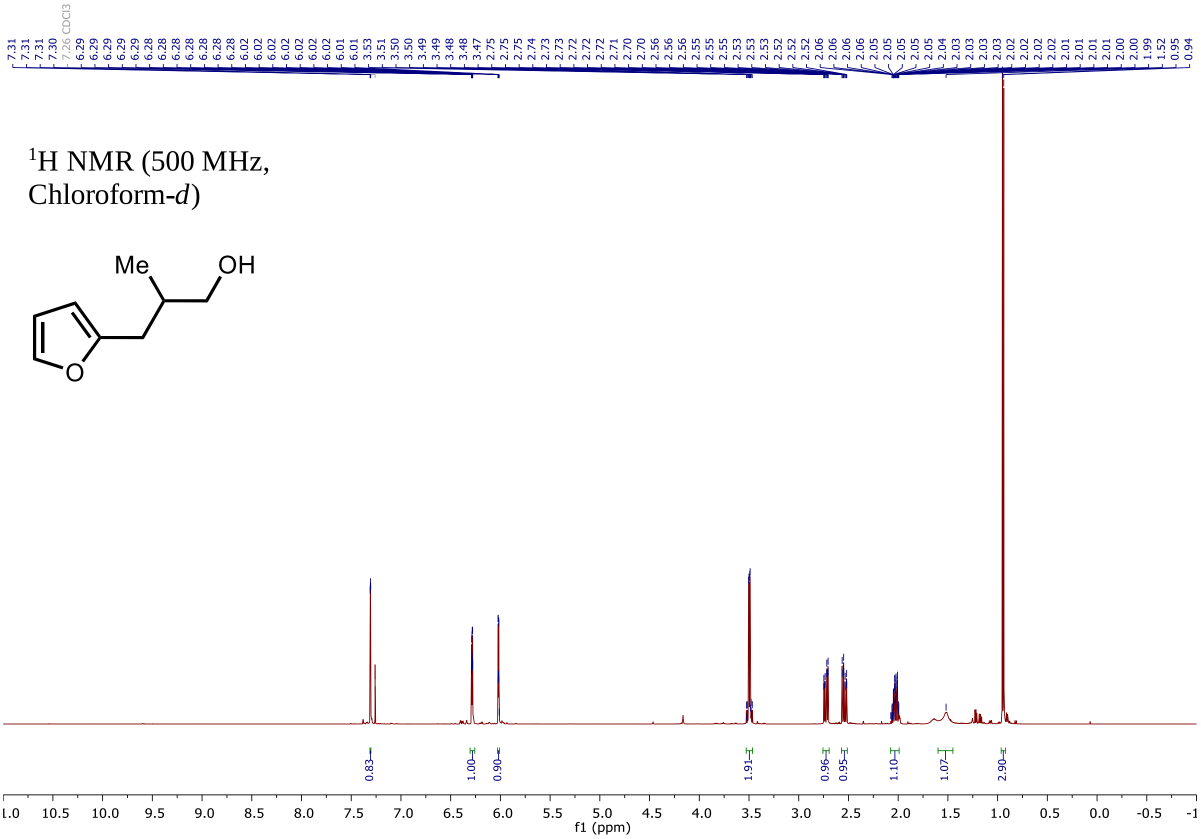

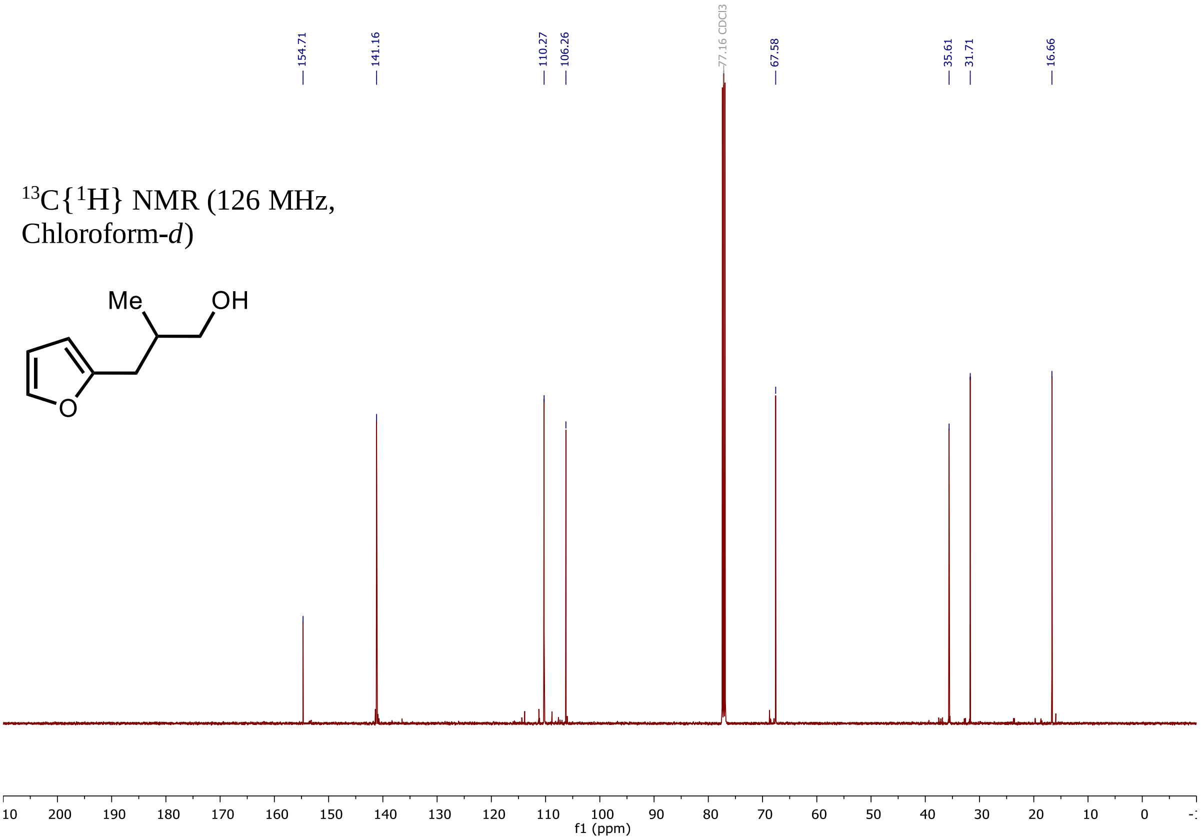

2-methyl-3-(thiophen-2-yl)propan-1-ol (23)

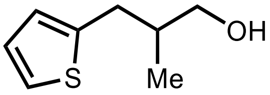

The title compound was prepared according to general procedure 7 using (*E*)-2-methyl-3-(thiophen-2-yl)prop-2-en-1-ol (154 mg, 1 mmol) for 24 h and purified by flash silica chromatography (10% EtOAc in petroleum ether, 35 × 160 mm silica) to give the title compound as a yellowish oil (111 mg, 61% isolated yield, 69% NMR yield); R_f = 0.22 (10% EtOAc in petroleum ether); ν_{max} / cm^{-1} (film) 3333, 2914, 2872, 1437, 1031, 849, 686; ^1H NMR (500 MHz, Chloroform-*d*) δ 7.13 (dd, *J* = 5.1, 1.2 Hz, 1H), 6.93 (dd, *J* = 5.1, 3.4 Hz, 1H), 6.80 (dd, *J* = 3.4, 0.9 Hz, 1H), 3.54 (qd, *J* = 10.6, 5.9 Hz, 2H), 2.97 (dd, *J* = 14.6, 6.2 Hz, 1H), 2.70 (dd, *J* = 14.6, 7.7 Hz, 1H), 2.11 – 1.80 (m, 1H), 1.44 (s, 1H), 0.97 (d, *J* = 6.8 Hz, 3H); $^{13}C\{^1H\}$ NMR (126 MHz, Chloroform-*d*) δ 143.3, 126.9, 125.4, 123.5, 67.4, 38.3, 33.6, 16.6; HRMS (EI-quadrupole) $(M)^+$ Calcd for $C_8H_{12}OS$ 156.0603; Found 156.0601.

^{1}H NMR (500 MHz, Chloroform-*d*)

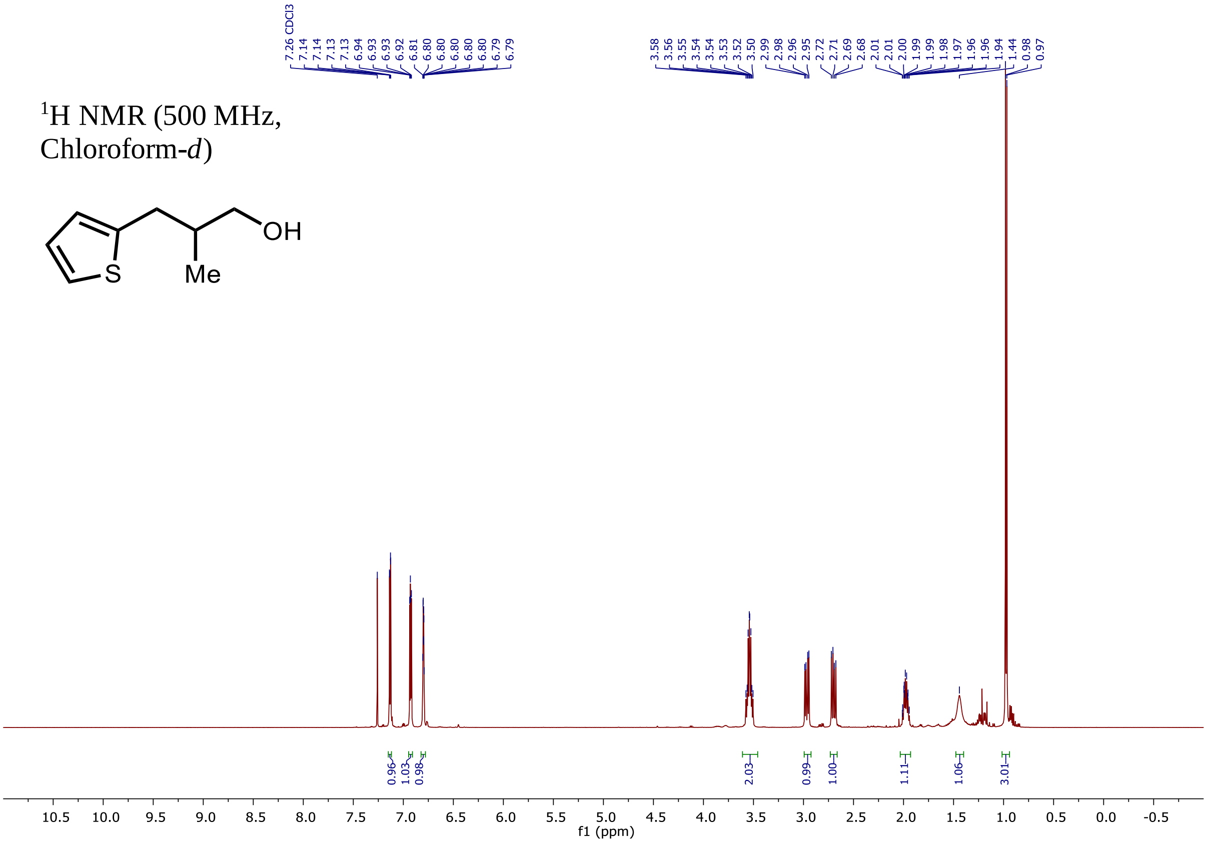

$^{13}C\{^{1}H\}$ NMR (126 MHz, Chloroform-*d*)

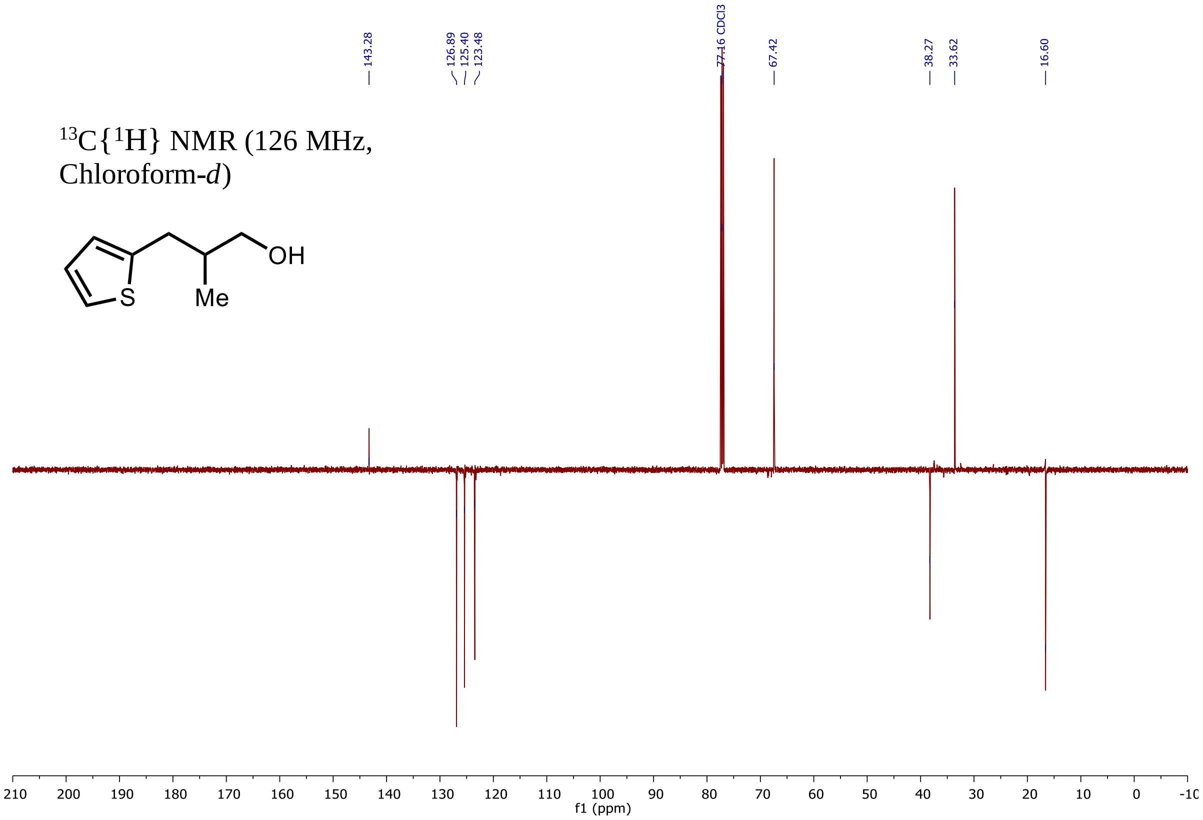

3-(furan-3-yl)-2-methylpropan-1-ol (24)

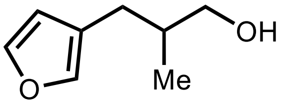

The title compound was prepared according to general procedure 7 using (*E*)-3-(furan-3-yl)-2-methylprop-2-en-1-ol (149 mg, 1 mmol) for 48 h and purified by flash silica chromatography (25% EtOAc in petroleum ether, 35 × 160 mm silica) to give the title compound as a pale yellow oil (103 mg, 74% isolated yield, 95% NMR yield); R_f = 0.63 (25% EtOAc in petroleum ether); ν_{max} / cm^{-1} (film) 3425, 2956, 2918, 2873, 1502, 1458, 1377, 1159, 1087, 1022, 871, 779, 727, 624, 599, 447, 432, 412, 401; ^{1}H NMR (500 MHz, Chloroform-*d*) δ 7.36 (qt, *J* = 1.1, 0.5 Hz, 1H), 7.23 (dp, *J* = 1.7, 0.9 Hz, 1H), 6.26 (ddd, *J* = 1.8, 0.9, 0.5 Hz, 1H), 3.50 (qdd, *J* = 10.6, 6.0, 1.6 Hz, 2H), 2.54 (dd, *J* = 14.3, 6.2 Hz, 1H), 2.28 (dd, *J* = 14.3, 8.2 Hz, 1H), 1.89 – 1.84 (m, 1H), 1.62 (bs, 1H), 0.92 (dd, *J* = 6.8, 1.1 Hz, 3H); ^{13}C{^{1}H} NMR (75 MHz, Chloroform-*d*) δ 142.9, 139.7, 123.1, 111.6, 67.7, 36.6, 28.5, 16.6; HRMS (CI-quadrupole) (M - H)$^+$ Calcd for $C_8H_{11}O_2$ 139.0754; Found 139.0754.

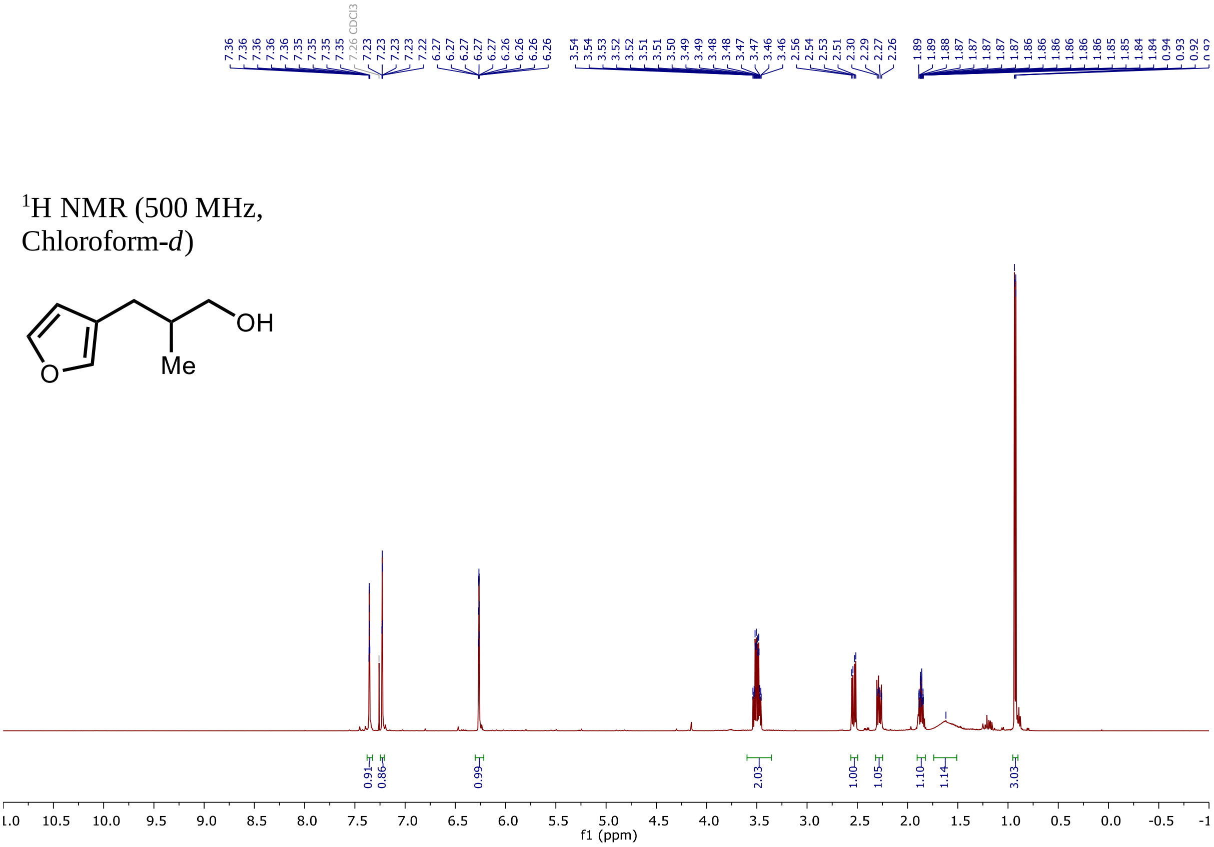

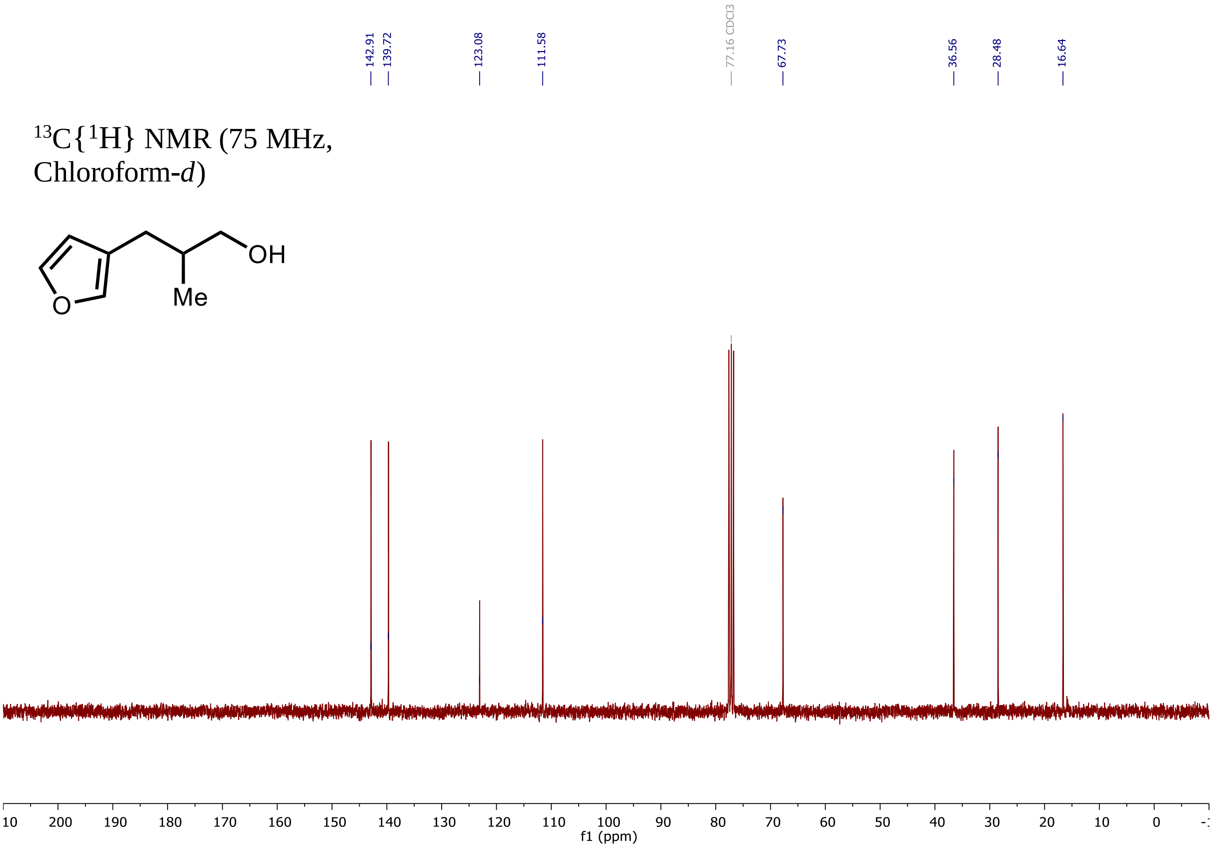

2-methyl-3-(thiophen-3-yl)propan-1-ol (25)

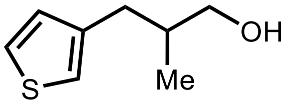

The title compound was prepared according to general procedure 7 using (*E*)-2-methyl-3-(thiophen-3-yl)prop-2-en-1-ol (182 mg, 1 mmol) for 24 h and purified by flash silica chromatography (10% EtOAc in petroleum ether, 35 × 160 mm silica) to give the title compound as a colourless oil (120 mg, 76% isolated yield, 90% NMR yield); R_f = 0.18 (10% EtOAc in petroleum ether); ν_{max} / cm^{-1} (film) 3335, 2921, 2871, 1456, 1027, 772, 633; ^{1}H NMR (500 MHz, Chloroform-*d*) δ 7.27 – 7.24 (m, 1H), 6.96 – 6.92 (m, 2H), 3.53 (dd, *J* = 10.6, 6.0 Hz, 1H), 3.48 (dd, *J* = 10.6, 6.0 Hz, 1H), 2.76 (dd, *J* = 13.9, 6.5 Hz, 1H), 2.50 (dd, *J* = 14.1, 7.8 Hz, 1H), 2.01 – 1.90 (m, 1H), 1.40 (s, 1H), 0.93 (d, *J* = 6.8 Hz, 3H); ^{13}C{^{1}H} NMR (126 MHz, Chloroform-*d*) δ 141.0, 128.8, 125.4, 121.2, 67.8, 37.2, 34.1, 16.7; HRMS (EI-quadrupole) $(M)^+$ Calcd for $C_8H_{12}OS$ 156.0603; Found 156.0602.

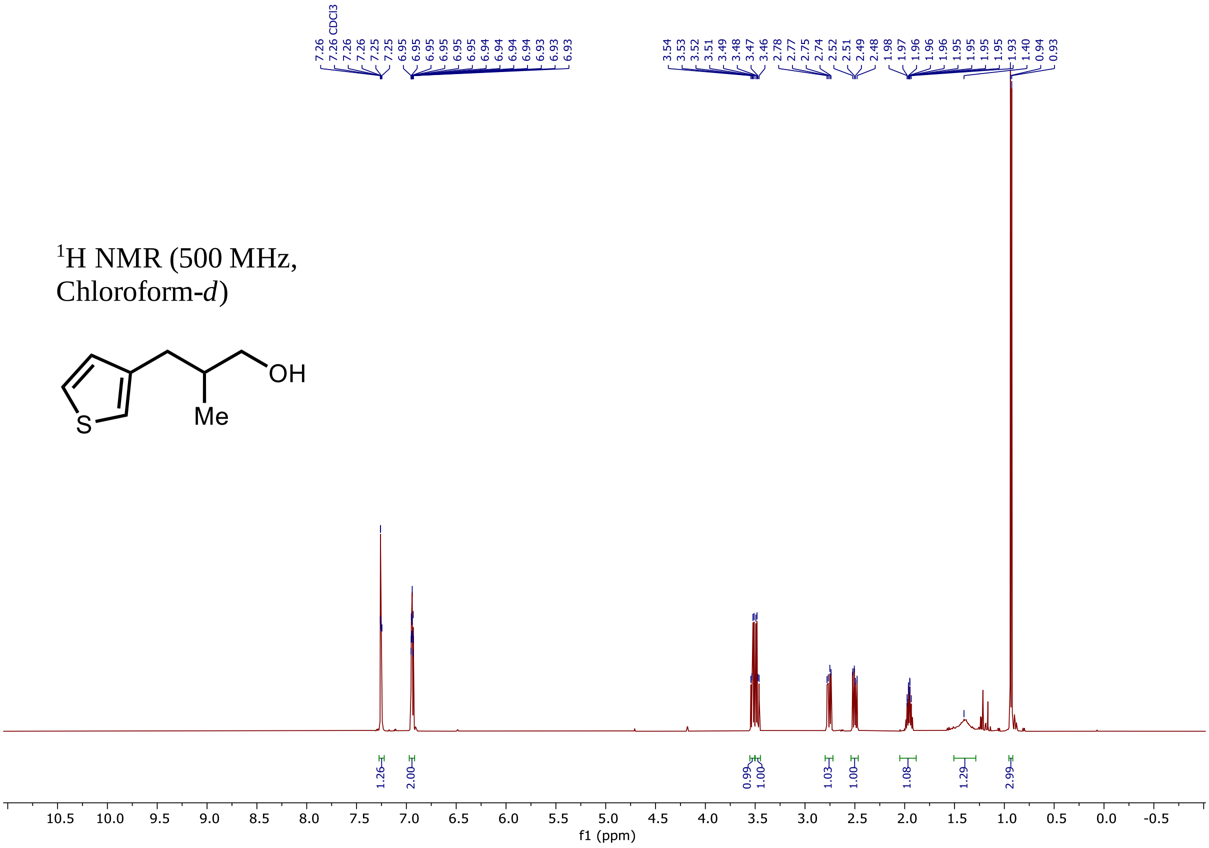

^{1}H NMR (500 MHz, Chloroform-*d*)
OH
Me
S
7.26 CDCl3
1.26
2.00
0.99
1.00
1.03
1.00
1.08
1.29
2.99
f1 (ppm)

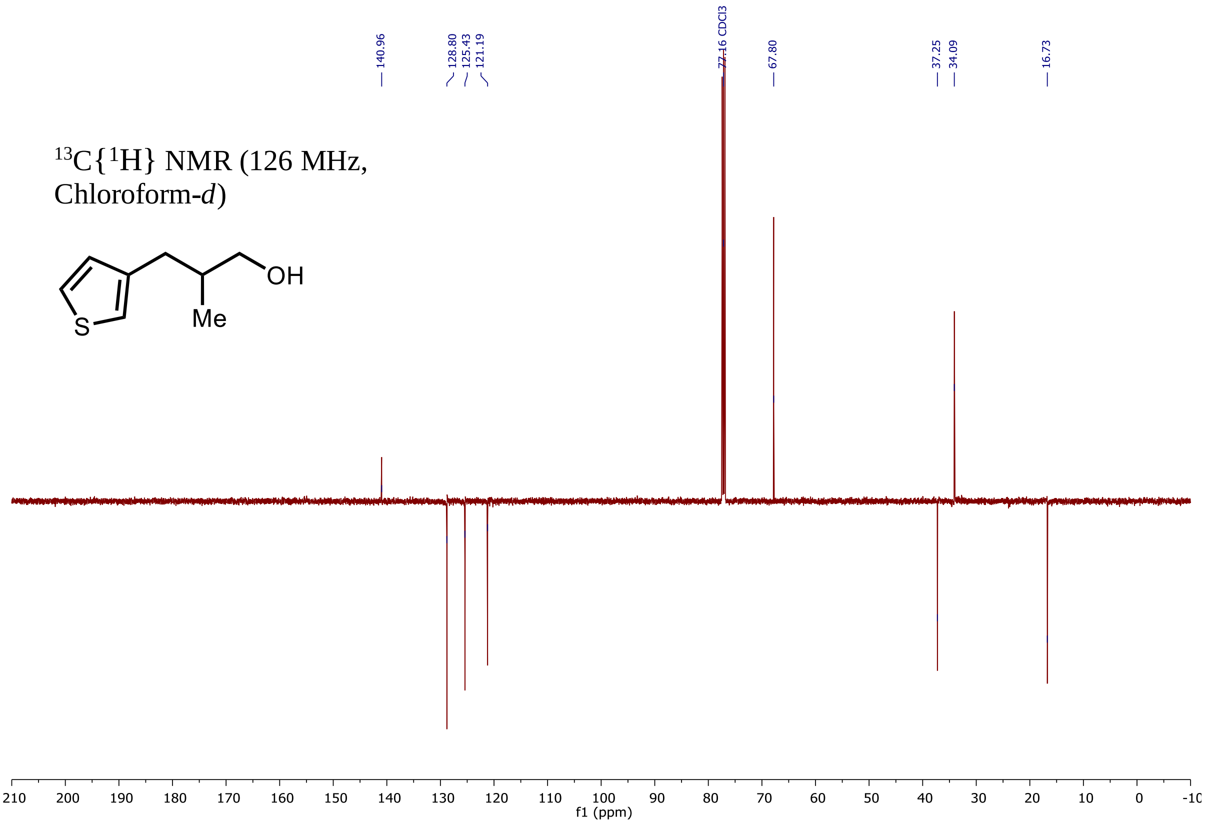

^{13}C{^{1}H} NMR (126 MHz, Chloroform-*d*)
OH
Me
S
140.96
128.80
125.43
121.19
77.16 CDCl3
67.80
37.25
34.09
16.73
f1 (ppm)

2-methyl-3-(pyridin-3-yl)propan-1-ol (26)

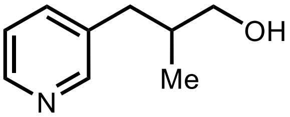

The title compound was prepared according to general procedure 7 using (*E*)-2-methyl-3-(pyridin-3-yl)prop-2-en-1-ol (149 mg, 1 mmol) for 48 h and purified by flash silica chromatography (50-80% EtOAc in petroleum ether, 35 × 160 mm silica) to give the title compound as a pale yellow oil (124 mg, 82% isolated yield, 85% NMR yield); R_f = 0.25 (80% EtOAc in petroleum ether); ν_{max} / cm^{-1} (film) 3304, 2956, 2916, 2872, 1577, 1479, 1423, 1377, 1109, 1029, 987, 823, 781, 713, 640, 437, 412; ^{1}H NMR (300 MHz, Chloroform-*d*) δ 8.44 (s, 2H), 7.52 (dt, *J* = 7.9, 1.9 Hz, 1H), 7.23 (dd, *J* = 7.8, 4.7 Hz, 1H), 3.51 (d, *J* = 6.0 Hz, 2H), 2.82 (dd, *J* = 13.6, 6.0 Hz, 1H), 2.42 (dd, *J* = 13.6, 8.3 Hz, 1H), 2.22 (bs, 1H), 1.96 – 1.88 (m, 1H), 0.91 (d, *J* = 6.8 Hz, 3H); ^{13}C{^{1}H} NMR (75 MHz, Chloroform-*d*) δ 150.3, 147.2, 136.9, 136.1, 123.4, 67.0, 37.5, 36.5, 16.2; HRMS (CI-quadrupole) (M + H)$^+$ Calcd for $C_9H_{14}NO$ 152.1070; Found 152.1070.

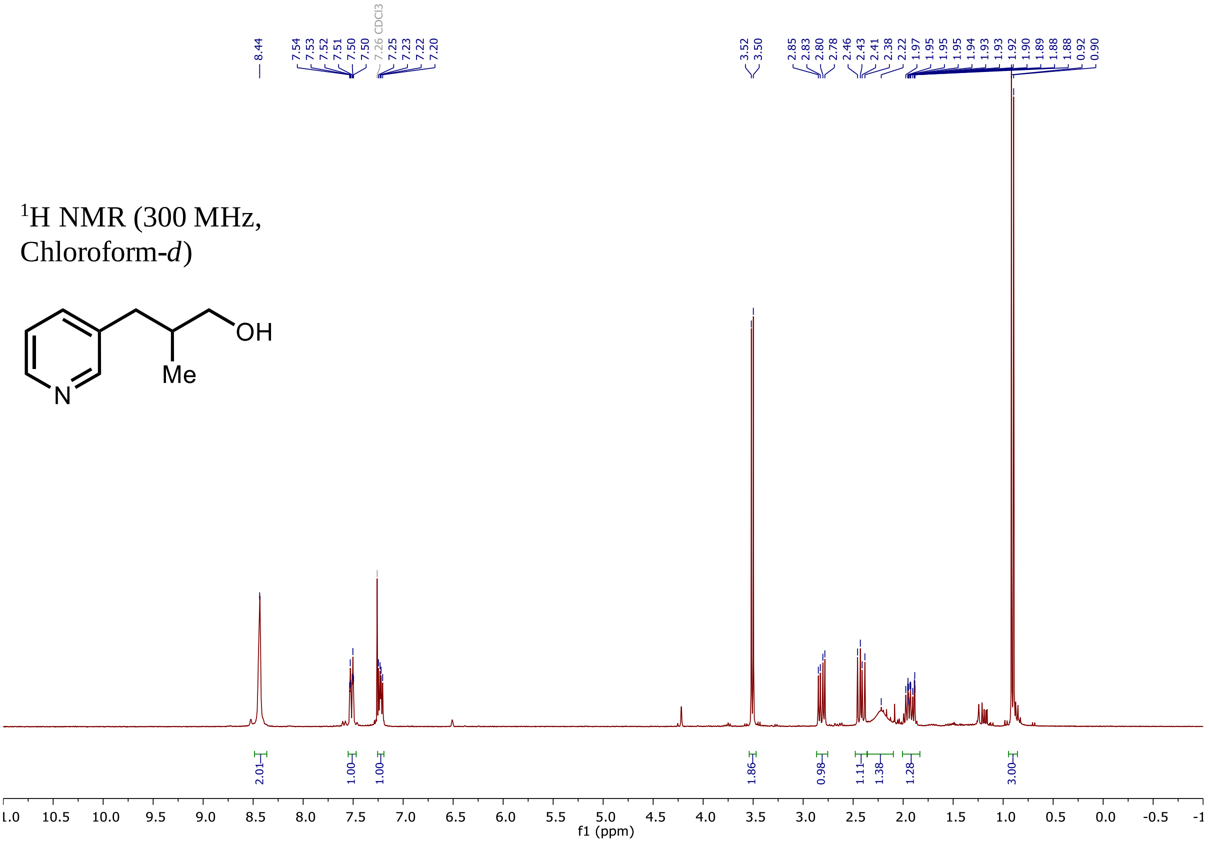

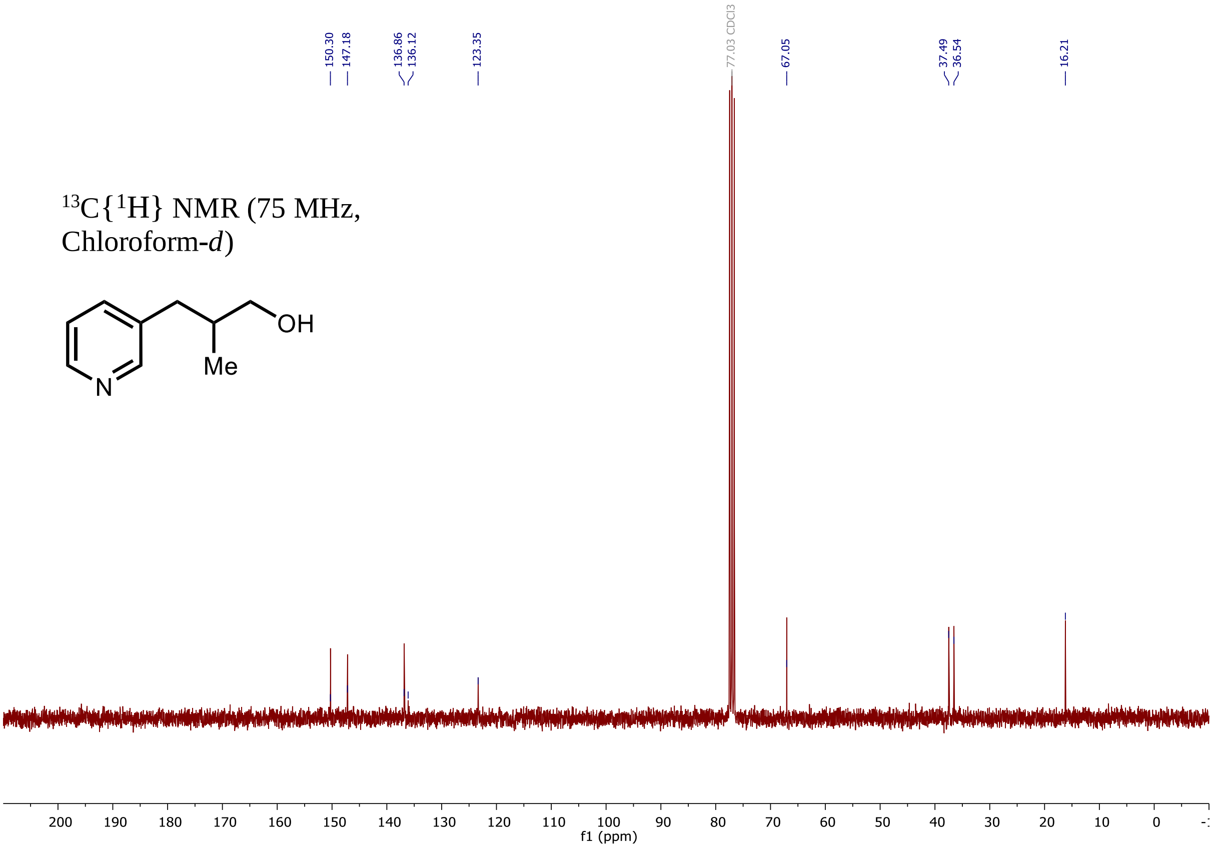

2-methyl-3-(pyridin-4-yl)propan-1-ol (27)

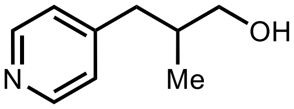

The title compound was prepared according to general procedure 7 using (*E*)-2-methyl-3-(pyridin-4-yl)prop-2-en-1-ol (149 mg, 1 mmol) for 48 h and purified by flash silica chromatography (100 % EtOAc, 35 × 160 mm silica) to give the title compound as a yellow oil (92 mg, 61% isolated yield, 85% NMR yield); v_{max} / cm^{-1} (film) 3360, 2970, 1923, 1607, 1418, 1034, 1003, 949; R_f = 0.17 (100% EtOAc); ^{1}H NMR (500 MHz, Chloroform-*d*) δ 8.51 – 8.46 (m, 2H), 7.14 – 7.08 (m, 2H), 3.51 (dd, *J* = 5.9, 0.8 Hz, 2H), 2.82 (dd, *J* = 13.4, 6.0 Hz, 1H), 2.40 (dd, *J* = 13.4, 8.4 Hz, 1H), 2.03 – 1.82 (m, 2H), 0.91 (d, *J* = 6.8 Hz, 3H); $^{13}C\{^{1}H\}$ NMR (126 MHz, Chloroform-*d*) δ 150.1, 149.7, 124.8, 67.3, 39.1, 37.2, 16.4; HRMS (EI-quadrupole) $(M)^+$ Calcd for $C_9H_{13}ON$ 151.0992; Found 151.0988.

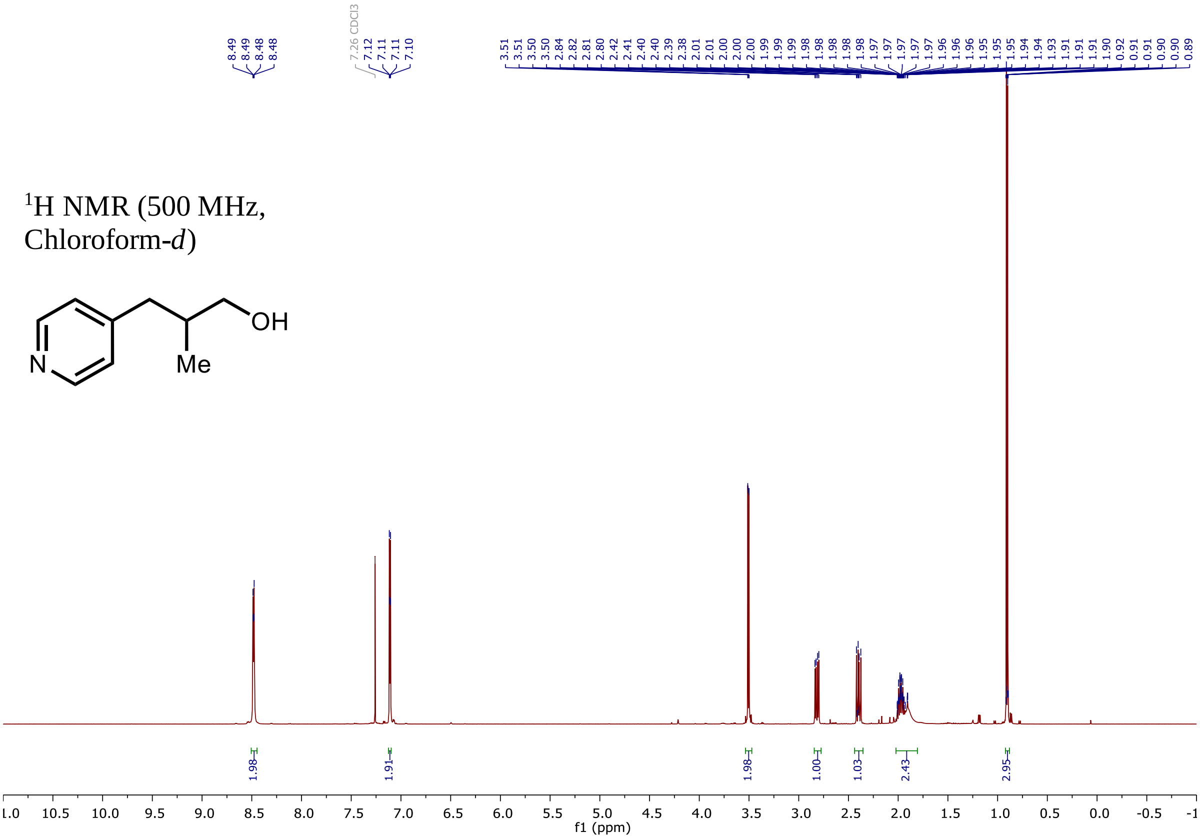

^{1}H NMR (500 MHz, Chloroform-*d*)
OH
N
Me
7.26 CDCl3
f1 (ppm)

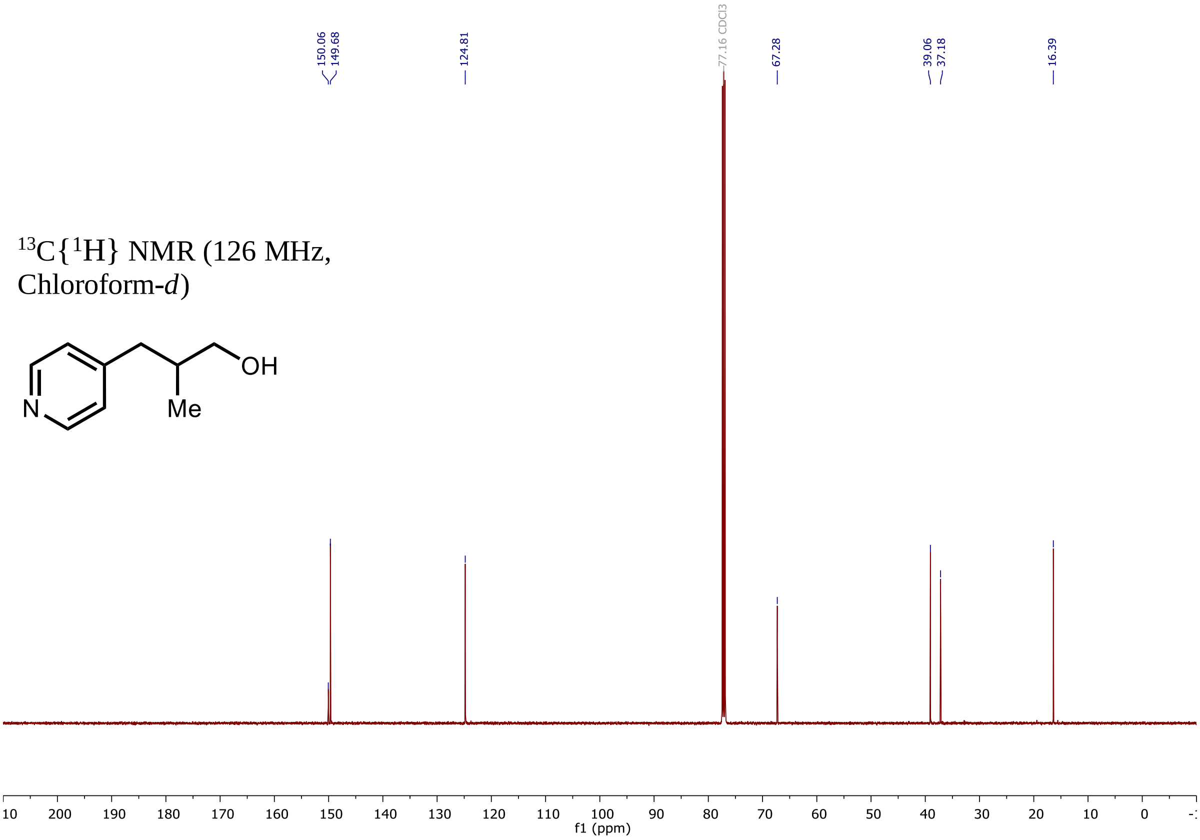

^{13}C{^{1}H} NMR (126 MHz, Chloroform-*d*)
OH
N
Me
150.06
149.68
124.81
77.16 CDCl3
67.28
39.06
37.18
16.39
f1 (ppm)

2-methyl-4-phenylbutan-1-ol (28)

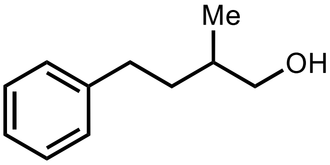

The title compound was prepared according to general procedure 7 using (*E*)-2-methyl-4-phenylbut-2-en-1-ol (162 mg, 1 mmol) for 48 h and purified by flash silica chromatography (15-20% EtOAc in petroleum ether, 35 × 160 mm silica) to give the title compound as a yellow oil (96 mg, 58% isolated yield, 88% NMR yield); R_f = 0.63 (20% EtOAc in petroleum ether); ν_{max} / cm^{-1} (film) 3337, 2955, 2922, 2872, 1495, 1454, 1032, 744, 698, 405; ^{1}H NMR (500 MHz, Chloroform-*d*) δ 7.32 – 7.24 (m, 2H), 7.22 – 7.14 (m, 2H), 3.54 (dt, *J* = 11.0, 5.6 Hz, 1H), 3.48 (dt, *J* = 10.5, 5.9 Hz, 1H), 2.71 (ddd, *J* = 13.7, 10.2, 5.6 Hz, 1H), 2.60 (ddd, *J* = 13.7, 10.1, 6.3 Hz, 1H), 1.76 (dddd, *J* = 13.2, 10.2, 6.3, 5.2 Hz, 1H), 1.67 (dddd, *J* = 12.0, 8.3, 6.7, 5.5 Hz, 1H), 1.45 (dddd, *J* = 13.4, 10.1, 8.0, 5.6 Hz, 1H), 1.25 (d, *J* = 6.2 Hz, 1H), 0.99 (d, *J* = 6.7 Hz, 3H); $^{13}C\{^{1}H\}$ NMR (126 MHz, Chloroform-*d*) δ 142.7, 128.5, 125.9, 68.4, 35.5, 35.1, 33.4, 16.7; HRMS (CI-quadrupole) $(M)^+$ Calcd for $C_{11}H_{16}O$ 164.1196; Found 164.1197.

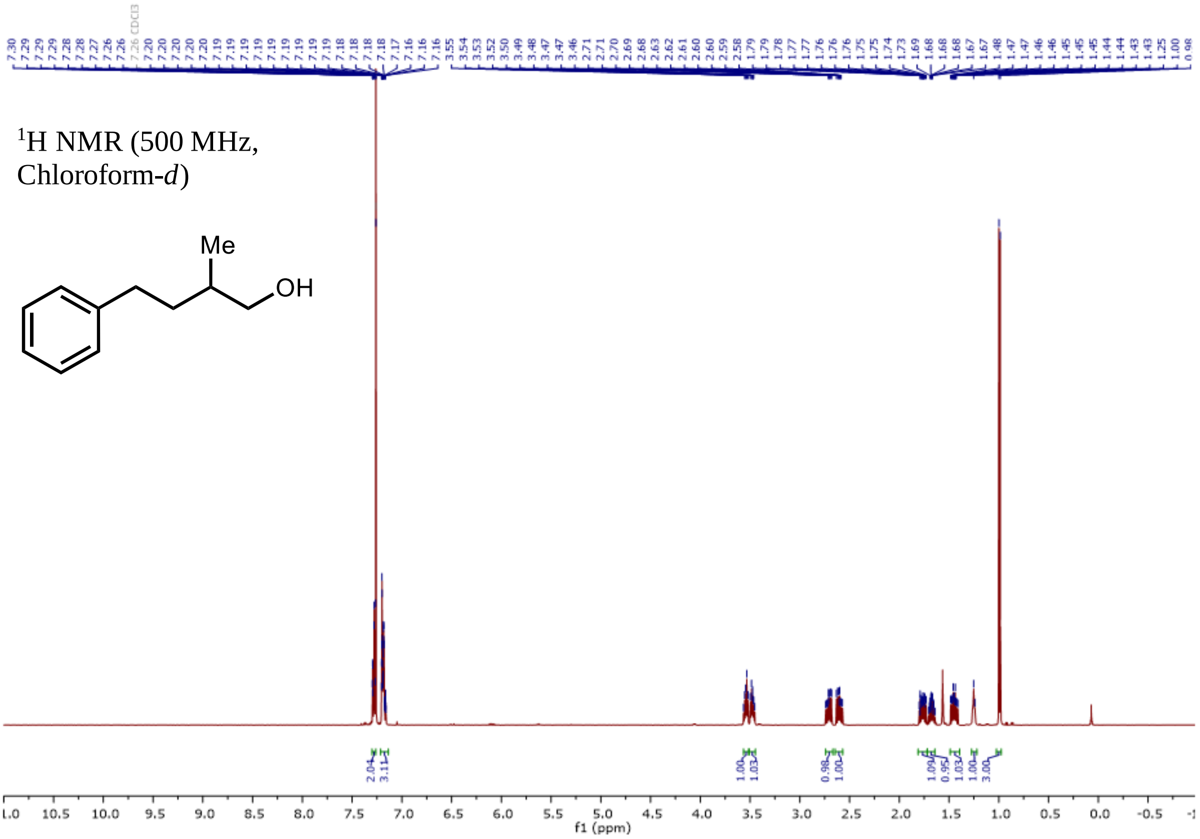

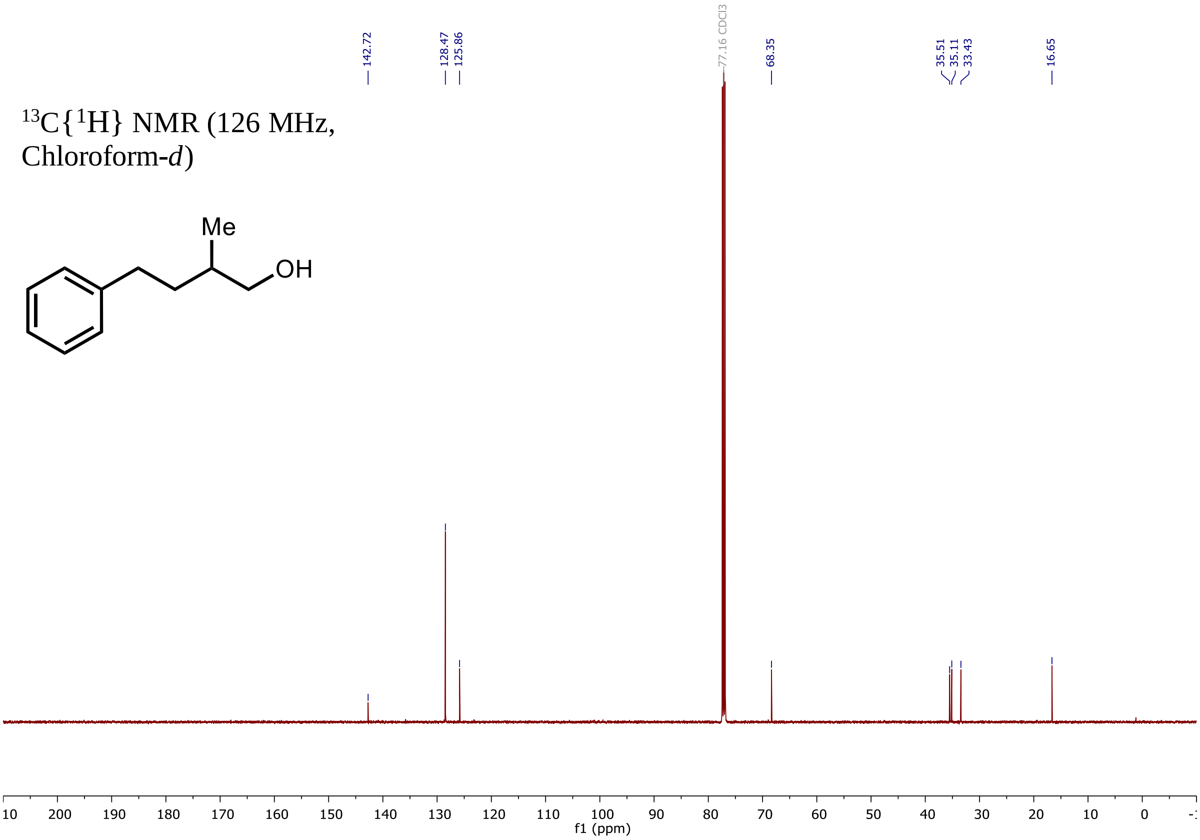

2-methyl-5-phenylpentan-1-ol (29)

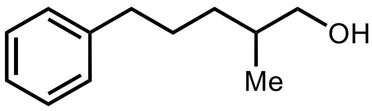

The title compound was prepared according to general procedure 7 using (*E*)-2-methyl-5-phenylpent-2-en-1-ol (176 mg, 1 mmol) for 24 h. The resulting crude was stirred with bromine water at 0 °C for 20 minutes and transferred to a separatory funnel and then aqueous phase was extracted with EtOAc (2 × 25 mL). Purification by flash silica chromatography (eluent = 20-40% EtOAc in petroleum ether) gave the title compound as a clear liquid (110 mg, 61% isolated yield, 89% NMR yield); R_f = 0.15 (20% EtOAc in petroleum ether); ν_{max} / cm^{-1} (film) 3342, 2927, 2858, 1494, 1452, 1099, 744, 696; ^{1}H NMR (400 MHz, Chloroform-*d*) δ 7.31 – 7.23 (m, 2H), 7.22 – 7.13 (m, 3H), 3.56 – 3.38 (m, 2H), 2.70 – 2.52 (m, 2H), 1.77 – 1.56 (m, 3H), 1.47 (ddt, *J* = 13.4, 10.8, 5.4 Hz, 1H), 1.29 – 1.10 (m, 2H), 0.92 (d, *J* = 6.7 Hz, 3H); ^{13}C{^{1}H} NMR (126 MHz, Chloroform-*d*) δ 142.8, 128.5, 128.4, 125.8, 68.4, 36.4, 35.8, 32.9, 29.1, 16.7; HRMS (EI-quadrupole) $(M)^+$ Calcd for $C_{12}H_{18}O$ 178.1352; Found 178.1347.

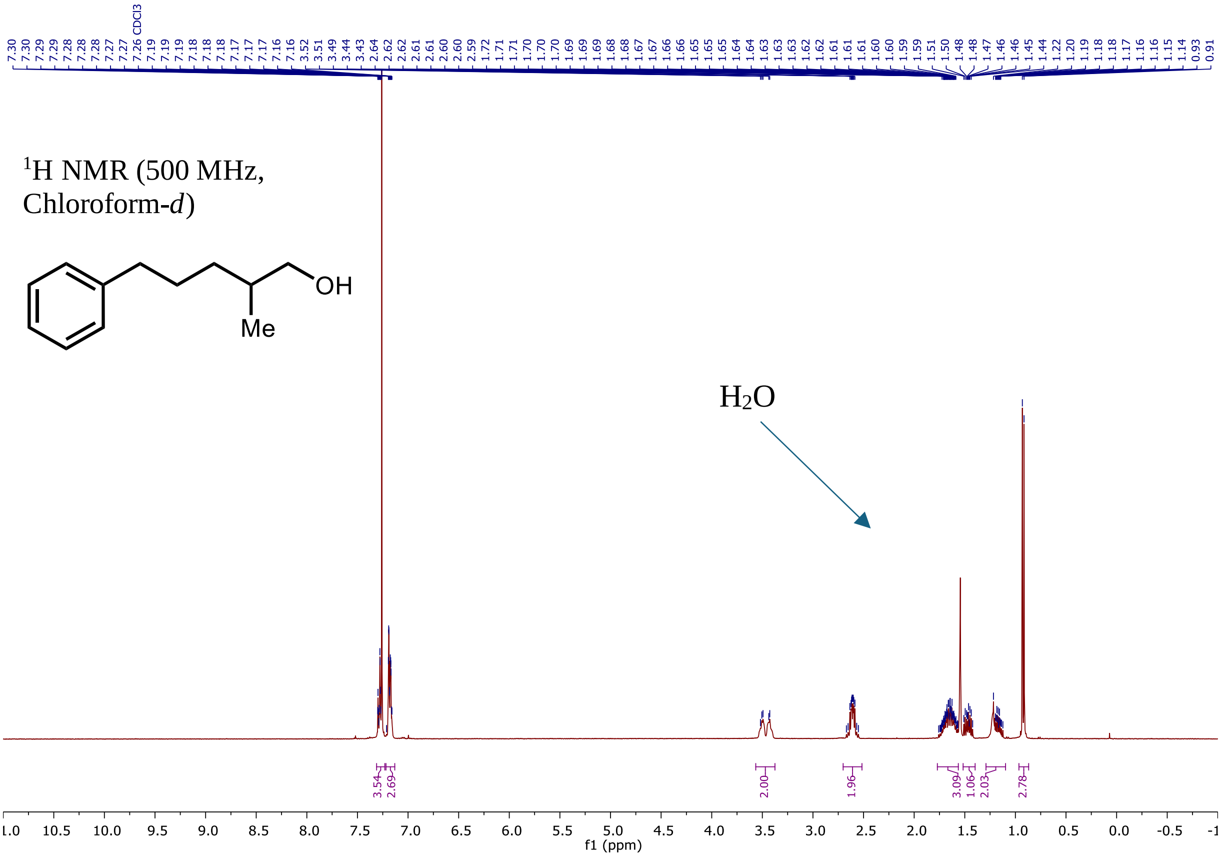

^{1}H NMR (500 MHz, Chloroform-d)
OH
Me
H2O
7.26 CDCl3
3.54
2.69
2.00
1.96
3.09
1.06
2.03
2.78
f1 (ppm)

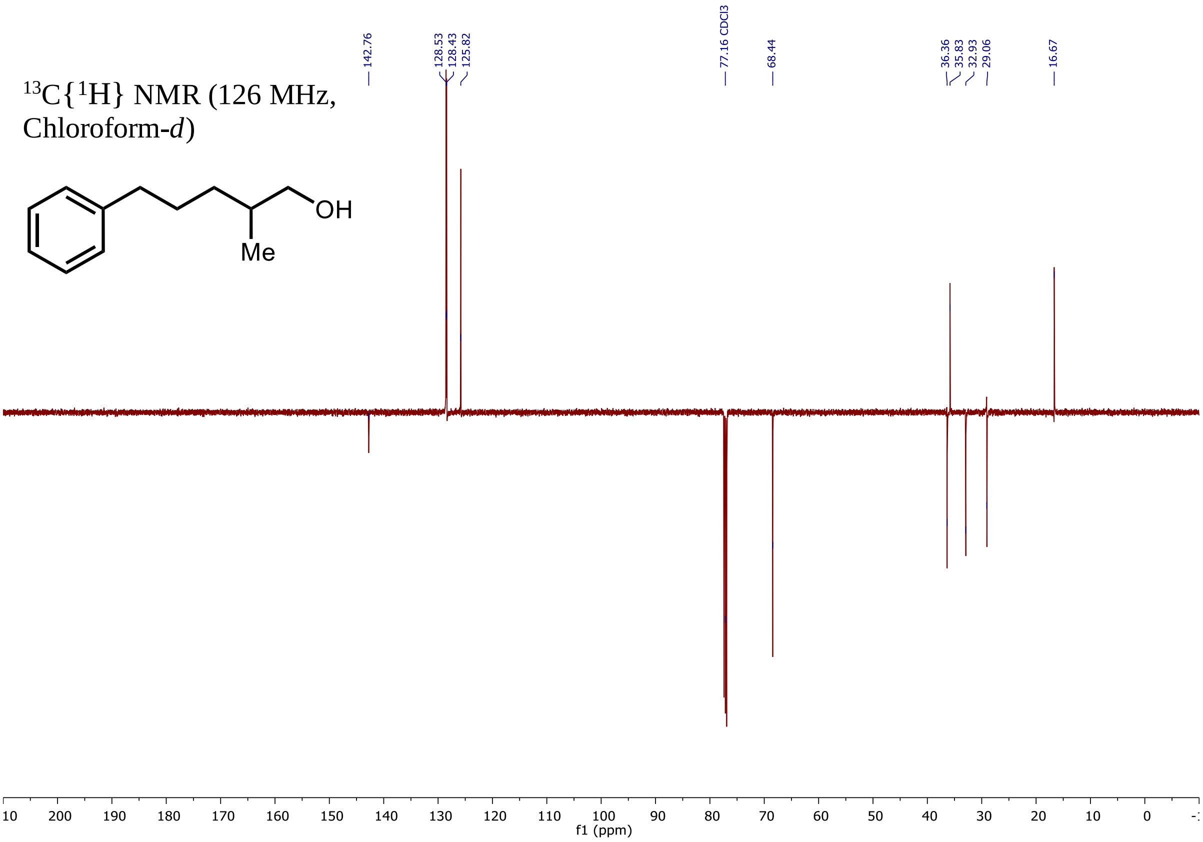

^{13}C{^{1}H} NMR (126 MHz, Chloroform-d)
OH
Me
142.76
128.53
128.43
125.82
77.16 CDCl3
68.44
36.36
35.83
32.93
29.06
16.67
f1 (ppm)

3-cyclohexyl-2-methylpropan-1-ol (30)

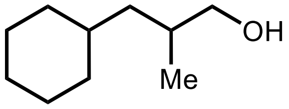

The title compound was prepared according to general procedure 7 using (*E*)-3-cyclohexyl-2-methylpropan-1-ol (156 mg, 1 mmol) for 24 h. The resulting crude was stirred with bromine at 0 °C for 20 minutes and transferred to a separatory funnel and then aqueous phase was extracted with EtOAc (2 × 25 mL). The purification by flash silica chromatography (5% EtOAc in petroleum ether, 35 × 160 mm silica) to give the title compound as a colourless oil (112 mg, 72% isolated yield, 82% NMR yield); R_f = 0.15 (10% EtOAc in petroleum ether); ν_{max} / cm^{-1} (film) 3319, 2918, 2848, 1448, 1031; ^1H NMR (500 MHz, Chloroform-*d*) δ 3.42 (dd, *J* = 10.5, 5.5 Hz, 1H), 3.31 (dd, *J* = 10.4, 6.7 Hz, 1H), 1.70 – 1.55 (m, 6H), 1.44 (s, 1H), 1.25 (ddt, *J* = 11.2, 5.5, 2.8 Hz, 1H), 1.20 – 1.03 (m, 4H), 0.91 (ddd, *J* = 13.4, 8.7, 5.7 Hz, 1H), 0.83 (d, *J* = 6.7 Hz, 3H), 0.82 – 0.70 (m, 2H); $^{13}C\{^1H\}$ NMR (126 MHz, Chloroform-*d*) δ 68.9, 41.2, 34.9, 34.4, 33.2, 32.7, 26.8, 26.6, 26.5, 17.0; HRMS (EI-quadrupole) (M - H_2O)$^+$ Calcd for $C_{10}H_{18}$ 138.1403; Found 138.1401.

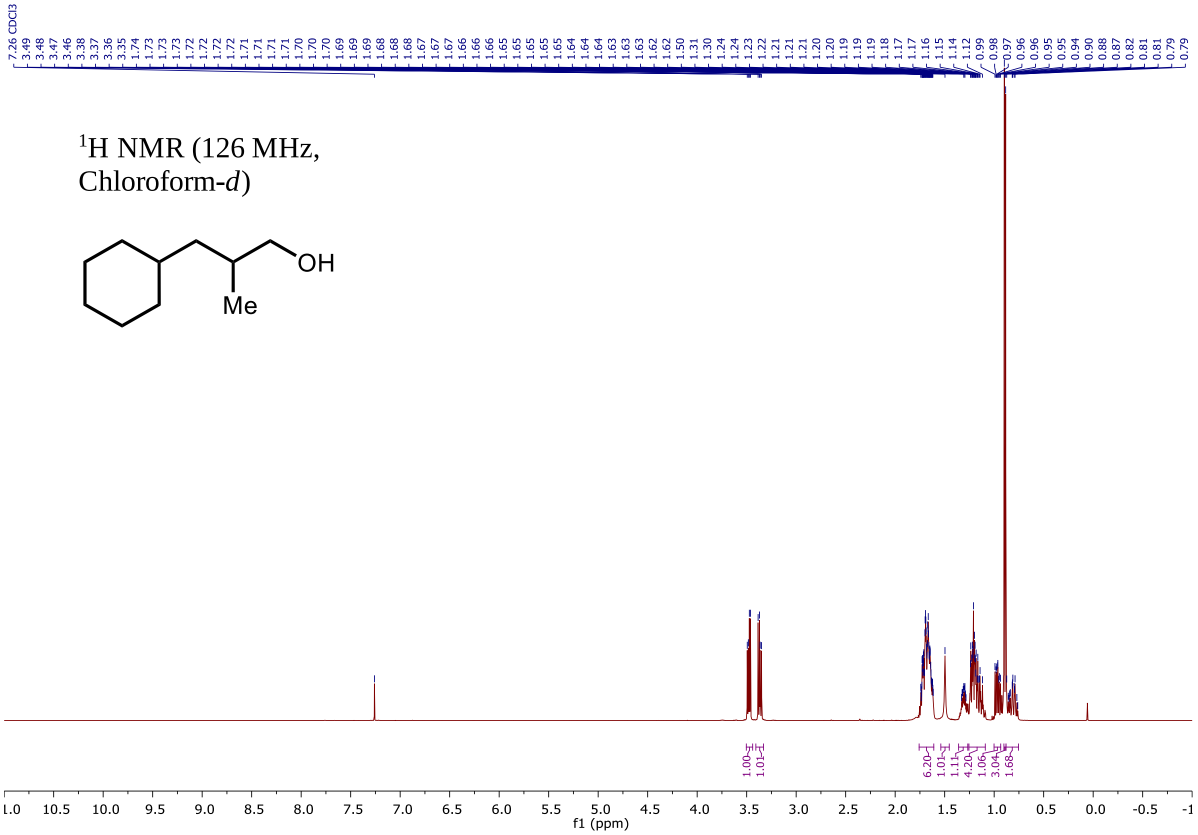

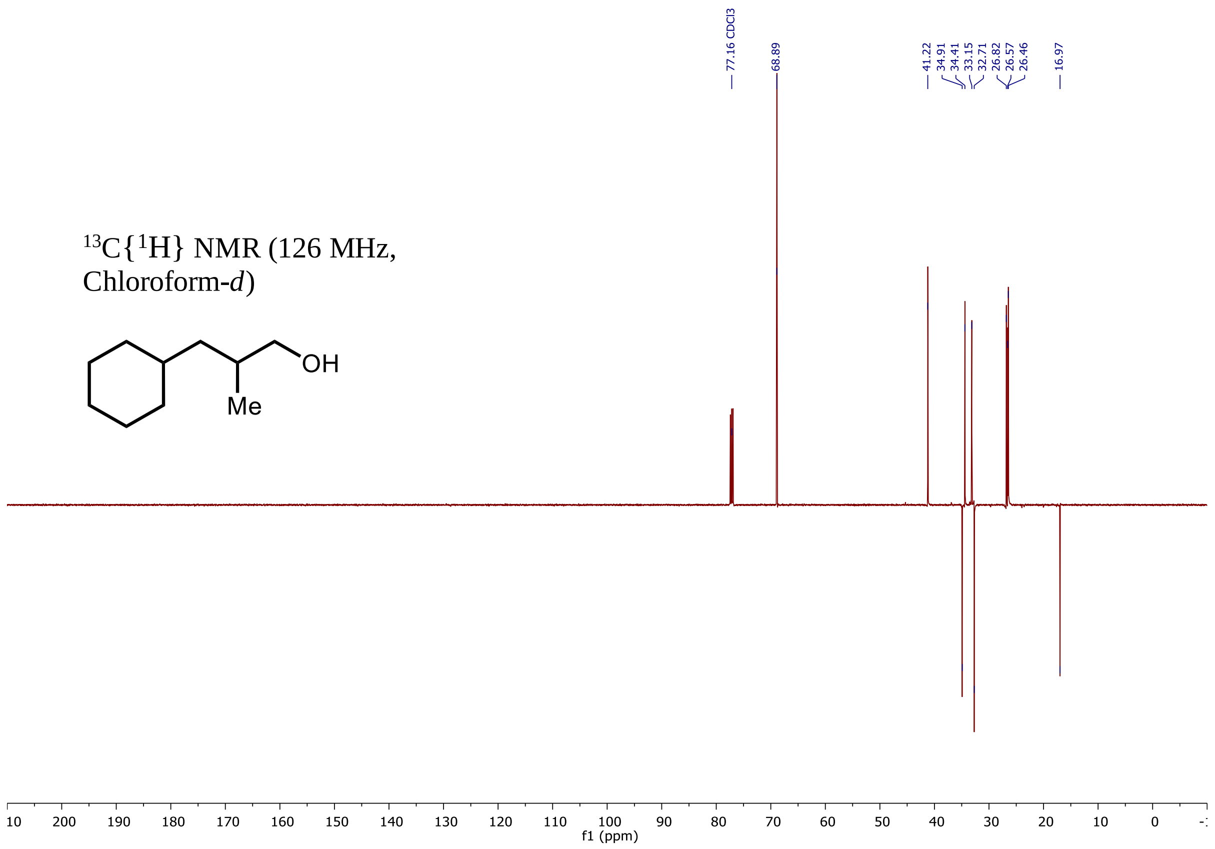

3-phenylpropan-1-ol (31)

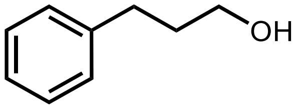

The title compound was prepared according to general procedure 7 using (*E*)-3-phenylprop-2-en-1-ol (134 mg, 1 mmol) for 24 h and purified by flash silica chromatography (10-20% EtOAc in petroleum ether, 35 × 160 mm silica) to give the title compound as a colourless oil (66 mg, 49% isolated yield, 86% NMR yield); $R_f = 0.17$ (5% EtOAc in petroleum ether); ^{1}H NMR (500 MHz, Chloroform-*d*) δ 7.31 – 7.27 (m, 2H), 7.22 – 7.16 (m, 3H), 3.69 (t, *J* = 7.7 Hz, 1H), 2.72 (t, *J* = 8 Hz, 2H), 1.95 – 1.86 (m, 2H), 1.26 (s, 1H); ^{13}C{^{1}H} NMR (126 MHz, Chloroform-*d*) δ 141.9, 128.57, 128.55, 126.0, 62.5, 34.4, 32.2. Spectroscopic data in accordance with that stated in the literature.[20]

^{1}H NMR (500 MHz, Chloroform-*d*)

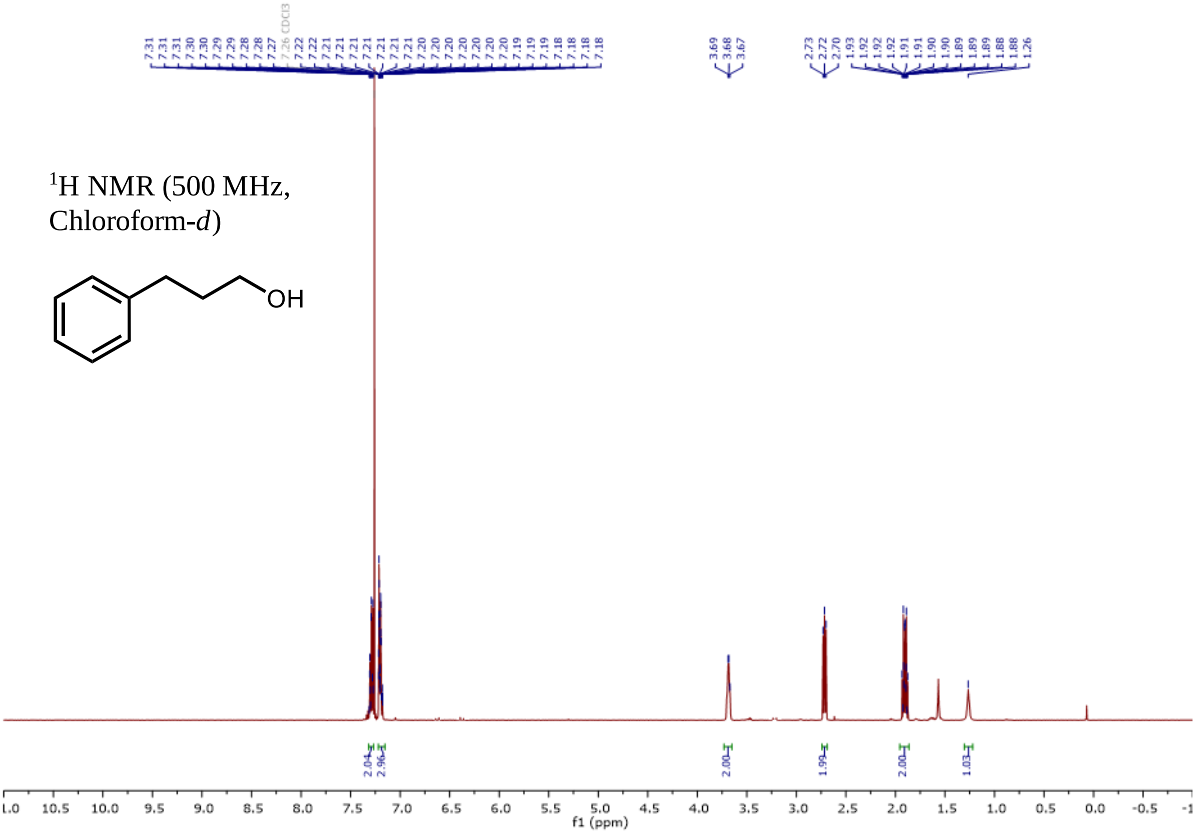

$^{13}C\{^{1}H\}$ NMR (126 MHz, Chloroform-*d*)

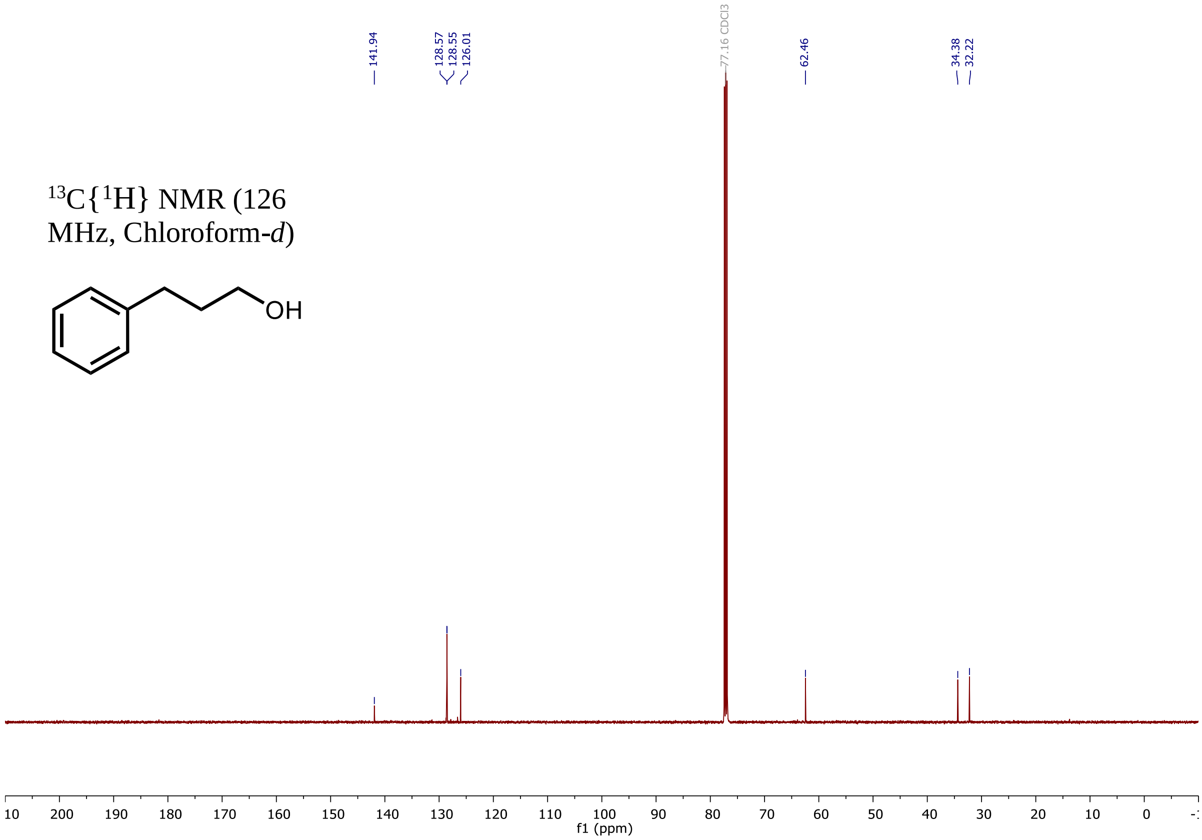

3-(4-fluorophenyl)propan-1-ol (32)

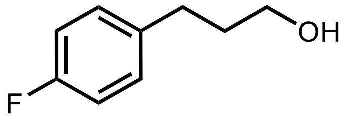

The title compound was prepared according to general procedure 7 using (*E*)-3-(4-fluorophenyl)prop-2-en-1-ol (152 mg, 1 mmol) for 24 h and purified by flash silica chromatography (10-20% EtOAc in petroleum ether, 35 × 160 mm silica) to give the title compound as a yellow oil (54 mg, 35% isolated yield, 66% NMR yield); R_f = 0.41 (30% EtOAc in petroleum ether); v_{max} / cm^{-1} (film) 3327, 2938, 2864, 1601, 1508, 1219, 1157, 1040, 820, 546, 501; ^{1}H NMR (500 MHz, Chloroform-*d*) δ 7.17 – 7.12 (m, 2H), 6.99 – 6.93 (m, 2H), 3.66 (t, *J* = 6.4 Hz, 2H), 2.68 (dd, *J* = 8.4, 7.1 Hz, 2H), 1.90 – 1.82 (m, 2H), 1.67 (bs, 1H); ^{13}C{^{1}H} NMR (126 MHz, Chloroform-*d*) δ 161.4 (d, J_{C-F} = 243.3 Hz), 137.5 (d, J_{C-F} = 3.1 Hz), 129.8 (d, J_{C-F} = 7.8 Hz), 115.2 (d, J_{C-F} = 21.1 Hz), 62.1, 34.4, 31.3; ^{19}F{^{1}H} NMR (471 MHz, Chloroform-*d*) δ -117.7; HRMS (EI-quadrupole) $(M)^+$ Calcd for $C_9H_{11}OF$ 154.0788; Found 154.0784.

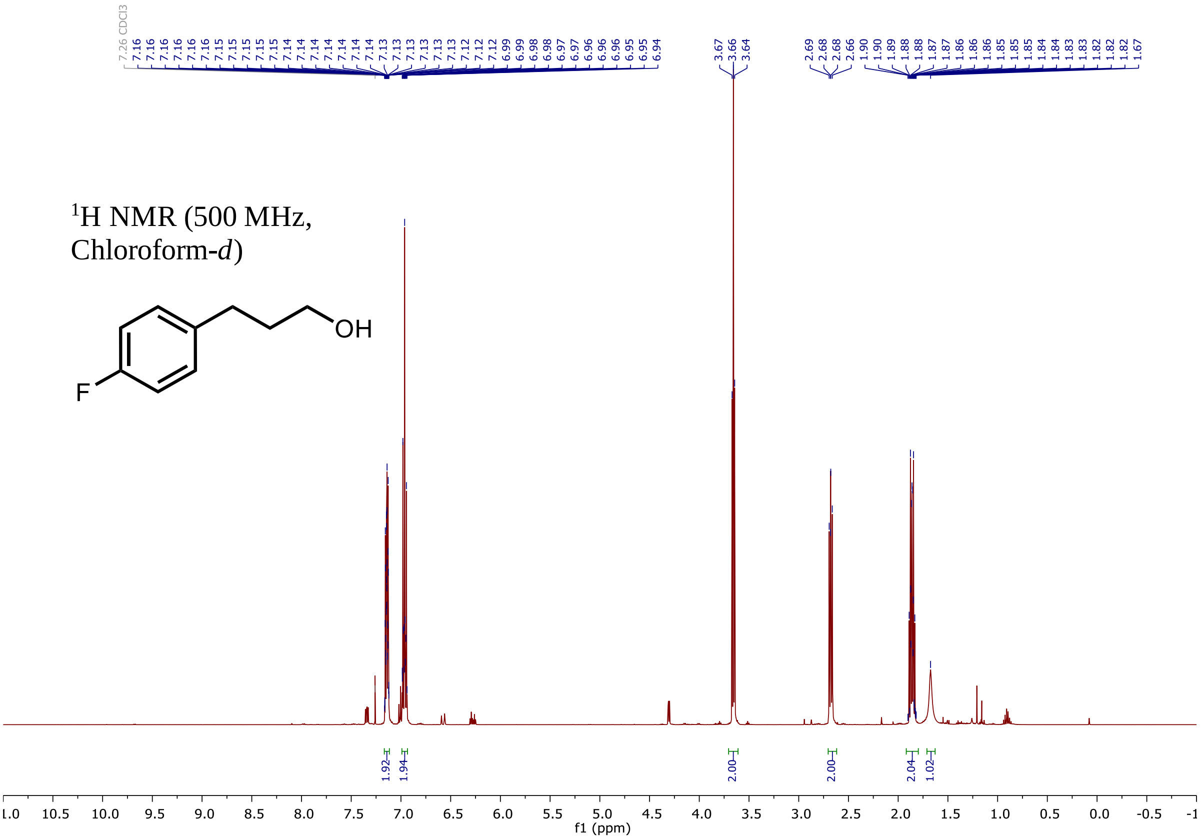

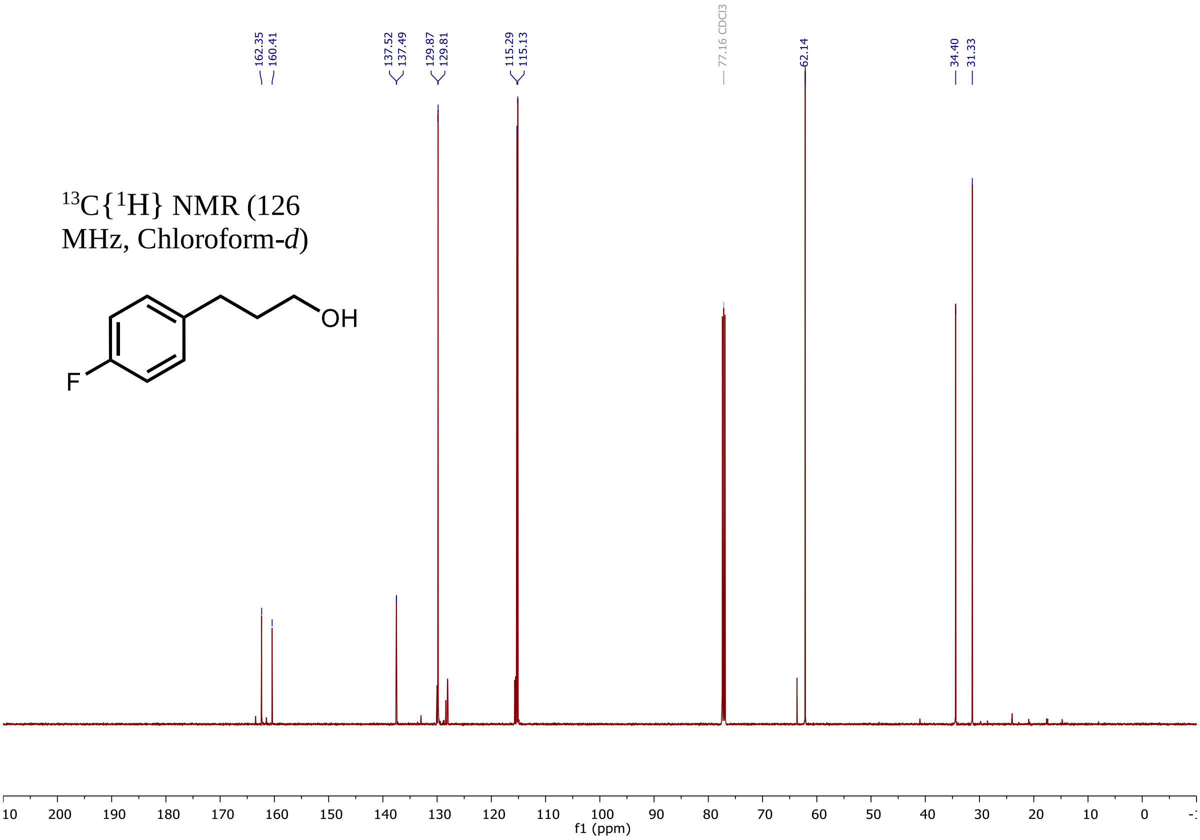

162.35
160.41
137.52
137.49
129.87
129.81
115.29
115.13
77.16 CDCl3
62.14
34.40
31.33
$^{13}C\{^{1}H\}$ NMR (126 MHz, Chloroform-*d*)
OH
F
f1 (ppm)

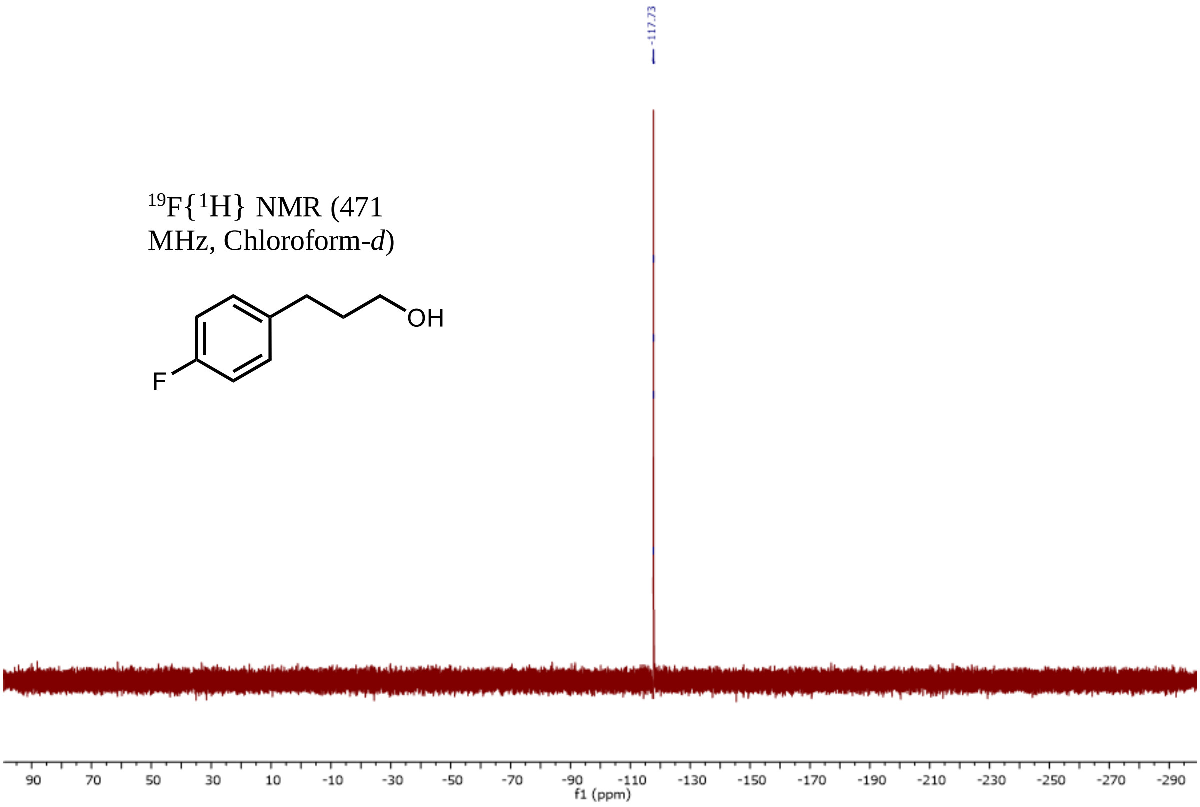

-117.73
$^{19}F\{^{1}H\}$ NMR (471 MHz, Chloroform-*d*)
OH
F
f1 (ppm)

3-(4-methoxyphenyl)propan-1-ol (33)

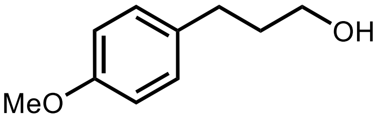

The title compound was prepared according to general procedure 7 using (*E*)-3-(4-methoxyphenyl)propan-1-ol (164 mg, 1 mmol) for 24 h and purified by flash silica chromatography (20% EtOAc in petroleum ether, 35 × 160 mm silica) to give the title compound as clear oil (125mg, 76% isolated yield, 88% NMR yield); R_f = 0.25 (20% EtOAc in petroleum ether); v_{max} / cm^{-1} (film) 3329, 2935, 1610, 1508, 130, 1242, 1176, 1031, 810, 518; ^1H NMR (500 MHz, Chloroform-*d*) δ 7.12 (d, *J* = 8.6 Hz, 2H), 6.84 (d, *J* = 9.4 Hz, 2H), 3.79 (s, 3H), 3.70 – 3.62 (m, 2H), 2.65 (d, *J* = 7.9 Hz, 2H), 1.86 (t, *J* = 6.3 Hz, 2H), 1.57 (s, 1H); $^{13}C\{^1H\}$ NMR (126 MHz, Chloroform-*d*) δ 157.9, 134.0, 129.4, 113.9, 62.3, 55.4, 34.5, 31.2; HRMS (EI-quadrupole) $(M + H - H_2O)^+$ Calcd for $C_{10}H_{13}O$ 149.0966; Found 149.0965.

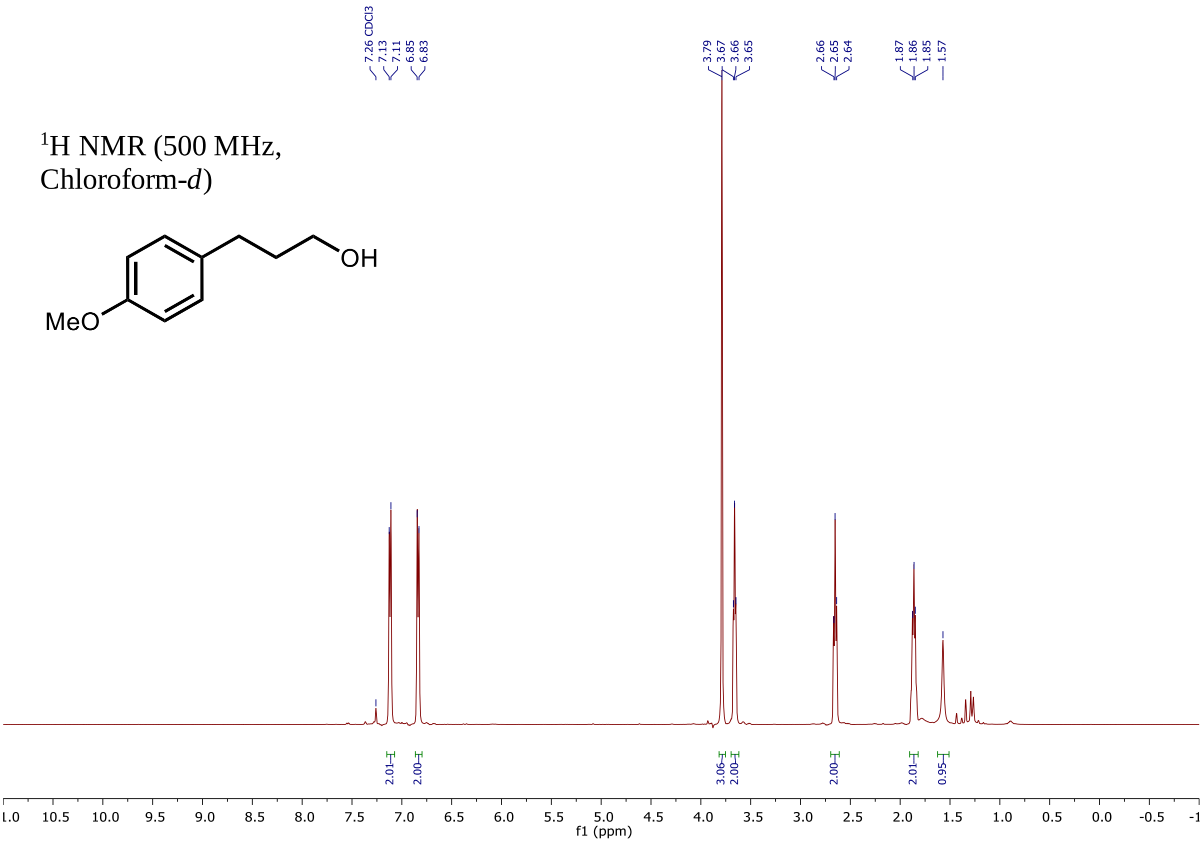

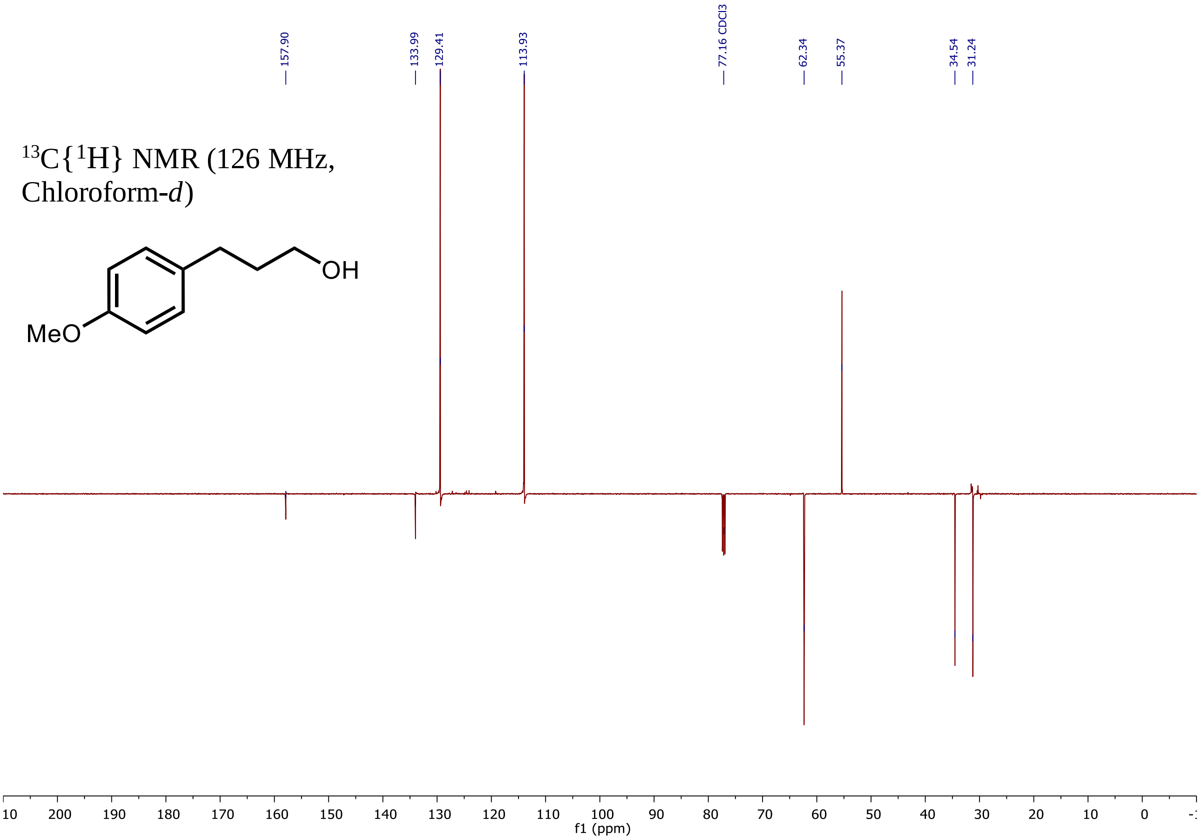

3-(4-(trifluoromethyl)phenyl)propan-1-ol (34)

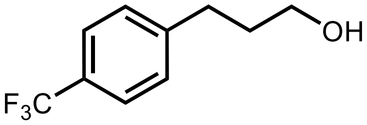

The title compound was prepared according to general procedure 7 using (*E*)-3-(4-(trifluoromethyl)phenyl)prop-2-en-1-ol (202 mg, 1 mmol) for 24 h and purified by flash silica chromatography (15-30% EtOAc in petroleum ether, 35 × 160 mm silica) to give the title compound as a yellow oil (96 mg, 47% isolated yield, 71% NMR yield); R_f = 0.31 (20% EtOAc in petroleum ether); ^{1}H NMR (500 MHz, Chloroform-*d*) δ 7.54 (d, *J* = 7.9 Hz, 2H), 7.31 (d, *J* = 7.9 Hz, 2H), 3.68 (t, *J* = 6.4 Hz, 2H), 2.81 – 2.74 (m, 2H), 1.95 – 1.85 (m, 2H), 1.53 (bs, 1H); ^{13}C{^{1}H} NMR (126 MHz, Chloroform-*d*) δ 146.1 (q, $J_{C\text{-}F}$ = 1.4 Hz), 128.9, 128.4 (q, $J_{C\text{-}F}$ = 32.2 Hz), 125.4 (q, $J_{C\text{-}F}$ = 3.8 Hz), 124.5 (q, $J_{C\text{-}F}$ = 272.2 Hz), 62.0, 34.0, 32.0; ^{19}F{^{1}H} NMR (471 MHz, Chloroform-*d*) δ -62.3. Spectroscopic data in accordance with that stated in the literature.[20]

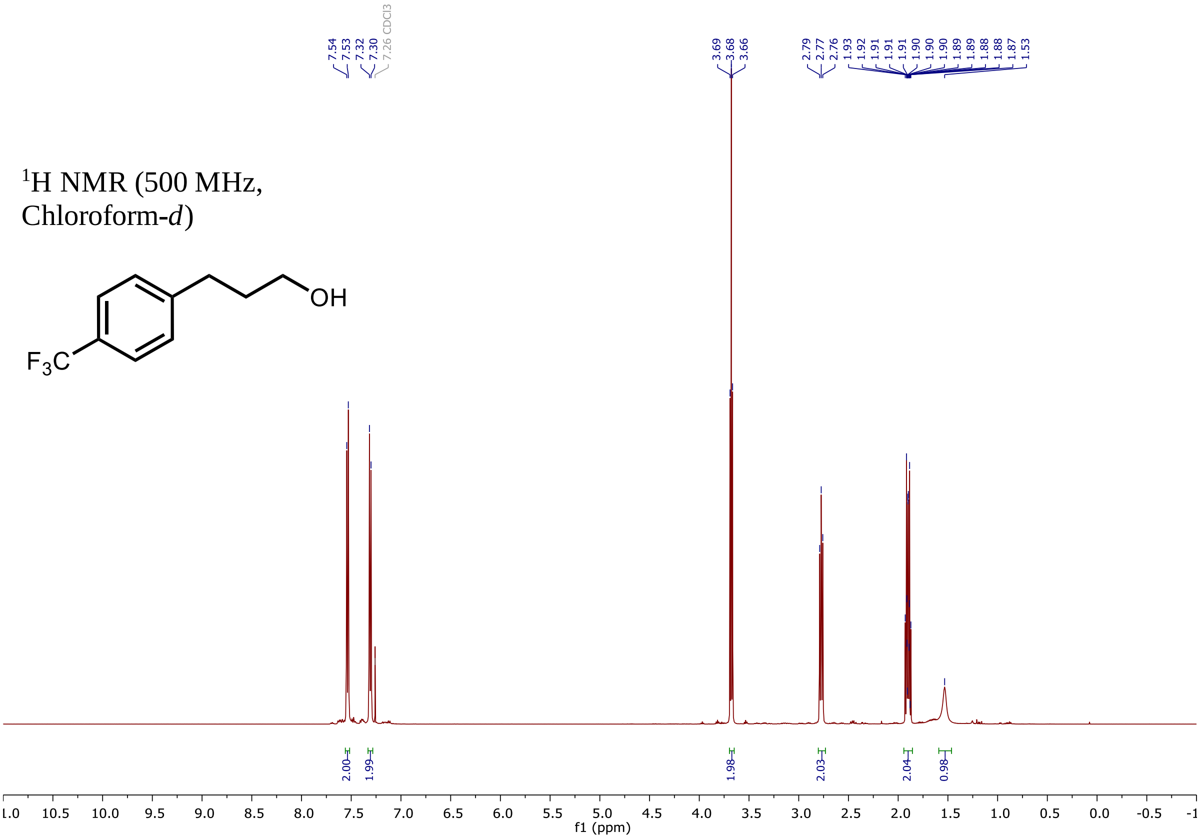

^{1}H NMR (500 MHz, Chloroform-d)
F3C
OH
7.54
7.53
7.32
7.30
7.26 CDCl3
3.69
3.68
3.66
2.79
2.77
2.76
1.93
1.92
1.91
1.91
1.91
1.90
1.90
1.90
1.89
1.89
1.88
1.88
1.87
1.53
2.00
1.99
1.98
2.03
2.04
0.98
f1 (ppm)

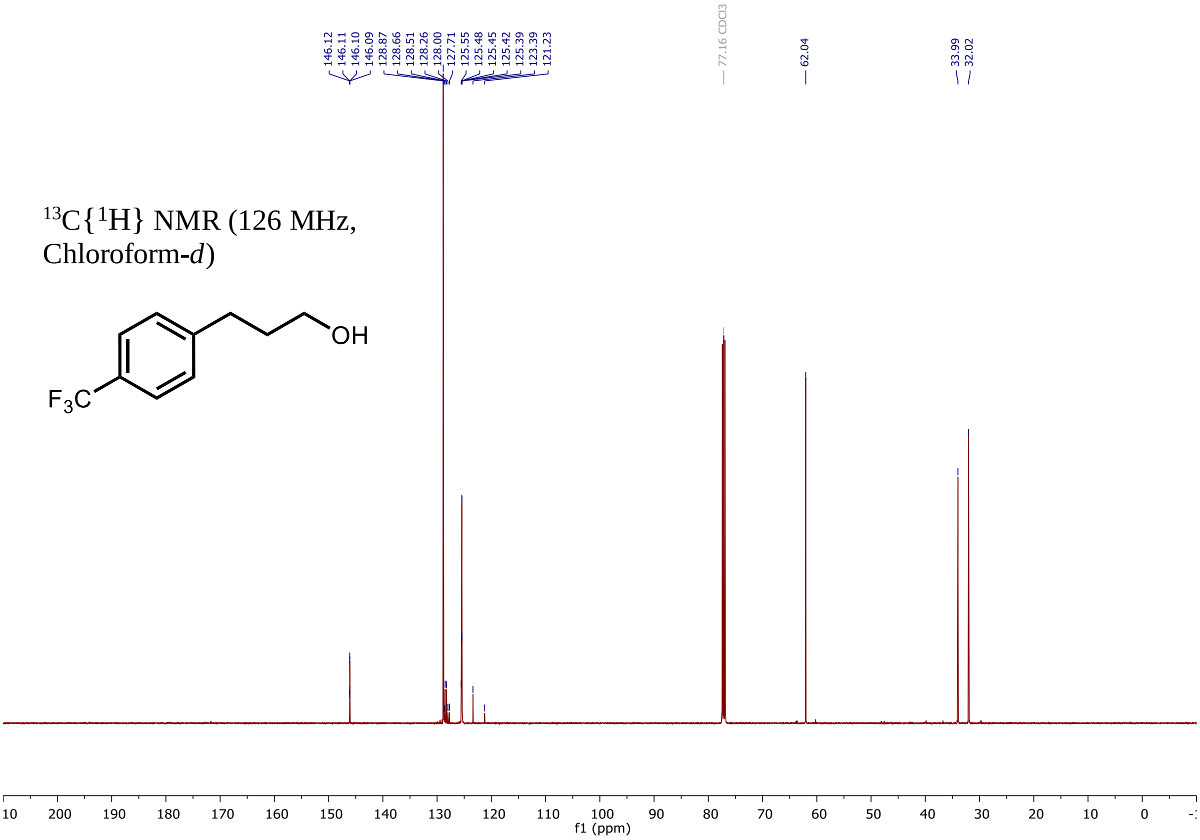

^{13}C{^{1}H} NMR (126 MHz, Chloroform-d)
F3C
OH
146.12
146.11
146.10
146.09
128.87
128.66
128.51
128.26
128.00
127.71
125.55
125.48
125.45
125.42
125.39
123.39
121.23
77.16 CDCl3
62.04
33.99
32.02
f1 (ppm)

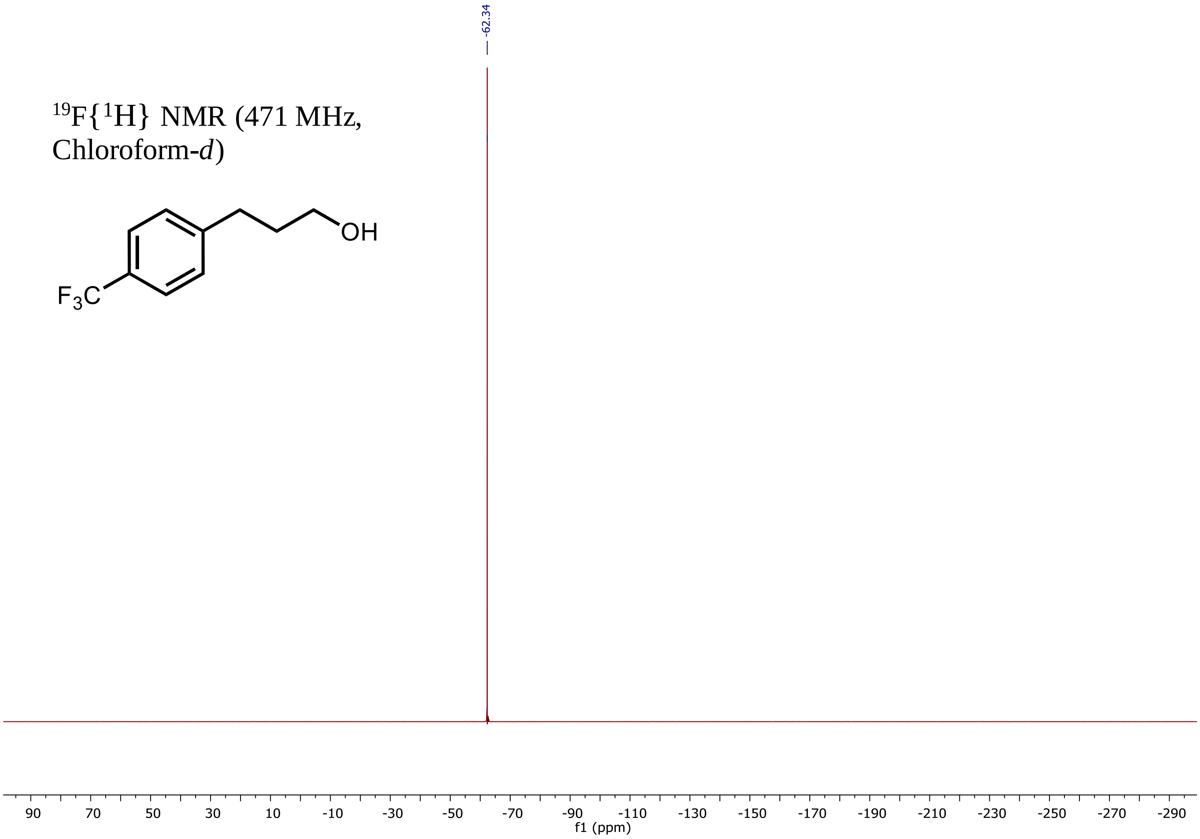

2-methylpropan-1-ol (35)

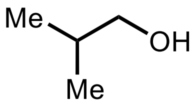

The title compound was prepared according to general procedure 7 using 2-methylprop-2-en-1-ol (85 µL, 72 mg, 1 mmol) for 24 h. Yield determined by crude ^{1}H NMR using 1,3,5-trimethylbenzene as internal standard: 62%.

^{1}H NMR (300 MHz, Chloroform-*d*)

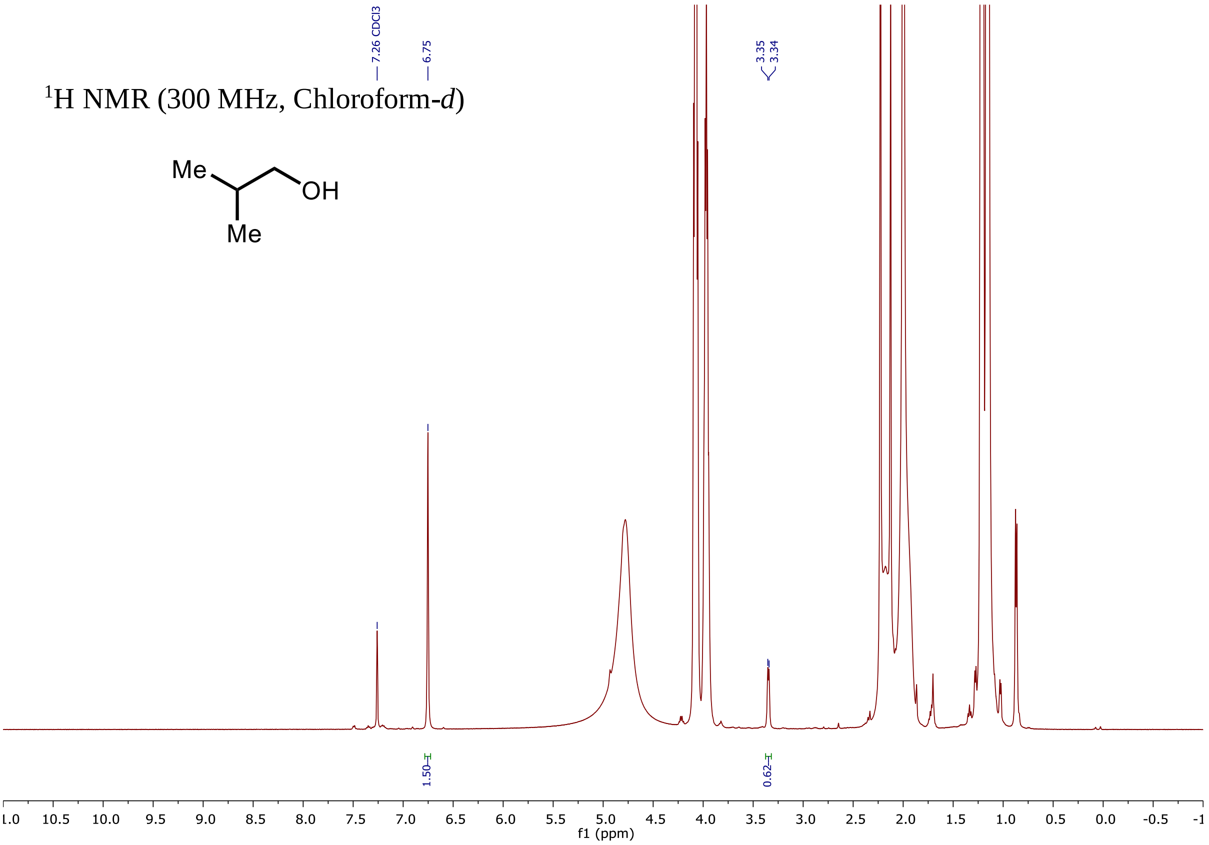

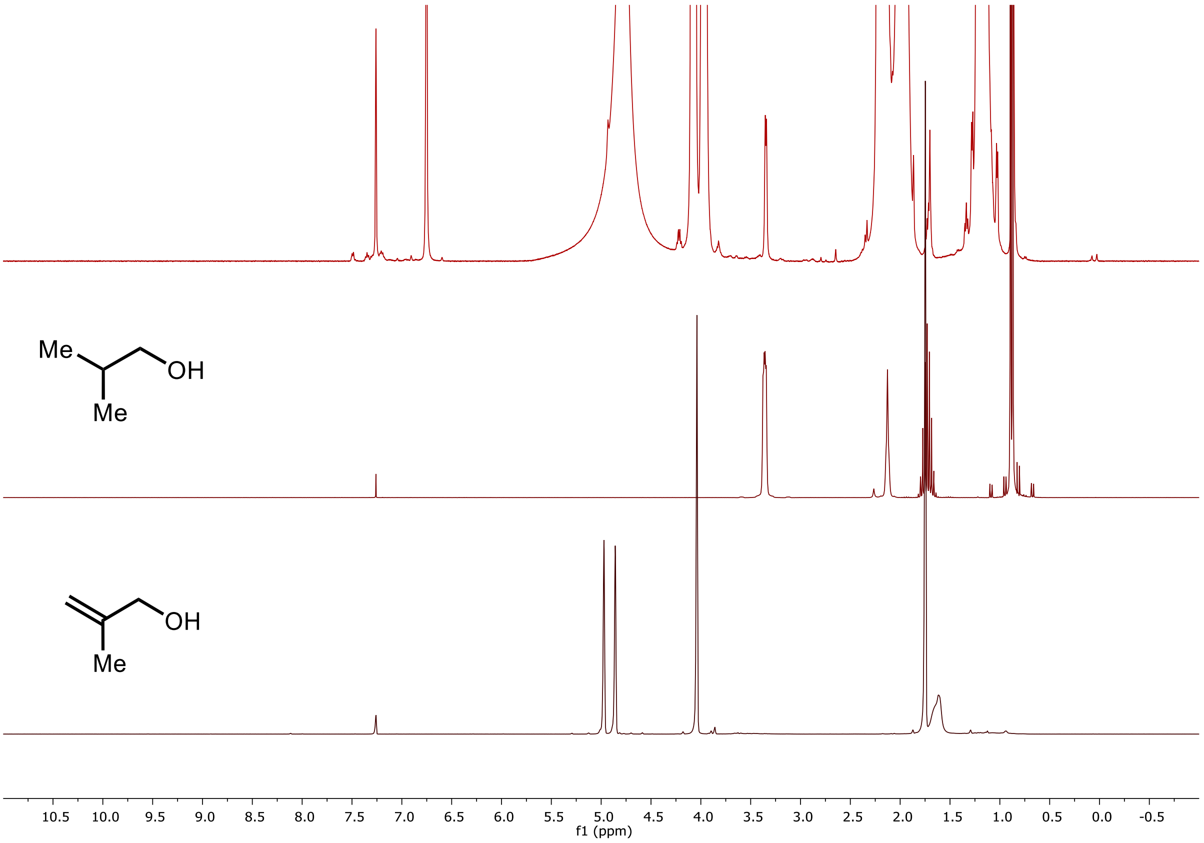

3-phenylbutan-1-ol (36)

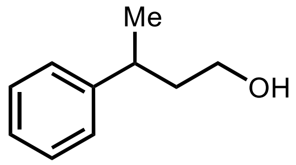

The title compound was prepared according to general procedure 7 using (*E*)-3-phenylbut-2-en-1-ol (74.1 mg, 0.5 mmol) for 48 h. Yield determined by crude ^{1}H NMR using 1,3,5-trimethylbenzene as internal standard: 24%

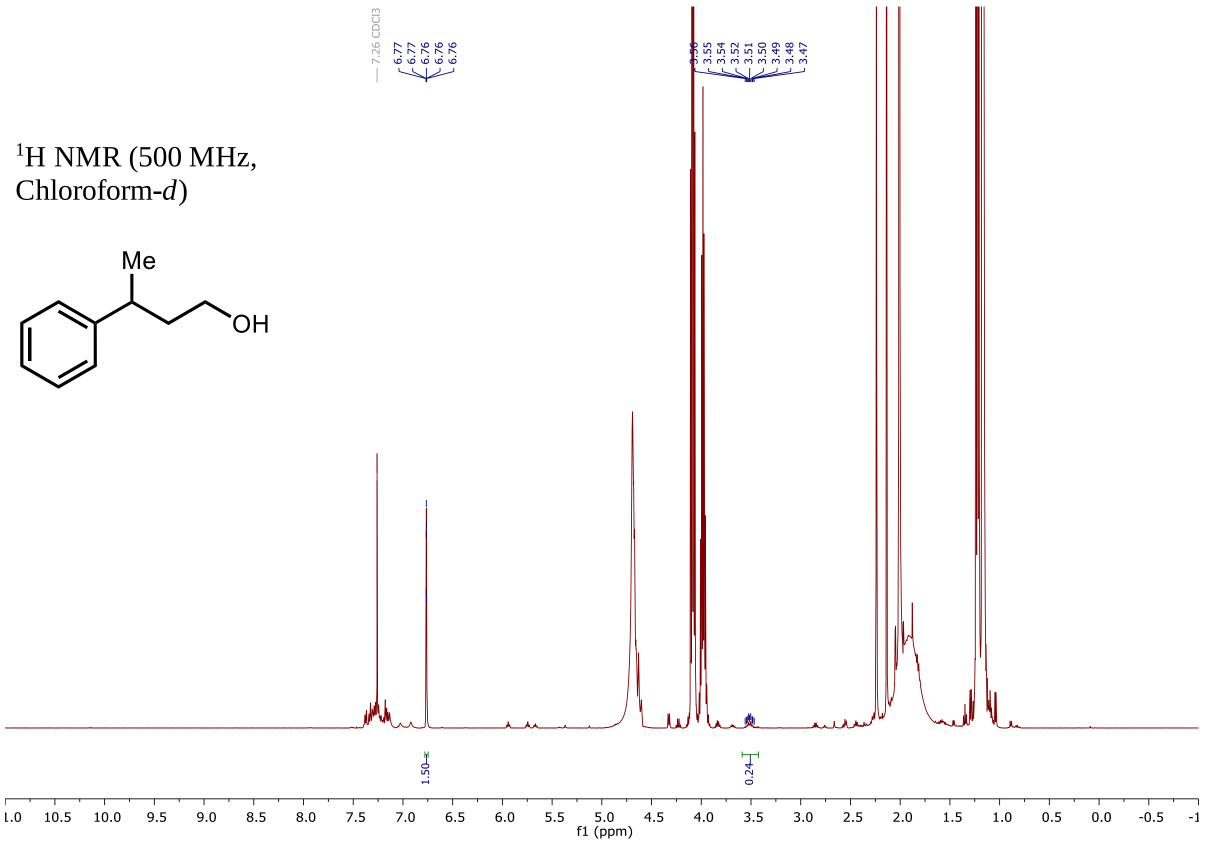

(*E*)-3,7-dimethylocta-2,6-dien-1-ol (37)

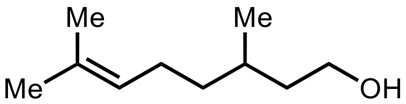

The title compound was prepared according to general procedure 7 using (*E*)-3-(4-iodophenyl)-2-methylprop-2-en-1-ol (274 mg, 1 mmol) for 24 h. Yield determined by crude ^{1}H NMR using 1,3,5-trimethylbenzene as internal standard: 61 %

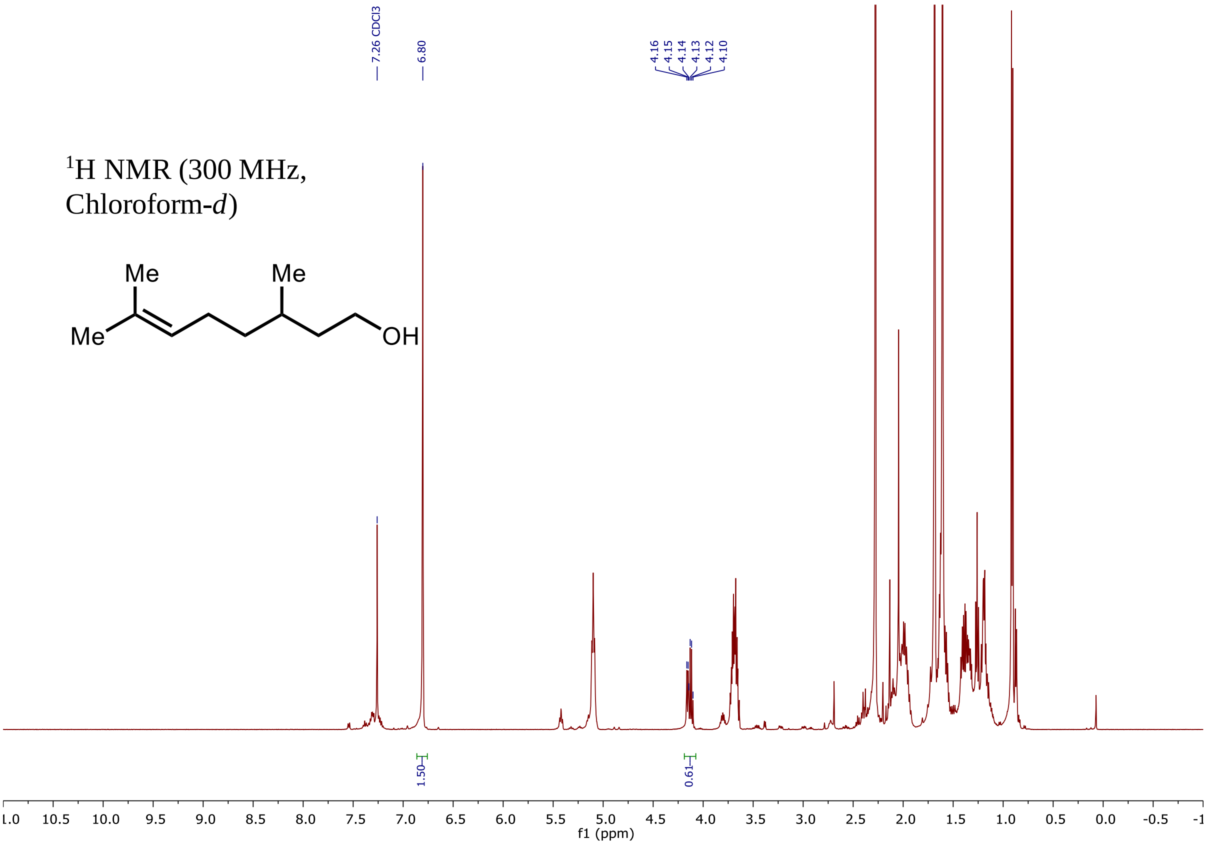

7.26 CDCl3
6.80
4.16
4.15
4.14
4.13
4.12
4.10
^{1}H NMR (300 MHz, Chloroform-*d*)
Me
Me
Me
OH
1.50
0.61
f1 (ppm)

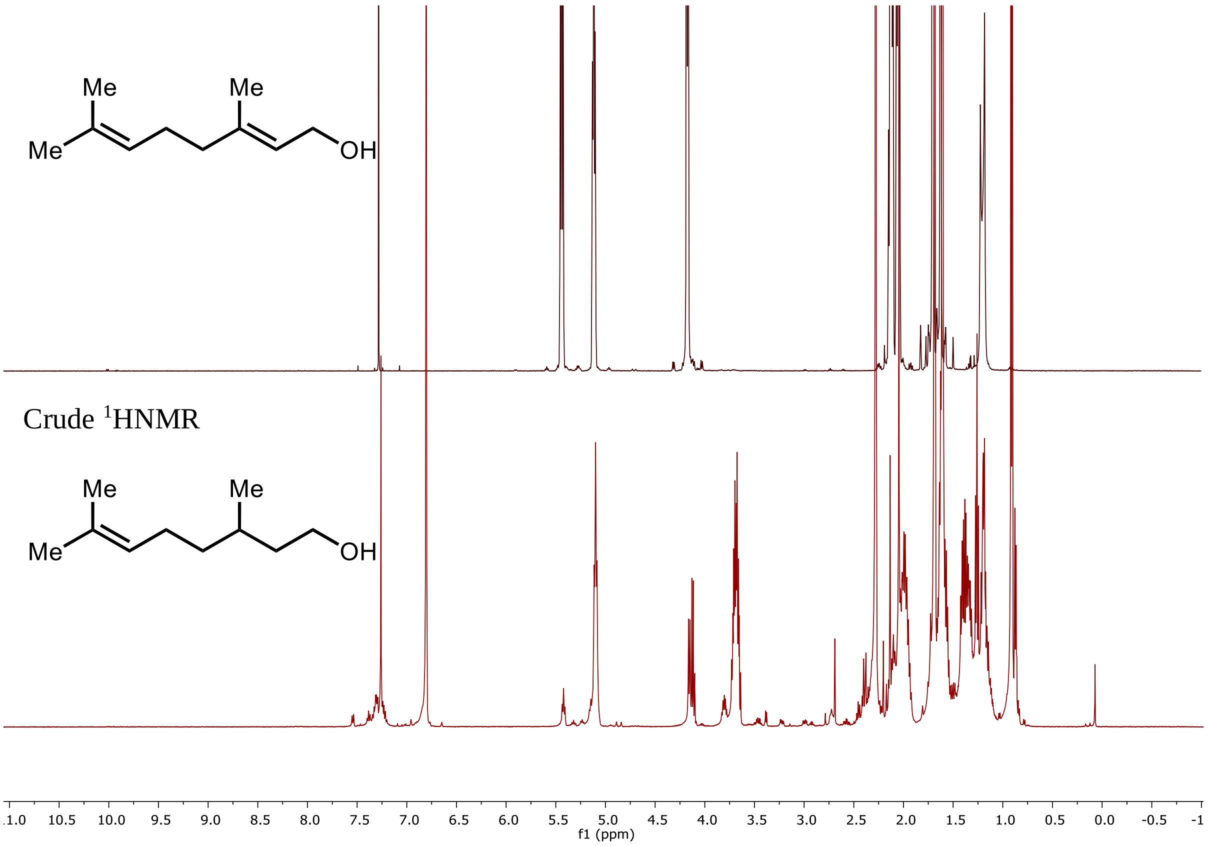

Me
Me
Me
OH
Crude ^{1}HNMR
Me
Me
Me
OH
f1 (ppm)

4-phenylbutan-2-ol (38)

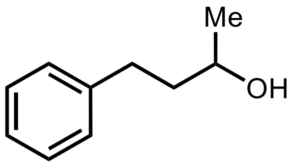

The title compound was prepared according to general procedure using (*E*)-4-phenylbut-3-en-2-ol (148 mg, 1 mmol) for 48h. Yield determined by crude ^{1}H NMR using 1,3,5-trimethylbenzene as internal standard: 76%

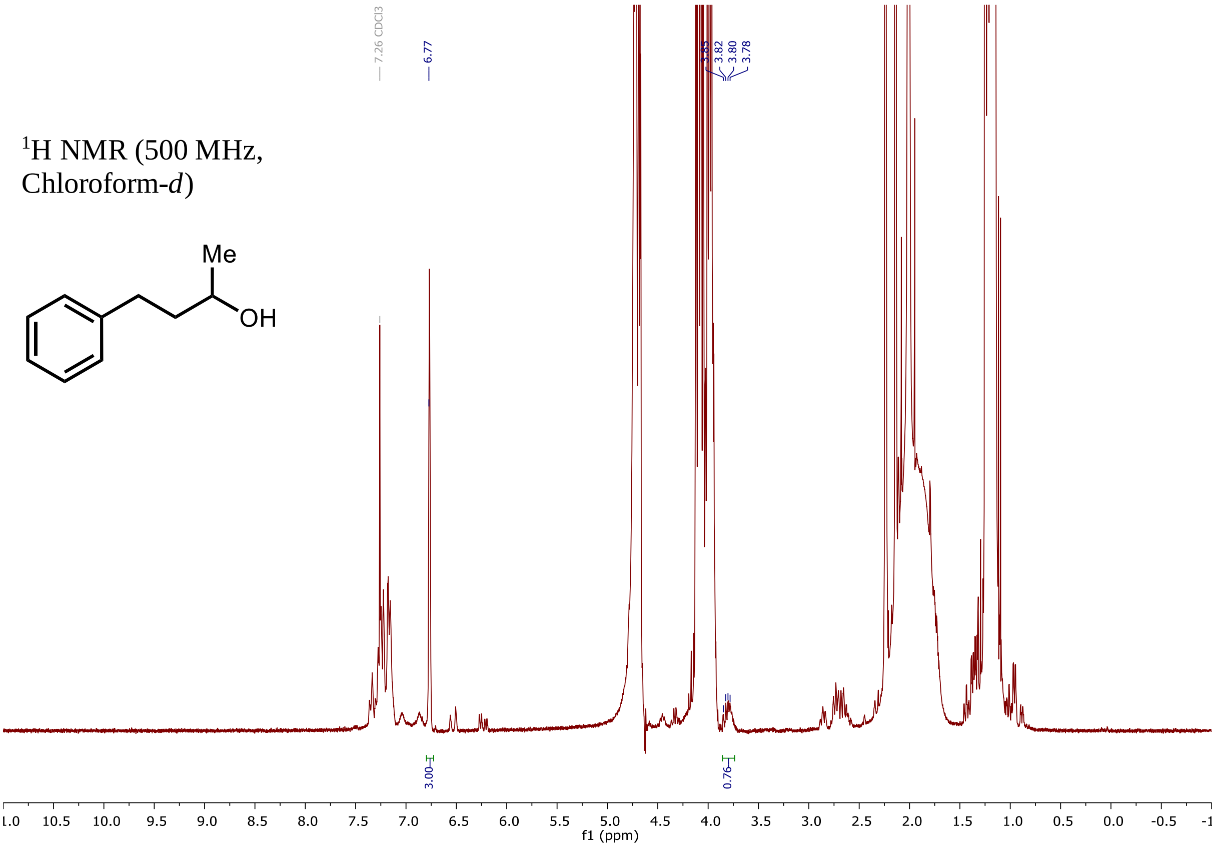

Isopropyl 4-(3-hydroxy-2-methylpropyl)benzoate (39)

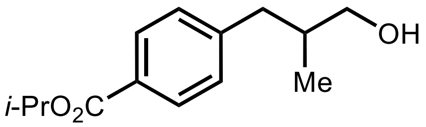

The title compound was prepared according to general procedure 7 using methyl (*E*)-4-(3-hydroxy-2-methylprop-1-en-1-yl)benzoate (103 mg, 0.5 mmol) for 24 h and purified by flash silica chromatography (15-20% EtOAc in petroleum ether, 35 × 160 mm silica) to give the title compound as a colourless oil (48.7 mg, 41% isolated yield, 88% NMR yield); R_f = 0.34 (30% EtOAc in petroleum ether); v_{max} / cm^{-1} (film) 3407, 2981, 2922, 2872, 1711, 1609, 1466, 1413, 1372, 1350, 1274, 1179, 1099, 1018, 918, 862, 755, 703; ^{1}H NMR (500 MHz, Chloroform-*d*) δ 7.99 – 7.91 (m, 2H), 7.27 – 7.20 (m, 2H), 5.24 (hept, *J* = 6.3 Hz, 1H), 3.58 – 3.44 (m, 2H),

2.84 (dd, J = 13.4, 6.1 Hz, 1H), 2.47 (dd, J = 13.4, 8.2 Hz, 1H), 2.02 – 1.91 (m, 1H), 1.58 (s, 1H), 1.36 (d, J = 6.3 Hz, 6H), 0.90 (d, J = 6.8 Hz, 3H); $^{13}C\{^{1}H\}$ NMR (126 MHz, Chloroform-*d*) δ 166.3, 146.5, 129.7, 129.2, 128.8, 68.3, 67.6, 39.8, 37.8, 22.1, 16.5; HRMS (EI-quadrupole) $(M)^+$ Calcd for $C_{14}H_{20}O_3$ 236.1407; Found 236.1407.

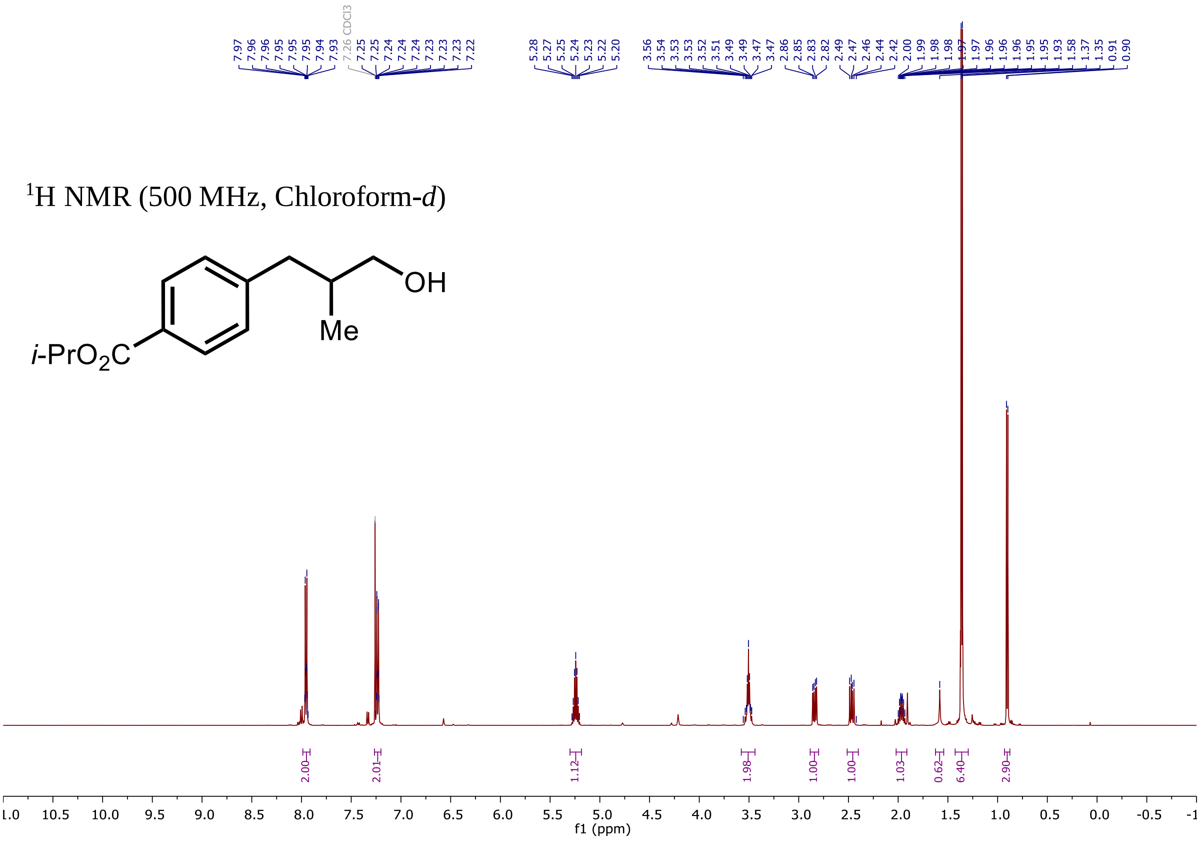

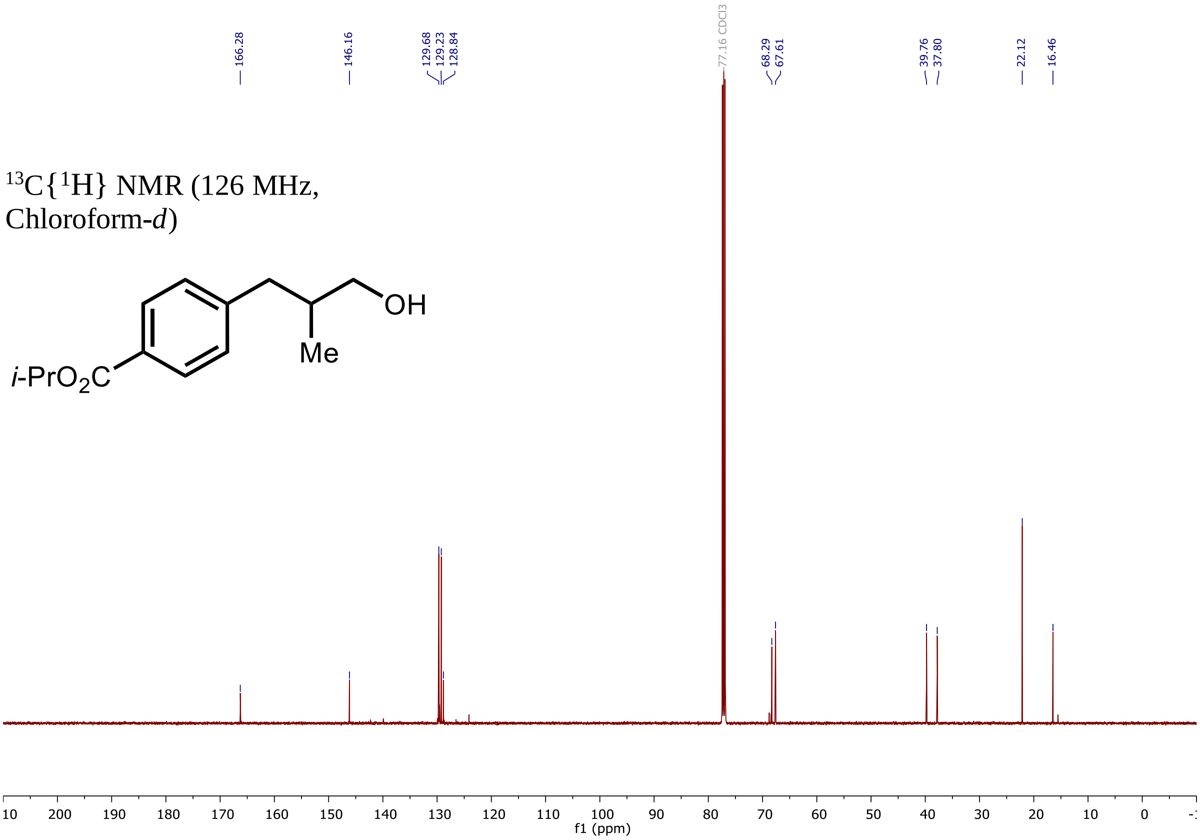

2.4. Validation of possible reaction intermediates

2.4.1. Synthesis of aldehyde (45)

2-methyl-3-phenylpropanal

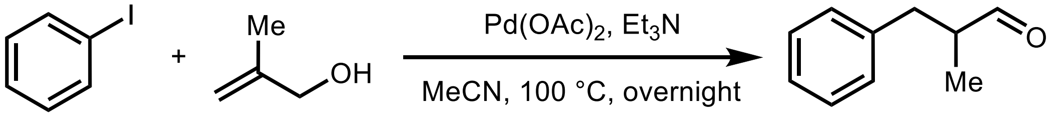

45

Iodobenzene (2.7 mL, 24.5 mmol, 1 equiv.), 2-methyl-2-propen-1-ol (2.6 mL, 30.6 mmol, 1.25 equiv.), $Pd(OAc)_2$ (110 mg, 0.49 mmol, 2 mol%) and triethylamine (4.3 mL, 30.6 mmol, 1.25 equiv.) were added to dry acetonitrile (98 mL) and the mixture was refluxed at 100 °C overnight. The reaction mixture was then allowed to cool and diluted with Et_2O:H_2O (1:1, 50 mL) and the organic layer was separated. The aqueous layer was extracted with Et_2O and the combined organic layers were washed with H_2O, dried over anhydrous $MgSO_4$, filtered and concentrated in vacuo. Purification by flash silica chromatography (eluent = 5% Et_2O in hexanes, 35 × 160 mm silica) gave the title compound as a yellow oil (558 mg, 15%); R_f = 0.17 (eluent = 5% Et_2O in hexanes); ν_{max} / cm^{-1} (film) 3028, 2976, 1703, 1495, 1454, 1234, 1176, 737, 698; ^1H NMR (500 MHz, Chloroform-*d*) δ 9.73 (d, *J* = 1.5 Hz, 1H), 7.34 – 7.26 (m, 2H), 7.22 (ddt, *J* = 8.3, 6.6, 1.4 Hz, 1H), 7.20 – 7.14 (m, 2H), 3.10 (dd, *J* = 13.5, 5.8 Hz, 1H), 2.74 – 2.62 (m, 1H), 2.61 (dd, *J* = 13.5, 8.2 Hz, 1H), 1.09 (d, *J* = 6.9 Hz, 3H); $^{13}C\{^1H\}$ NMR (126

MHz, Chloroform-*d*) δ 204.6, 139.1, 129.3, 128.8, 126.7, 48.3, 36.9, 13.5; HRMS (CI-quadrupole) $(M)^+$ Calcd for $C_{10}H_{12}O$ 148.0883; Found 148.0882.

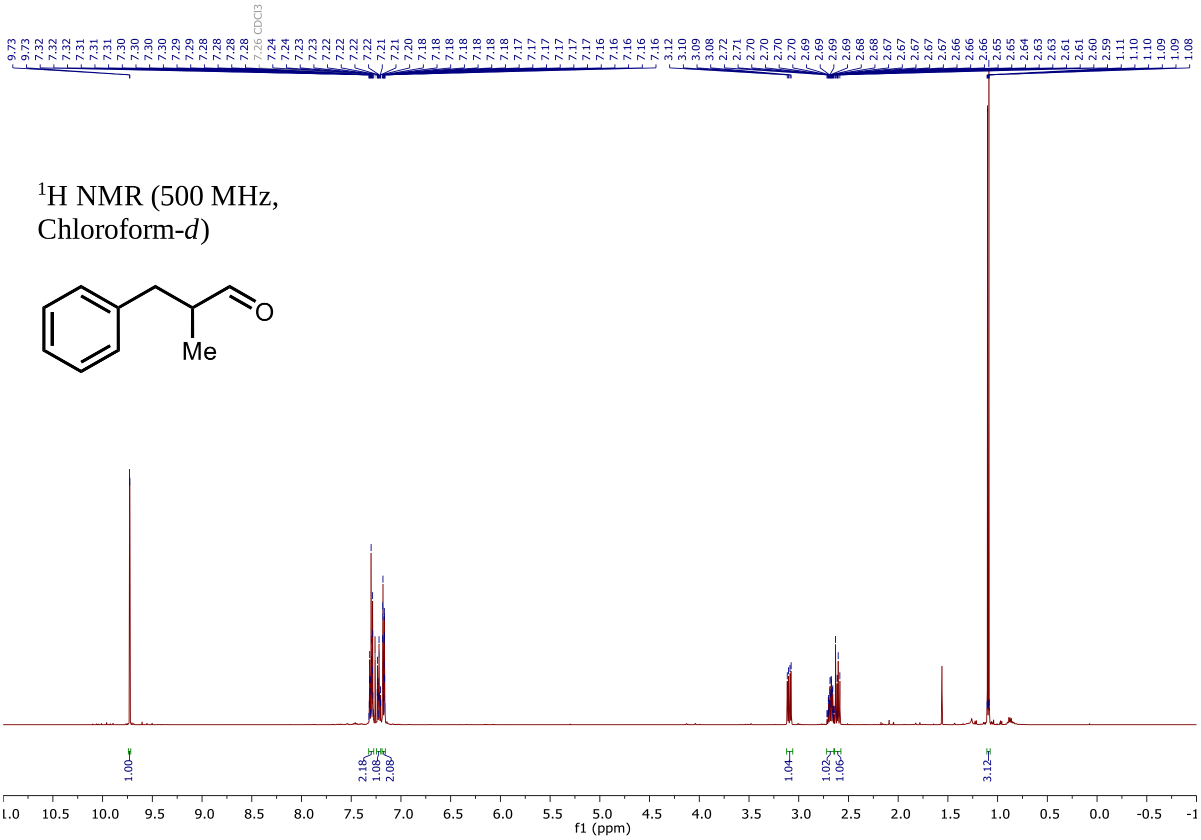

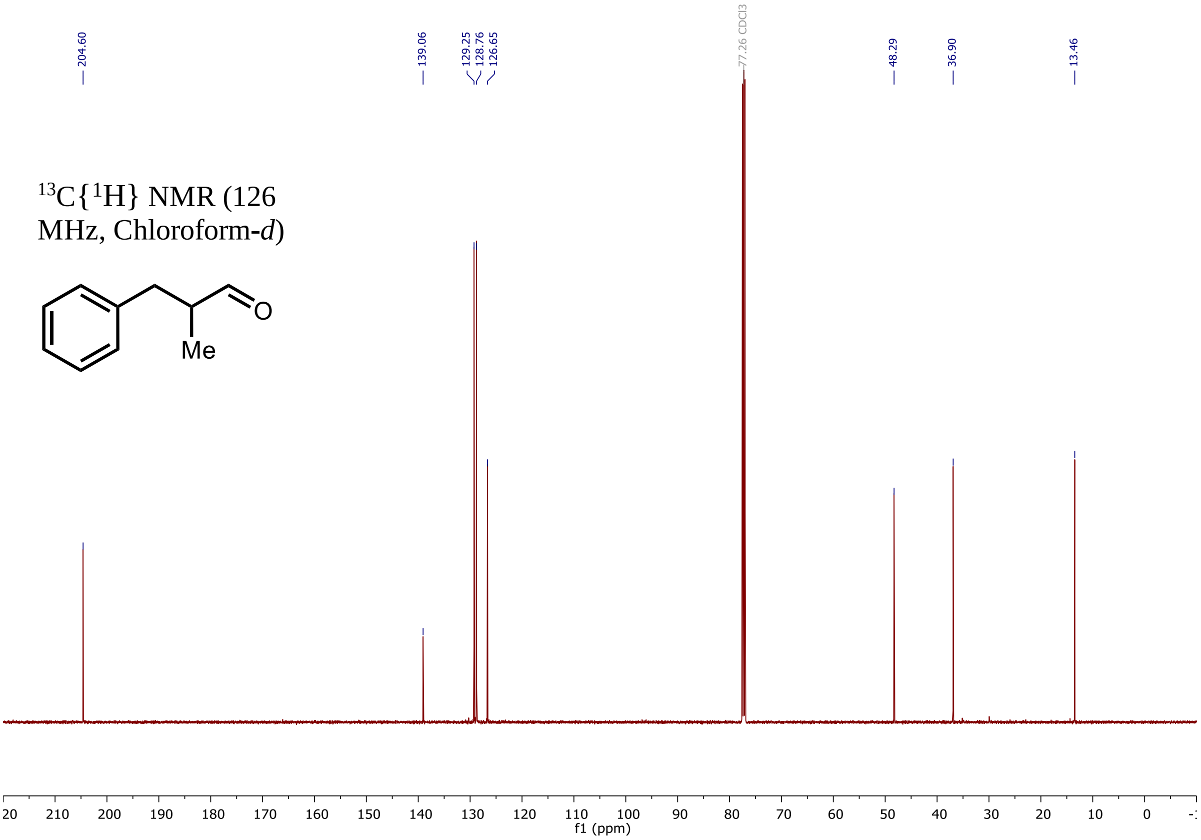

2.4.2. Mechanistic Experiments

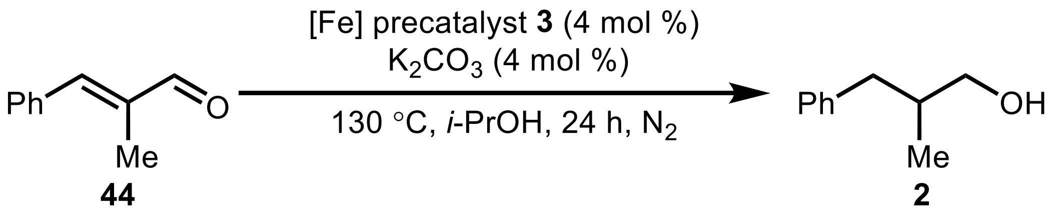

A 20 mL microwave vial with a magnetic stirrer bar was charged with potassium carbonate (5.52 mg, 0.04 mmol, 4 mol%), 2-methylcinnamaldehyde (146 µL, 146 mg, 1 mmol) and [Fe] precatalyst **3** (18 mg, 0.04 mmol, 4 mol%). The vial was firmly sealed with a cap and was placed under vacuum. After 5 minutes, the vial was flushed with nitrogen and the cycle was repeated three times. Under nitrogen, the vial was then charged with dry isopropanol (2 mL), placed on a preheated heating block at 130 °C and stirred for 24 h. The mixture was then cooled followed by the addition of mesitylene (139 µL, 1.00 mmol) and it was stirred for 2 minutes. Saturated aqueous NH_4Cl (2 mL) was then added and the mixture was stirred for further 2 minutes, followed by the addition of EtOAc (2 mL) and stirring for 2 minutes. Then the mixture was left to settle for a 5 minutes, the top layer was sampled and analysed using ^{1}H NMR. Yield determined by crude ^{1}H NMR using 1,3,5-trimethylbenzene as internal standard: 90% of **2**.

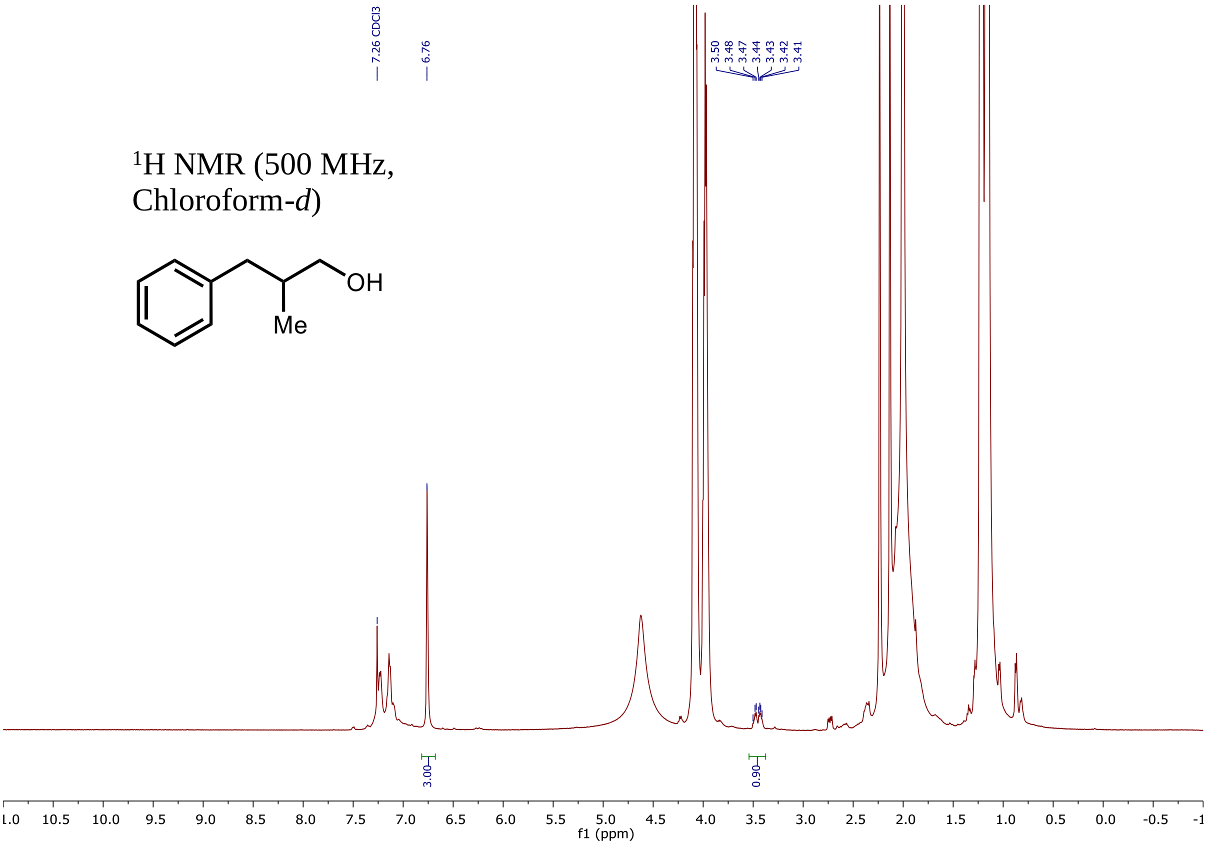

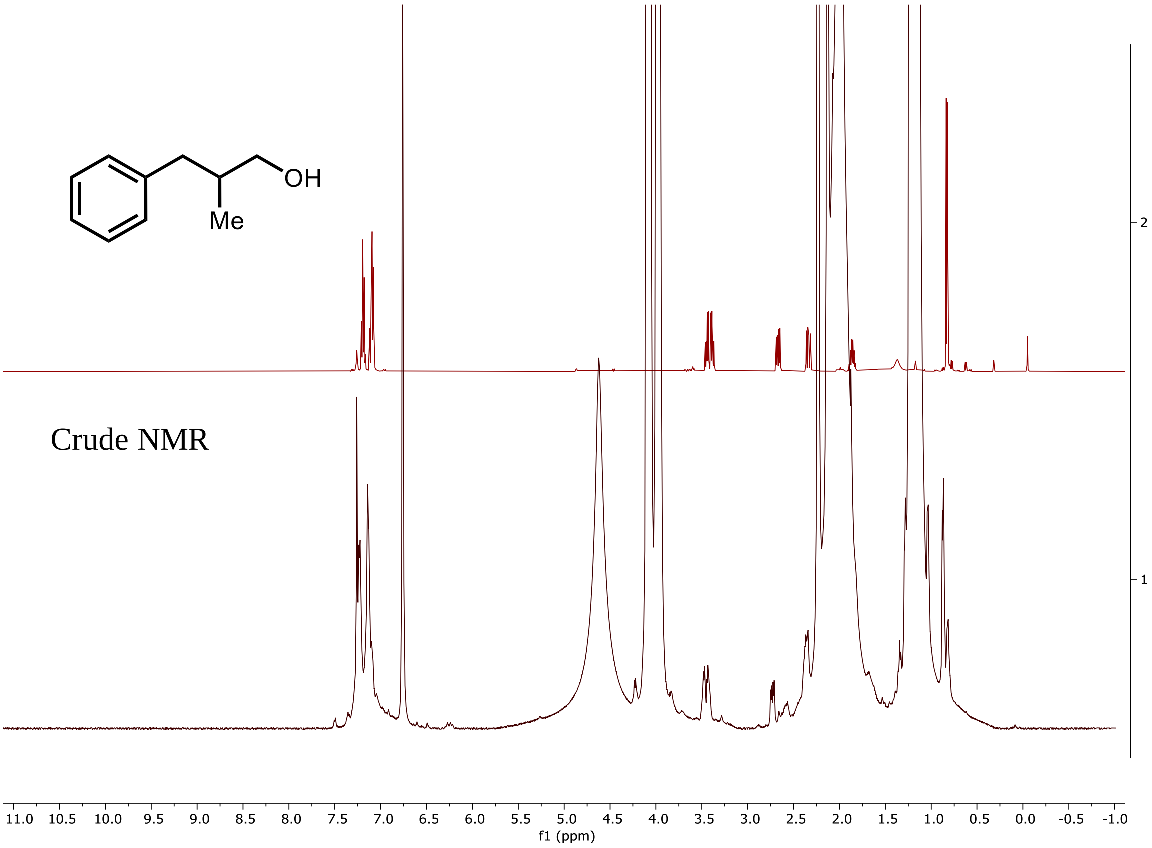

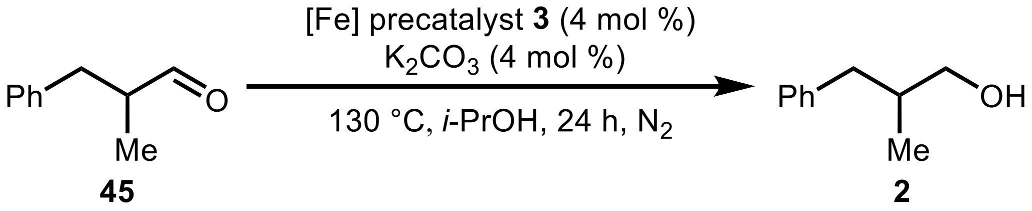

A 20 mL microwave vial with a magnetic stirrer bar was charged with potassium carbonate (5.52 mg, 0.04 mmol, 4 mol%), 2-methyl-3-phenylpropanal (148 mg, 1 mmol) and [Fe] precatalyst (18 mg, 0.04 mmol, 4 mol%). The vial was firmly sealed with a cap and was placed under vacuum. After 5 minutes, the vial was flushed with nitrogen and the cycle was repeated three times. Under nitrogen, the vial was then charged with dry isopropanol (2 mL), placed on a preheated heating block at 130 °C and stirred for 24 h. The mixture was then cooled followed by the addition of mesitylene (139 µL, 1.00 mmol) and it was stirred for 2 minutes. Saturated aqueous NH_4Cl (2 mL) was then added and the mixture was stirred for further 2 minutes, followed by the addition of EtOAc (2 mL) and stirring for 2 minutes. Then the mixture was left to settle for a 5 minutes, the top layer was sampled and analysed using ^{1}H NMR. Yield determined by crude ^{1}H NMR using 1,3,5-trimethylbenzene as internal standard: 43% of **2**.

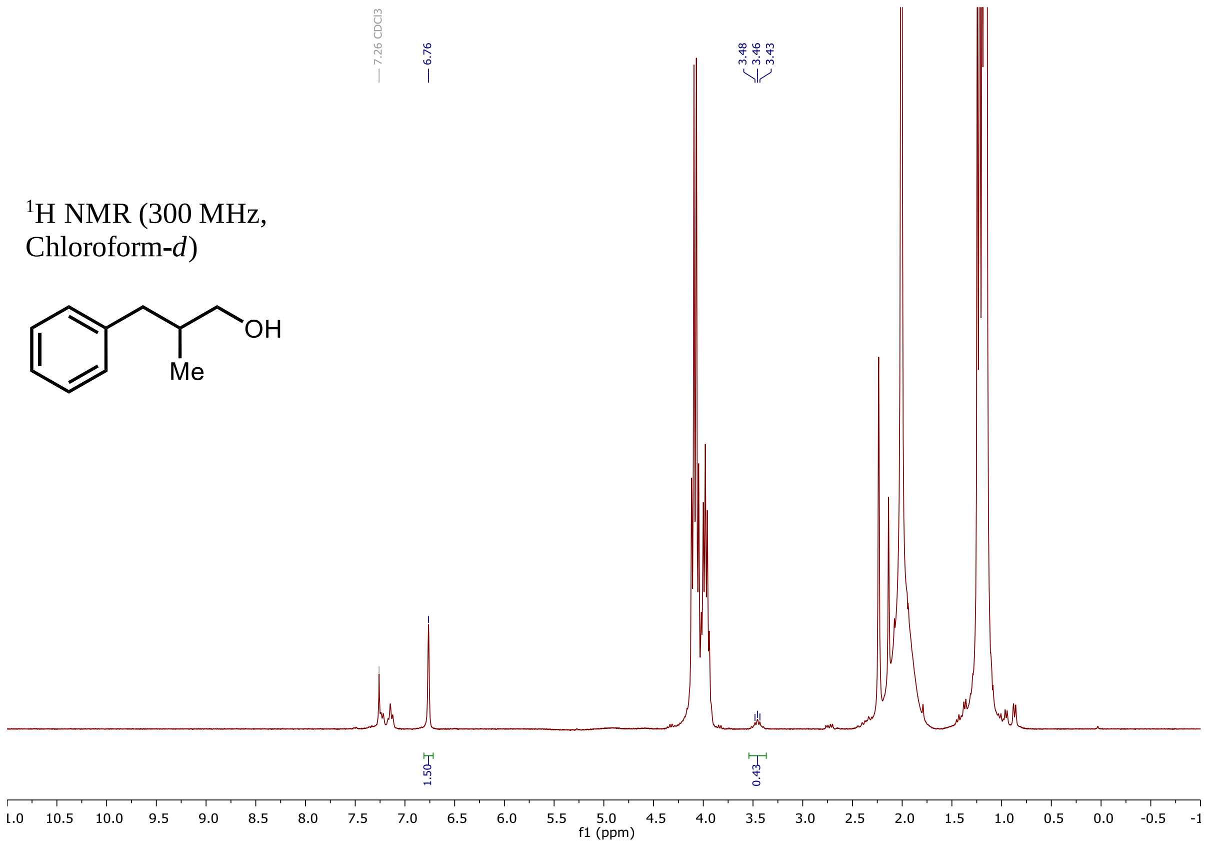

^{1}H NMR (300 MHz, Chloroform-d)
OH
Me
7.26 CDCl3
6.76
3.48
3.46
3.43
1.50
0.43
f1 (ppm)

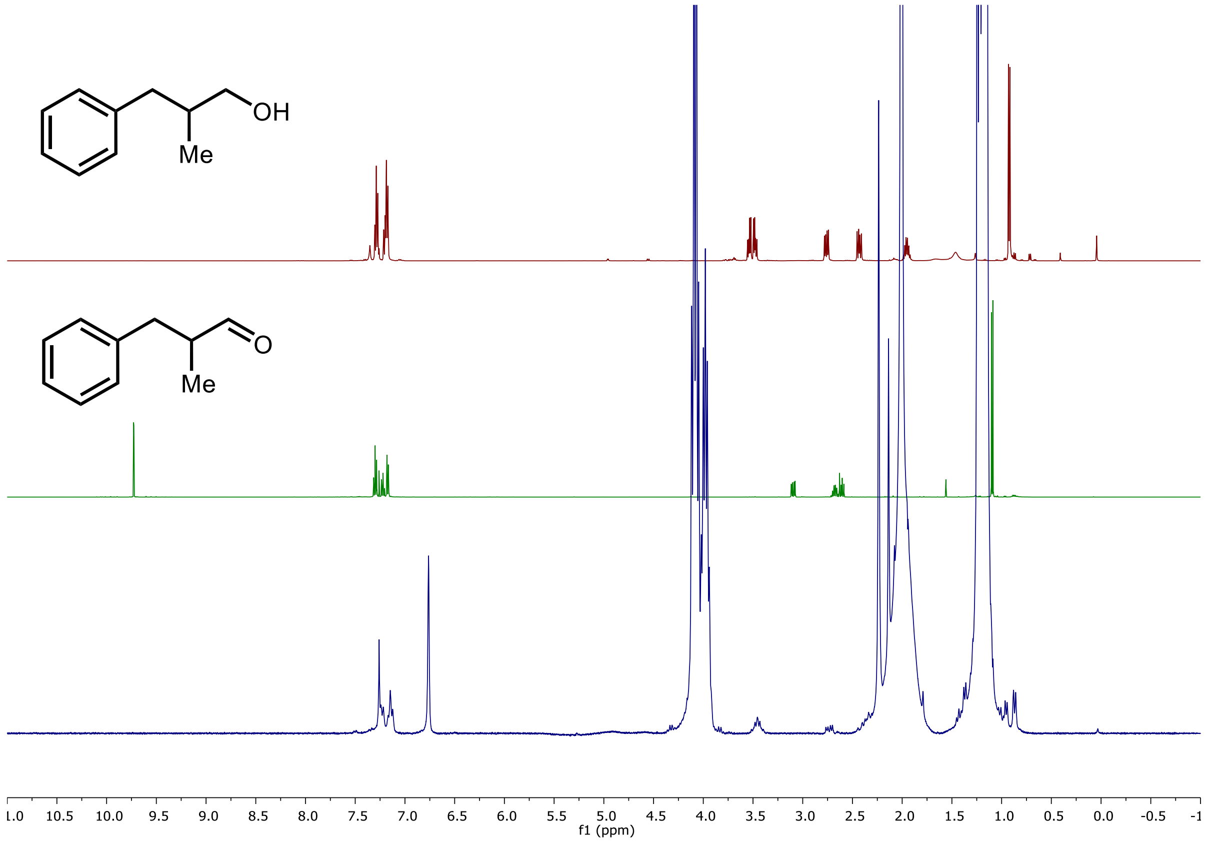

OH
Me
O
Me
f1 (ppm)

2.5. Kinetic time course experiments

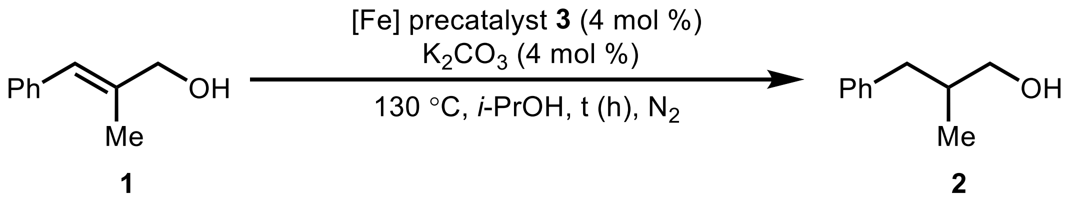

A 20 mL microwave vial with a magnetic stirrer bar was charged with potassium carbonate (5.52 mg, 0.04 mmol, 4 mol%), 2-methyl-3-phenylpropan-1-ol (144 µL, 148 mg, 1 mmol) and [Fe] precatalyst **3** (18 mg, 0.04 mmol, 4 mol %). The vial was firmly sealed with a cap and was placed under vacuum. After 5 minutes, the vial was flushed with nitrogen and the cycle was repeated three times. Under nitrogen, the vial was then charged with dry isopropanol (2 mL), placed on a preheated heating block at 130 °C and stirred for 18 or 24 h. The mixture was then cooled and the cap was removed followed by the addition of mesitylene (139 µL, 120 mg, 1.00 mmol) and it was stirred for 2 minutes. Saturated aqueous NH_4Cl (2 mL) was then added and the mixture was stirred for further 2 minutes, followed by the addition of EtOAc (2 mL) and stirring for 2 minutes. Then the mixture was left to settle for 5 minutes, the top layer was sampled and analysed using ^{1}H NMR.

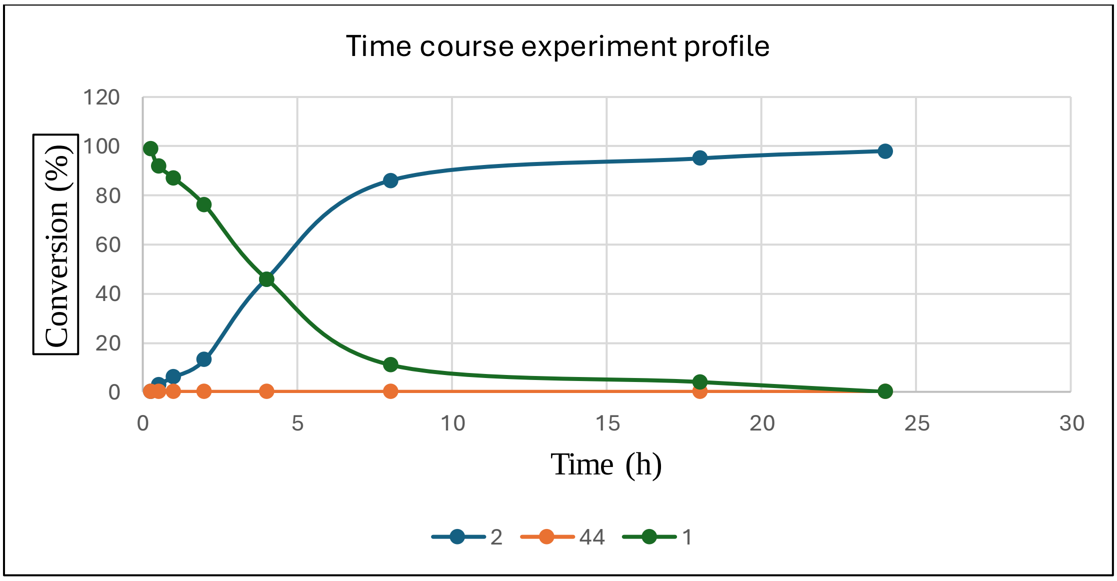

Time (h)	0.25	0.5	1	2	4	8	18	24
**2** (%)	0	3	6	13	46	86	95	99

**44** (%)	0	<2	<2	<2	<2	<2	<2	0
**1** (%)	99	92	87	76	46	11	4	0

2.6. Deuterium labelling experiments

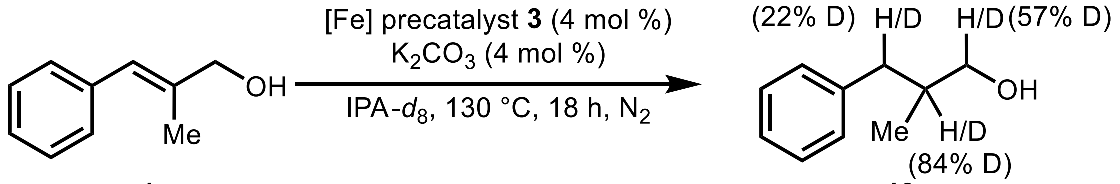

1 **46**

A 20 mL microwave vial with a magnetic stirrer bar was charged with potassium carbonate (2.8 mg, 0.04 mmol, 4 mol%), 2-methyl-3-phenylprop-2-en-1-ol (74 mg, 0.5 mmol) and [Fe] precatalyst **3** (9 mg, 0.04 mmol, 4 mol %). The vial was sealed with a cap and was placed under vacuum. After 5 minutes, the vial was flushed with nitrogen and the cycle was repeated three times. Under nitrogen, the vial was then charged with isopropanol-d_8 (1 mL), placed on a preheated heating block at 130 °C and stirred for 24 h. The mixture was then cooled and the cap was removed followed by the addition of mesitylene (70 µL, 60 mg, 1.00 mmol) and it was stirred for 2 minutes. Saturated aqueous NH_4Cl (1 mL) was then added and the mixture was stirred for further 2 minutes, followed by the addition of EtOAc (1 mL) and stirring for 2 minutes. Then the mixture was left to settle for 5 minutes, the top layer was sampled and analysed using ^{1}H NMR. Yield determined by crude ^{1}H NMR using 1,3,5-trimethylbenzene as internal standard: 54%

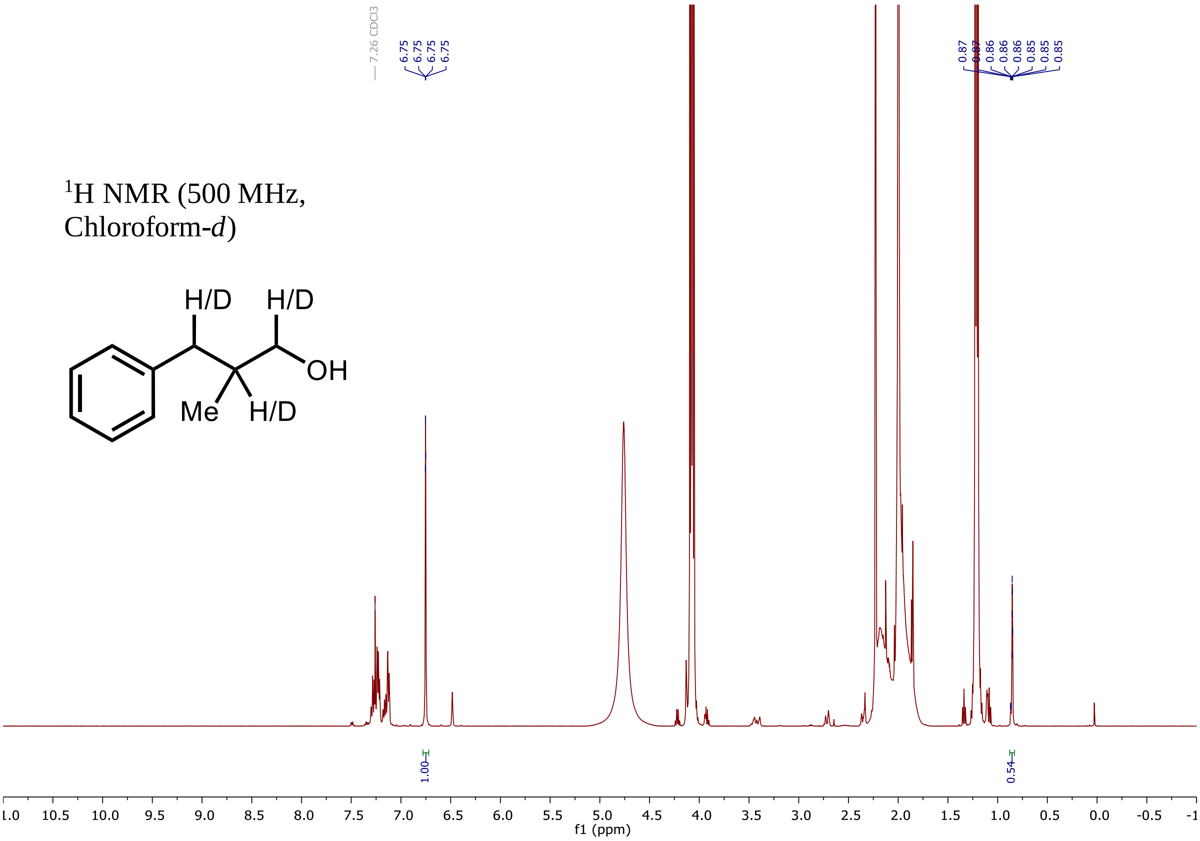

The mixture was diluted with EtOAc (12 mL), and transferred to a separatory funnel containing brine (12 mL). The aqueous phase was extracted with EtOAc (2 × 12 mL). The combined organic layers were dried over $MgSO_4$, filtered and concentrated in vacuo. Deuterium incorporation was determined by ^{1}H NMR after purification by flash silica chromatography (15-20% EtOAc in petroleum ether, 35 × 160 mm silica).

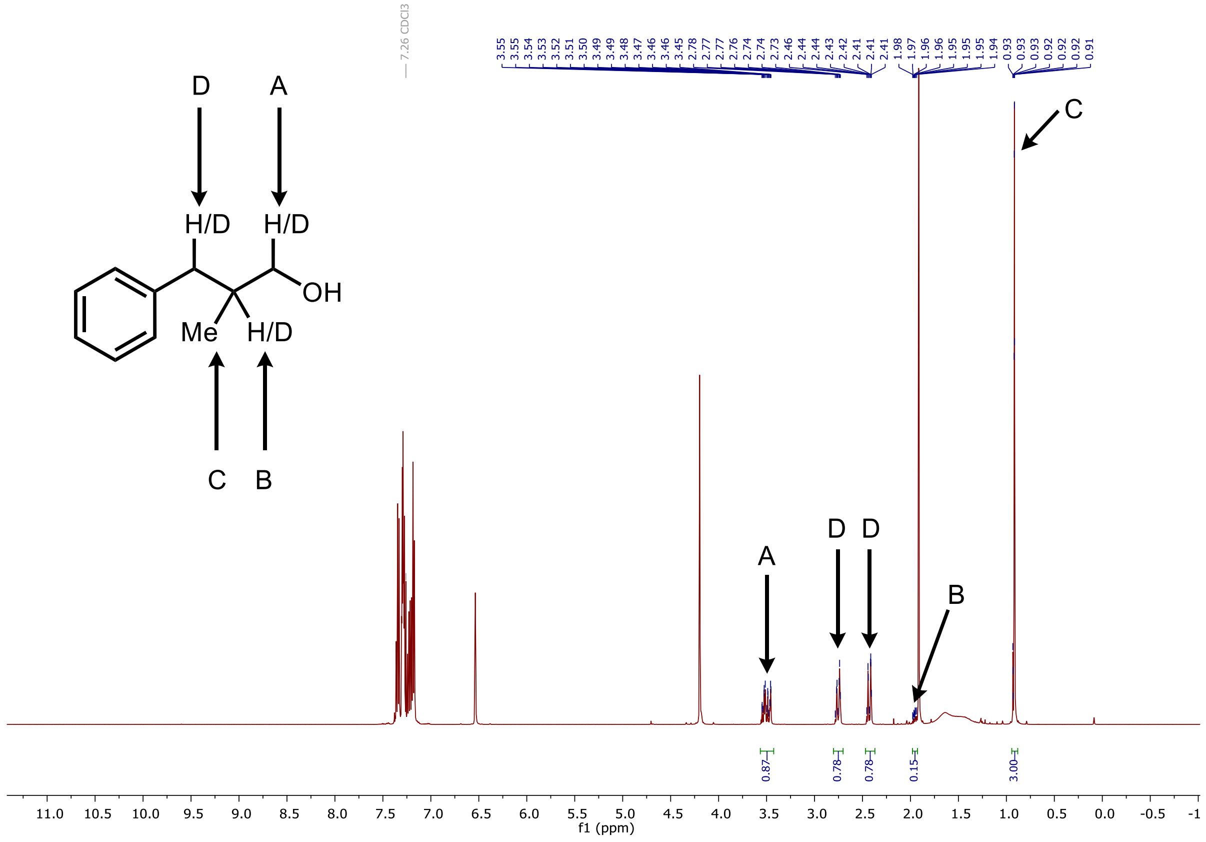
7.26 CDCl3
3.55 3.55 3.54 3.53 3.52 3.51 3.50 3.49 3.49 3.48 3.47 3.46 3.46 3.45 2.78 2.77 2.77 2.76 2.74 2.74 2.73 2.46 2.44 2.44 2.43 2.42 2.41 2.41 2.41 1.98 1.97 1.96 1.96 1.95 1.95 1.95 1.94 0.93 0.93 0.93 0.92 0.92 0.92 0.91
D
A
H/D
H/D
OH
Me
H/D
C
B
A
D
D
B
C
0.87
0.78
0.78
0.15
3.00
f1 (ppm)

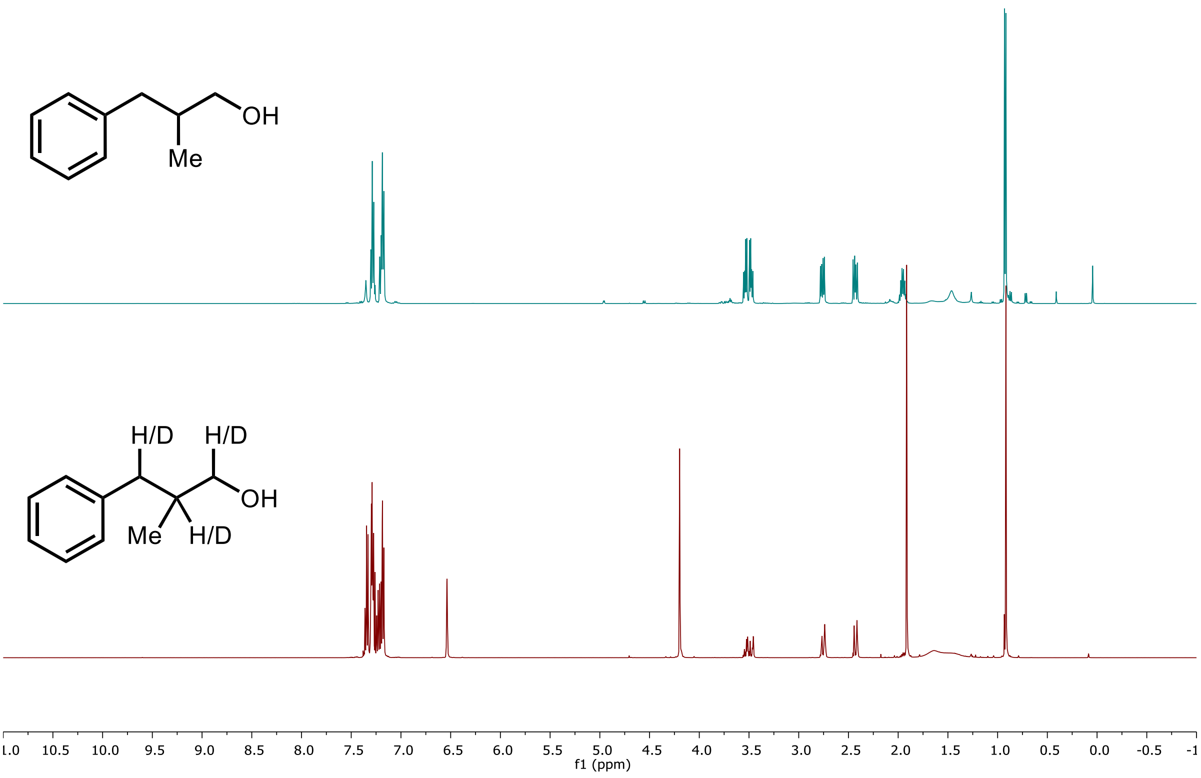
OH
Me
H/D
H/D
OH
Me
H/D
f1 (ppm)

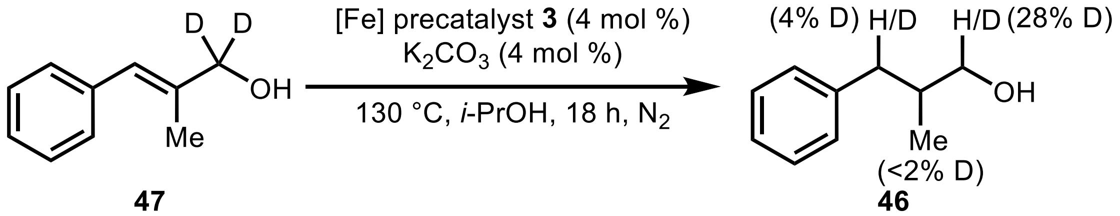

A 20 mL microwave vial with a magnetic stirrer bar was charged with potassium carbonate (2.8 mg, 0.04 mmol, 4 mol%), 2-methyl-3-phenylprop-2-en-1,1-*d*$_2$-1-ol (75 mg, 0.5 mmol) and [Fe] precatalyst **2** (9 mg, 0.04 mmol, 4 mol %). The vial was sealed with a cap and was placed under vacuum. After 5 minutes, the vial was flushed with nitrogen and the cycle was repeated three times. Under nitrogen, the vial was then charged with dry isopropanol (1 mL), placed on a preheated heating block at 130 °C and stirred for 24 h. The mixture was then cooled and the cap was removed followed by the addition of mesitylene (70 µL, 60 mg, 1.00 mmol) and it was stirred for 2 minutes. Saturated aqueous NH_4Cl (1 mL) was then added and the mixture was stirred for further 2 minutes, followed by the addition of EtOAc (1 mL) and stirring for 2 minutes. Then the mixture was left to settle for 5 minutes, the top layer was sampled and analysed using ^{1}H NMR. Yield determined by crude ^{1}H NMR using 1,3,5-trimethylbenzene as internal standard: 68%

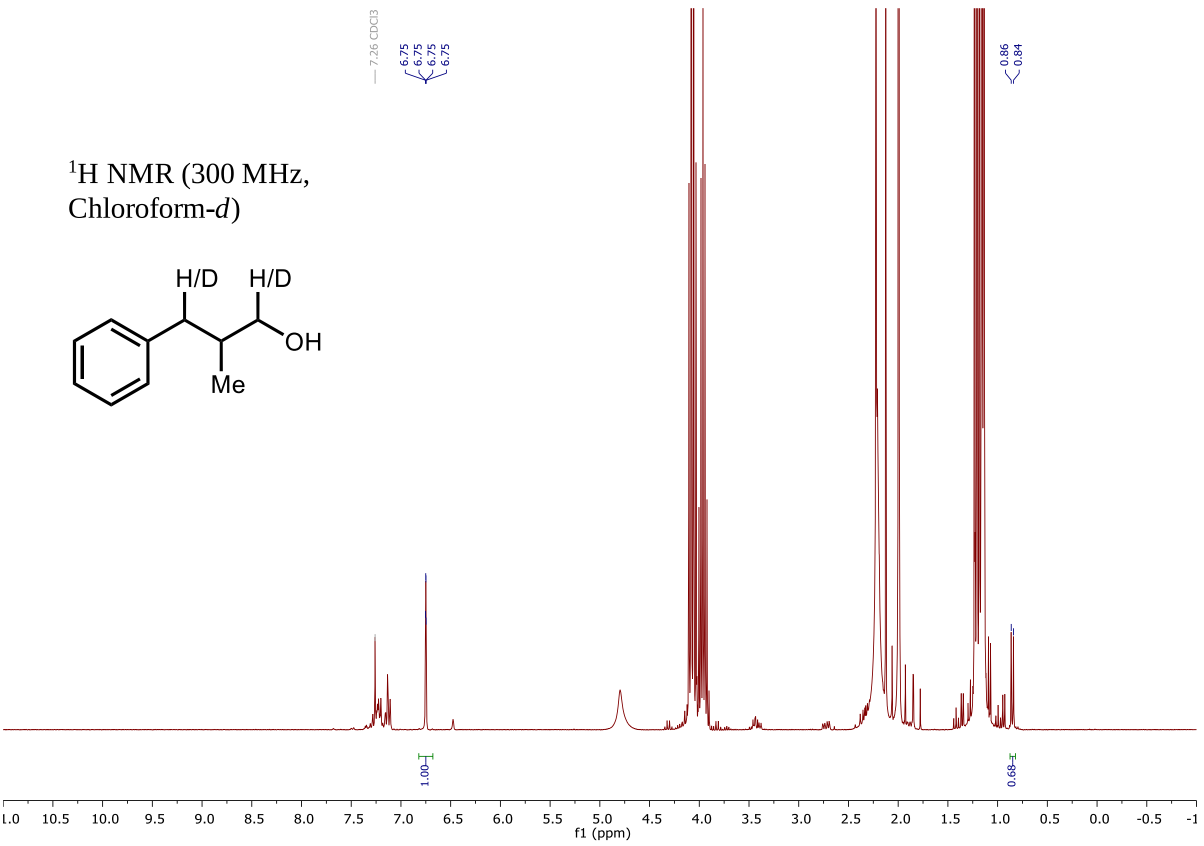

The mixture was diluted with EtOAc (12 mL), and transferred to a separatory funnel containing brine (12 mL). The aqueous phase was extracted with EtOAc (2 × 12 mL). The combined organic layers were dried over $MgSO_4$, filtered and concentrated in vacuo. Deuterium incorporation was determined by ^1H NMR after purification by flash silica chromatography (15-20% EtOAc in petroleum ether, 35 × 160 mm silica).

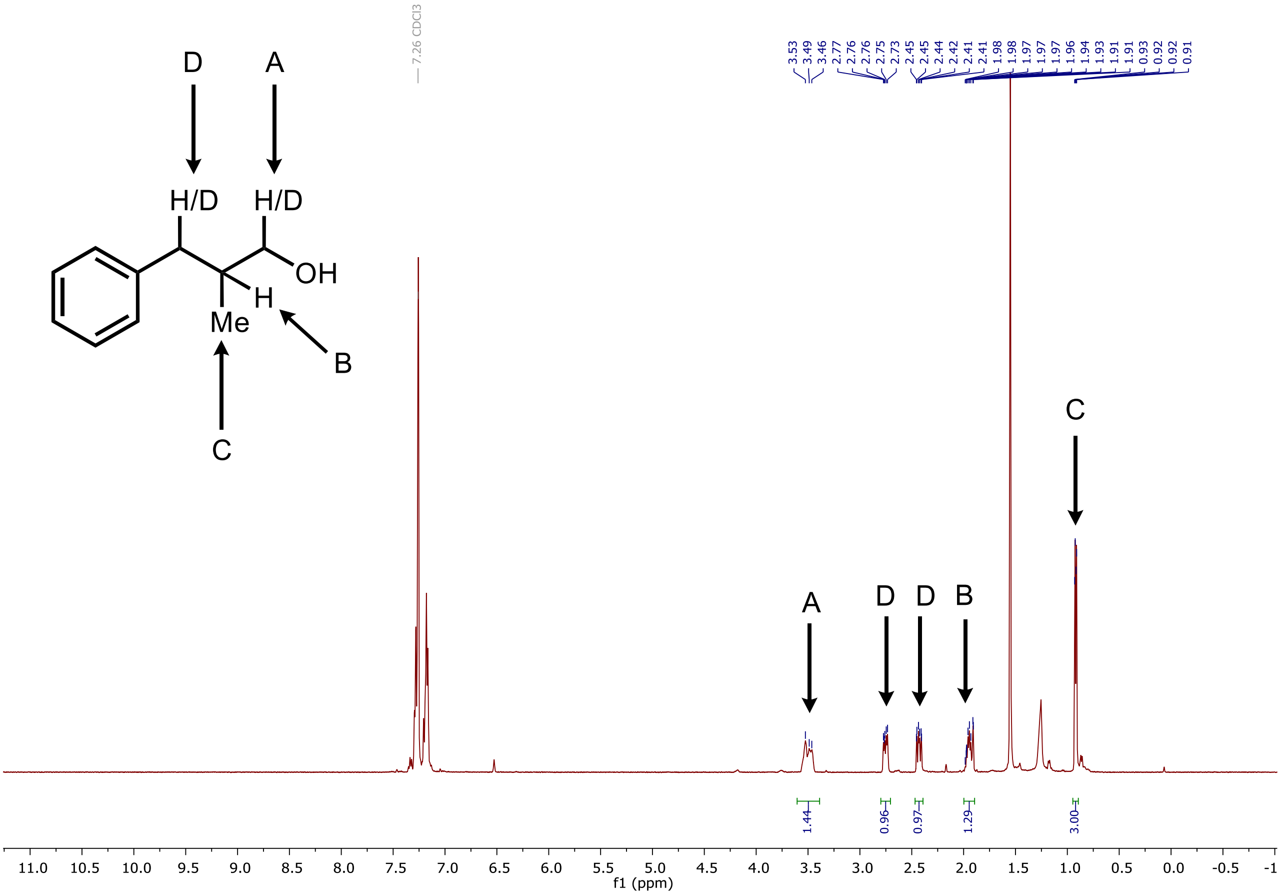

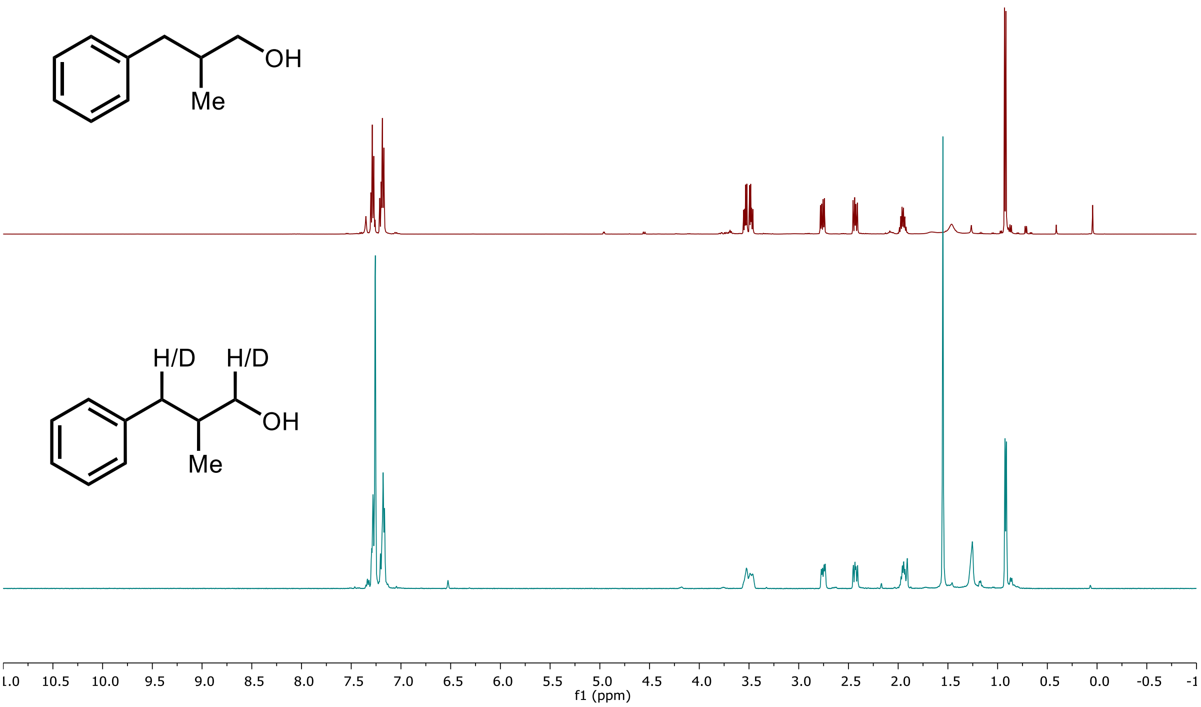

References

1. M. B. Dambatta, K. Polidano, A. D. Northey, J. M. J. Williams, L. C. Morrill, *ChemSusChem* **2019**, 12, 2345–2349.
2. T. N. Plank, J. L. Drake, D. K. Kim, T. W. Funk, *Adv. Synth. Catal.* **2012**, 354, 597–601.
3. T. W. Funk, A. R. Mahoney, R. A. Sponenburg, K. P. Zimmerman, D. K. Kim, E. E. Harrison, *Organometallics* **2018**, 37, 1133–1140.
4. S. Moulin, A. Pagnoux-ozherelyeva, *Chem. Eur. J.* **2013**, 19, 17881–17890.
5. C. C. Bausch, R. L. Patman, B. Breit, M. J. Krische, *Angew. Chem. Int. Ed.* **2011**, 50, 5687–569.
6. A. V. Malkov, L. Czemerys, D. A. Malyshev, *J. Org. Chem.* **2009**, 74, 3350–3355.
7. Q. Wang, X. Liu, X. Liu, B. Li, H. Nie, S. Zhang, W. Chen, *Chem. Commun.* **2014**, 50, 978–980.
8. W. Xu, R. Wang, G. Wu, P. Chen, *RSC Advances* **2012**, 2, 6005–6010.
9. M. Flinker, H. Yin, R. W. Juhl, E. Z. Eikeland, J. Overgaard, D. U. Nielsen, T. Skrydstrup, *Angew. Chem. Int. Ed.* **2017**, 56, 15910 –15915.

10. T. H. West, D. M. Walden, J. E. Taylor, A. C. Brueckner, R. C. Johnston, P. Cheong, G. C. Lloyd-Jones, A. D. Smith, *J. Am. Chem. Soc.* **2017**, 139, 4366–4375.
11. A. Kondoh, N. Tasato, T. Aoki, M. Terada, *Org. Lett.* **2020**, 22, 5170–5175.
12. C. Li, H. Chen, J. Li, M. Li, J. Liao, W. Wu, H. Jiang, *Adv. Synth. Catal.* **2018**, 360, 1600–1604.
13. N. M. Weldy, A. G. Schafer, C. P. Owens, C. J. Herting, A. Varela-Alvarez, S. Chen, Z. Niemeyer, D. G. Musaev, M. S. Sigman, H. M. L. Davies, S. B. Blakey, *Chem. Sci.* **2016**, 7, 3142–3146.
14. C. Bérubé, C. Carpentier, N. Voye, *Tetrahedron Letters* **2017**, 58, 2334–2336.
15. P. V. Kattamuri, G. Julian, J. G. West, *J. Am. Chem. Soc.* **2020**, 142, 19316–19326.
16. D. L. Bymaster, R. E. Pickering, T. K. Dobbs, E. J. Eisenbraun, *J. Label. Compd. Radiopharm.* **1986**, 23, 657-665.
17. J. A. Varela, B. Goldfuss, D. Denisenko, J. Kulhanek, K. Polborn, P. Knochel, *Chem. Eur. J.* **2004**, 10, 4252–4264.
18. S. M. A. H. Siddiki, A. S. Touchy, Md. A. R. Jamil, T. Toyao, K. Shimizu, *ACS Catal.* **2018**, 8, 3091–3103.
19. A. Maleckis, E. H. Abdelkader, G. Otting, I. D. Herath, *Org. Biomol. Chem.* **2022**, 20, 2424–2432.
20. C. Wang, Z. Xing, Q. Ge, Y. Yu, M.Wang, W. -L. Duan, *Org. Chem. Front.* **2022**, 9, 4316.
